# Terminal Cyclohexane-Type Meroterpenoids from the Fruiting Bodies of *Ganoderma cochlear*

**DOI:** 10.3389/fchem.2021.783705

**Published:** 2021-12-03

**Authors:** Fu-Ying Qin, Te Xu, Yan-Peng Li, Hao-Xing Zhang, Dan Cai, Li-Zhong Liu, Yong-Xian Cheng

**Affiliations:** ^1^ School of Pharmaceutical Sciences, School of Medicine, College of Life Sciences and Oceanography, Health Science Center, Institute for Inheritance-Based Innovation of Chinese Medicine, Shenzhen University, Shenzhen, China; ^2^ Guangdong Key Laboratory for Functional Substances in Medicinal Edible Resources and Healthcare Products, School of Life Sciences and Food Engineering, Hanshan Normal University, Chaozhou, China

**Keywords:** *Ganoderma cochlear*, meroterpenoids, renal fibrosis, triple negative breast cancer, BT549 cells

## Abstract

Eleven new cyclohexane-type meroterpenoids (**1**, **3**–**5**, **7**, **8**, **11**–**15**) and four known similar meroterpenoids (**2**, **6**, **9**, and **10**) were isolated from *Ganoderma cochlear.* Their structures and absolute configurations at stereogenic centers were elucidated by using HRESIMS, NMR spectroscopy and computational methods. In addition, the structure of the known meroterpenoid, cochlearol G (**2**), was revised, and the absolute configurations at the stereogenic centers of known meroterpenoids **9** and **10** were determined. All the isolated meroterpenoids were evaluated for their activities against renal fibrosis and triple negative breast cancer, and their insulin resistance. The results of the renal fibrosis study showed that meroterpenoid **11** inhibits over-expression of fibronectin, collagen I and *α*-SMA. Results of the wound healing study revealed that **4**, **6** and **8** significantly inhibit migration of BT549 cells. Observations made in Western blotting experiments showed that **6** decreases the levels of TWIST1 and ZEB1, and increases the level of E-cadherin. Finally, meroterpenoids **7**, **9**, **11**, and **15** significantly up-regulate p-AMPK protein expression in normal L6 myotubes cells.

## Introduction

*Ganoderma* is not only a famous Chinese medicine, it is also used globally as a food, in the form of tea, coffee and other beverages, and in syrups and dietary supplements (Wang et al., 2020; Kumar, 2021). Polysaccharides and triterpenoids are representative of the important biologically active components of *Ganoderma* (Wang et al., 2020). Recent ongoing research studies exploring *Ganoderma* demonstrated that it contains meroterpenoid components that possess extensive biological activities, such as renal protection and neuroprotection, anti-inflammation, -tumor and -oxidation properties, and analgesic effects (Jiang et al., 2021). These studies led to a deeper understanding of the components of *Ganoderma* and insight into active ingredients responsible for its traditional medical properties.

*Ganoderma* meroterpenoids comprise a class of substances with great potential, not only because they contain a variety of structural subtypes, but also because many members possess a host of biological activities. For instance, in 2009 ganomycin I was found to inhibit HIV-1 protease, and then in 2014 it was shown to have inhibitory effects on the production of monocyte chemotactic protein 1 (MCP-1) and fibronectin. Following these discoveries, ganomycin I was observed to inhibit NSC proliferation in 2015, and then in 2017 it was discovered to display hypoglycemic, hypolipidemic and insulin-sensitizing effects (Dine et al., 2009; Luo et al., 2015; Yan et al., 2015; Wang et al., 2017). As a result, we have been engaged in a program to isolate and identify new *Ganoderma* meroterpenoids and to assess their unique biological activities. In a previous effort, we found that these substances have inhibitory effects on renal fibrosis (Luo et al., 2015; Meng et al., 2021). In the current investigation, we isolated fifteen terminal cyclohexane-type meroterpenoids (**1**–**15**) from *Ganoderma cochlear* and evaluated their biological activities against renal fibrosis and insulin resistance. Breast cancer is one of the malignant cancer, and the morbidity and mortality is highest in women, with triple negative breast cancer (TNBC) being. Based on the fact that *Ganoderma* has been used to treat cancer, we also investigated the activities of the meroterpenoids against cells of triple negative breast cancer, which is difficult to treat cancers and has an extremely high mortality rate (Collignon et al., 2016). The results of this study are described below.

## Materials and Methods

### General

Optical rotations were determined using an Anton Paar MCP-100 digital polarimeter. UV and CD spectra were recorded on a Chirascan instrument. NMR spectra were obtained by using a Bruker Avance Ⅲ 600 MHz or 500 MHz spectrometer, with TMS as an internal standard. All NMR chemical shifts are given in ppm. HRESIMS were recorded using a Shimazu LC-20AD AB SCIEX triple TOF 6600+ MS spectrometer (Shimadzu Corporation, Tokyo, Japan). MCI gel CHP 20P (75–150 *μ*m, Mitsubishi Chemical Industries, Tokyo, Japan), C-18 silica gel (40–60 μm; Daiso Co., Japan), and Sephadex LH-20 (Amersham Pharmacia, Uppsala, Sweden) were used for column chromatography. Silica gel (Qingdao Marine Chemical Inc., Qingdao, China) was used for vacuum column chromatography (VLC). Preparative HPLC was carried out using a Saipuruisi chromatograph equipped with a Thermo Hypersil GOLD-C18 column (250 × 21.2 mm, i. d., 5 *μ*m). Semi-preparative HPLC was carried out using a Saipuruisi chromatograph with a YMC-Pack ODS-A column (250 × 10 mm, i. d., 5 *μ*m). Chiral HPLC analysis was run using an Agilent 1260 chromatograph with a Daicel Chiralpak column (IC, 250 mm × 10 mm, i. d., 5 *μ*m) or a Daicel Chiralpak column (IC, 250 mm × 4.6 mm, i. d., 5 *μ*m).

### Fungal Material

The dried fruiting bodies of *G. cochlear* were purchased from Tongkang Pharmaceutical Co. Ltd. Guangdong province, China, in July 2014. This fungus was authenticated by Prof. Zhu-Liang Yang at Kunming Institute of Botany, Chinese Academy of Sciences, China, and a voucher specimen (CHYX-0589) is deposited at Institute for Inheritance-Based Innovation of Chinese Medicine, Shenzhen University Health Science Center, China.

### Extraction and Isolation

Powders of *G. cochlear* (200 kg) fruiting bodies were extracted with refluxing 80% EtOH (3 *×* 120 L, 4, 3, 3 h) and the extract was concentrated under reduced pressure to afford a crude residue. An aliquot (8 kg of the residue corresponding to 95 kg fungal material) was suspended in water and extracted three times with EtOAc, followed by concentration of the combined extracts to afford an EtOAc soluble residue (4 kg). The residue was subjected to silica gel column using an eluant comprised of increasing amounts of acetone in petroleum ether to provide four parts (Fr.1–Fr.4). Fr.2 (860 g) was further divided into six parts (Fr.2.1–Fr.2.6) by MCI gel CHP 20P (MeOH/H_2_O, 60–100%). Fr.2.2 (120.0 g) was divided into five parts (Fr.2.2.1−Fr.2.2.5) by using RP-18 column chromatography (eluting solvent: MeOH/H_2_O, 40–100%). Fr.2.2.3 (21.0 g) was subjected to a MCI gel CHP 20P column chromatography (MeOH/H_2_O, 40–100%) to obtain five parts (Fr.2.2.3.1−Fr.2.2.3.5). Fr.2.2.3.3 (3.7 g) was gel filtered through Sephadex LH-20 (MeOH) to produce three parts (Fr.2.2.3.3.1−Fr.2.2.3.3.3.). Fr.2.2.3.3.1 (1.9 g) was subjected to preparative HPLC (MeOH/H_2_O, 65–100%) to produce three parts (Fr.2.2.3.3.1.1−Fr.2.2.3.3.1.3). Semi-preparative HPLC (eluting solvent: MeOH/H_2_O containing 0.05% TFA, 60–100%, flow rate: 3 mL/min) of Fr.2.2.3.3.1.2 (0.9 g) gave three subfractions (Fr.2.2.3.3.1.2.1−Fr.2.2.3.3.1.2.3). Semi-preparative HPLC (eluting solvent: MeOH/H_2_O containing 0.05% TFA, 70%, flow rate: 3 mL/min) of subfraction Fr.2.2.3.3.1.2.3 (300 mg) yielded **12** (5.6 mg, t_R_ = 20.5 min). Semi-preparative HPLC (eluting solvent: MeOH/H_2_O containing 0.05% TFA, 65%, flow rate: 3 mL/min) of Fr.2.2.3.3.1.3 (200 mg) produced subfraction Fr.2.2.3.3.1.3.3 (13.1 mg, t_R_ = 20.7 min), which by using chiral Daicel Chialpak IC column (n-hexane/ethanol containing 0.05% TFA, 92:8, flow rate: 1 mL/min) yielded **14** (4.0 mg, t_R_ = 13.9 min) and **15** (5.9 mg, t_R_ = 20.0 min).

Fr.3 (780 g) was fractionated into eight parts (Fr.3.1–Fr.3.8) by using a MCI gel CHP 20P column chromatography (MeOH/H_2_O, 40–100%). Fr.3.4 (120 g) was divided into three portions (Fr.3.4.1−Fr.3.4.3) by using RP-18 column chromatography (MeOH/H_2_O, 20%–100%). Submission of Fr.3.4.1 (115 g) to a MCI gel CHP 20P column chromatography (MeOH/H_2_O, 40–100%) provided six portions (Fr.3.4.1.1–Fr.3.4.1.6). Fr.3.4.1.3 (21.6 g) was separated by using Sephadex LH-20 (MeOH) into four parts (Fr.3.4.1.3.1–Fr.3.4.1.3.4). Fr.3.4.1.3.3 (6.5 g) was subjected to RP-18 (50 *µ*m) column chromatography to obtain five parts (Fr.3.4.1.3.3.1–Fr.3.4.1.3.3.5). Fr.3.4.1.3.3.1 (1.1 g) was gel filtered through Sephadex LH-20 (MeOH) to produce two subfractions (Fr.3.4.1.3.3.1.1 and Fr.3.4.1.3.3.1.2). Fr.3.4.1.3.3.1.1 (0.7 g) was separated into three portions by using preparative TLC (CH_2_Cl_2_:MeOH = 7:1). Submission of Fr.3.4.1.3.3.1.1.2 (Rf = 0.6, 100 mg) to semi-preparative HPLC (acetonitrile:MeOH:H_2_O containing 0.05% TFA, 25.5:25.5:49.0, flow rate: 3 mL/min) yielded **2** (34.8 mg, t_R_ = 18.5 min) and **1** (11.4 mg, t_R_ = 20.0 min). Subjection of Fr.3.4.1.3.3.1.1.3 (Rf = 0.8, 200 mg) to chiral Daicel Chialpak IC column chromatography (n-hexane/ethanol containing 0.05% TFA, 91:9, flow rate: 3 mL/min) yielded **13** (76.9 mg, t_R_ = 16.0 min). Fr.3.4.1.3.3.3 (1.4 g) was filtered through Sephadex LH-20 (MeOH) to produce four fractions (Fr.3.4.1.3.3.3.1–Fr.3.4.1.3.3.3.4). Fr.3.4.1.3.3.3.1 (79 mg) was subjected to semi-preparative HPLC (MeOH:H_2_O containing 0.05% TFA, 62%, flow rate: 3 mL/min) to yield **11** (7.2 mg, t_R_ = 37.4 min). Fr.3.4.1.3.3.3.3 (0.8 g) was subjected to preparative HPLC (acetonitrile:H_2_O containing 0.05% TFA, 42%, flow rate: 8 mL/min) to generate six fractions (Fr.3.4.1.3.3.3.3.1–Fr.3.4.1.3.3.3.3.6). Fr.3.4.1.3.3.3.3.2 (178.3 mg) was subjected to chiral Daicel Chiralpak IC column chromatography (n-hexane/isopropanol containing 0.05% TFA, 91:9, flow rate: 3 mL/min) to yield **7** (18.3 mg, t_R_ = 34.6 min) and **8** (18.0 mg, t_R_ = 47.2 min). Fr.3.4.1.3.3.3.3.3 (0.4 g) subjected to chiral Daicel Chiralpak IC column chromatography (n-hexane/ethanol containing 0.05% TFA, 90:10) to produce **9** (8.82 mg, t_R_ = 32.5 min) and **10** (6.15 mg, t_R_ = 24.0 min).

Fr.3.4.1.3.3.4 (1.0 g) was filtered through Sephadex LH-20 (MeOH) to produce four fractions (Fr.3.4.1.3.3.4.1–Fr.3.4.1.3.3.4.4). Fr.3.4.1.3.3.4.4 (110 mg) was subjected to semi-preparative HPLC (MeOH:H_2_O containing 0.05% TFA, 60%, flow rate: 3 mL/min) to form four fractions, the fourth fraction of which was subjected to preparative HPLC (acetonitrile:MeOH:H_2_O containing 0.05% TFA, 28:28:44, flow rate: 12 mL/min) to yield **6** (37.4 mg, t_R_ = 24.2 min).

Fr.3.4.1.1 (39.4 g) was gel filtered though Sephadex LH-20 (MeOH) and then subjected to C-18 silica gel column chromatography (MeOH/H_2_O, 20–100%) to produce eight fractions (Fr.3.4.1.1.1–Fr.3.4.1.1.8). Fr.3.4.1.1.5 (10 g) was separated into seven parts (Fr.3.4.1.1.5.1–Fr.3.4.1.1.5.7) by using silica gel column chromatography using an eluant comprised of increasing amounts of methanol in dichloromethane. Fr.3.4.1.1.5.5 (2.0 g) was gel filtered through Sephadex LH-20 (MeOH) to obtain five fractions (Fr.3.4.1.1.5.5.1-Fr.3.4.1.1.5.5.5). Fr.3.4.1.1.5.5.5 (0.6 g) was subjected to semi-preparative HPLC (MeOH:H_2_O containing 0.05% TFA, 55%, flow rate: 3 mL/min) to produce four subfractions (Fr.3.4.1.1.5.5.5.1–Fr.3.4.1.1.5.5.5.4). Fr.3.4.1.1.5.5.5.2 (116 mg) was subjected to semi-preparative HPLC (acetonitrile:MeOH:H_2_O containing 0.05% TFA, 19:19:62, flow rate: 3 mL/min) to yield **3** (10.6 mg, t_R_ = 24.2 min). Fr.3.4.1.1.5.5.4 (0.3 g) was divided into two subfractions by using semi-preparative HPLC (MeOH:H_2_O containing 0.05% TFA, 55%, flow rate: 3 mL/min), the first subfraction was subjected to chiral Daicel Chiralpak IC column chromatography (n-hexane/ethanol containing 0.05% TFA, 90:10) to yield **4** (18.2 mg, t_R_ = 19.7 min) and **5** (17.6 mg, t_R_ = 24.1 min).

Meroterpenoid **6** (2.5 mg) was also isolated from 40 kg *Ganoderma lucidum* cultivated in Yongsheng County of Yunnan Province, China. This material was also authenticated by Prof. Zhu-Liang Yang at Kunming Institute of Botany, Chinese Academy of Sciences, China. A voucher specimen (CHYX-0609) is deposited at Institute for Inheritance-Based Innovation of Chinese Medicine, Health Science Center, Shenzhen University, China.

### Compound Characterization

*Ganodercin G* (***1***), yellow gum; [α]_D_^20^ –14.5 (*c* 0.06, MeOH); UV (MeOH) *λ*_max_ (log *ε*) 362 (3.40), 201 (4.29) nm; CD (MeOH) *Δ* *ε*_202_–1.83, HRESIMS *m/z* 375.1812 [M + H]^+^, (calcd for C_21_H_27_O_6_, 375.1802), ^1^H and ^13^C NMR data, see Tables 1 and 2.

**TABLE 1:** ^1^H NMR (500 MHz) data of **1**, **3**–**5**, **7**, and **8** (*δ* in ppm, *J* in Hz).

	1	3	4	5	7	8
No	*δ*_H_ ^*a*^	*δ*_H_ ^*a*^	*δ*_H_ ^*a*^	*δ*_H_ ^*a*^	*δ*_H_ ^*b*^	*δ*_H_ ^*b*^
3′	7.37 d (2.2)	7.36 d (2.9)	6.58 d (3.0)	6.58 d (3.0)	7.42 d (3.0)	7.42 d (3.0)
5′	7.02 dd (8.9, 2.2)	7.02 overlap	6.50 dd (8.6, 3.0)	6.49 dd (8.6, 3.0)	7.10 dd (8.9, 3.0)	7.10 dd (8.9, 3.0)
6′	6.81 d (8.9)	6.81 d (8.9)	6.61 d (8.6)	6.60 d (8.6)	6.81 d (8.9)	6.81 d (8.9)
1	—	—	Ha: 3.73 dd (15.0, 8.3) Hb: 3.54 dd (15.0, 7.3)	Ha: 3.67 dd (15.6, 7.8) Hb: 3.65 dd (15.6, 8.3)	—	—
2	4.12 s	Ha: 4.09 d (18.2); Hb: 4.05 d (18.2)	5.89 t-like (7.8)	6.02 t-like (7.7)	Ha: 3.56 dd (17.9, 9.3) Hb: 3.24 dd (17.9, 4.6)	Ha: 3.57 dd (17.9, 9.3) Hb: 3.23 dd (17.9, 4.5)
3	—	—	—	—	3.05 m	3.06 m
4	6.90 t (6.4)	7.02 overlap	Ha: 2.47 m; Hb: 2.03 m	Ha: 2.52 m; Hb: 2.23 m	Ha: 1.82 m; Hb: 1.78 m	Ha: 1.82 m; Hb: 1.74 m
5	Ha: 2.97 dd (17.7, 6.4) Hb: 2.91 dd (17.7, 6.4)	2.45 m	Ha: 1.71 m; Hb: 1.63 m	Ha: 1.80 m; Hb: 1.39 m	Ha: 2.22 td (13.0, 5.6) Hb: 2.12 td (13.0, 4.6)	2.17 m
6	—	1.94 dd (9.8, 4.3)	1.66 brs	1.66 brs	—	—
8	2.07 m	Ha: 2.35 dt (12.4, 4.7) Hb: 2.07 td (12.4, 4.7)	Ha: 2.23 dt (12.5, 4.4); Hb: 1.84 td (12.5, 4.4)	5.22 brs	2.01 dd (12.6, 6.2)	2.01 dd (12.4, 5.7)
9	Ha: 1.75 m; Hb: 1.30 m	Ha: 1.81 m; Hb: 1.52 m	Ha: 1.75 m; Hb: 1.44 m	Ha: 2.11 m; Hb: 1.94 m	Ha: 1.73 m; Hb: 1.66 m	Ha: 1.73 m; Hb: 1.67 m
10	3.45 dd (10.0, 2.9)	3.38 dd (9.9, 4.2)	3.29 dd (10.4, 4.3)	3.36 dd (8.9, 5.7)	3.41 dd (10.3, 3.4)	3.41 dd (10.3, 3.4)
12	1.04 s	1.01 s	0.97 s	0.94 s	1.09 s	1.09 s
13	0.96 s	0.72 s	0.64 s	0.76 s	0.98 s	0.98 s
14	1.58 s	Ha: 4.90 s; Hb: 4.59 s	Ha: 4.84 s; Hb: 4.61 s	1.70 s	1.61 s	1.61 s
OH-1′	—	—	—	—	11.59 s	11.59 s

a Record in methanol-d_4_.

b Record in acetone-d_6_.

**TABLE 2:** ^13^C NMR data of **1**, **3**–**5**, **7**–**15** (*δ* in ppm).

	1	3	4	5	7	8	9	10	11	12	13	14	15
No	*δ*_C_ ^*a*^	*δ*_C_ ^*a*^	*δ*_C_ ^*a*^	*δ*_C_ ^*a*^	*δ*_C_ ^*b*^	*δ*_C_ ^*b*^	*δ*_C_ ^*b*^	*δ*_C_ ^*b*^	*δ*_C_ ^*a*^	*δ*_C_ ^*c*^	*δ*_C_ ^*a*^	*δ*_C_ ^*a*^	*δ*_C_ ^*a*^
1′	150.7 s	150.7 s	149.3 s	149.2 s	149.3 s	149.3 s	149.3 s	149.3 s	150.7 s	150.9 s	150.5 s	150.7 s	150.7 s
2′	120.6 s	120.6 s	127.9 s	128.2 s	119.1 s	119.1 s	119.1 s	119.1 s	120.4 s	121.3 s	120.4 s	120.4 s	120.5 s
3′	115.7 d	115.6 d	117.8 d	117.7 d	114.6 d	114.6 d	114.6 d	114.6 d	115.4 d	115.9 d	115.6 d	115.4 d	115.4 d
4′	156.5 s	156.5 s	151.2 s	151.2 s	155.3 s	155.3 s	155.3 s	155.3 s	156.5 s	157.2 s	156.4 s	156.5 s	156.5 s
5′	125.8 d	125.9 d	114.8 d	114.8 d	124.7 d	124.8 d	124.7 d	124.7 d	126.0 d	126.8 d	125.8 d	125.9 d	125.9 d
6′	119.7 d	119.7 d	116.9 d	117.9 d	118.5 d	118.5 d	118.5 d	118.5 d	119.7 d	120.0 d	119.6 d	119.7 d	119.7 d
1	204.1 s	204.3 s	31.7 t	31.7 t	204.8 s	204.8 s	204.8 s	204.9 s	205.5 s	199.2 s	203.9 s	205.6 s	205.6 s
2	37.6 t	37.7 t	140.5 d	141.1 d	39.6 t	39.6 t	40.2 t	39.6 t	41.8 t	132.8 d	37.7 t	41.5 t	41.2 t
3	126.6 s	127.0 s	133.6 s	133.9 s	40.3 d	40.2 d	40.0 d	39.9 d	41.7 d	146.3 s	127.4 s	41.9 d	41.8 d
4	148.6 d	148.5 d	34.6 t	37.9 t	32.1 t	32.1 t	31.2 t	31.1 t	32.5 t	29.8 t	147.7 d	33.3 t	33.1 t
5	29.4 t	26.9 t	25.4 t	29.3 t	26.2 t	26.2 t	23.1 t	23.0 t	24.1 t	28.3 t	28.6 t	26.4 t	26.2 t
6	135.0 s	52.8 d	51.4 d	50.2 d	135.8 s	135.8 s	52.1 d	52.2 d	52.9 d	57.1 d	56.8 d	56.8 d	56.8 d
7	129.9 s	148.7 s	148.8 s	137.8 s	126.3 s	126.3 s	148.2 s	148.1 s	149.0 s	88.6 s	88.3 s	88.6 s	88.6 s
8	31.6 t	33.9 t	34.6 t	120.1 d	30.4 t	30.4 t	32.5 t	32.5 t	34.1 t	39.7 t	39.2 t	39.8 t	39.8 t
9	27.8 t	32.8 t	33.1 t	32.5 t	27.1 t	27.1 t	32.2 t	32.2 t	33.1 t	26.5 t	26.7 t	26.6 t	26.6 t
10	76.6 d	77.5 d	77.9 d	75.6 d	74.8 d	74.8 d	75.7 d	75.6 d	77.7 d	87.6 d	87.5 d	87.7 d	87.7 d
11	41.3 s	41.5 s	41.6 s	39.3 s	40.1 s	40.1 s	40.4 s	40.4 s	41.9 s	46.4 s	46.2 s	46.3 s	46.4 s
12	26.2 q	26.3 q	26.2 q	25.7 q	25.4 q	25.4 q	25.7 q	26.4 q	26.5 q	18.9 q	26.1 q	26.3 q	26.2 q
13	21.5 q	16.0 q	15.5 q	15.8 q	20.8 q	20.9 q	15.7 q	15.8 q	16.1 q	26.0 q	24.2 q	23.7 q	23.7 q
14	19.9 q	110.0 t	108.6 t	22.7 q	18.9 q	18.9 q	107.6 t	107.6 t	108.7 t	23.7 q	19.1 q	19.0 q	19.0 q
15	170.5 s	170.5 s	172.3 s	172.0 s	175.4 s	175.5 s	175.6 s	175.6 s	177.7 s	170.2 s	170.3 s	179.0 s	178.9 s
OCH_3_	—	—	—	—	—	—	—	—	52.2 q	—	—	—	—

a Record in methanol-d_4_ at 125 MHz.

b Record in acetone-d_6_ at 125 MHz.

c Record in methanol-d_4_ at 150 MHz.

*Ganodercin H (* ***3*** *)*, yellow gum; [α]_D_^20^ +10.6 (*c* 0.09, MeOH); CD (MeOH) *Δ* *ε*_361_–1.52, *Δ* *ε*_211_ +1.18; UV (MeOH) *λ*_max_ (log *ε*) 361 (3.52), 261 (3.74), 216 (4.26) nm, HRESIMS *m/z* 375.1804 [M + H]^+^, (calcd for C_21_H_27_O_6_, 375.1802), ^1^H and ^13^C NMR data, see Tables 1 and 2.

*Ganodercin I *(***4***), yellow gum; [α]_D_^20^ +37.6 (*c* 0.11, MeOH); UV (MeOH) *λ*_max_ (log *ε*) 297 (3.66), 223 (4.19), 202 (4.33) nm, CD (MeOH) *Δ* *ε*_299_ +0.83, *Δ* *ε*_260_–0.31, *Δ* *ε*_216_ +4.43, HRESIMS *m/z* 361.2012 [M + H]^+^, (calcd for C_21_H_29_O_5_, 361.2010), ^1^H and ^13^C NMR data, Tables 1 and 2.

*Ganodercin J *(***5***), yellow gum; [α]_D_^20^ –5.9 (*c* 0.10, MeOH); UV (MeOH) *λ*_max_ (log *ε*) 296 (3.54), 222 (3.83), 201 (4.31) nm, CD (MeOH) *Δ* *ε*_204_ +2.89, HRESIMS *m/z* 361.2008 [M + H]^+^, (calcd for C_21_H_29_O_5_, 361.2010), ^1^H and ^13^C NMR data, see Tables 1 and 2.

*Ganodercin K *(***7***), yellow gum; [α]_D_^20^ –35.0 (*c* 0.08, MeOH); CD (MeOH) *Δ* *ε*_257_–0.40, *Δ* *ε*_231_–1.38, *Δ* *ε*_215_ +1.62, *Δ* *ε*_200_–4.86; UV (MeOH) *λ*_max_ (log *ε*) 364 (3.55), 256 (3.80), 225 (4.14), 201 (4.21) nm, HRESIMS *m/z* 377.1964 [M + H]^+^, (calcd for C_21_H_29_O_6_, 377.1959), ^1^H and ^13^C NMR data, see Tables 1 and 2.

*3-epi-Ganodercin K* (***8***), yellow gum; [α]_D_^20^ –5.0 (*c* 0.02, MeOH); CD (MeOH) *Δ* *ε*_259_ + 0.32, *Δ* *ε*_227_ +1.38, *Δ* *ε*_216_ +2.18, *Δ* *ε*_201_–3.21; UV (MeOH) *λ*_max_ (log *ε*) 364 (3.61), 256 (3.87), 225 (4.21), 201 (4.30) nm, HRESIMS *m/z* 377.1964 [M + H]^+^, (calcd for C_21_H_29_O_6_, 377.1959), ^1^H and ^13^C NMR data, see Tables 1 and 2.

*Ganodercin L* (***9***), yellow gum; [α]_D_^20^ +12.0 (*c* 0.03, MeOH); CD (MeOH) *Δ* *ε*_255_ +0.38, *Δ* *ε*_228_ +1.48, *Δ* *ε*_205_ +0.75, *Δ* *ε*_196_–1.02; UV (MeOH) *λ*_max_ (log *ε*) 363 (3.48), 256 (3.72), 226 (4.00), 202 (4.01) nm, HRESIMS *m/z* 377.1961 [M + H]^+^, (calcd for C_21_H_29_O_6_, 377.1959), ^1^H and ^13^C NMR data, see Tables 2 and 3.

**TABLE 3:** ^1^H NMR data of **9**–**15** in methanol-*d*_4_ (*δ* in ppm, *J* in Hz).

	9	10	11	12	13	14	15
No	*δ*_H_ ^*a*^	*δ*_H_ ^*a*^	*δ*_H_ ^*b*^	*δ*_H_ ^*c*^	*δ*_H_ ^*b*^	*δ*_H_ ^*b*^	*δ*_H_ ^*b*^
3′	7.40 d (2.9)	7.42 d (2.9)	7.23 d (2.9)	7.15 d (3.0)	7.35 d (3.0)	7.26 d (2.9)	7.26 d (2.9)
5′	7.10 dd (8.9, 2.9)	7.09 dd (8.9, 2.9)	7.00 dd (8.9, 2.9)	7.05 dd (8.9, 3.0)	7.01 dd (8.9, 3.0)	7.01 dd (8.9, 2.9)	7.01 dd (8.9, 3.0)
6′	6.81 d (8.9)	6.81 d (8.9)	6.79 d (8.9)	6.84 d (8.9)	6.80 d (8.9)	6.79 d (8.9)	6.79 d (8.9)
2	Ha: 3.51 dd (17.8, 9.3); Hb: 3.17 dd (17.8, 4.5)	Ha: 3.52 dd (17.9, 9.6); Hb: 3.19 dd (17.9, 4.2)	Ha: 3.42 dd (17.9, 9.5); Hb: 3.15 dd (17.9, 4.6)	7.64 s	Ha: 4.09 d (18.0) Hb: 4.04 d (18.0)	Ha: 3.44 dd (17.9, 9.1); Hb: 3.14 dd (17.9, 4.8)	Ha: 3.44 dd (17.8, 9.1); Hb: 3.14 dd (17.8, 4.8)
3	3.02 m	3.03 m	2.97 m	—	—	2.95 m	2.97 m
4	Ha: 1.86 m; Hb: 1.44 m	Ha: 1.82 m; Hb: 1.54 m	Ha: 1.82 m; Hb: 1.38 m	Ha: 2.58 m; Hb: 2.52 m	7.07 t (6.8)	Ha: 1.68 m; Hb: 1.59 m	Ha: 1.69 m; Hb: 1.63 m
5	Ha: 1.73 m; Hb: 1.69 m	Ha: 1.74 m; Hb: 1.71 m	Ha: 1.60 m	Ha: 1.51 m; Hb: 1.46 m	2.20 m	1.47 m	Ha: 1.46 m; Hb: 1.36 m
6	1.74 brs	1.72 m	1.70 dd (8.3, 4.3)	1.26 t (7.3)	1.58 t (7.7)	1.29 dd (5.2, 3.1)	1.29 dd (8.2, 5.8)
8	Ha: 2.33 dt (12.9, 5.0); Hb: 2.00 dt (12.9, 4.8)	Ha: 2.34 dt (12.9, 5.0); Hb: 2.00 dt (12.9, 4.9)	Ha: 2.32 dt (12.9, 4.8); Hb: 2.00 dt (12.9, 4.3)	Ha: 1.51 m; Hb: 1.39 td (12.1, 4.5)	Ha: 1.62 m; Hb: 1.45 m	Ha: 1.59 m; Hb: 1.46 m	Ha: 1.59 m; Hb: 1.46 m
9	Ha: 1.80 m; Hb: 1.52 m	Ha: 1.80 m; Hb: 1.50 m	Ha: 1.80 m; Hb: 1.52 m	Ha: 1.94 m; Hb:1.63 m	Ha: 1.99 m; Hb: 1.67 m	Ha: 1.99 m; Hb: 1.67 m	Ha: 1.98 m; Hb:1.66 m
10	3.39 dd (9.4, 4.1)	3.39 dd (9.3, 4.1)	3.36 dd (9.9, 4.2)	3.70 brd (5.4)	3.41 dd (10.3, 3.4)	3.73 brd (5.3)	3.73 brd (5.3)
12	1.04 s	1.04 s	1.03 s	1.02 s	1.08 s	1.09 s	1.09 s
13	0.75 s	0.75 s	0.71 s	0.97 s	0.97 s	1.01 s	1.01 s
14	Ha: 4.85 s; Hb: 4.64 s	Ha: 4.86 s; Hb: 4.65 s	Ha: 4.86 s; Hb: 4.55 s	1.28 s	1.30 s	1.32 s	1.32 s
OCH_3_	—	—	3.68 s	—	—	—	—
OH-1′	11.59 s	11.59 s	—	—	—	—	—
OH-4′	8.15 s	8.15 s	—	—	—	—	—

a Record in acetone-d_6_ at 500 MHz.

b Record in methanol-d_4_ at 500 MHz.

c Record in methanol-d_4_ at 600 MHz.

*3-epi-Ganodercin L* (***10***), yellow gum; [α]_D_^20^ –17.3 (*c* 0.08, MeOH); CD (MeOH) *Δ* *ε*_256_–0.13, *Δ* *ε*_228_–0.49, *Δ* *ε*_201_ +0.31, *Δ* *ε*_192_–1.74; UV (MeOH) *λ*_max_ (log *ε*) 363 (3.35), 256 (3.59), 227 (3.88), 201 (3.89) nm, HRESIMS *m/z* 377.1964 [M + H]^+^, (calcd for C_21_H_29_O_6_, 377.1959), ^1^H and ^13^C NMR data, see Tables 2 and 3.

*Ganodercin M* (***11***), yellow gum; [α]_D_^20^ –16.7 (*c* 0.03, MeOH); CD (MeOH) *Δ* *ε*_256_–0.18, *Δ* *ε*_230_–0.70, *Δ* *ε*_202_ +0.66, *Δ* *ε*_192_–1.75; UV (MeOH) *λ*_max_ (log *ε*) 363 (3.28), 257 (3.58), 227 (3.88), 201 (3.89) nm, HRESIMS *m/z* 391.2120 [M + H]^+^, (calcd for C_22_H_31_O_6_, 391.2115), ^1^H and ^13^C NMR data, see Tables 2 and 3.

*Ganodercin N* (***12***), yellow gum; [α]_D_^20^ +22.5 (*c* 0.04, MeOH); UV (MeOH) *λ*_max_ (log *ε*) 386 (3.38), 267 (3.91), 227 (3.89), 200 (4.09) nm, HRESIMS *m/z* 375.1812 [M + H]^+^, (calcd for C_21_H_27_O_6_, 375.1802), ^1^H and ^13^C NMR data, see Tables 2 and 3.

*Ganodercin O* (***13***), yellow gum; [α]_D_^20^ +10.0 (*c* 0.06, MeOH); CD (MeOH) *Δ* *ε*_228_–1.19, *Δ* *ε*_216_–2.12, *Δ* *ε*_202_ +1.65; UV (MeOH) *λ*_max_ (log *ε*) 364 (3.49), 219 (4.34) nm, HRESIMS *m/z* 375.1805 [M + H]^+^, (calcd for C_21_H_27_O_6_, 375.1802), ^1^H and ^13^C NMR data, see Tables 2 and 3.

*Ganodercin P* (***14***), yellow gum; [α]_D_^20^ +24.0 (*c* 0.03, MeOH); CD (MeOH) *Δ* *ε*_254_ +0.28, *Δ* *ε*_229_ +0.83; UV (MeOH) *λ*_max_ (log *ε*) 363 (3.45), 256 (3.71), 226 (4.00) nm, HRESIMS *m/z* 377.1965 [M + H]^+^, (calcd for C_21_H_29_O_6_, 377.1959), ^1^H and ^13^C NMR data, see Tables 2 and 3.

*3-epi-Ganodercin P* (***15***), yellow gum; [α]_D_^20^ –4.0 (*c* 0.05, MeOH); CD (MeOH) *Δ* *ε*_256_–0.33, *Δ* *ε*_226_–1.24; UV (MeOH) *λ*_max_ (log *ε*) 363 (3.38), 256 (3.63), 227 (3.92) nm, HRESIMS *m/z* 377.1968 [M + H]^+^, (calcd for C_21_H_29_O_6_, 377.1959), ^1^H and ^13^C NMR data, see Tables 2 and 3.

Crystal Data for C_21_H_28_O_6_ (*M* = 376.43 g/mol): monoclinic, space group P2_1_ (no. 4), *a* = 6.62350(10) Å, *b* = 11.59270(10) Å, *c* = 13.40440(10) Å, *β* = 100.4300(10)°, *V* = 1012.240(19) Å^3^, *Z* = 2, *T* = 99.9(9) K, *μ*(CuK*α*) = 0.737 mm^−1^, *Dcalc* = 1.235 g/cm^3^, 9814 reflections measured (6.704° ≤ 2Θ ≤ 148.606°), 3973 unique (*R*_int_ = 0.0305, *R*_sigma_ = 0.0308) which were used in all calculations. The final *R*_1_ was 0.0324 (I > 2σ(I)) and *wR*_2_ was 0.0865 (all data). Crystallographic data of 3-*epi*-ganodercin P (**15**) have been deposited at the Cambridge Crystallographic Data Centre (CCDC deposition no. 2105988).

### Renal Fibrosis Activity Assay

TGF-*β*1 induced rat renal proximal tubular cells (NRK-52E) were used to assess expression of the target gene. The cell culture method, and cell viability and western blotting assays were conducted following our previously reported protocols (Meng et al., 2021).

### Biological Activity Assay on Triple Negative Breast Cancer Cell Lines (MDA-MB-231, BT549 and HCC 1806)

#### Cell Culture

MDA-MB-231 (ATCC, Cat No. HTB-26, United States), BT549 (ATCC, Cat No. HTB-122, United States) and HCC1806 (ATCC, Cat No. CRL-2335, United States) cells were cultured in Dulbecco’s Modified Eagle Medium (HyClone, Cat No.SH30243.01, United States) or RPMI Medium Modified (HyClone, Cat No.SH30809.01, United States) supplemented with 10% fetal bovine serum (HyClone, Cat No.SV30160.03, United States) and 1% penicillin/streptomycin (Hyclone, Cat No.SV30010, United States) in CO_2_ (5%) incubator at 37°C.

#### Cell Viability Assay

Cell viability was evaluated by using a CCK-8 assay kit (meilunbio, Cat No. MA0218, China) according to the manufacturer’s instructions. MDA-MB-231, BT549 and HCC1806 cells were seeded into 96-well plates with 3 × 10^3^ cells per well. After 72 h exposure in the medium containing desired compounds (20 *μ*M), 100 *μ*L fresh medium including 10 *μ*L CCK-8 reagent was added, and then the plates were incubated at 37°C for 2 h. The absorbances of CCK-8 in each well was measured at 450 and 600 nm by using a Cytation5 (BioTek, United States). DMSO was used as a control. Each sample was plated in triplicate.

#### Wound Healing Assays

Confluent MDA-MB-231, BT549, and HCC1806 cells were wounded by scratching using Wound Making Tool-Auto Scratch (BioTek, United States). After exposure for 24 h in the medium containing desired compounds (20 *μ*M), the scratched gap area of each cell monolayer was photographed by using Cytation5 (BioTek, United States) and quantified by using Image-Pro Plus 6.0. (http://rsb.info.nih.gov/ij/download.html). The migration efficiency of each cell was then determined as average percentage of closure of the scratch area. Each sample was plated in triplicate. All product recommended protocols were followed.

#### Western Blot Analysis

The total proteins of the whole cell lysates were separated by using SDS-PAGE and transferred to a PVDF membrane (Millipore, Cat No. IPVH00010, United States). After being probed with primary antibodies overnight at 4°C, the proteins of interest were then detected using HRP-conjugated IgG (CST, Cat No.7074P2, United States) and visualized by using ECL substrate (4ABiotech, Cat No.4AW011, China) based imaging with a Minichemi™ chemiluminescence imaging system (SAGECREATION, China).

#### Statistical Analysis

Analysis of statistical data, obtained from triplicate measurements, was performed by using the Student’s *t*-test for two groups or by one-way ANOVA for multiple groups. #*p* < 0.05 was considered to be significant.

### Biological Activity Assay on L6 Myotubes Cells

#### Cell Culture

L6 myotubes cells were maintained in *α*-MEM culture medium supplemented with 10% (v/v) fetal bovine serum (FBS), 100 U/mL penicillin-streptomycin, and incubated at 37°C in an atmosphere of 5% CO_2_.

#### Western Blotting Analysis

Cells were washed with pre-cold PBS, lysed using RIPA buffer supplemented with Proteinase Inhibitors Cocktail (MCE), and PMSF (MCE). AMPK, p-AMPK, AKT, p-AKT and GAPDH antibody were incubated overnight and the secondary antibody was incubated for 1 h at room temperature. Signals were detected using a Western blotting Imaging System according to the manufacturer’s specifications. The following primary antibodies were used for blotting: p-AKT (4060S, CST), AKT (9272S, CST), p-AMPK (2535S, CST), AMPK (2532, CST) and GAPDH (5174S, CST).

## Results and Discussion

**FIGURE 1:**
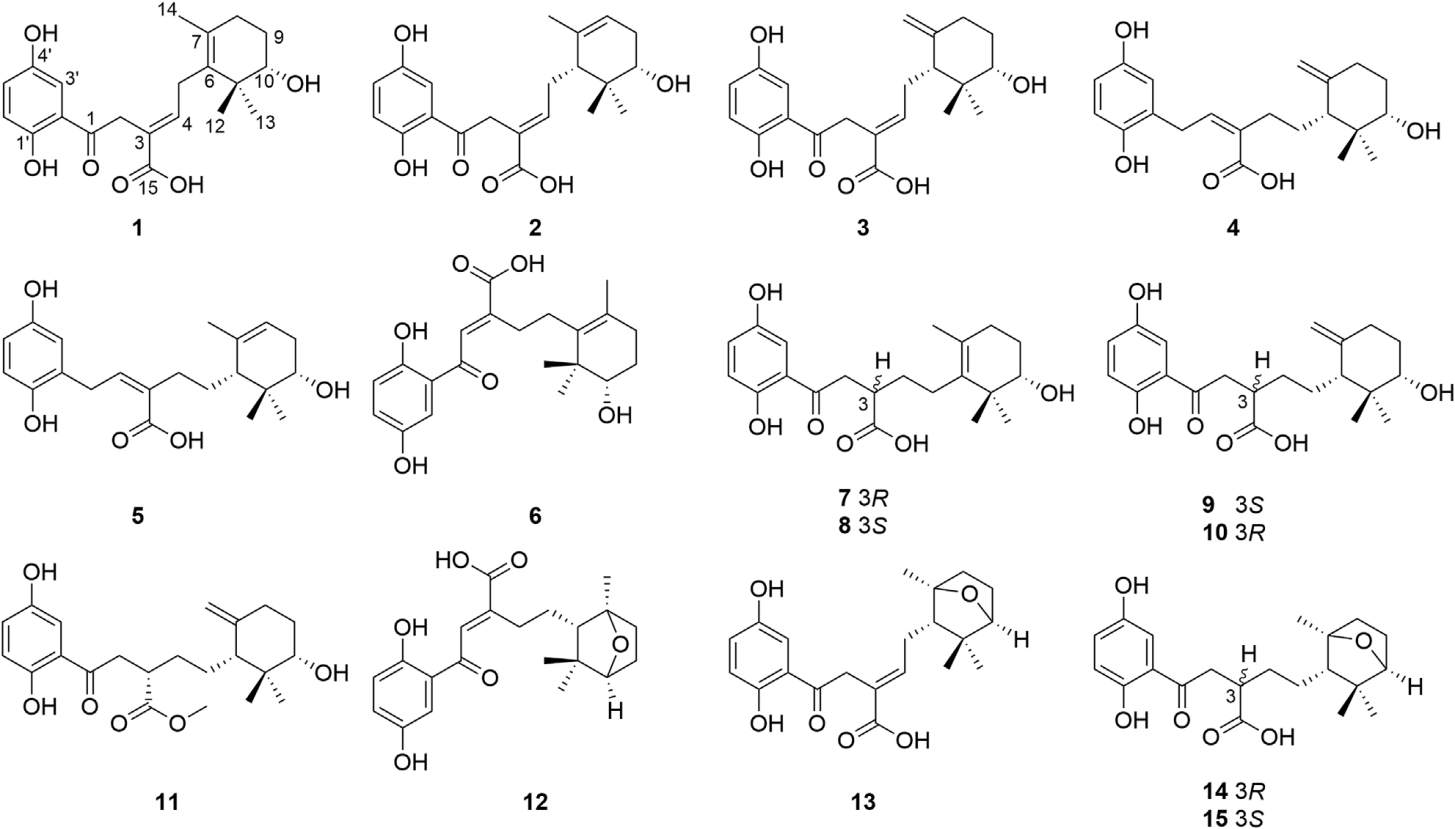
Structures of **1**–**15** isolated from *G. cochlear.*

Powders of the dried fruiting bodies of the fungus *G. cochlear* (200 kg) were first extracted with refluxing 80% EtOH and partitioned in water to obtain an ethyl acetate soluble fractions, an qliquote was then subjected to multiple chromatographic separation steps. This procedure led to isolation of fifteen the cyclohexane-type meroterpenoids **1**–**15** (Figure 1).

**FIGURE 2:**
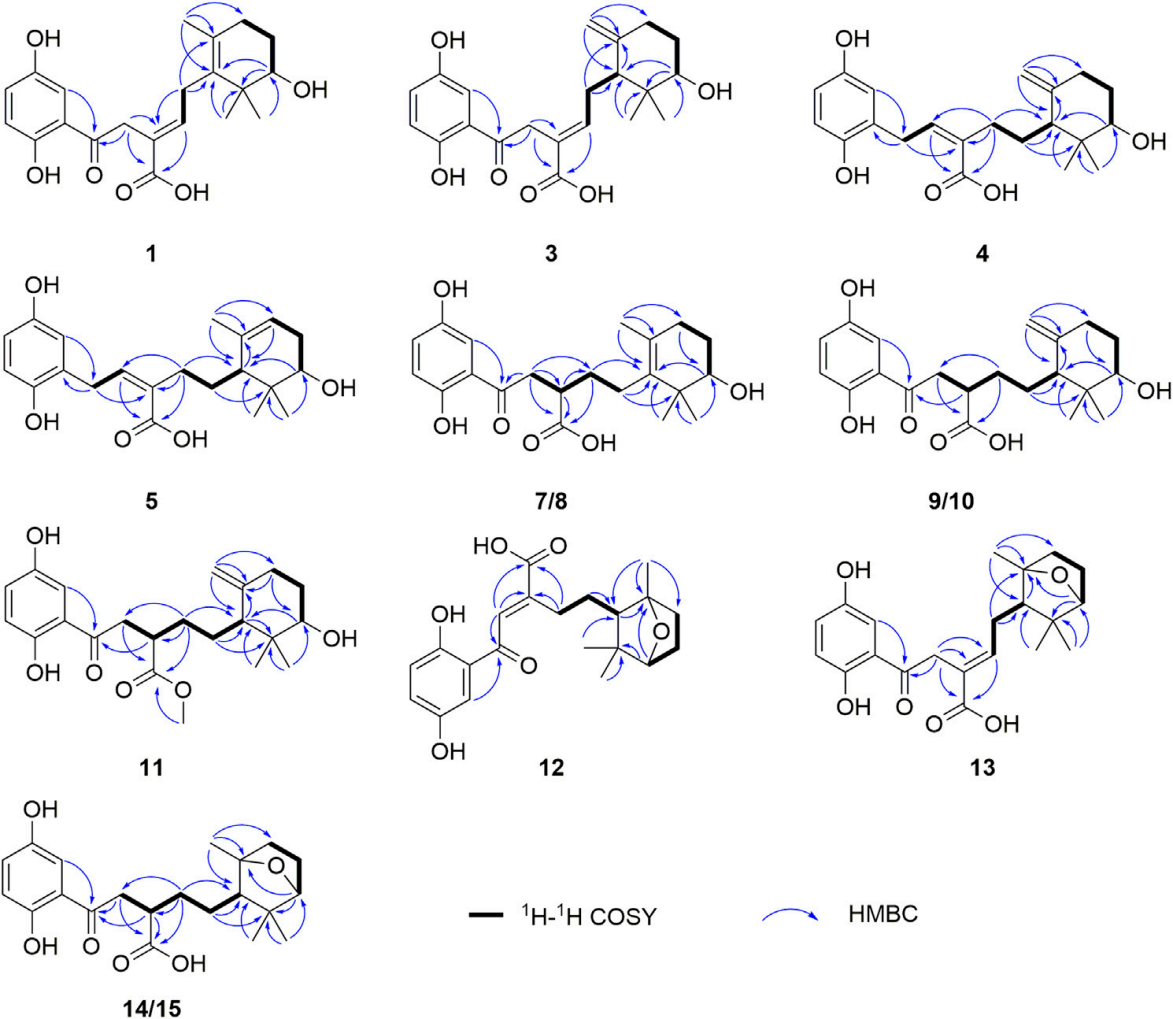
Key COSY and HMBC correlations of **1**, **3**–**5**, **7**–**15**.

**FIGURE 3:**
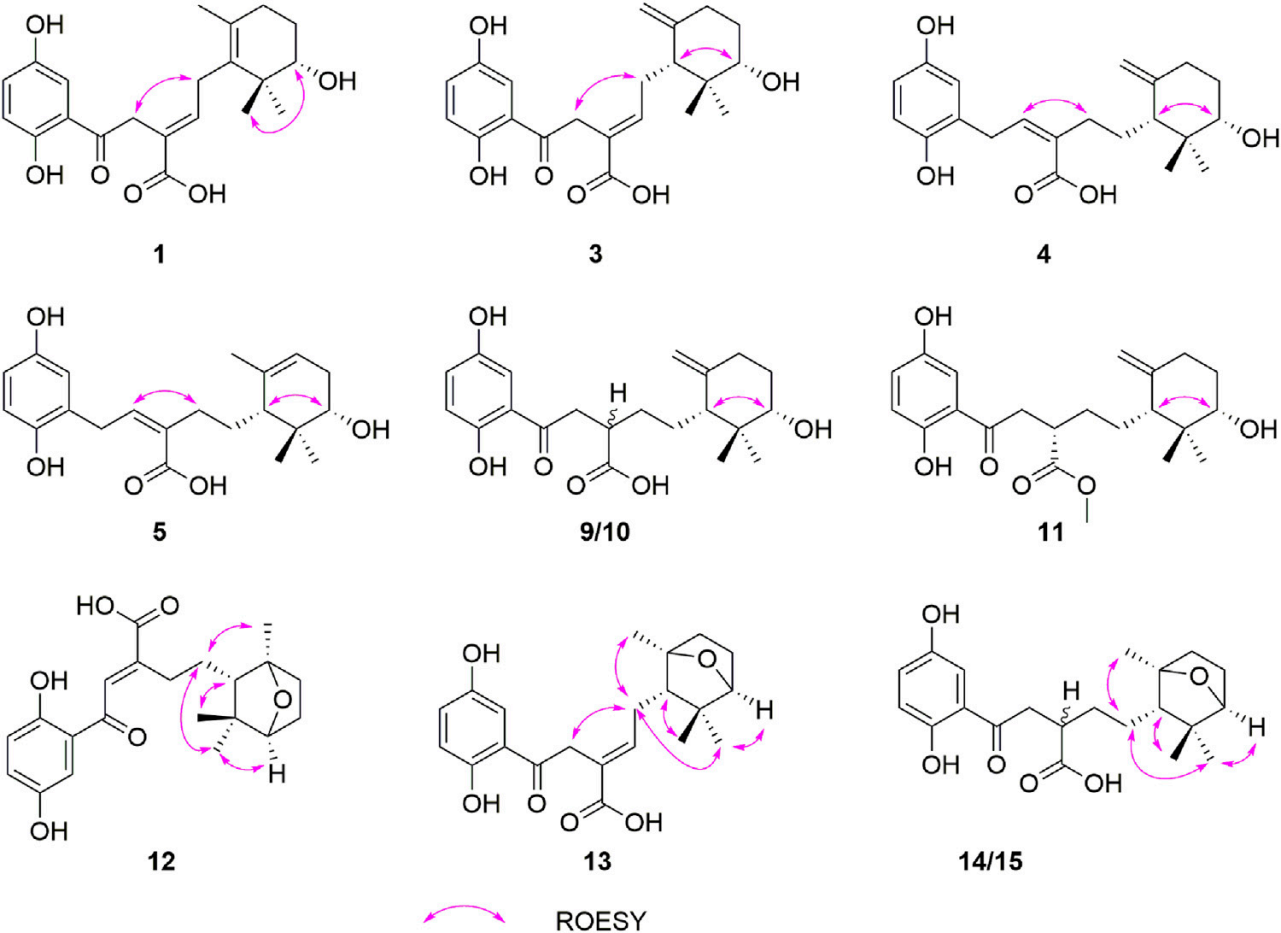
Key ROESY correlations of **1**, **3**–**5**, **9**–**15**.

**FIGURE 4:**
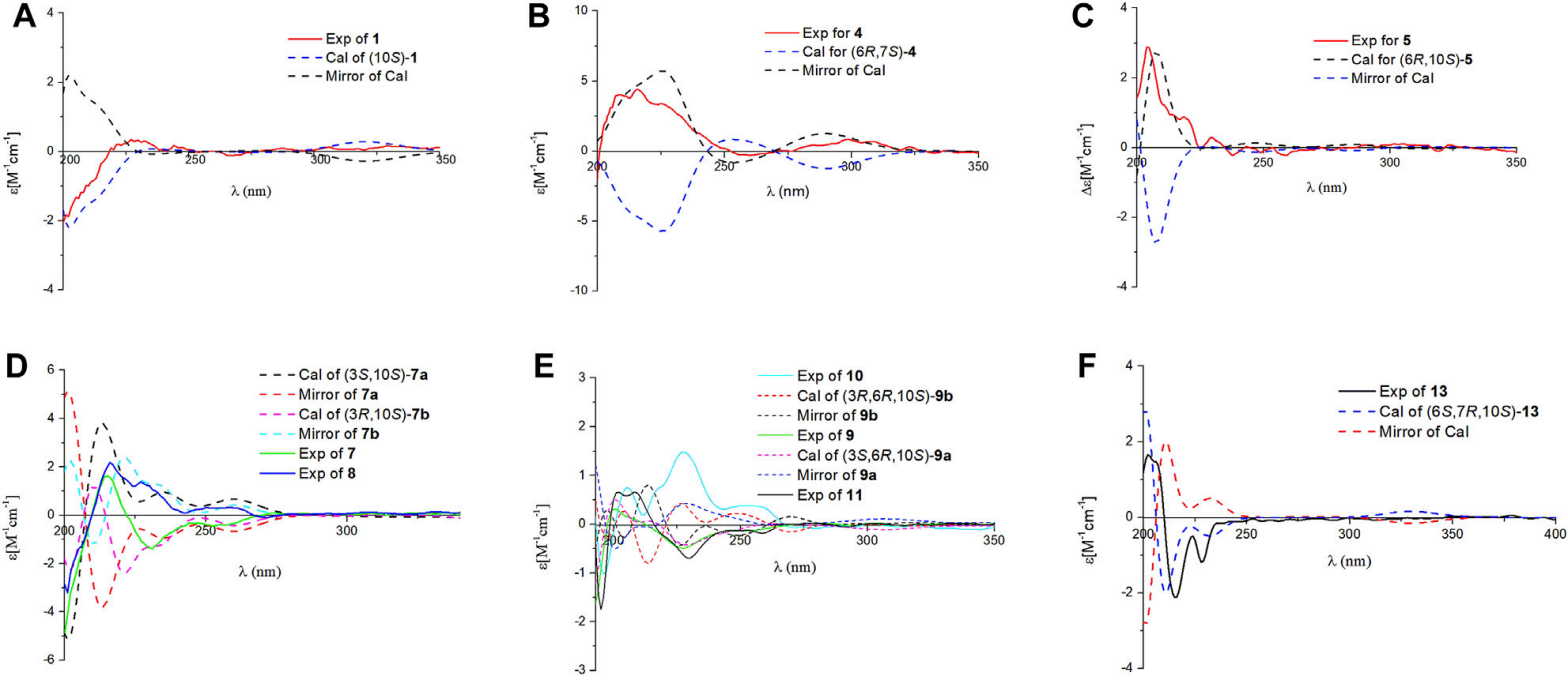
Comparison of the calculated ECD and experimental CD spectra in MeOH. **(A)**: The calculated ECD spectrum of (10*S*)-**1** at CAM-B3LYP/def2SVP level, σ = 0.20 eV; shift = –13 nm. **(B)**: The calculated ECD spectrum of (6*R*,10*S*)-**4** at CAM-B3LYP/def2SVP level, σ = 0.28 eV; shift = +8 nm. **(C)**: The calculated ECD spectrum of (6*R*,10*S*)-**5** at B3LYP/6-31G (d,p) level, σ = 0.25 eV; shift = +20 nm. **(D)**: The calculated ECD spectrum of (3*S*,10*S*)-**7a** at B3LYP/6-31G (d,p) level, σ = 0.25 eV; shift = +20 nm and calculated ECD spectrum of (3*R*,10*S*)-**7b** at B3LYP/6-31G (d,p) level, σ = 0.23 eV; shift = +20 nm. **(E)**: The calculated ECD spectrum of (3*S*,6*R*,10*S*)-**9a** at CAM-B3LYP/def2SVP level, σ = 0.28 eV; shift = +13 nm and calculated ECD spectrum of (3*R*,6*R*,10*S*)-**9b** at B3LYP/6-31G (d,p) level, σ = 0.20 eV; shift = +16 nm. **(F)**: The calculated ECD spectrum of (6*S*,6*R*,10*S*)-**13** at CAM-B3LYP/def2SVP level, σ = 0.20 eV; shift = –2 nm.

Ganodercin G (**1**), obtained as a yellow gum, has a molecular formula of C_21_H_26_O_6_ (nine degrees of unsaturation) established by using HRESIMS (*m/z* 375.1812 [M + H]^+^, calcd for 375.1802), and ^13^C NMR and DEPT spectroscopy. The presence of a 1,2,4-trisubstituted benzene ring in **1** was assigned by analysis of the ^1^H NMR spectrum of **1** (Table 1), which contain resonances in the aromatic region (*δ*_H_ 7.37, d, *J* = 2.2 Hz, H-3′; *δ*_H_ 7.02, dd, *J* = 8.9, 2.2 Hz, H-5′; *δ*_H_ 6.81, d, *J* = 8.9 Hz, H-6′). The ^13^C NMR and DEPT data of **1** (Table 2) revealed the presence of three methyls, four sp^3^ methylenes, five methines (four sp^2^ and one sp^3^) and nine nonprotonated carbons (one ketone, one carbonyl, six aromatic including two oxygenated and one aliphatic). The NMR spectra of **1** are similar to those of (–)-ganotheaecoloid F (Luo et al., 2018), which was isolated from the fruiting bodies of *G. theaecolum*, with the main difference being the presence of a *Δ* ^3(4)^ double bond in **1** instead of the *Δ* ^2(3)^ double bond in (–)-ganotheaecoloid F. This difference is supported by the ^1^H–^1^H COSY correlation of H-4 (*δ*_H_ 6.90)/H_2_-5 (*δ*_H_ 2.97 and 2.91) and the HMBC (Figure 2) correlations of H_2_-2 (*δ*_H_ 4.12)/C-3 (*δ*_C_ 126.6), C-15 (*δ*_C_ 170.5), H-4/C-15, and H_2_-5/C-3. The ROESY correlation (Figure 3) of H-2/H-5 suggested that the *Δ* ^3(4)^ double bond in **1** has *E* configuration. Chiral HPLC analysis revealed that compound **1** is enantiomerically pure. Time-dependent density functional theory (TDDFT) ECD calculations were used to determine the absolute configuration at the stereogenic center in **1**. It was found that the calculated ECD curve of (10*S*)-**1** obtained at the CAM-B3LYP/def2SVP level matches well with the experimental CD spectrum (Figure 4).

Cochlearol G (**2**) has been previously described as a component of fruiting bodies of *G. cochlear* by Wang and co-workers (Wang et al., 2019a). Although the ROESY experiment performed by Wang et al. led to assignment of the relative configurations of the two stereogenic centers in the terminal six-member ring of **2**, the configuration of the *Δ* ^3(4)^ double bond remained unresolved. As a result, we carefully analyzed the ROESY spectrum and found the existence of a correlation H_2_-2 (*δ*_H_ 4.16)/H_2_-5 (*δ*_H_ 2.65 and 2.42). This finding demonstrated that the *Δ* ^3(4)^ double bond in **2** has *E* configuration, rather than the *Z* configuration depicted by Wang et al. (Wang et al., 2019a).

Ganodercin H (**3**) was isolated as a yellow gum. The HRESIMS of **3** gave a molecular ion m/z 375.1804 [M + H]^+^ consistent with the molecular formula C_21_H_26_O_6_, indicating nine degrees of unsaturation. Detailed analysis of the 1D and 2D NMR data of **3** and ganotheaecoloid D shows that the *E Δ* ^3(4)^-double bond is present in **3** rather than *Z*-configuration found in ganotheaecoloid D (**8**). This conclusion is supported by the ROESY correlation in **3** of H-2 (*δ*_H_ 4.09)/H-5 (*δ*_H_ 2.45). The relative configurations of their stereogenic centers in the terminal cyclohexane ring in **3** were assigned as 6R*,10S* by using ROESY correlation of H-6 (*δ*_H_ 1.94)/H-10 (*δ*_H_ 3.38). Moreover, the absolute configurations at these centers were assigned as 6*R*,10*S* by comparing its CD spectrum to that of ganotheaecoloid D.

Ganodercin I (**4**), isolated as a yellow gum, has the molecular formula C_21_H_28_O_5_ (eight degrees of unsaturation) based on HRESIMS analysis (m/z 361.2012 [M + H]^+^; calcd for C_21_H_29_O_5_^+^, 361.2010). 1D and 2D NMR analysis shows that **4** has a structure that is similar to that of **3**. The presence of resonances associated with a terminal double bond (*δ*_H_ 4.84, Ha-14, *δ*_H_ 4.61, Hb-14) and two singlet methyls (*δ*_H_ 0.97, H_3_-12, *δ*_H_ 0.64, H_3_-13) in ^1^H NMR spectrum reveal that **4** contains the same terminal cyclohexane part that is present in **3**. The differences between **3** and **4** are that a methylene appears at C-1 in the latter instead of a ketone in the former and the double bond *Δ* ^3(4)^ of **3** is at *Δ* ^2(3)^ in **4**. The ^1^H-^1^H COSY correlation of H_2_-1 (*δ*_H_ 3.73 and 3.54)/H-2 (*δ*_H_ 5.89) and the HMBC correlations of H-3′ (*δ*_H_ 6.58)/C-1 (*δ*_C_ 31.7), H_2_-1/C-3 (*δ*_C_ 133.6), H-2/C-15 (*δ*_C_ 172.3) and H_2_-4/C-2 (*δ*_C_ 140.5), C-3, C-15 support the above conclusions. The relative configurations of the two stereogenic centers in **4** were assigned as 6R*,10S* based on ROESY correlation of H-6 (*δ*_H_ 1.66)/H-10 (*δ*_H_ 3.29)*.* Further examination of ROESY correlation of H-2/H-4 indicate that the *Δ* ^2(3)^ double bond is *Z-*configuration. Finally, the experimental CD spectrum matched the calculated (CAM-B3LYP/def2SVP level) spectrum of (6*R*,10*S*)-**4**.

Ganodercin J **(5)** has the same molecular formula as **4** (HRESIMS). Comariasons of ^1^H, ^13^C NMR, and DEPT spectra of **5** and **4** suggest they have similar structures. The difference between **5** and **4**, inferred from 1D NMR data, is that the double bond on the terminal ring in **5** is at the *Δ* ^7(8)^, whereas in compound **4** it is at *Δ* ^7(14)^. This conclusion is supported by the observation of HMBC correlations of H_3_-14 (*δ*_H_ 1.70)/C-6 (*δ*_C_ 50.2), C-7 (*δ*_C_ 137.8), C-8 (*δ*_C_ 120.1) and H-8 (*δ*_H_ 5.22)/C-6. The configuration of the *Δ* ^2(3)^ double bond was determined as *Z* by observing a ROESY correlation of H-2/H_2_-4. Moreover, the observation of ROESY correlation of H-6 (*δ*_H_ 1.66)/H-10 (*δ*_H_ 3.36) indicated that **5** is the 6R*,10S* diastereomer. The absolute configurations at the stereogenic centers in **5** were assigned as 6*R* 10*S* by comparing experimental CD spectrum with calculated ECD curve (Figure 4).

Two isolated meroterpenoids **7 (**ganodercin K, yellow gum**)** and **8** (3-*epi*-ganodercin K, yellow gum) were found to have the same molecular formula (C_21_H_28_O_6_) and nearly identical NMR data, suggesting that they have the same planar structure with both lacking unsaturation in chains connecting the aryl and cyclohexane moieties. Indeed, a detailed comparison of their 1D NMR data with those of **1** shows that **7** and **8** lack the *Δ* ^3(4)^ double bond in **1**, a conclusion supported by ^1^H–^1^H COSY correlations of H-2/H-3/H-4/H-5, and the HMBC correlations of H-2/C-1, C-3, C-4, C-15 and H-3/C-4, C-15. It was likely that **7** and **8** are diastereomers having 3S*,10S* and 3R*,10S* relative configurations at their stereogenic centers. Analysis of the CD spectra of **7** and **8** enabled the use of computational methods to elucidate the absolute configurations at their stereogenic centers. As seen by viewing Figure 4, the experimental CD spectrum of **7** well matches the calculated ECD curve of 3*R*,10*S.* In addition, the calculated ECD spectrum of 3*S*,10*S* matches the experimental CD spectrum of **8**.

Compounds **9** and **10** were previously isolated from *G. theaecolum* by Luo et al. (Luo et al., 2018) and characterized the enantiomers of ganotheaecoloid C. However, the absolute configurations at the three stereogenic centers in these substances were not determined in the earlier effort. As a result, we used TDDFT-ECD calculations to determine their absolute configurations. It can be seen from viewing Figure 4, that the experimental CD spectrum of **9** agrees well with the calculated ECD curve of the 3*S*,6*R*,10*S* stereoisomer. Importantly, the experimental CD spectrum of **10** is in close agreement with the calculated ECD spectrum of the 3*R*,6*R*,10*S* stereoisomer, showing that **9** and **10** are actually epimers rather than enantiomers. By carefully analyzing the 1D NMR data reported by Luo, it was found that pairs of signals are present in the ^13^C NMR spectrum, supporting our conclusion. The revised structures of **9** and **10** were renamed as ganodercin L for **9** and 3-*epi*-ganodercin L for **10**.

Ganodercin M (**11**) was obtained as a yellow gum and has the molecular formula C_22_H_30_O_6_ (HRESIMS ion observed at m/z 391.2120 [M + H]^+^, calcd for 391.2115). The NMR data of **11** are similar to those of **9**, except that the free C-15 carboxylic acid group in **9** is a methyl ester in **11**. This conclusion is confirmed by observations of the HMBC correlation of OCH_3_/C-15. The ROESY correlation of H-6/H-10 showed that the relative configurations at the stereogenic centers in the terminal ring in **11** are 6R*,10S*. The computational methods applied to **9** and **10** were used to determine the absolute configurations at the stereogenic centers in **11**. Matching experimental and calculated CD spectra showed that **11** is the 3*S*,6*R*,10*S* stereoisomer.

The NMR spectroscopic data of ganodercin N (**12**) (yellow powder, C_21_H_26_O_6_) are similar to those of (–)-ganotheaecoloid F (Luo et al., 2018). One difference is that a methine (*δ*_C_ 57.1) and a nonprotonated carbon (*δ*_C_ 88.6) are present in **12** instead of the double bond (*δ*_C_ 136.7 and *δ*_C_ 128.6) in (–)-ganotheaecoloid F. This proposal is supported by the HMBC correlations of H_3_-14 (*δ*_H_ 1.28)/C-6 (*δ*_C_ 57.1), C-7 (*δ*_C_ 88.6), C-8 (*δ*_C_ 39.7). Also, the downfield chemical shift of C-10 at 87.6 ppm, indicated that C-10 is the oxygenated. Moreover, the HMBC correlation of H-10/C-7 indicated that C-10 is linked to C-7 via an oxygen bridge. The relative configurations at the stereogenic centers in the terminal bicyclic ring in **12** were determined by using ROESY data. ROESY correlation of H-6 (*δ*_H_ 1.26)/H_3_-12 (*δ*_H_ 1.02) suggested that both H-6 and CH_3_-12 have a *β*-orientation. Accordingly, the ROESY correlation of H-10 (*δ*_H_ 3.70)/H_3_-13 (*δ*_H_ 0.97) revealed that H-10 and CH_3_-13 have a *α-*orientation. The C-14 methyl was determined to have a *α-*orientation, when consideration is given to the existence of the oxygen bridge. This proposal was further supported by the ROESY correlation of H_2_-4 (*δ*_H_ 2.58 and 2.52)/H_3_-14. Thus, the relative configurations at the stereogenic centers in **12** were assigned as 6S*,7R*,10S*. Since no strong correlation of H-2/H-4 was observed, we hypothesized that the *Δ* ^2(3)^ double bond in **12** is *E*. Density functional theory (DFT, B3LYP/6-311G(d,p) level) was used to verify this proposal. The experimental NMR data of **12** were compared with those calculated for **12a** (*E*-isomer) and **12b** (*Z*-isomer). It was found that the calculated ^13^C NMR data for **12a** possesses the highest *R* ^2^ value and a 100% probability in DP4+ analysis. Because no observable Cotton effects are present in the experimental CD spectrum of **12**, it was not possible to use ECD calculations to assign stereochemistry. However, the absolute configuration at the stereogenic centers in **12** were assigned as 6*S*,7*R*,10*S* by comparing the calculated specific optical rotation +22.2 for (6*S*,7*R*,10*S*) with the experimental one of **12** ([α]_D_^20^+22.5).

Ganodercin O (**13**) has the same molecular formula as **12** and similar NMR data, differing only by resonances associated with the location of the double bond. The position of double bond in **13** was demonstrated to be *Δ* ^3(4)^ by the observation of ^1^H–^1^H COSY correlations of H-4/H-5/H-6, and the HMBC correlations of H-2/C-1, C-3, C-4, C-15 and H-4/C-15. The double bond *Δ* ^7(8)^ was deduced to have the *E-*configuration by existence of the ROESY correlation of H-2/H-4. Similar to that in **12**, H-6 and CH_3_-12 were determined to have a *β-*orientation by presence of the ROESY correlation of H-6/H_3_-12. Moreover, H-10 and CH_3_-14 were assigned to have a *α-*orientation by the ROESY correlations between H-10/H_3_-13, H_2_-5/H-13, H-14. As a result, the relative configurations of the stereogenic centers in **13** were determined to be 6S*,7R*,10S*. Finally, a match between the computed [B3LYP/6-31 g(d,p)] ECD spectrum of 6*S*,7*R*,10*S-* **13** and the experimental one provided the absolute configurations at the stereogenic centers in **13** (Figure 1).

**FIGURE 5:**
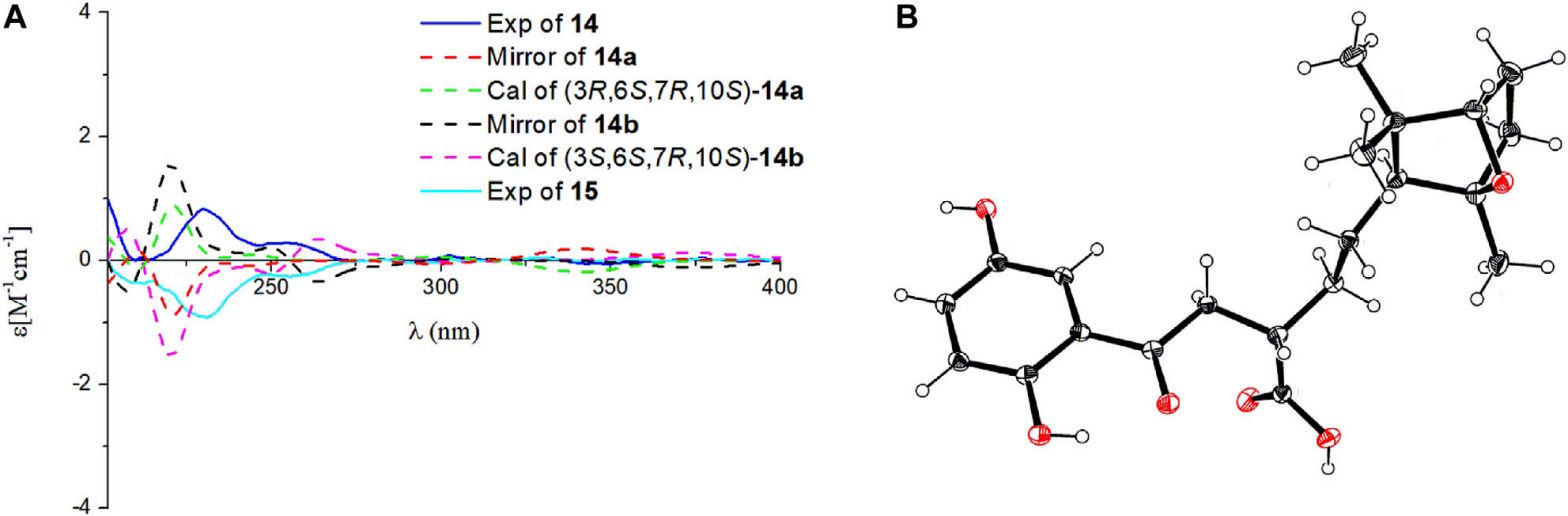
Comparison of the calculated ECD spectrum of (3*R*,6*S*,7*R*,10*S*)-**14a** at CAM-B3LYP/def2SVP level, σ = 0.20 eV, shift = +10 nm; and the calculated ECD spectrum of (3*S*,6*S*,7*R*,10*S*)-**14b** at B3LYP/6-31G (d,p) level, σ = 0.20 eV, shift = +10 nm; with the experimental CD spectra of **14** and **15** in MeOH **(A)**. Ortep plot of the X-ray crystallographic data of **15**. Anisotropic displacement ellipsoids display 50% probability levels **(B)**.

Ganodercin P (**14**) and 3-*epi*-ganodercin P (**15**) were found to have the same molecular formula of C_21_H_28_O_6_ by using HRESIMS. Careful analysis revealed that their NMR data were similar, suggesting that they have the same planar structures. The observations of ^1^H–^1^H COSY correlations of H-2/H-3/H-4, and the HMBC correlations of H-2/C-1, C-15 and H-3/C-15 clearly revealed that one methylene (C-2) and one methine (C-3) in both **14** and **15** replace the *E*-double bond in **12**. Because **14** and **15** contain the same terminal bicyclic ring that is present in **12** and **13**, the relative configurations at the stereogenic centers were determined to be 6S*,7R*,10S* by the presence of ROESY correlations H-6/H_3_-12, H-10/H_3_-13, H_2_-5/H_3_-13, H_3_-14. The relatively configuration of C-3 is difficult to determined owing to the flexible nature of the chain. As a result, both **14** and **15** can be the two possible diastereomers 3R*,6S*,7R*,10S* and 3S*,6S*,7R*,10S*. Computational methods were used to determine the absolute configurations at the stereogenic centers in **14** and **15**. The calculated curves of these substances showed that the terminal oxygen bridge doe not contribute to the Cotton effects (Figure 5). As a result, the absolute configuration of C-3 in **14** is *R* and that of compound **15** is *S*.

X-ray crustallographic analysis of **15** was carried out, which was generated by crystallization from Kappa single diffractometer in CuK*α* with a Flack parameter of –0.08 (7). Analysis of the crystal data leads to assignment of the absolute configurations at the stereogenic centers in **15** to be 3*S*,6*S*,7*R*,10*S*. Since **14** and **15** are C-3 epimers, the absolute configurations at the centers in **14** are 3*R*,6*S*,7*R*,10*S.*

The final substance isolated was (−)-ganotheaecoloid F (**6**), which was identified by comparison its spectroscopic data with those reported in the literature (Luo et al., 2018).

In this study, we have determined the structures and absolute configurations at the stereogenic centers in 15 meroterpenoids isolated from dried fruiting bodies of *G. cochlear.* It is worthy noting that all of these substances contain a terminal cyclohexane ring and all are enantiomerically pure. Meroterpenoids of this type isolated from *Ganoderma* have been also found to exist in enantiomerically pure (Luo et al., 2018; Wang et al., 2019a; Wang et al., 2019b). Among them, the absolute configuration of four structures was successfully determined. The absolute configurations at the centers in these substances as well as the fact that C-10 in all have the *S*-configuration might be related to their biosynthetic origin, and might aid subsequent structural identification of analogues.

**FIGURE 6:**
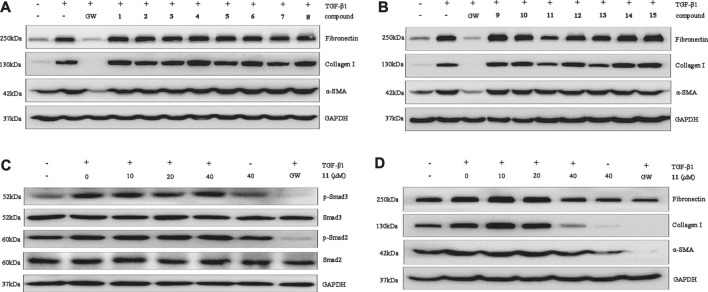
Compounds **1**–**15** inhibit renal fibrosis *in vitro*. **(A,B)**: Protein expression of collagen I, fibronectin, and *α*-SMA was examined by Western blot analysis. **(C)**: Compound **11** at 0 *μ*M, 10 *μ*M, 20 *μ*M, and 40 *μ*M affected TGF-*β*1-induced Smad phosphorylation. GAPDH was used as an internal control. Three independent experiments were performed. **(D)**: Compound **11** dose-dependently inhibits fibronectin and collagen I expression. GAPDH was used as an internal control. GW was used as a positive control. GW: GW788388. Three independent experiments were performed.

The protective activity of the meroterpenoids isolated in this effort were assessed by observing the expression of renal fibroblast biomarkers in TGF-*β*1–induced NRK-52E, which plays an important role in stimulation of renal fibroblasts (Zhang et al., 2012; Meng et al., 2015). The results showed that **11** displays selective inhibitory activity in that it significantly inhibits over-expression of fibronectin, collagen I and *α*-SMA at the protein level at a concentration of 40 *μ*M (Figures 6A,B). In addition, western blot analysis, carried out in a dose-concentration dependent manner, demonstrated that the optimum activity of **11** is 40 *μ*M (Figure 6D). To explore the mechanism underlying the antifibrotic effect of, Smad2/3 phosphorylation was investigated. We found that **11** has no effect on phosphorylation of Smad2 or Smad3 in TGF-*β*1-induced NRK-52E cells at concentrations of 10 *μ*M, 20 and 40 *μ*M (Figure 6C), suggesting that its effects could play potential roles in renal fibrosis through a non-Smad pathway.

**FIGURE 7:**
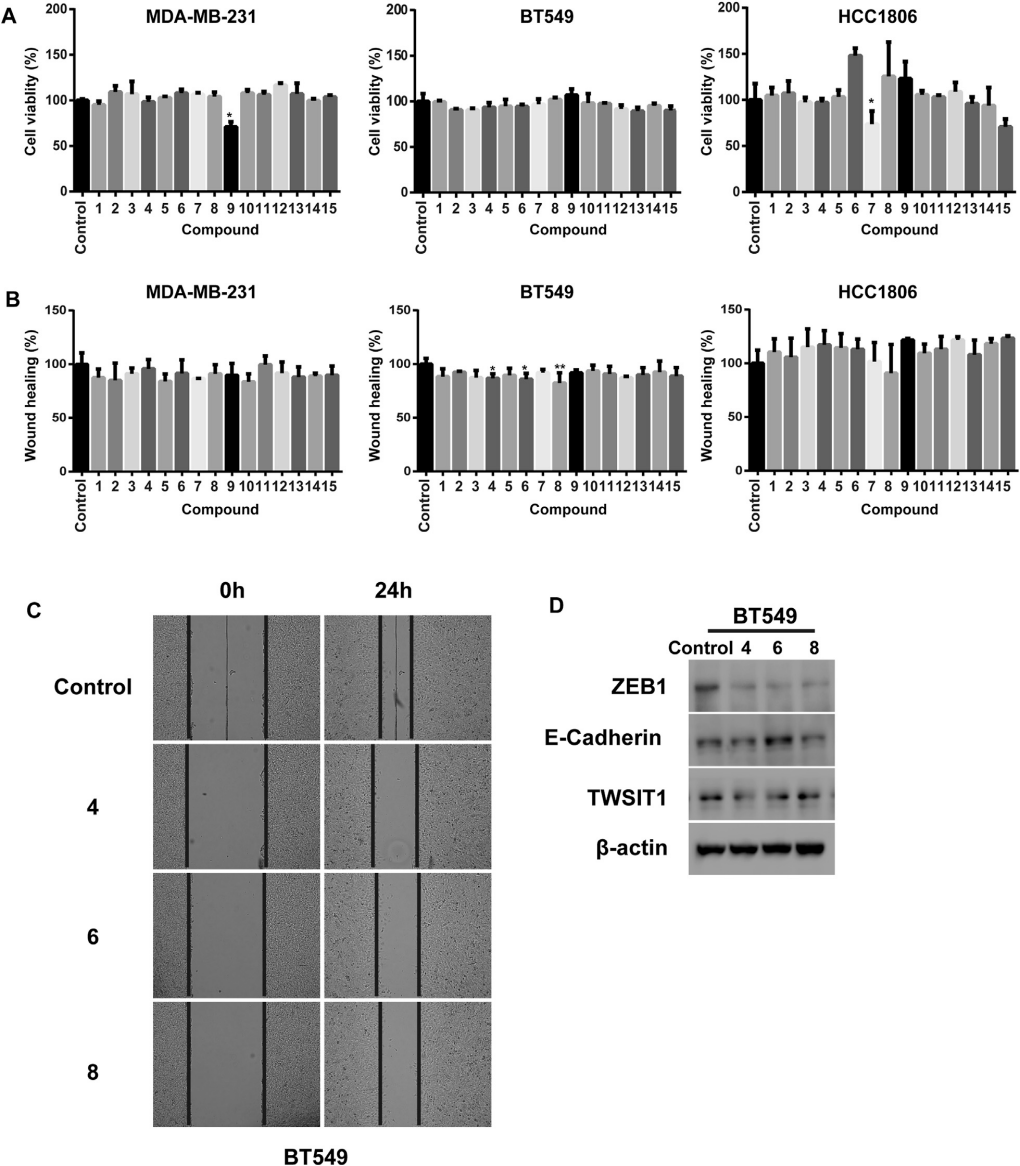
**(A)** Cell viabilities of MDA-MB-231, BT549 and HCC1806 were analyzed by using CCK8. All compounds concentrations were 20 *μ*M; **(B)** Quantification of the migratory ability of MDA-MB-231, BT549 and HCC1806 cells. All compounds concentrations were 20 *μ*M; **(C)** Representative images of the wound healing assay in BT549 cells; **(D)** Compounds **4**, **6** and **8** (40 *μ*M) treated BT549 celllysates were subjected to immunobloting with the indicated antibodies respectively. The results were represented as mean ± S.D. of biological triplicates. #*p* < 0.05.

We investigated the cellular phenotype promoted by the isolated substances in breast cancer cells by using a cell viability and a wound healing assay in three TNBC cell lines including MDA-MB-231, BT549 and HCC1806. Inspection of the plots given in Figure 7A shows that treatment with **7** and **9** results in significant decreases in cell viability of 29.2 and 26.3% in MDA-MB-231 and HCC1806 cells, respectively. The other meroterpenoids have negligible inhibitory effects on cell viability even at concentrations as high as 20 *μ*M (Figure 7A). Interestingly, although all the fifteen substances have rather low cytotoxicities toward these three breast cancer cell lines, three of the isolates including **4**, **6** and **8** significant inhibit the migration ability of BT549 cells. All of the other meroterpenoids display no significant effects on MDA-MB-231 and HCC1806 cells (Figures 7B,C). Moreover, we observed that treatment with **4** and **8** markedly decreases the protein level of TWIST1 and ZEB1 without noticeably affecting the E-cadherin level in BT549 cell lysates. Meroterpenoid **6** not only decreases the protein level of TWIST1 and ZEB1, it also increases the protein level of E-cadherin (Figure 7D). ZEB1, E-cadherin and TWIST1 are generally acknowledged to be transcriptional factors driving the Epithelial–Mesenchymal Transition (EMT), one of the most important pathogenic events occurring in the initiation of cancer metastasis. Thus, the data indicate that **4**, **6** and **8** suppress the metastatic potential of TNBC cells through down regulation of EMT, and consequently they are promising lead compounds for the development of the anti-cancer drugs against metastasis of TNBC.

**FIGURE 8:**
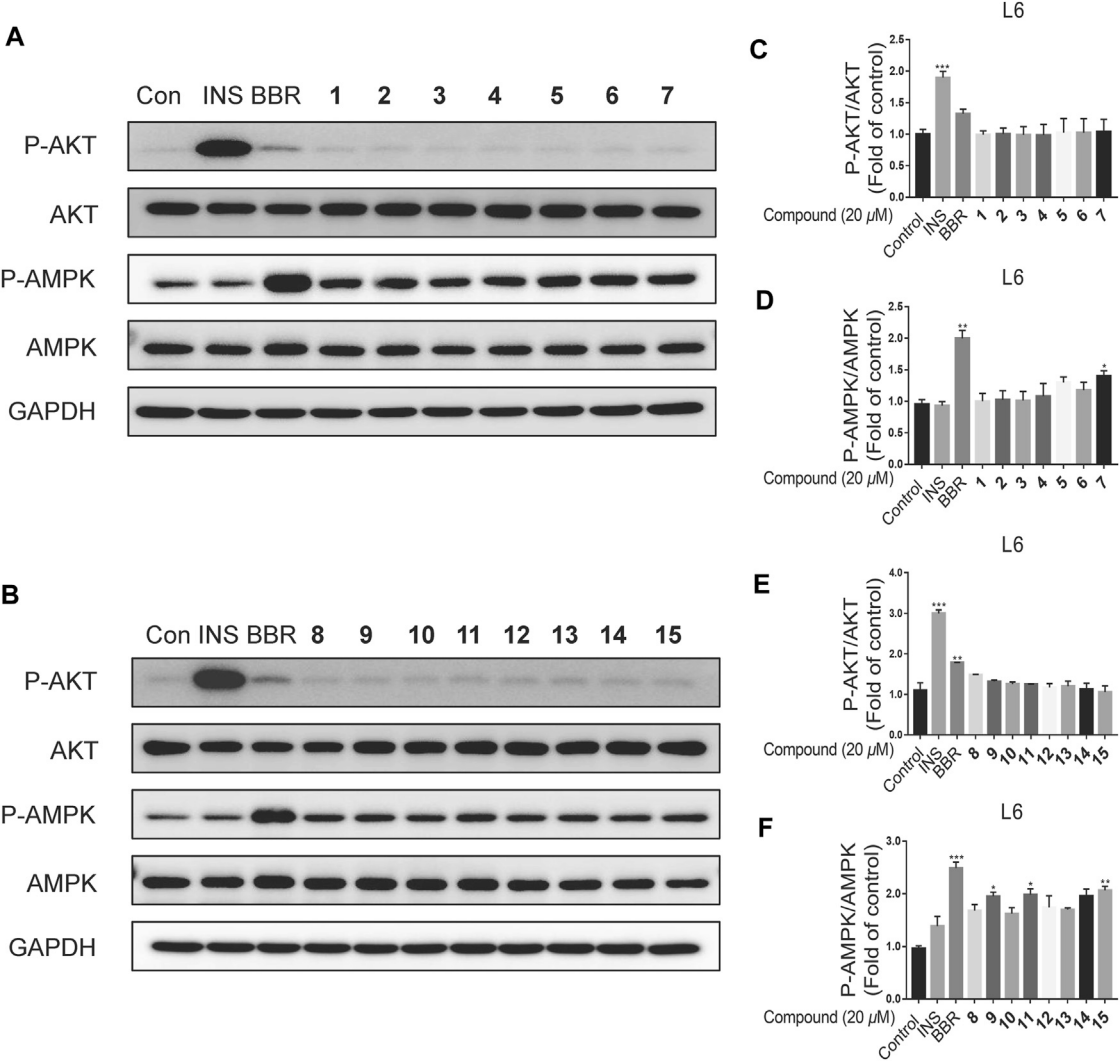
The effects of compounds on L6 myotubes. **(A–F)**: L6 myotubes were treated with vehicle or 20 *μ*M compounds or 10 *μ*M BBR for 24 h (*n* = 3). Data are for three replicate experiments. (*n* = 3) *p < 0.05, **p < 0.01 ***p < 0.001, one-way ANOVA.

All the isolated meroterpenoids at a concentration of 20 *μ*M were exposed to normal L6 myotubes cells for 24 h. The control group was treated with insulin (INS, 100 nM, 15 min) and Berberine (BBR, 10 *μ*M, 24 h). Notably, INS and BBR treatment led to significant increases in the phosphorylation AKT and AMPK, respectively. Compared with the control group, the meroterpenoids have no significant impact on the AKT pathway. Meanwhile, **7**, **9**, **11** and **15** significantly up-regulate p-AMPK protein expression (Figure 8). This finding suggests that these substances might enhance insulin sensitivity by activating AMP-activated protein kinase (AMPK) in normal L6 myotubes cells.

## Conclusion

In conclusion, the study described above resulted in the isolation of eleven new meroterpenoids, and four known meroterpenoids. The structure of cochlearol G (**2**) was revised, and the absolute configurations at the stereogenic centers in **9** and **10** were determined by using ECD calculations. Biological studies related to renal fibrosis showed that (1) **11** inhibits over-expression of fibronectin, collagen I and *α*-SMA, (2) **4**, **6** and **8** significantly inhibitof the migration ability of BT549 cells, (3) **6** decreases the protein level of TWIST1 and ZEB1 and increases the protein level of E-cadherin, and (4) **7**, **9**, **11** and **15** significantly up-regulate p-AMPK protein expression in normal L6 myotubes cells.

## Data Availability

The original contributions presented in the study are included in the article/Supplementary Material, further inquiries can be directed to the corresponding authors.
