# Supplementary material for: Terminal Cyclohexane-Type Meroterpenoids from the Fruiting Bodies of Ganoderma cochlear
Source: Front Chem. 2021 Dec 3;9:783705. doi: 10.3389/fchem.2021.783705 (PMC8677669; doi:10.3389/fchem.2021.783705)
Supplement: Supplementary file 2 [file DataSheet4.doc]

Supplementary Material

# Terminal cyclohexane-type meroterpenoids from the fruiting bodies of *Ganoderma cochlear*

Fu-Ying Qin^1^, Te Xu^1^, Yan-Peng Li^1^, Hao-Xing Zhang^1^, Dan Cai^1^, Li-Zhong Liu^1^*, Yong-Xian Cheng^1,2^*

^1^Institute for Inheritance-Based Innovation of Chinese Medicine, School of Pharmaceutical Sciences, School of Medicine, College of Life Sciences and Oceanography, Health Science Center, Shenzhen University, Shenzhen 518060, China.

^2^Guangdong Key Laboratory for Functional Substances in Medicinal Edible Resources and Healthcare Products, School of Life Sciences and Food Engineering, Hanshan Normal University, Chaozhou 521041, China

*** Correspondence:** Yong-Xian Cheng, Li-Zhong Liu

email: [yxcheng@szu.edu.cn](mailto:yxcheng@szu.edu.cn) (Y.X. Cheng); [liulz@szu.edu.cn](mailto:liulz@szu.edu.cn) (L.Z. Liu).

**Contents**

Figure S1– S6. 1D and 2D NMR spectra of **1** in methanol-*d*_4_.

Figure S7. HRESIMS of **1**.

Figure S8– S13. 1D and 2D NMR spectra of **2** in methanol-*d*_4_.

Figure S14. HRESIMS of **2**.

Figure S15– S20. 1D and 2D NMR spectra of **3** in methanol-*d*_4_.

Figure S21. CD spectrum of **3**.

Figure S22. HRESIMS of **3**.

Figure S23– S28. 1D and 2D NMR spectra of **4** in methanol-*d*_4_.

Figure S29. HRESIMS of **4**.

Figure S30– S35. 1D and 2D NMR spectra of **5** in methanol-*d*_4_.

Figure S36. HRESIMS of **5**.

Figure S37– S42. 1D and 2D NMR spectra of **7** in acetone-*d*_6_.

Figure S43. HRESIMS of **7**.

Figure S44– S49. 1D and 2D NMR spectra of **8** in acetone-*d*_6_.

Figure S50. HRESIMS of **8**.

Figure S51– S56. 1D and 2D NMR spectra of **9** in acetone-*d*_6_.

Figure S57. HRESIMS of **9**.

Figure S58– S63. 1D and 2D NMR spectra of **10** in acetone-*d*_6_.

Figure S64. HRESIMS of **10**.

Figure S65– S70. 1D and 2D NMR spectra of **11** in methanol-*d*_4_.

Figure S71. HRESIMS of **11**.

Figure S72– S77. 1D and 2D NMR spectra of **12** in methanol-*d*_4_.

Figure S78. HRESIMS of **12**.

Figure S79– S84. 1D and 2D NMR spectra of **13** in methanol-*d*_4_.

Figure S85. HRESIMS of **13**.

Figure S86– S91. 1D and 2D NMR spectra of **14** in methanol-*d*_4_.

Figure S92. HRESIMS of **14**.

Figure S93– S98. 1D and 2D NMR spectra of **15** in methanol-*d*_4_.

Figure S99. HRESIMS of **15**.

Crystal data of **15**.

ECD calculations

NMR calculations of **12**

Specific optical rotation data (ORD) calculations of **12**


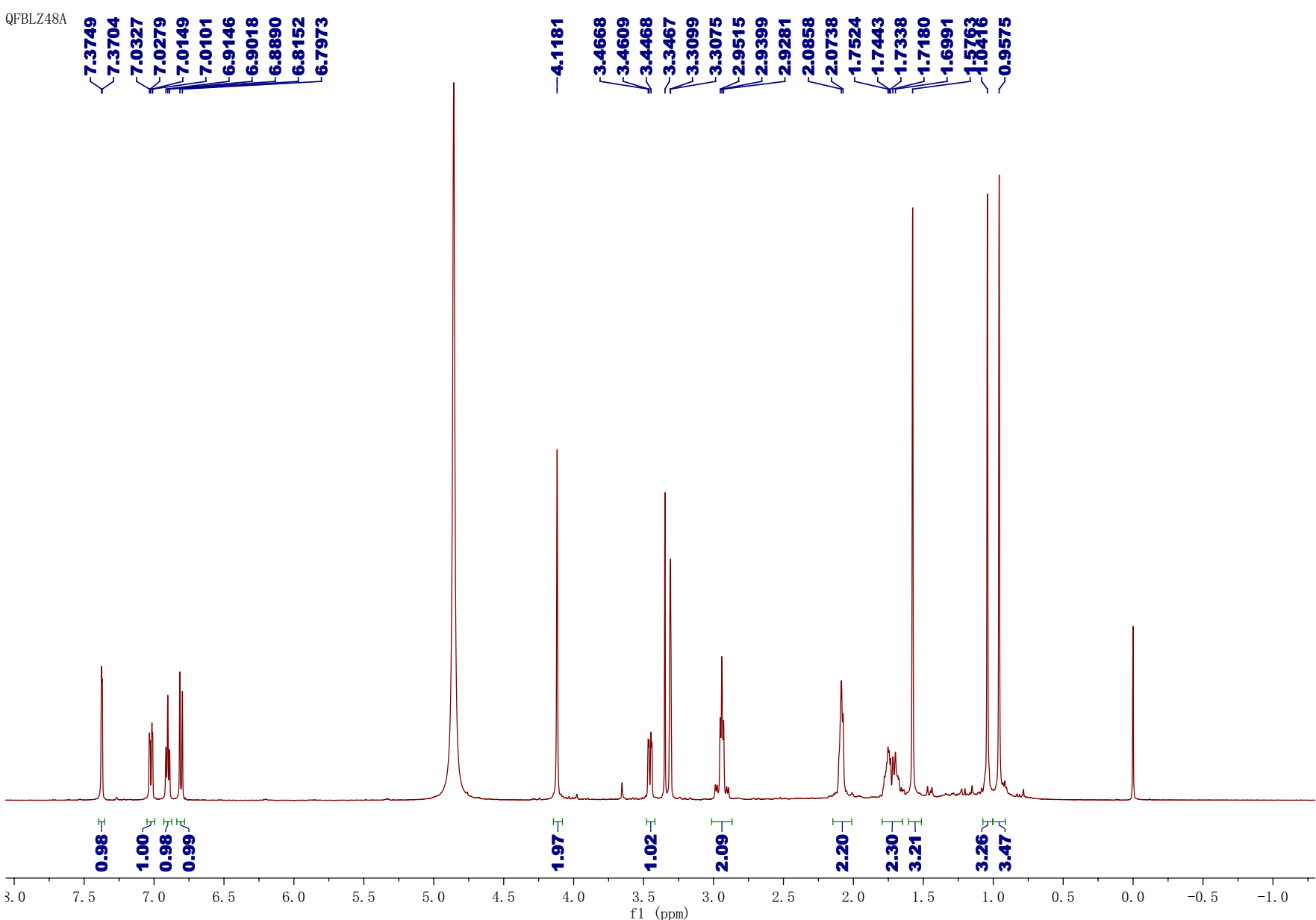


Figure S1. ^1^H NMR spectrum of **1** in methanol-*d*_4_.


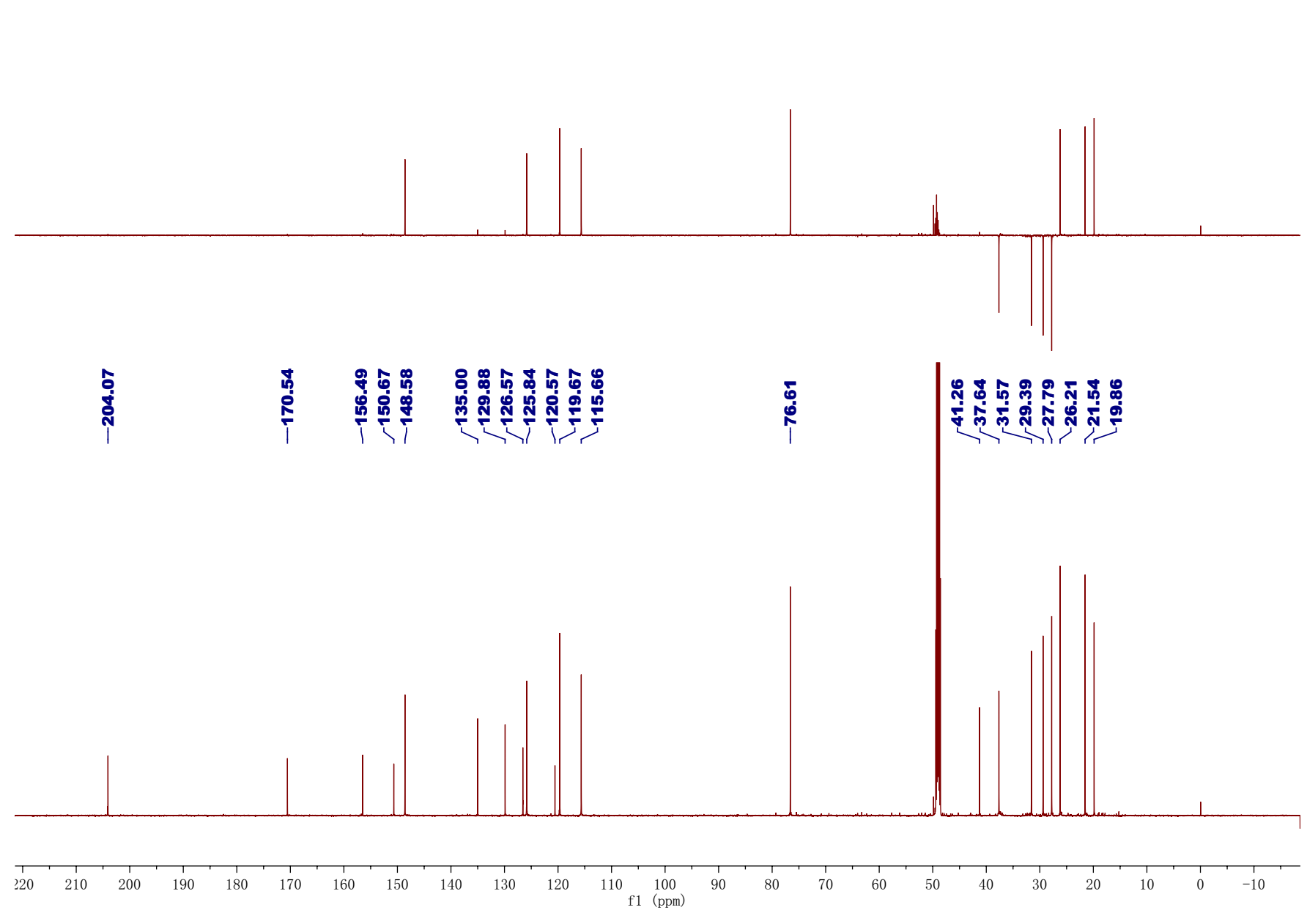


Figure S2. ^13^C NMR and DEPT spectra of **1** in methanol*-d*_4_.


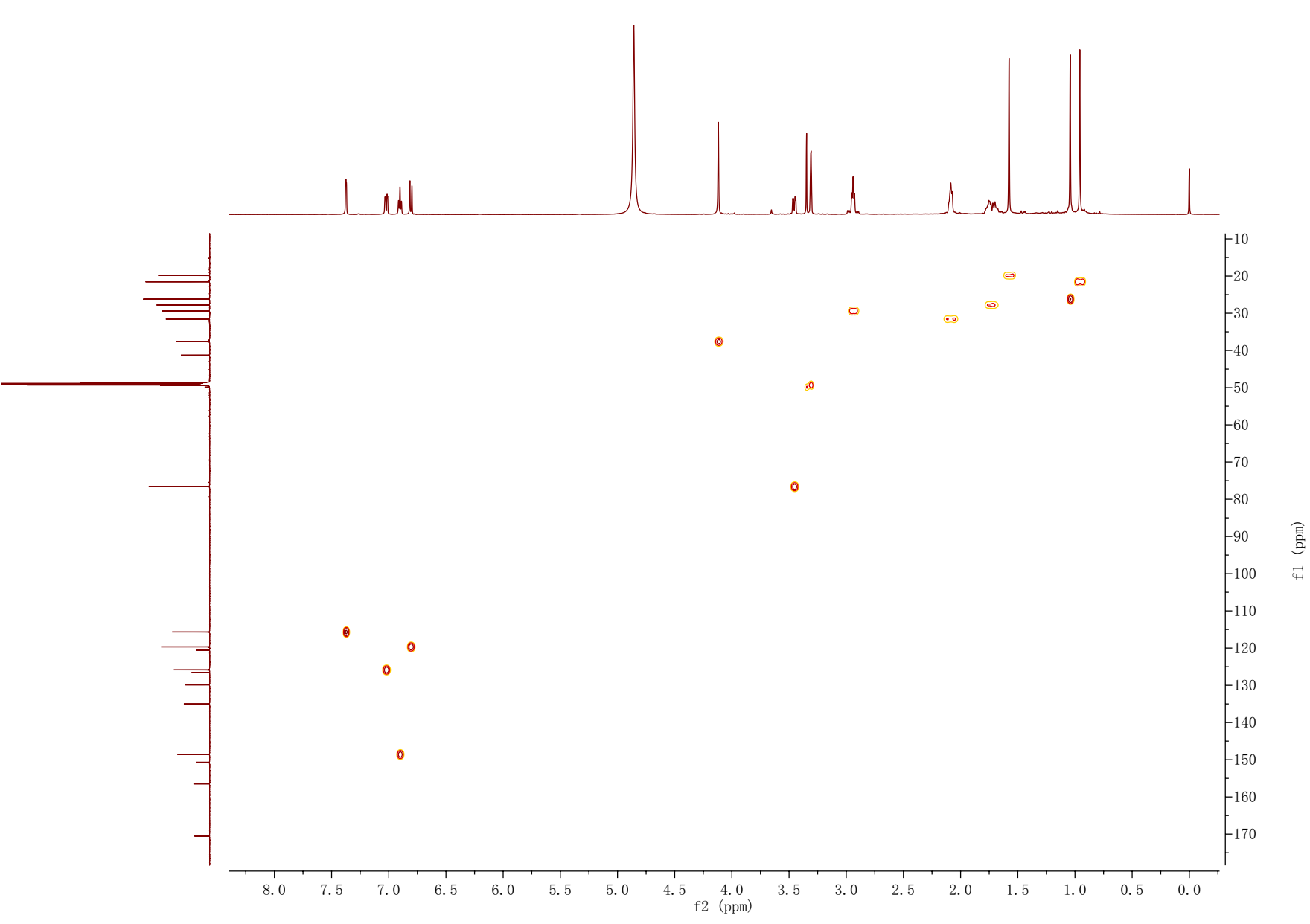


Figure S3. HSQC spectrum of **1** in methanol-*d*_4_.


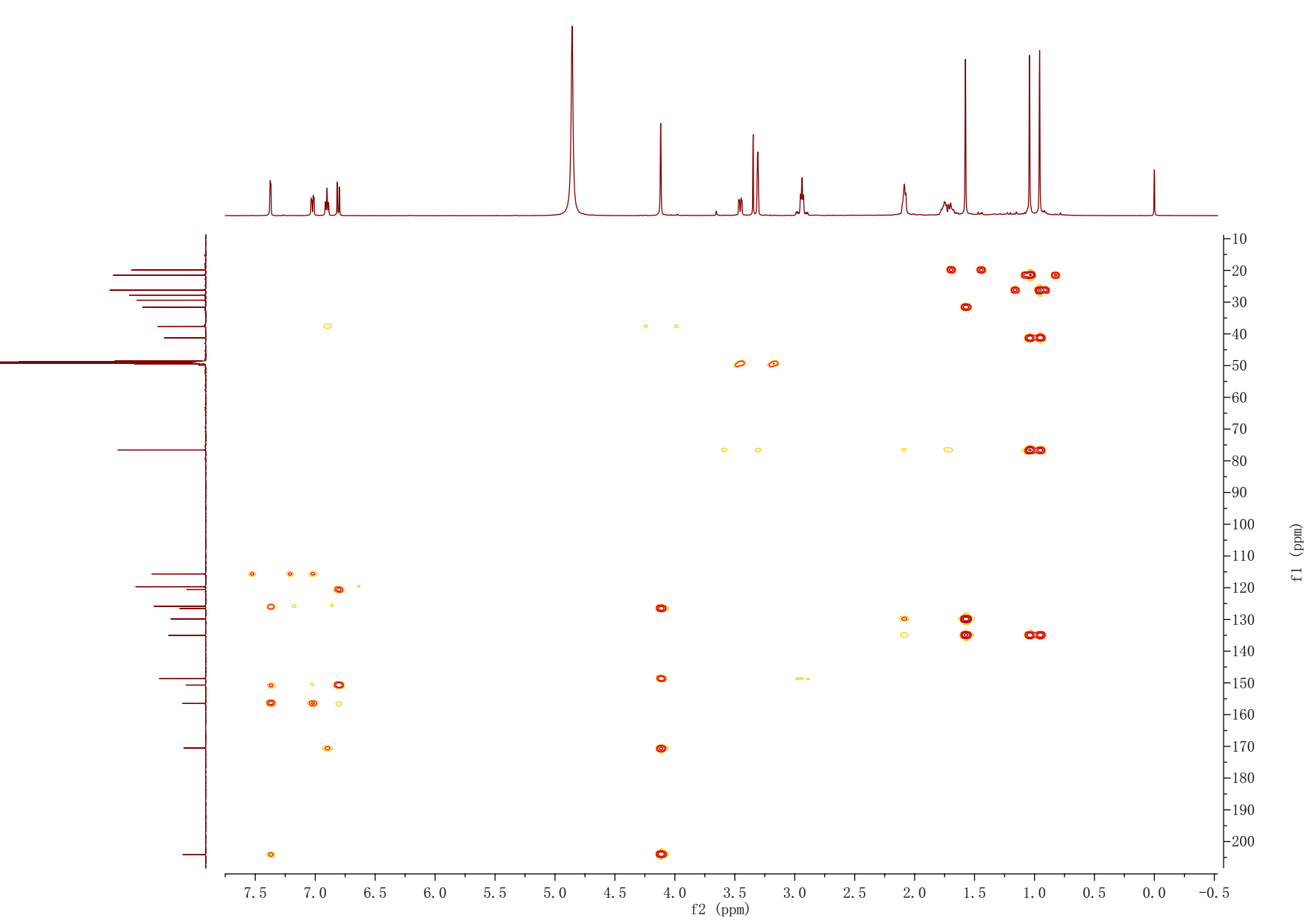


Figure S4. HMBC spectrum of **1** in methanol-*d*_4_.


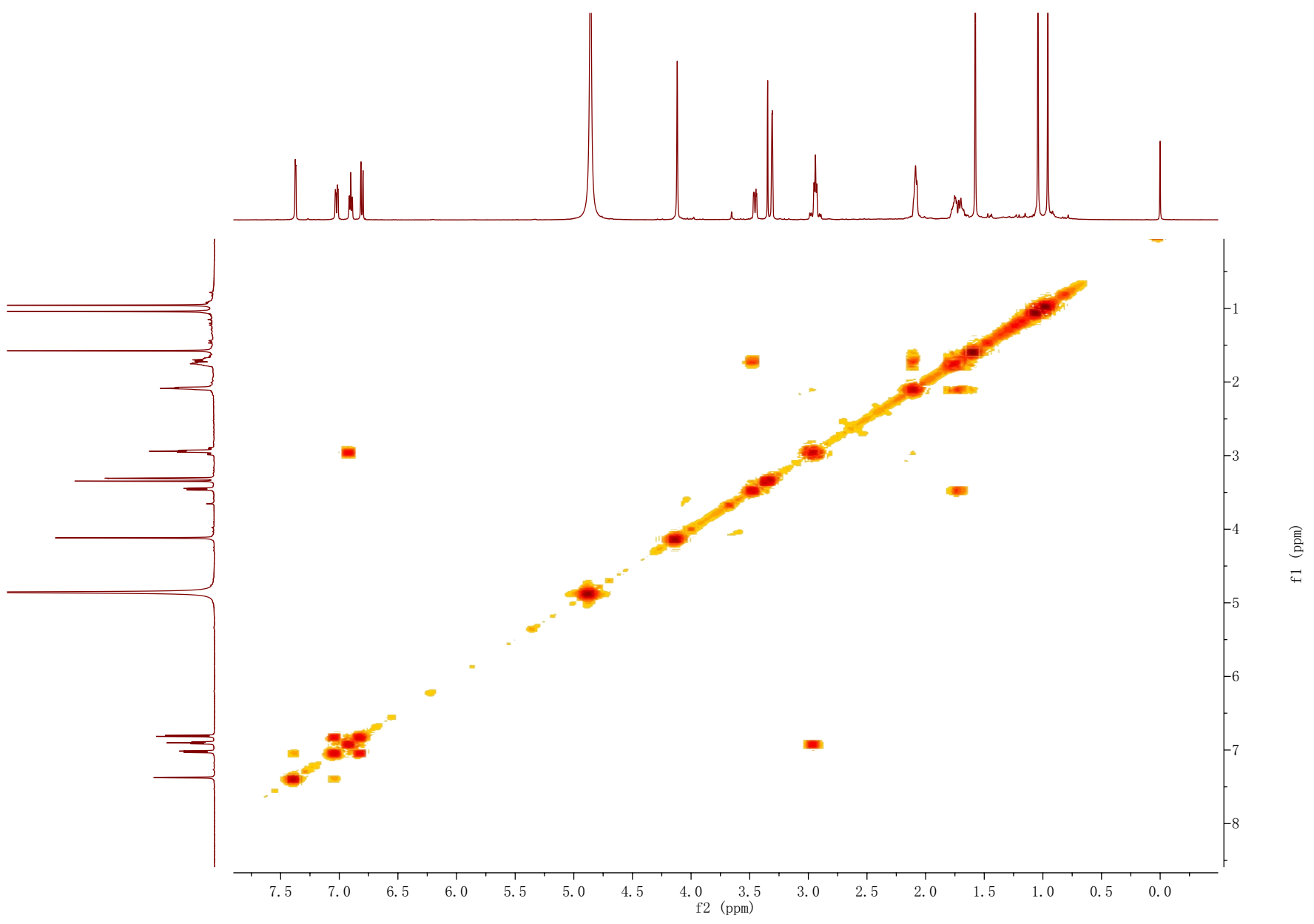


Figure S5. ^1^H-^1^H COSY spectrum of **1** in methanol-*d*_4_.


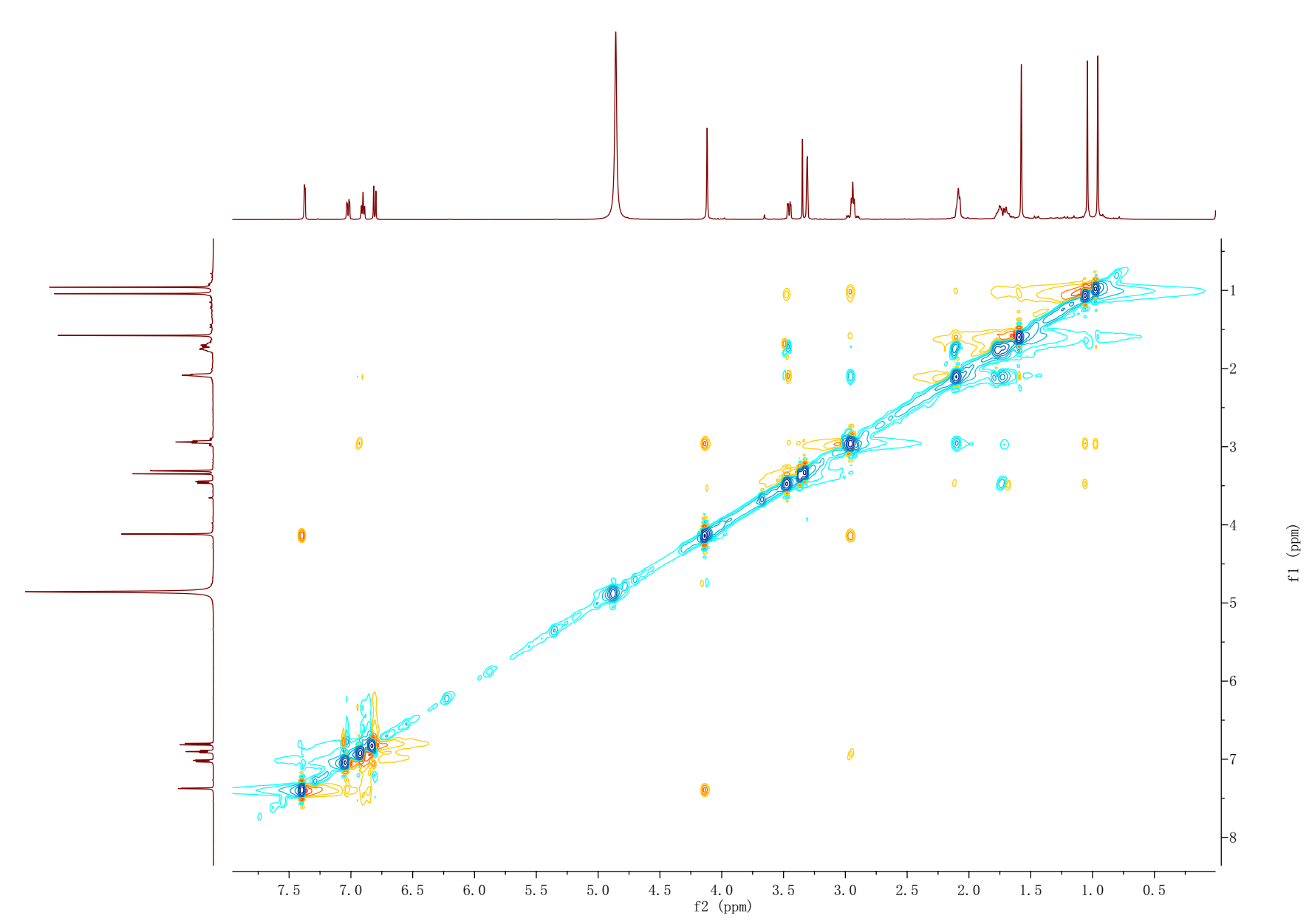


Figure S6. ROESY spectrum of **1** in methanol-*d*_4_.

[M+H]^+^ m/z 375.1812

| Hit | Formula | m/z | RDB | ppm |
| --- | --- | --- | --- | --- |
| 1 | C_21_H_27_O_6_ | 375.1802 | 9.0 | 2.6 |

Figure S7. HRESIMS of **1**.


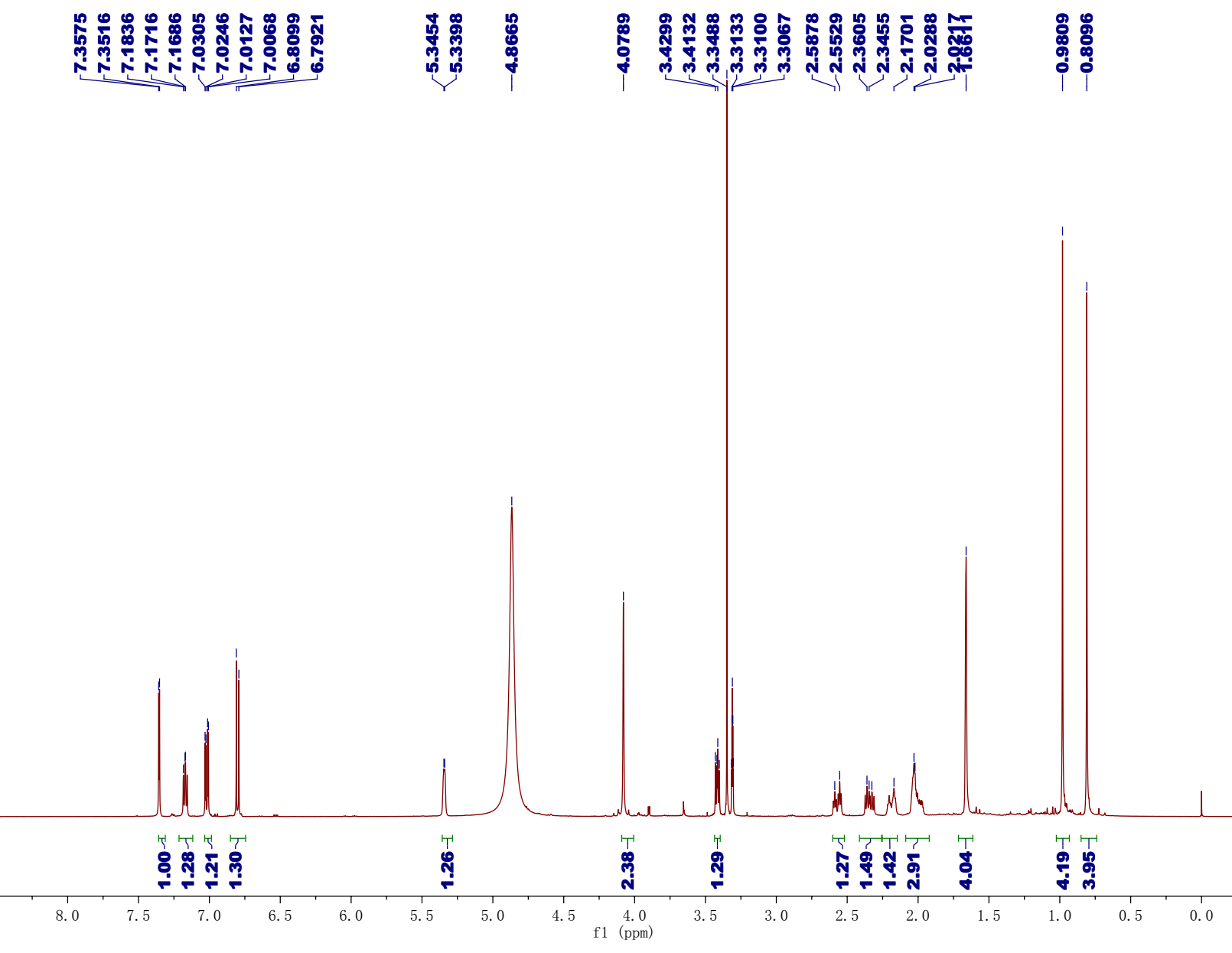


Figure S8. ^1^H NMR spectrum of **2** in methanol-*d*_4_.


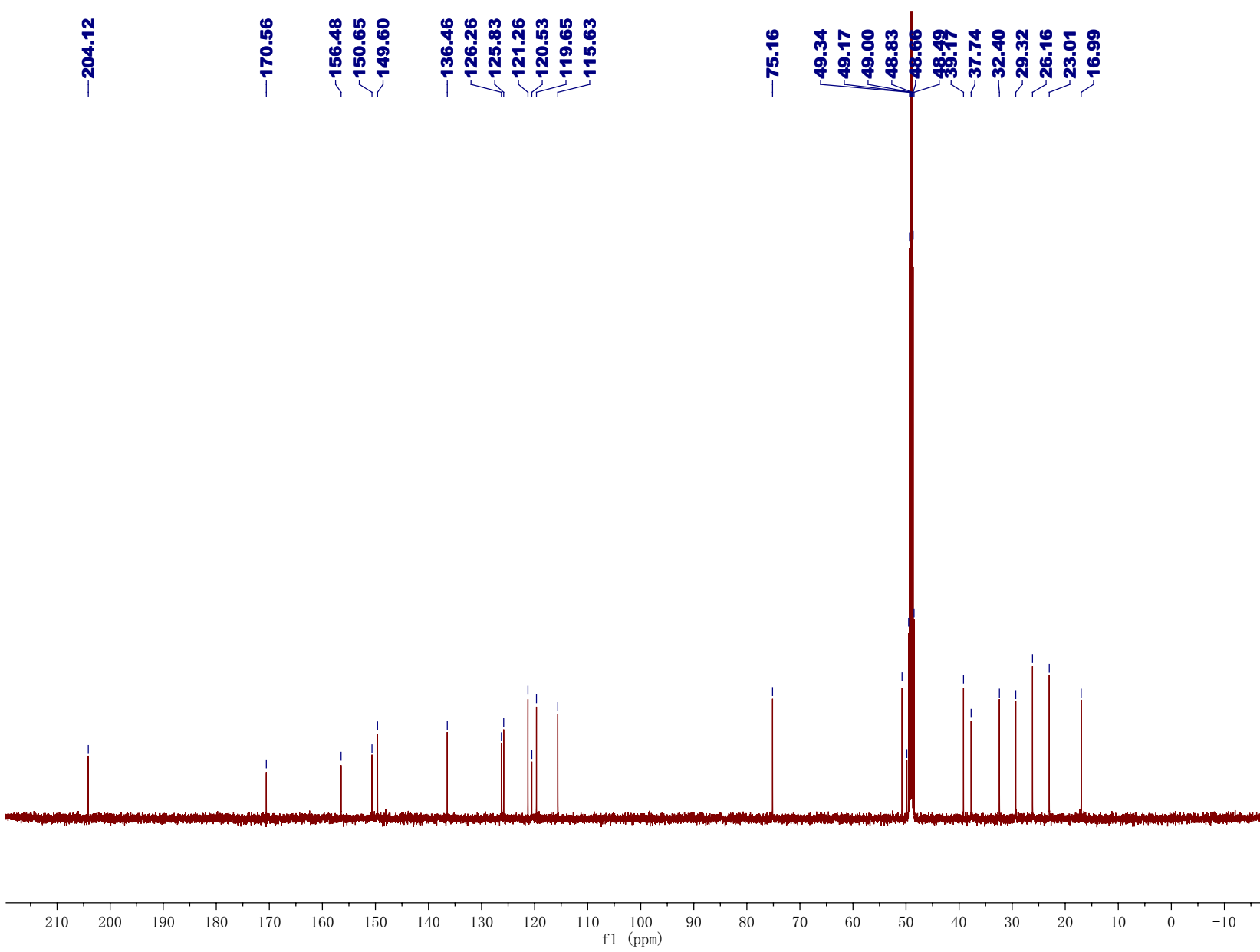


Figure S9. ^13^C NMR spectrum of **2** in methanol-*d*_4_.


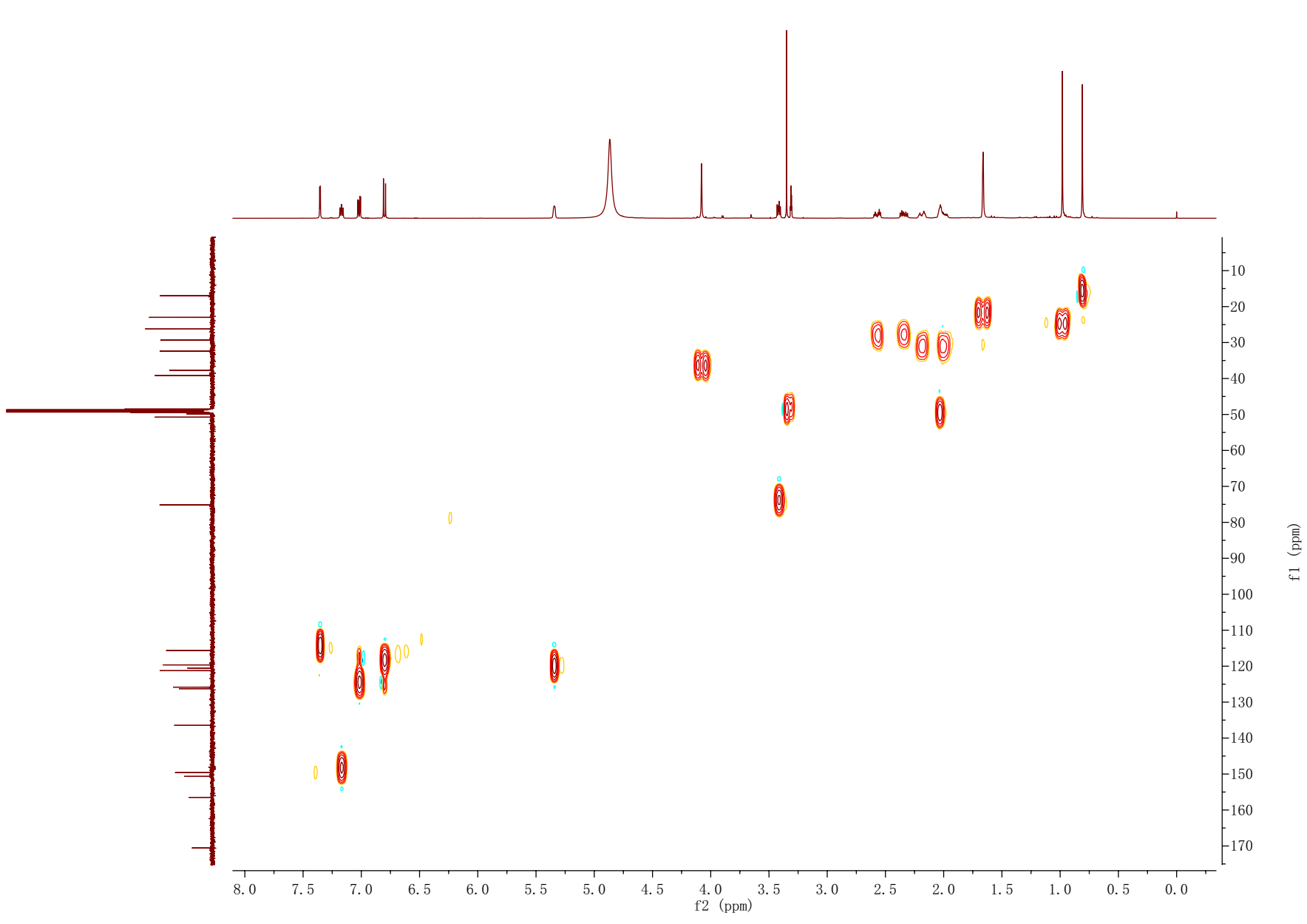


Figure S10. HSQC spectrum of **2** in methanol-*d*_4_.


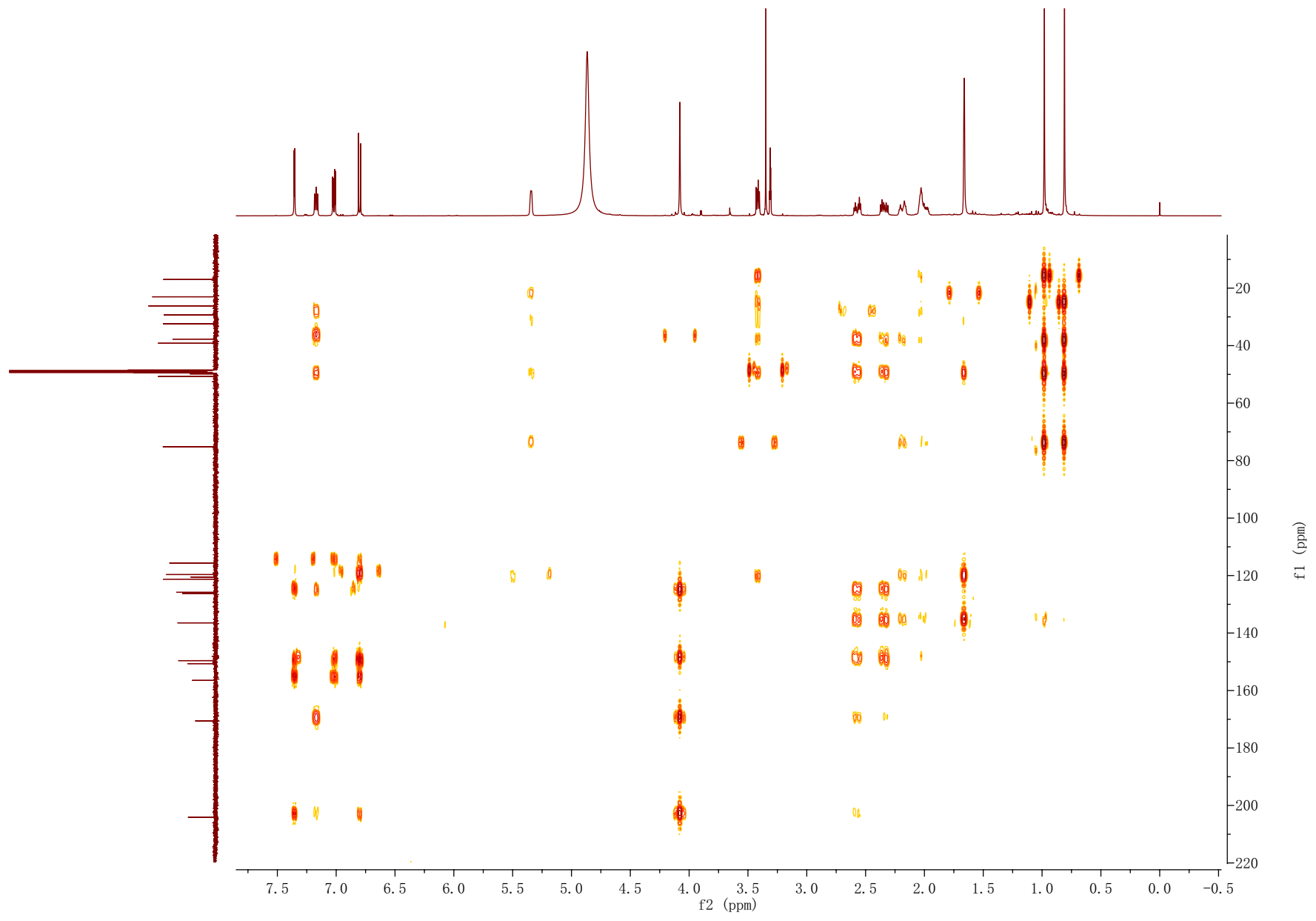


Figure S11. HMBC spectrum of **2** in methanol-*d*_4_.


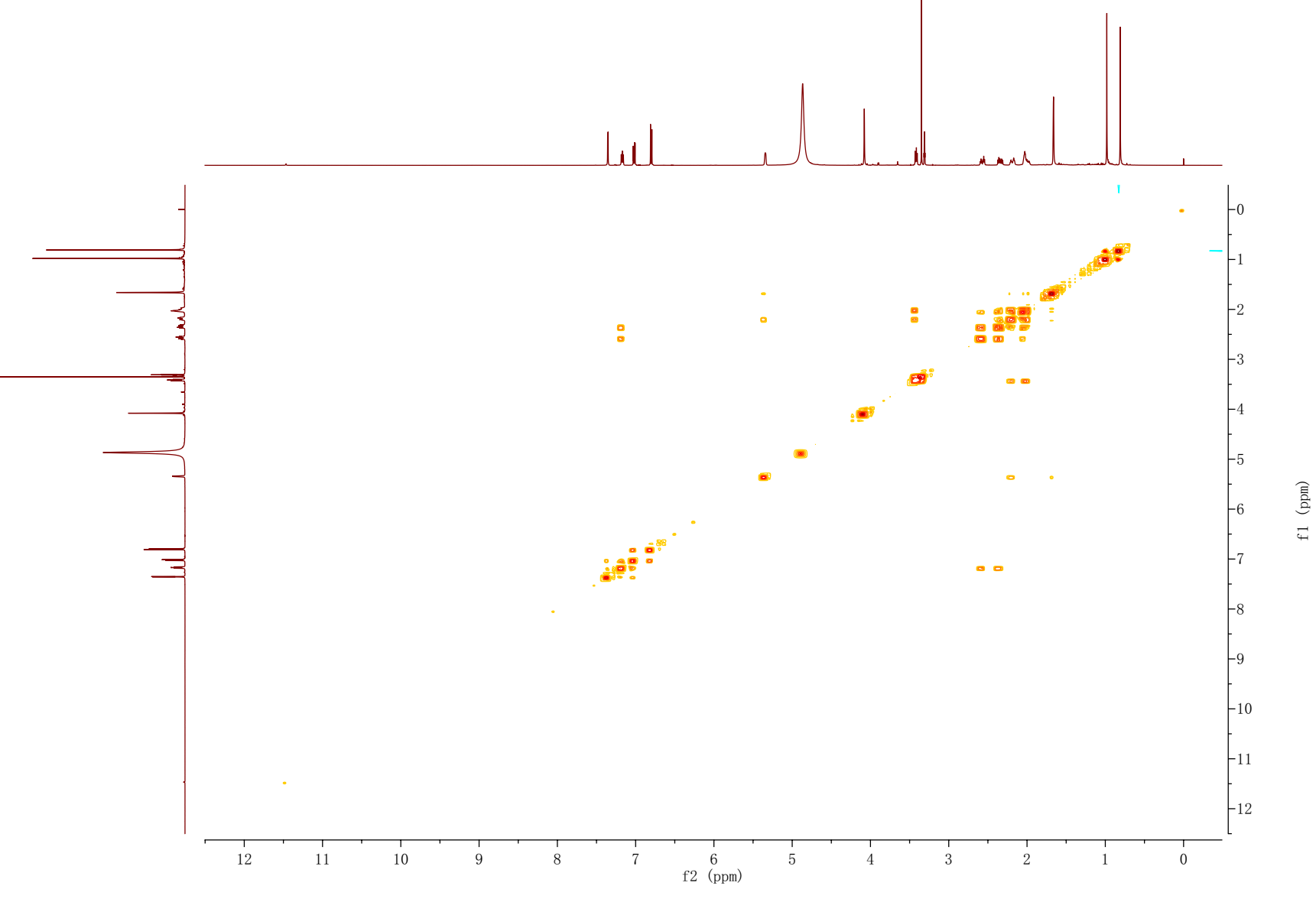


Figure S12. ^1^H-^1^H COSY spectrum of **2** in methanol-*d*_4_.


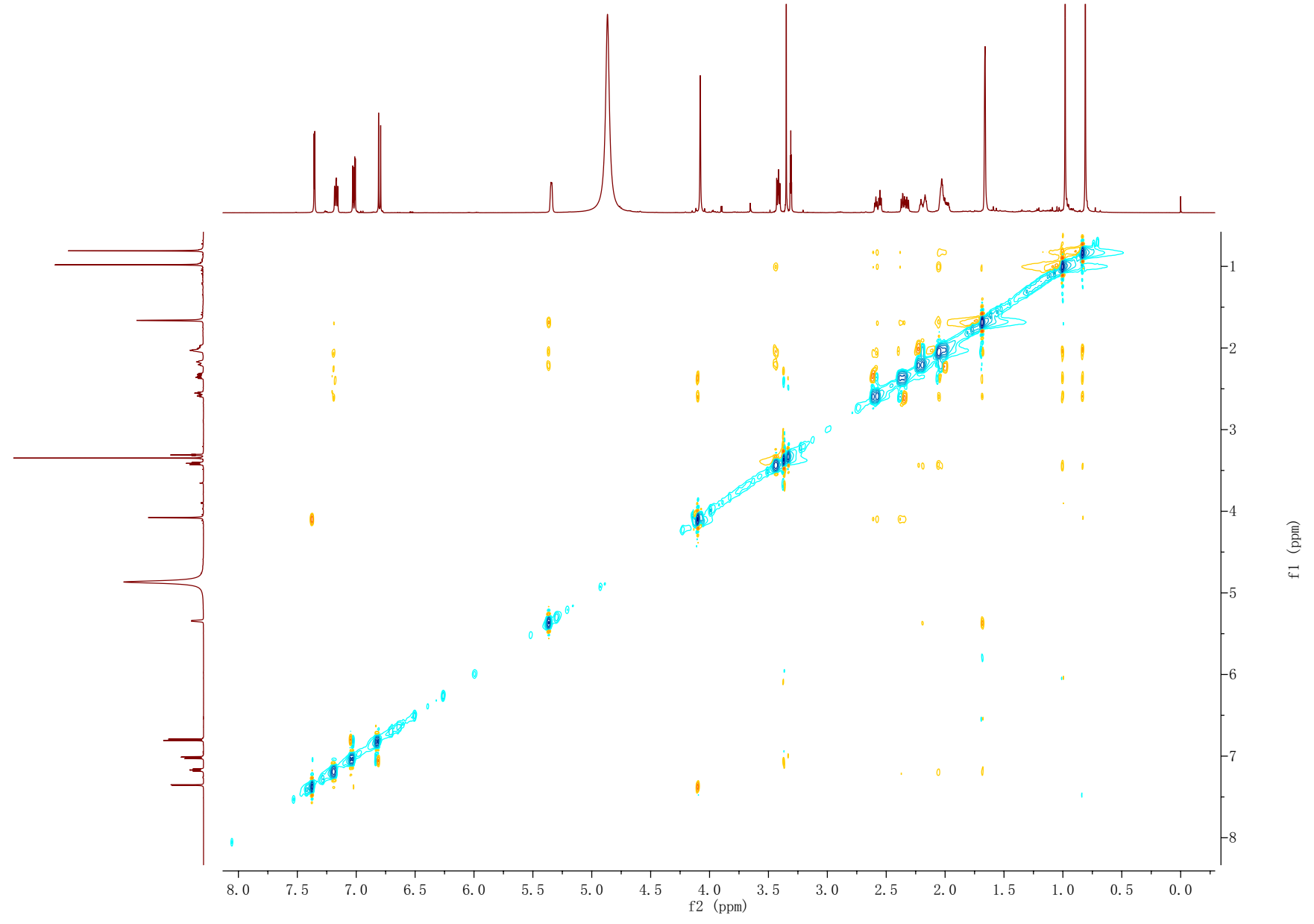


Figure S13. ROESY spectrum of **2** in methanol-*d*_4_.

[M+H]^+^ m/z 375.1810

| Hit | Formula | m/z | RDB | ppm |
| --- | --- | --- | --- | --- |
| 1 | C_21_H_27_O_6_ | 375.1802 | 9.0 | 2.1 |

Figure S14. HRESIMS of **2**


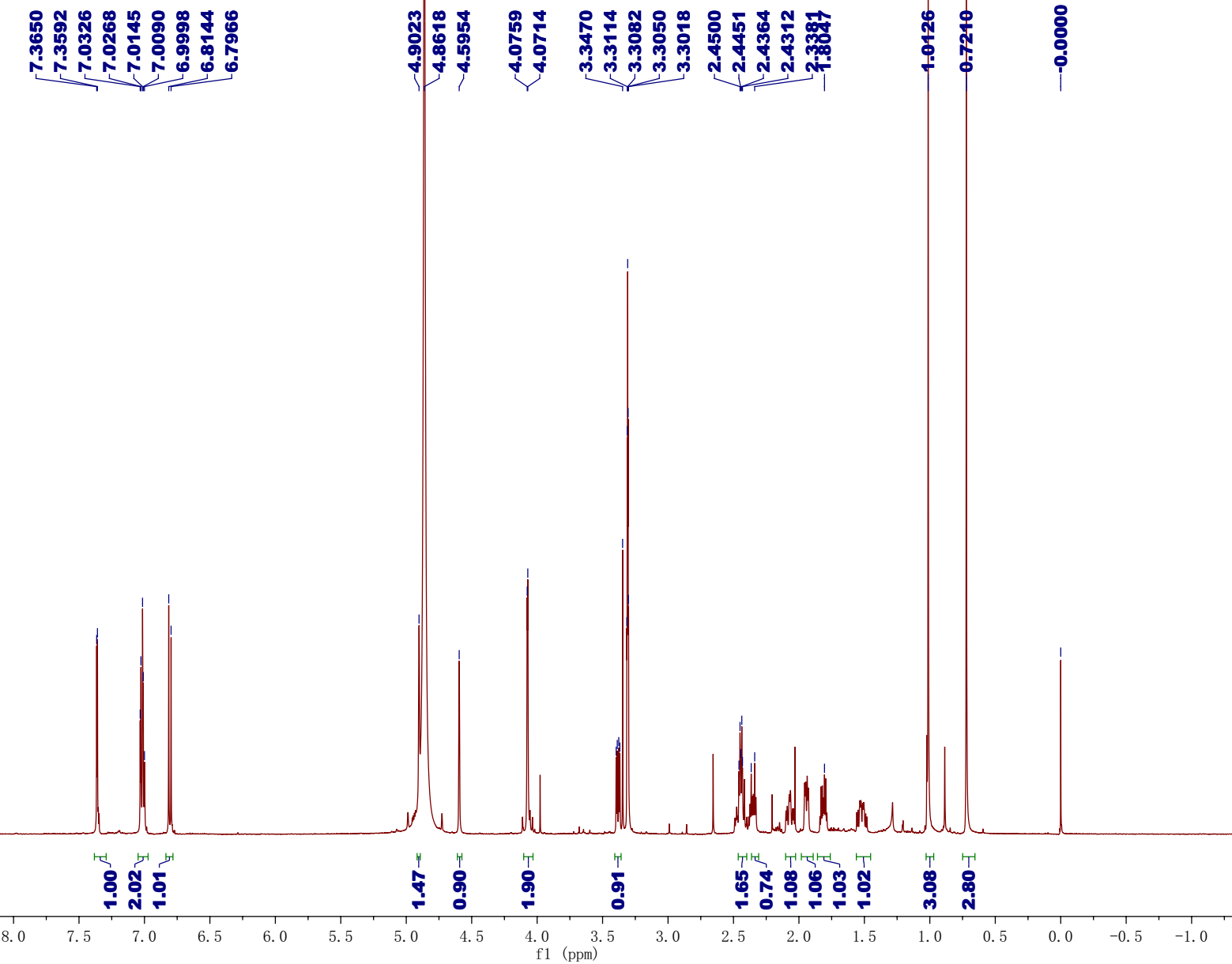


Figure S15 ^1^H NMR spectrum of **3** in methanol-*d*_4_.


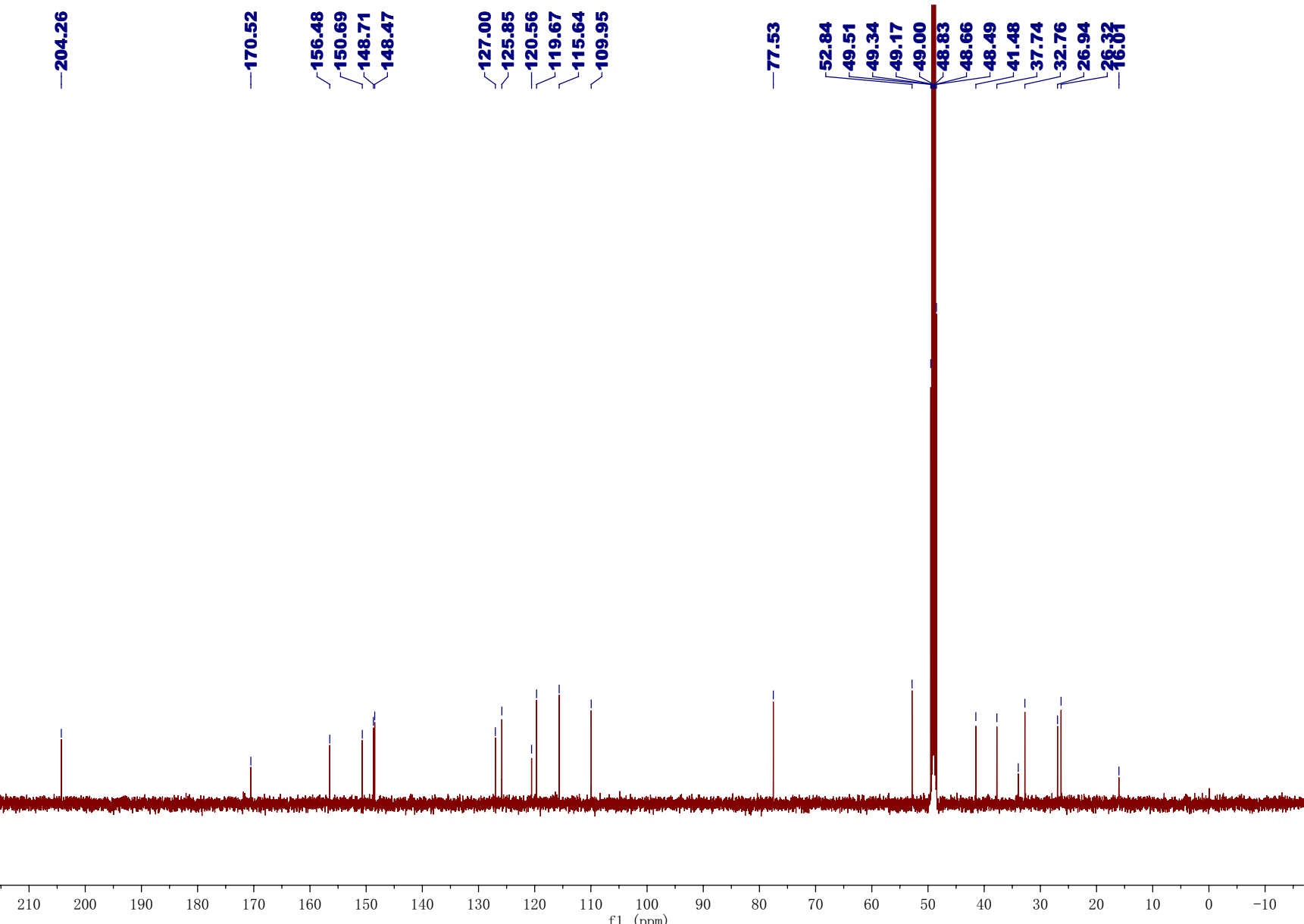


Figure S16. ^13^C NMR spectrum of **3** in methanol-*d*_4_.


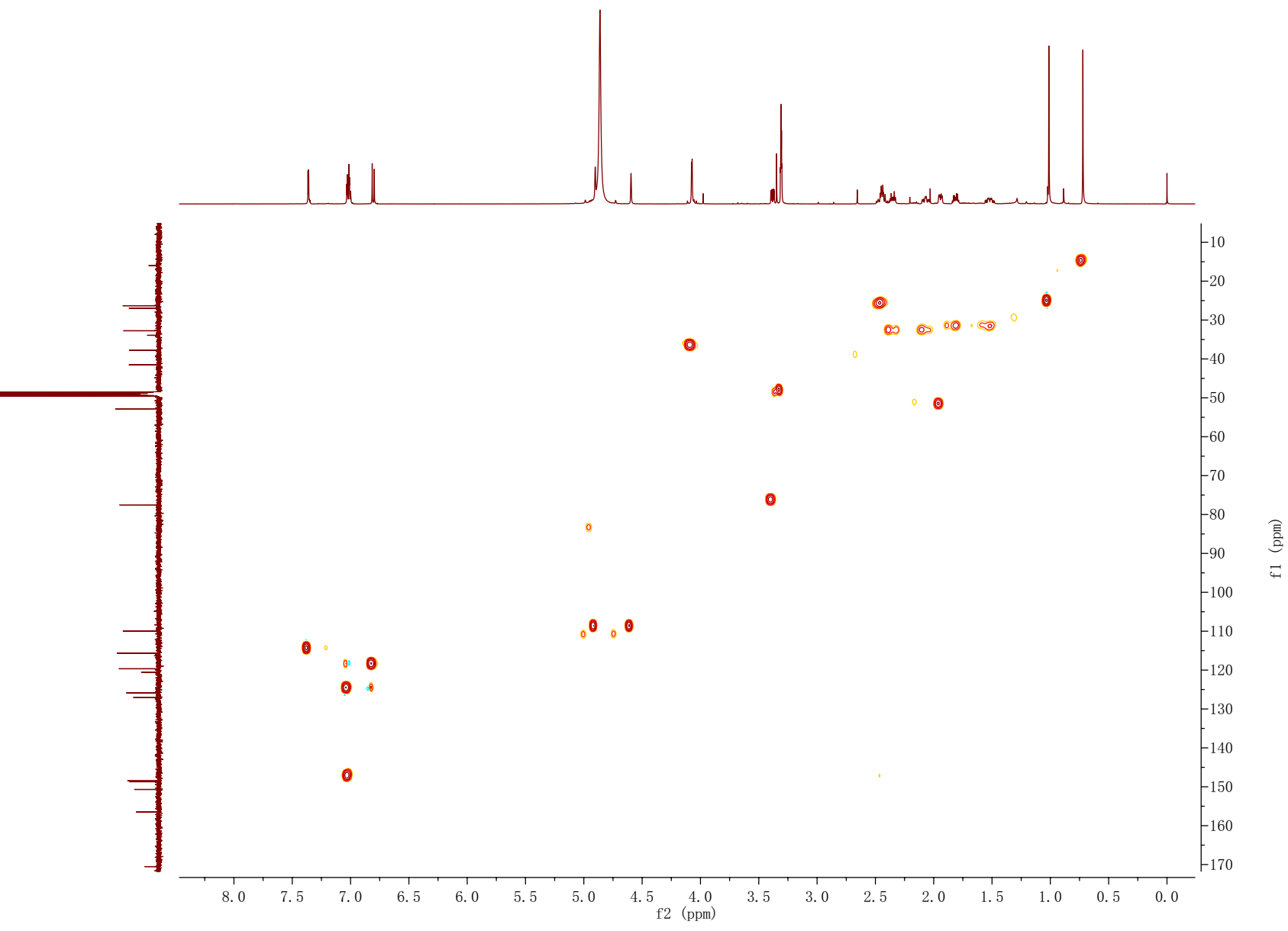


Figure S17. HSQC spectrum of **3** in methanol-*d*_4_.


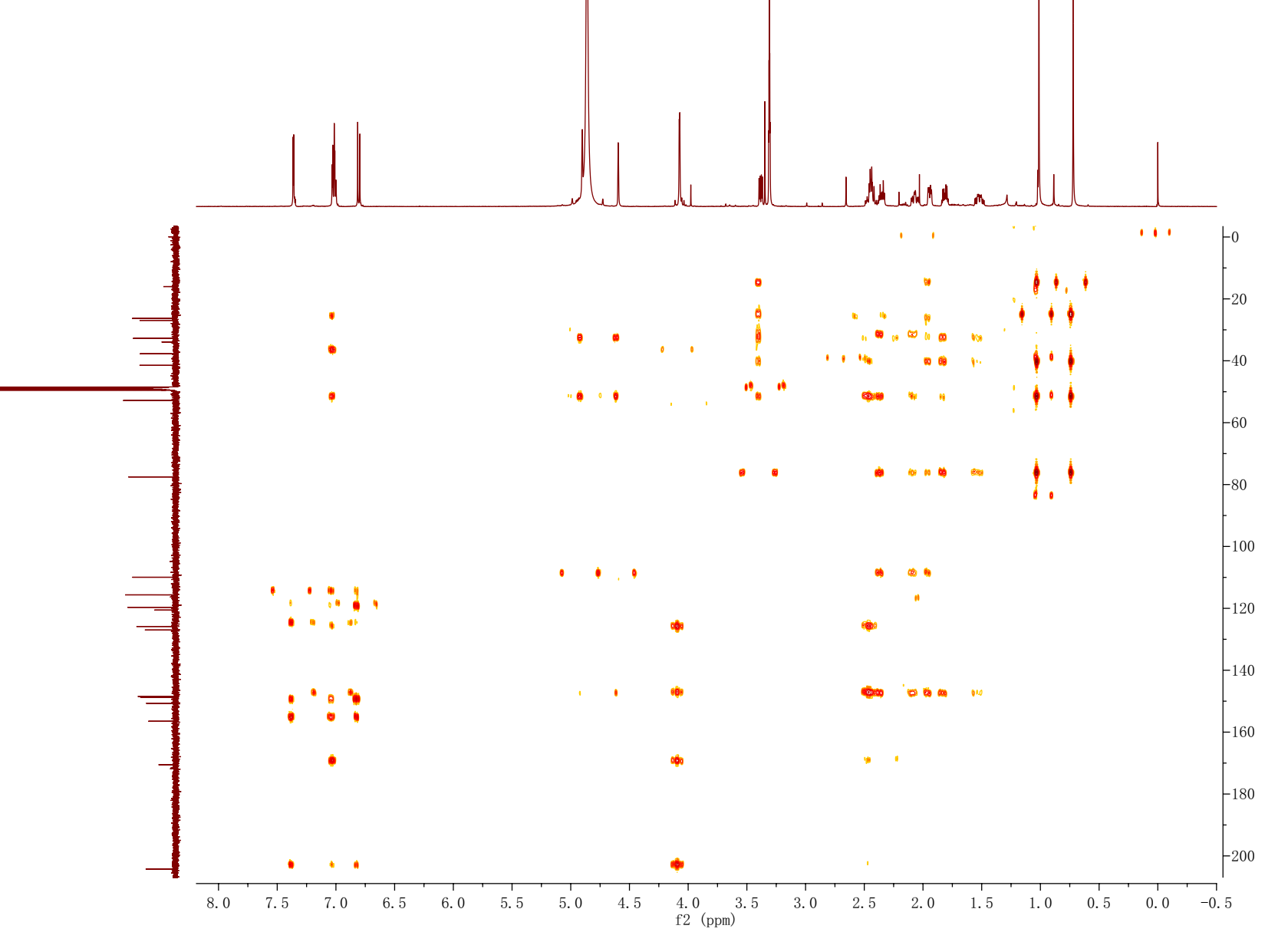


Figure S18. HMBC spectrum of **3** in methanol-*d*_4_.


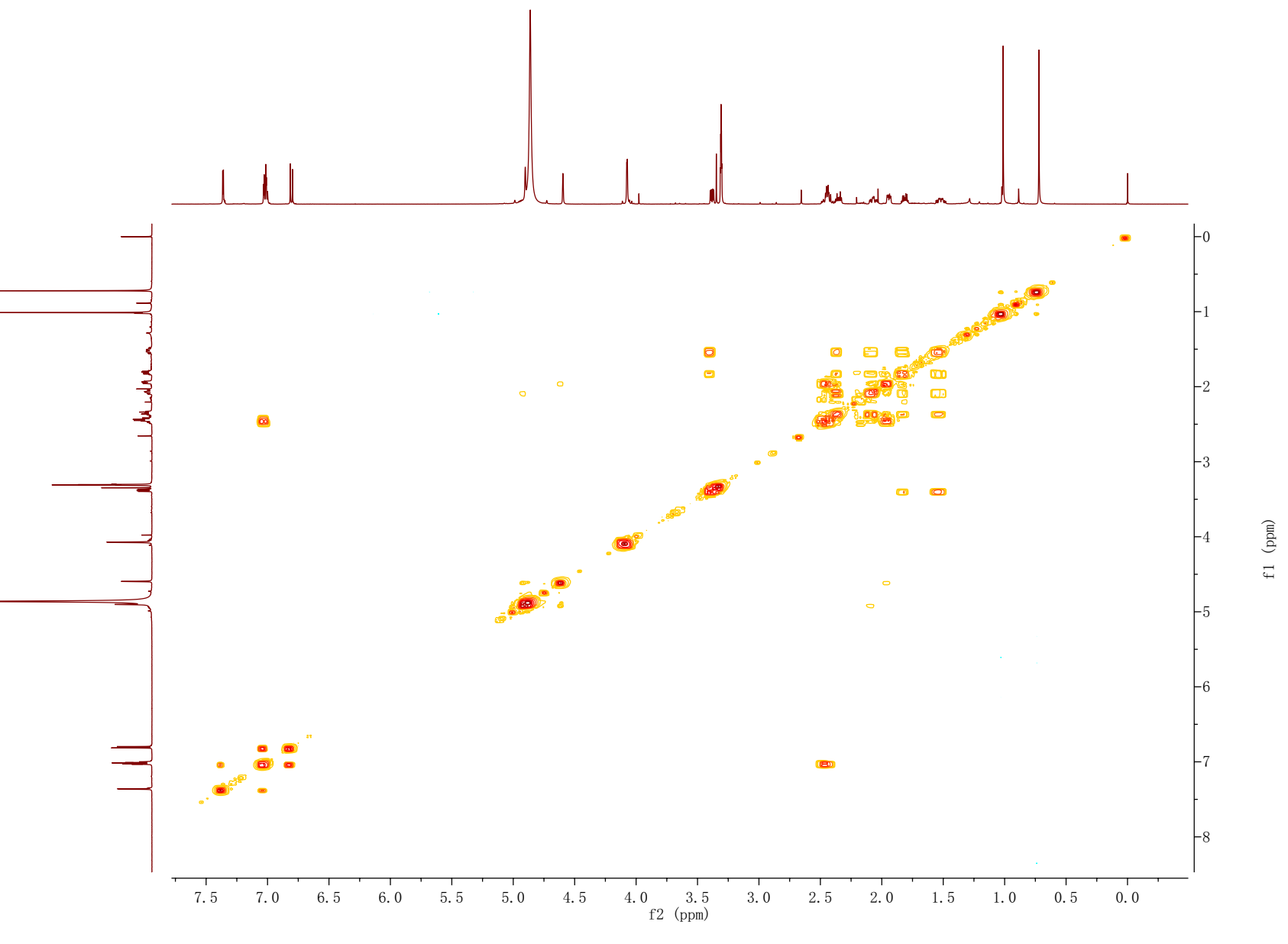


Figure S19. ^1^H-^1^H COSY spectrum of **3** in methanol-*d*_4_.


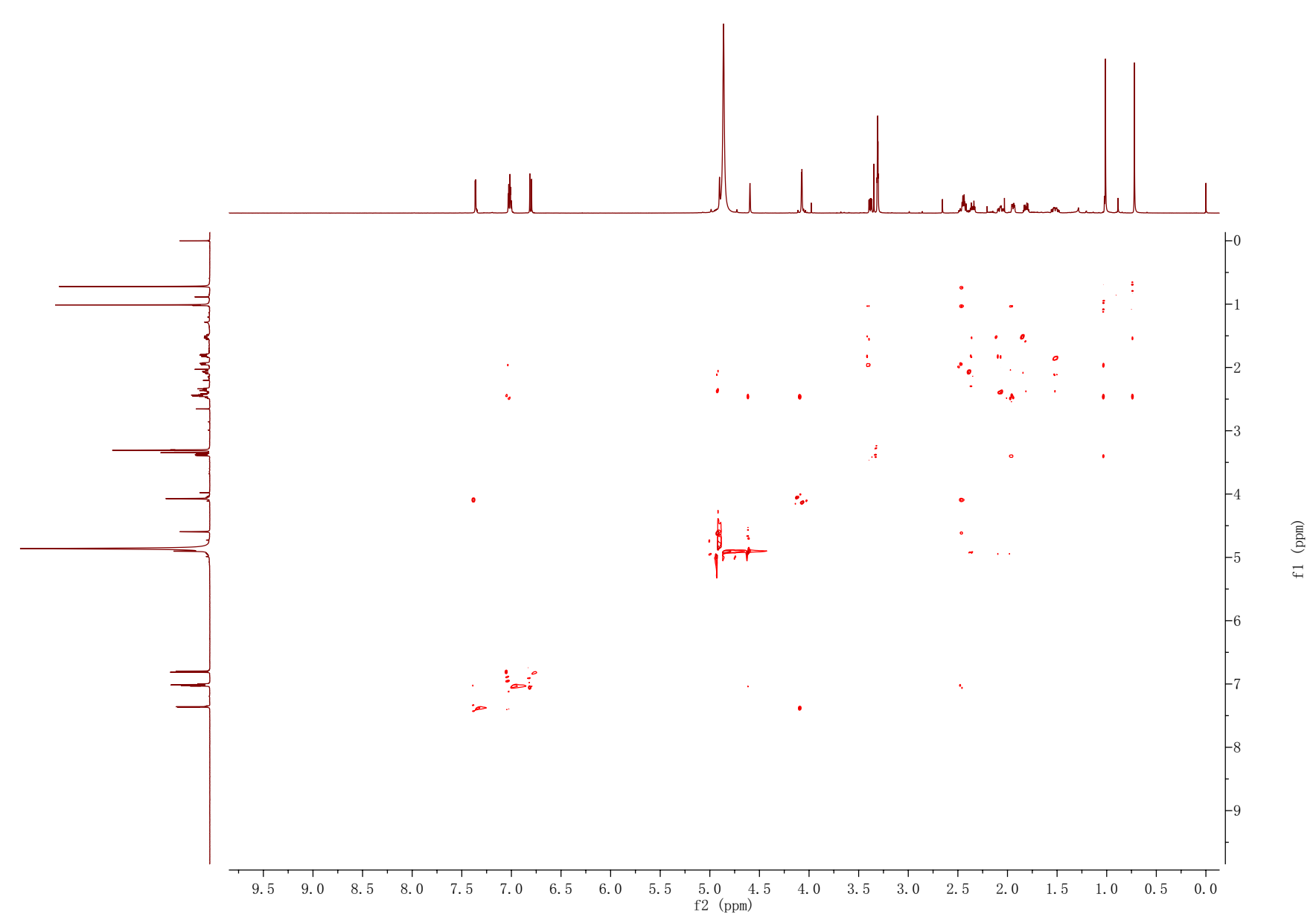


Figure S20. ROESY spectrum of **3** in methanol-*d*_4_.


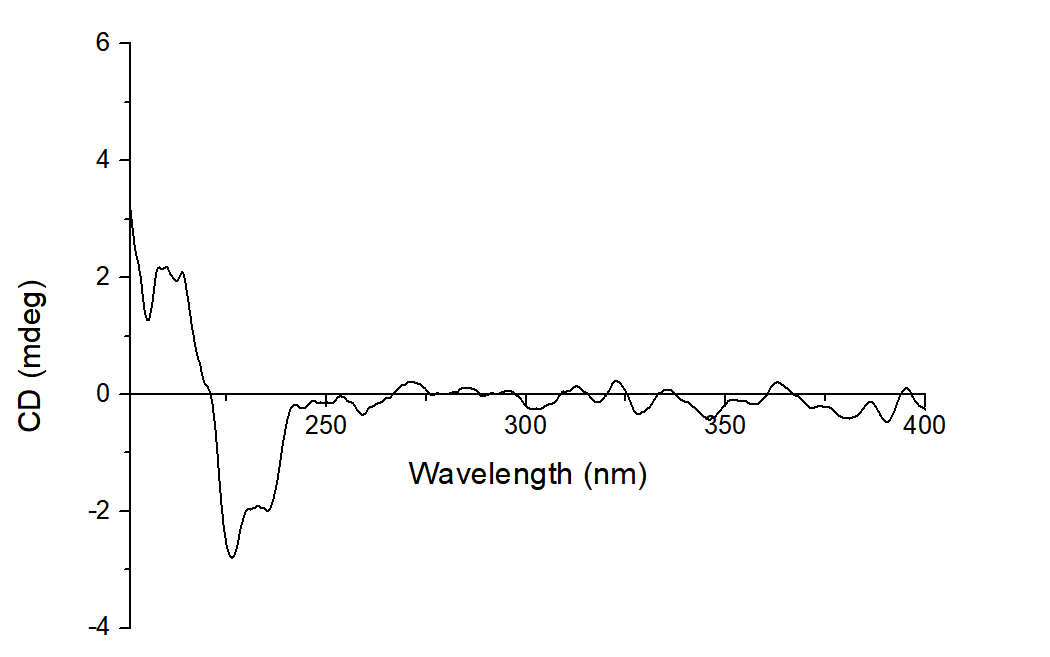


Figure S21. CD spectrum of **3**.

[M+H]^+^ m/z 375.1804

| Hit | Formula | m/z | RDB | ppm |
| --- | --- | --- | --- | --- |
| 1 | C_21_H_27_O_6_ | 375.1802 | 9.0 | 0.5 |

Figure S22. HRESIMS of **3**.


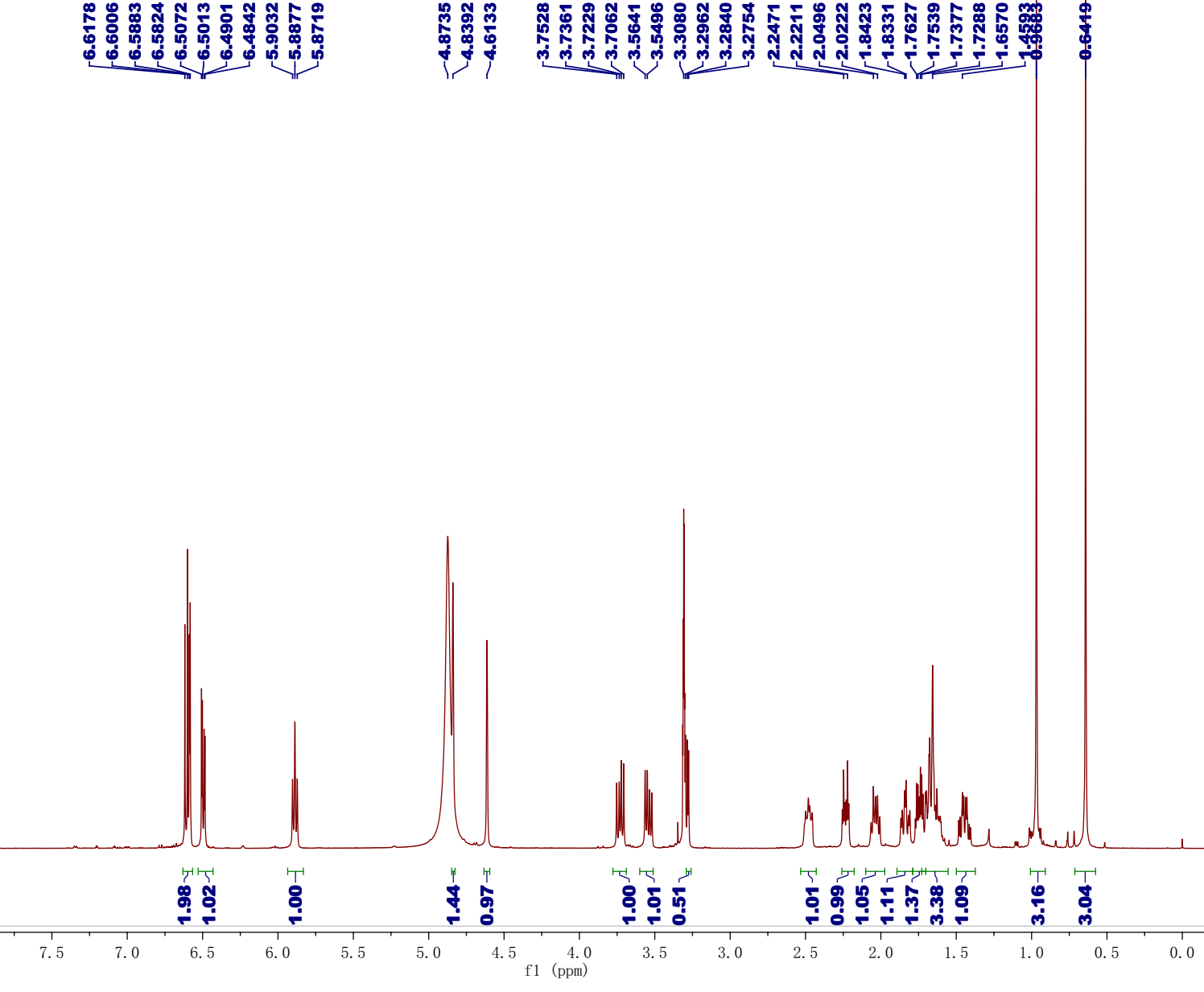


Figure S23. ^1^H NMR spectrum of **4** in methanol-*d*_4_.


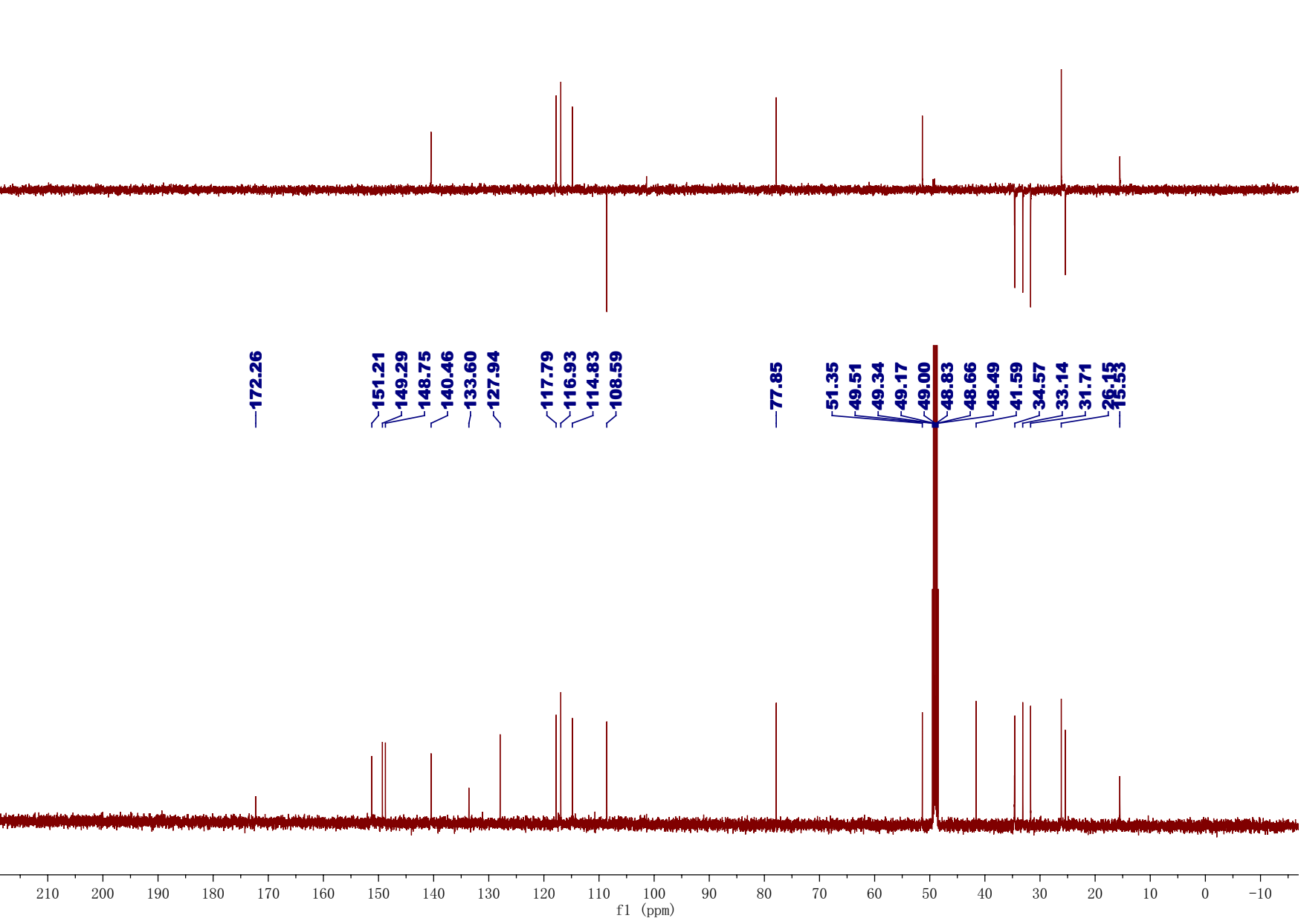


Figure S24. ^13^C NMR and DEPT spectra of **4** in methanol-*d*_4_.


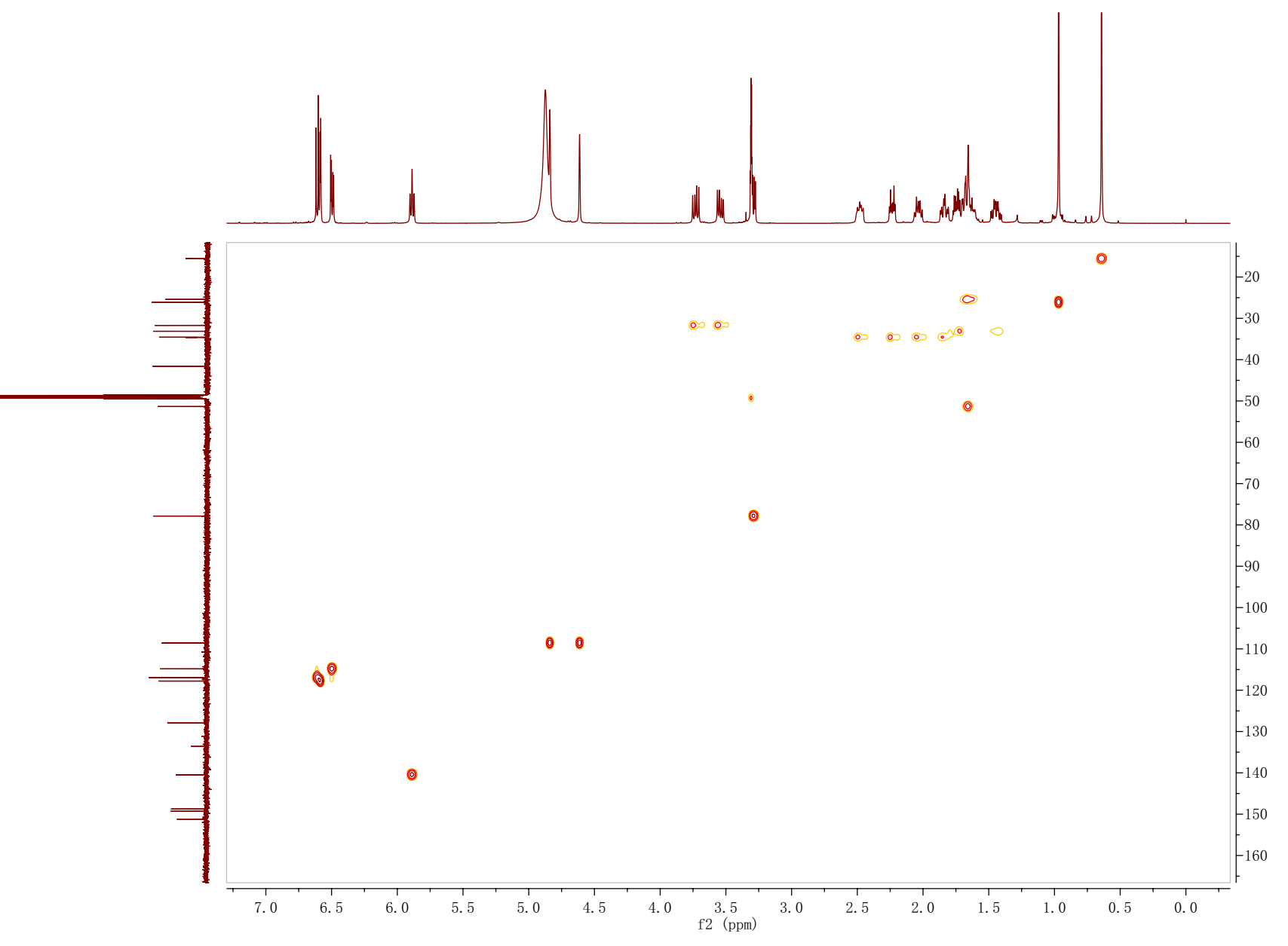


Figure S25. HSQC spectrum of **4** in methanol-*d*_4_.


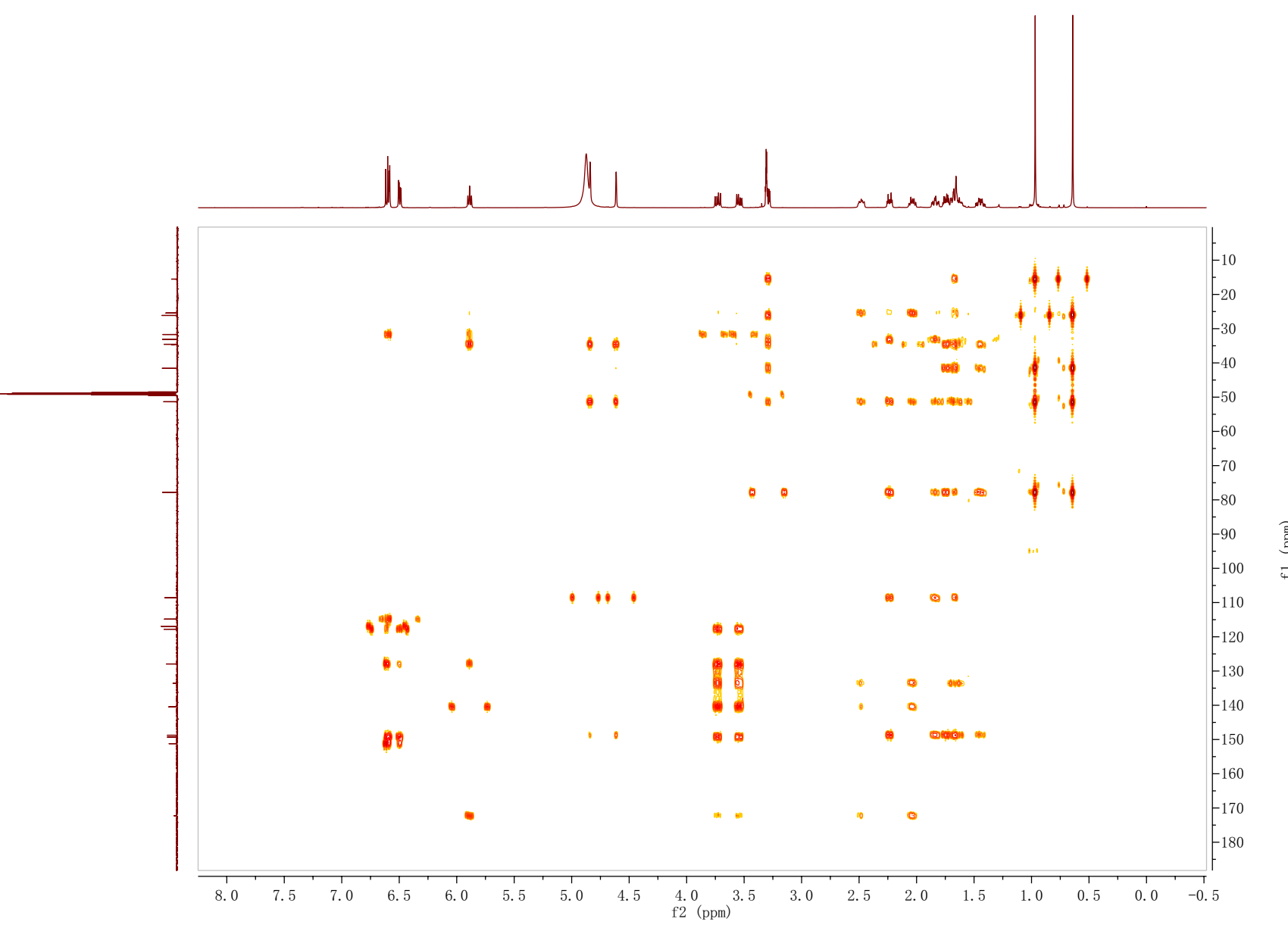


Figure S26. HMBC spectrum of **4** in methanol-*d*_4_.


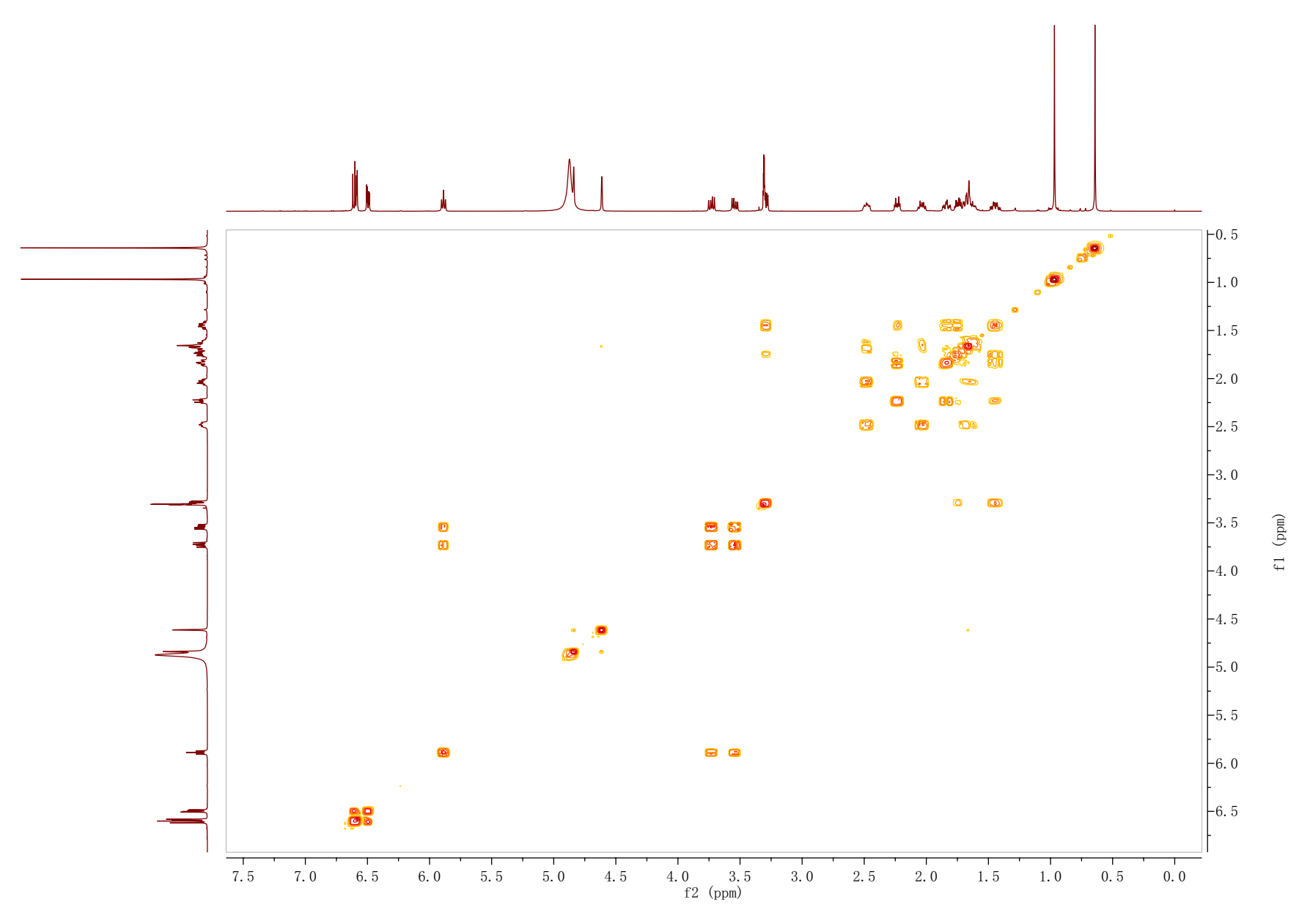


Figure S27. ^1^H-^1^H COSY spectrum of **4** in methanol-*d*_4_.


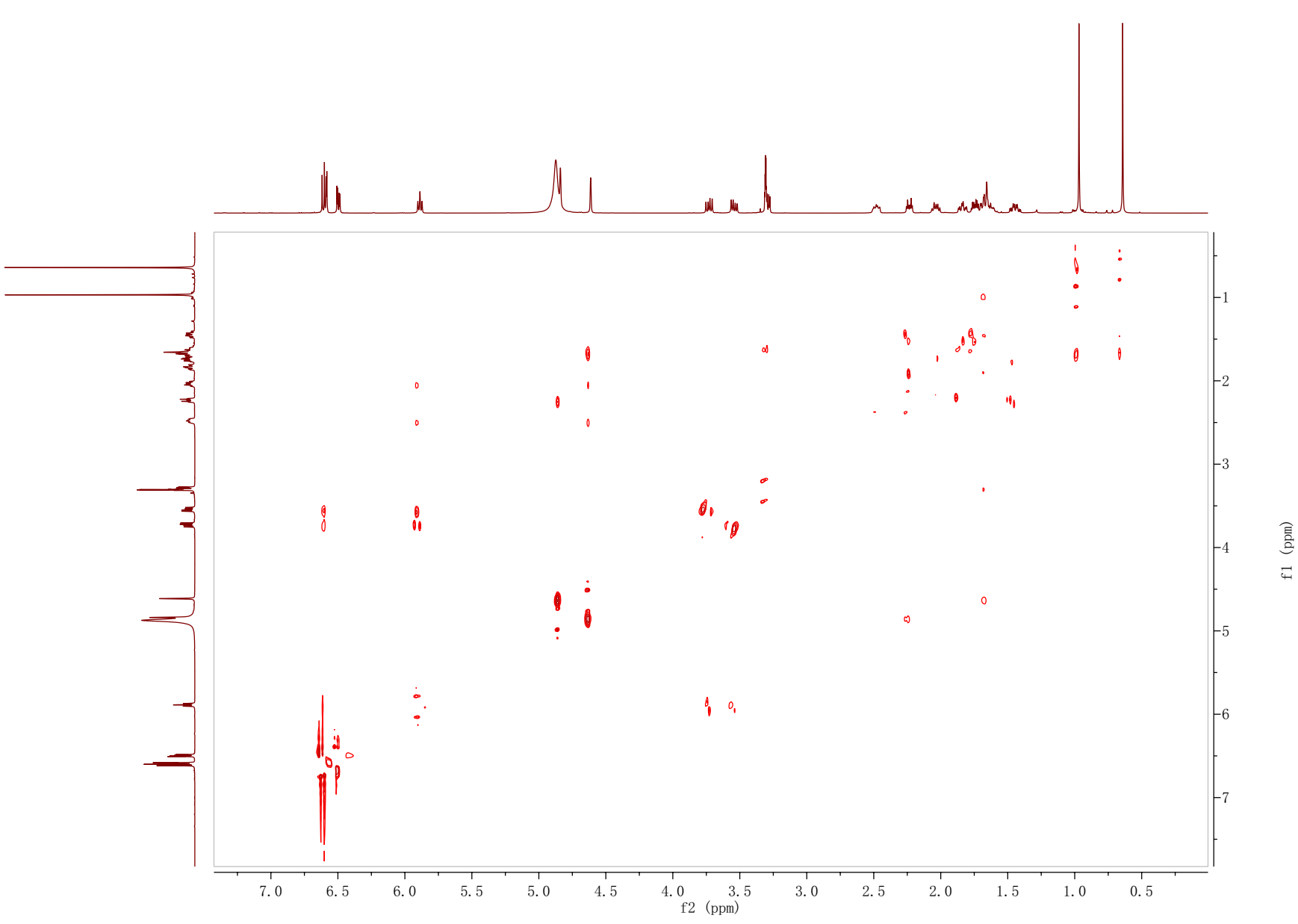


Figure S28. ROESY spectrum of **4** in methanol-*d*_4_.

[M+H]^+^ m/z 361.2012

| Hit | Formula | m/z | RDB | ppm |
| --- | --- | --- | --- | --- |
| 1 | C_21_H_29_O_5_ | 361.2010 | 8.0 | 0.7 |

Figure S29. HRESIMS of **4**.


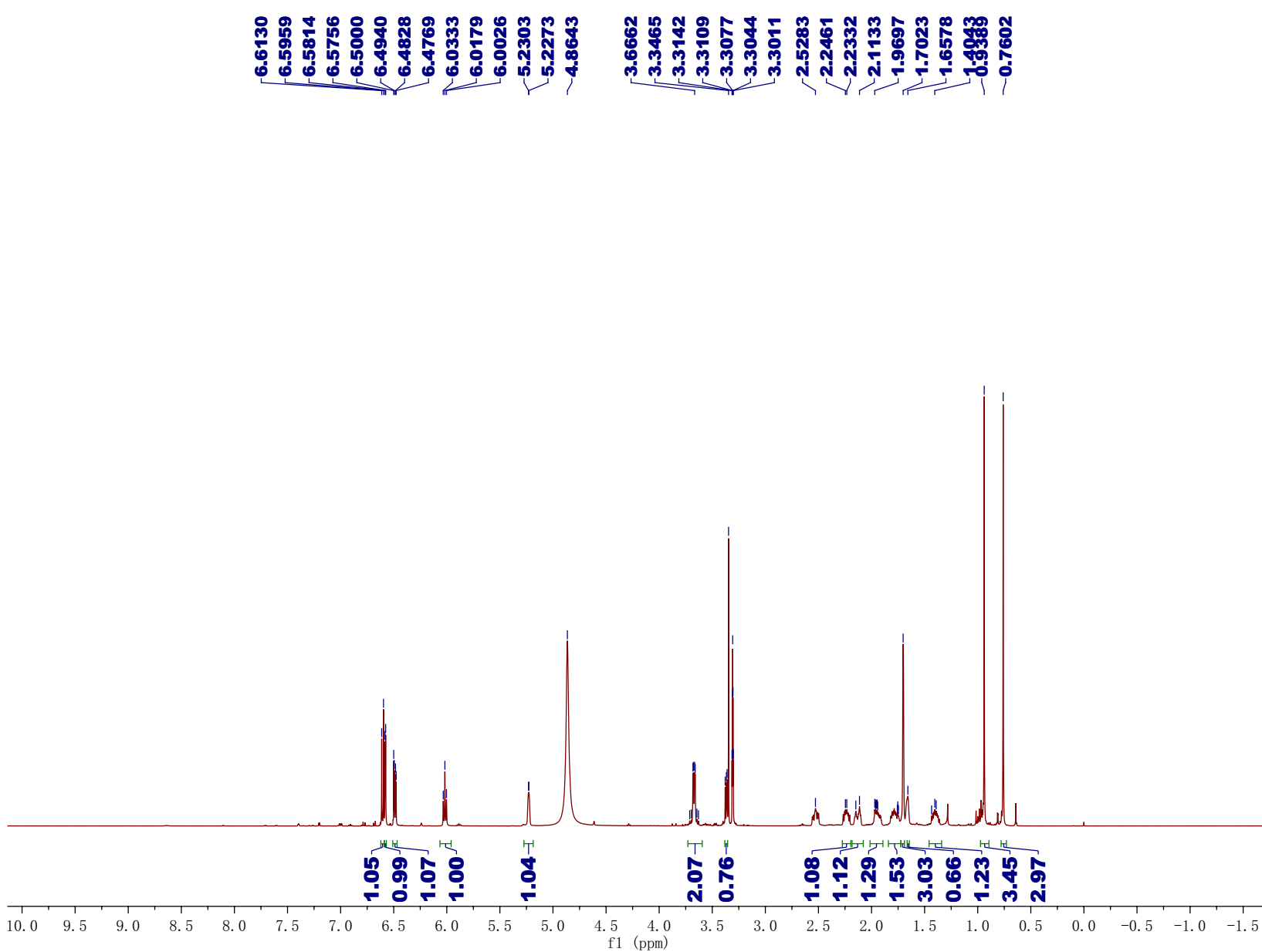


Figure S30. ^1^H NMR spectrum of **5** in methanol-*d*_4_.


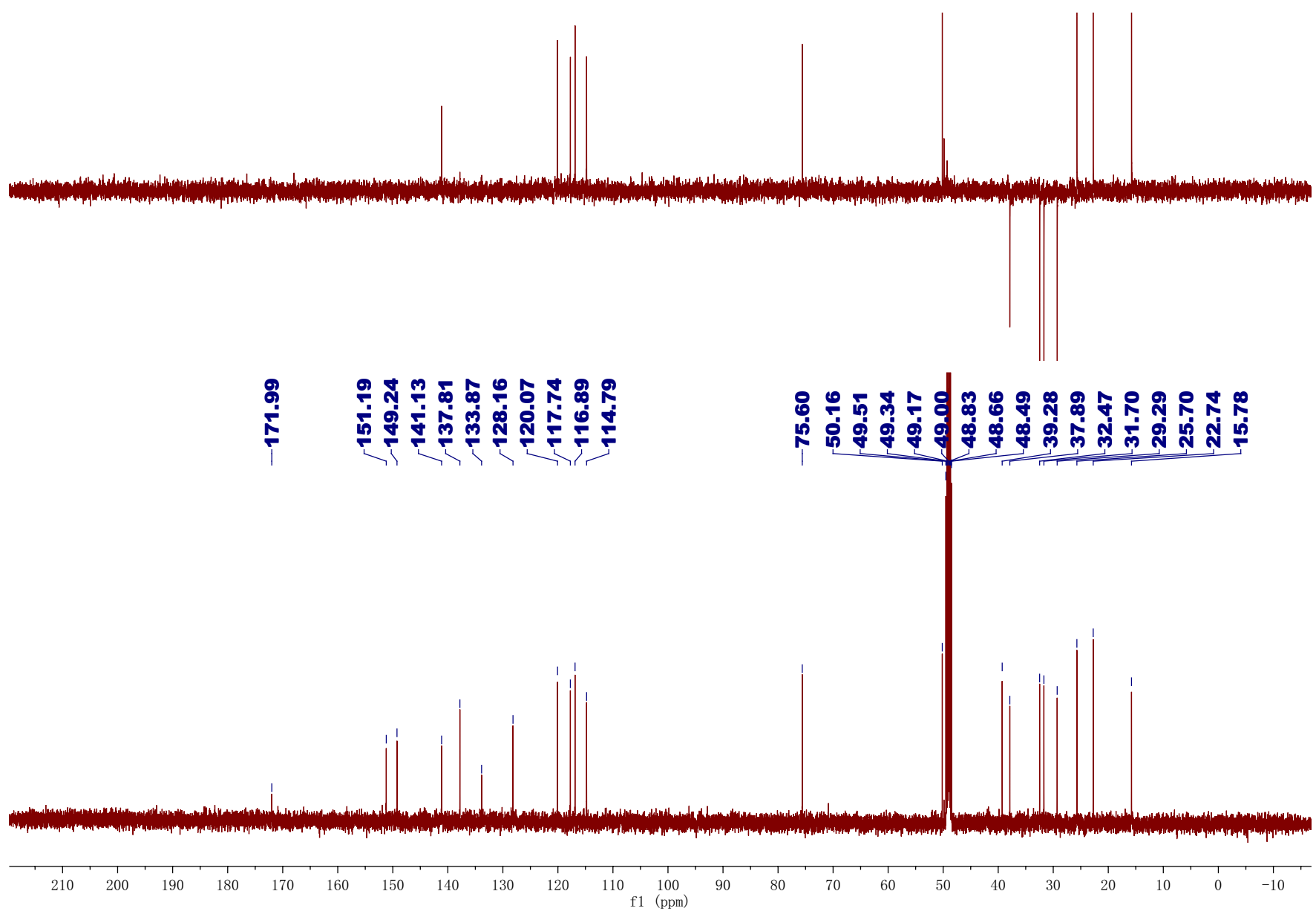


Figure S31. ^13^C NMR and DEPT spectra of **5** in methanol-*d*_4_.


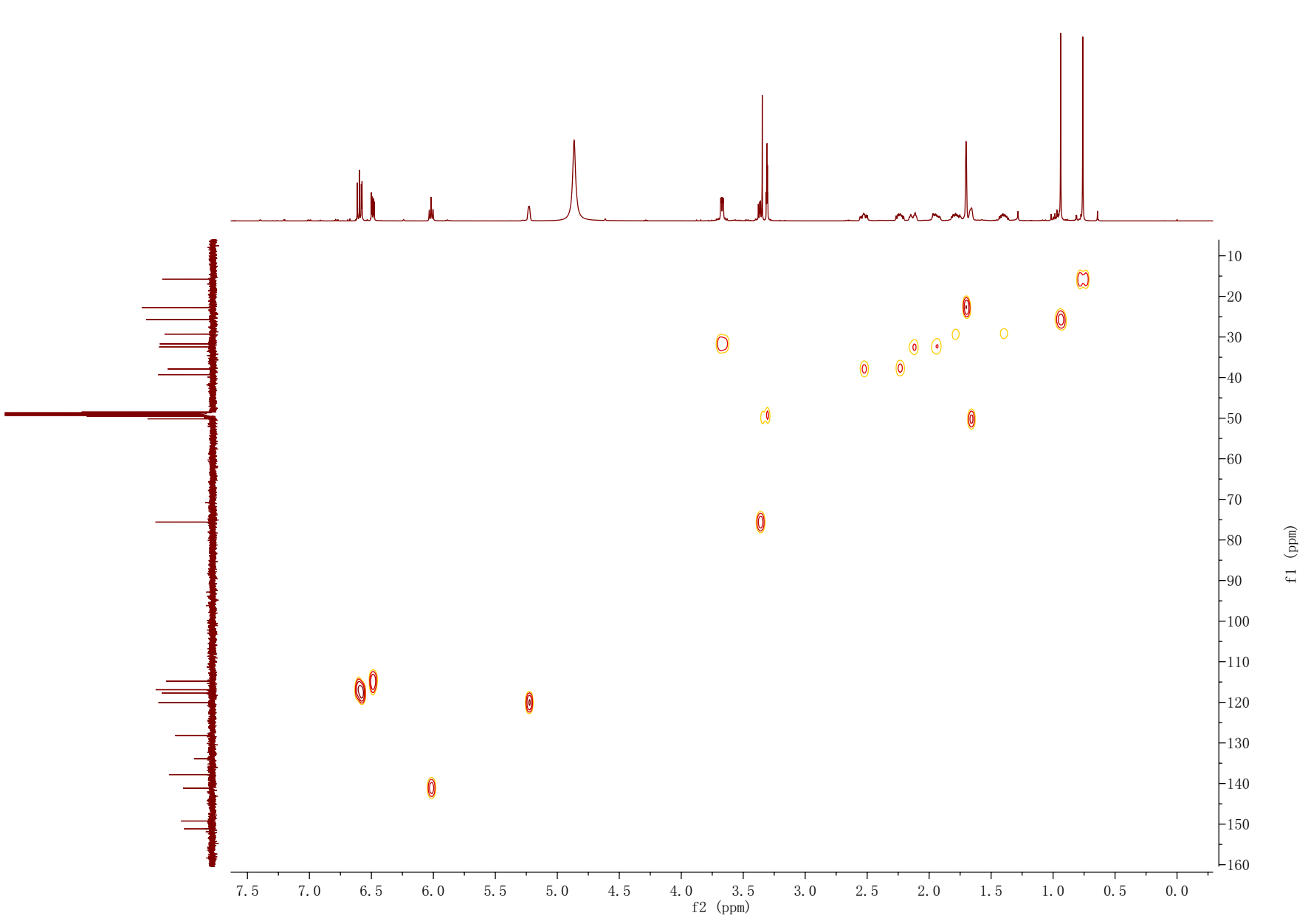


Figure S32. HSQC spectrum of **5** in methanol-*d*_4_.


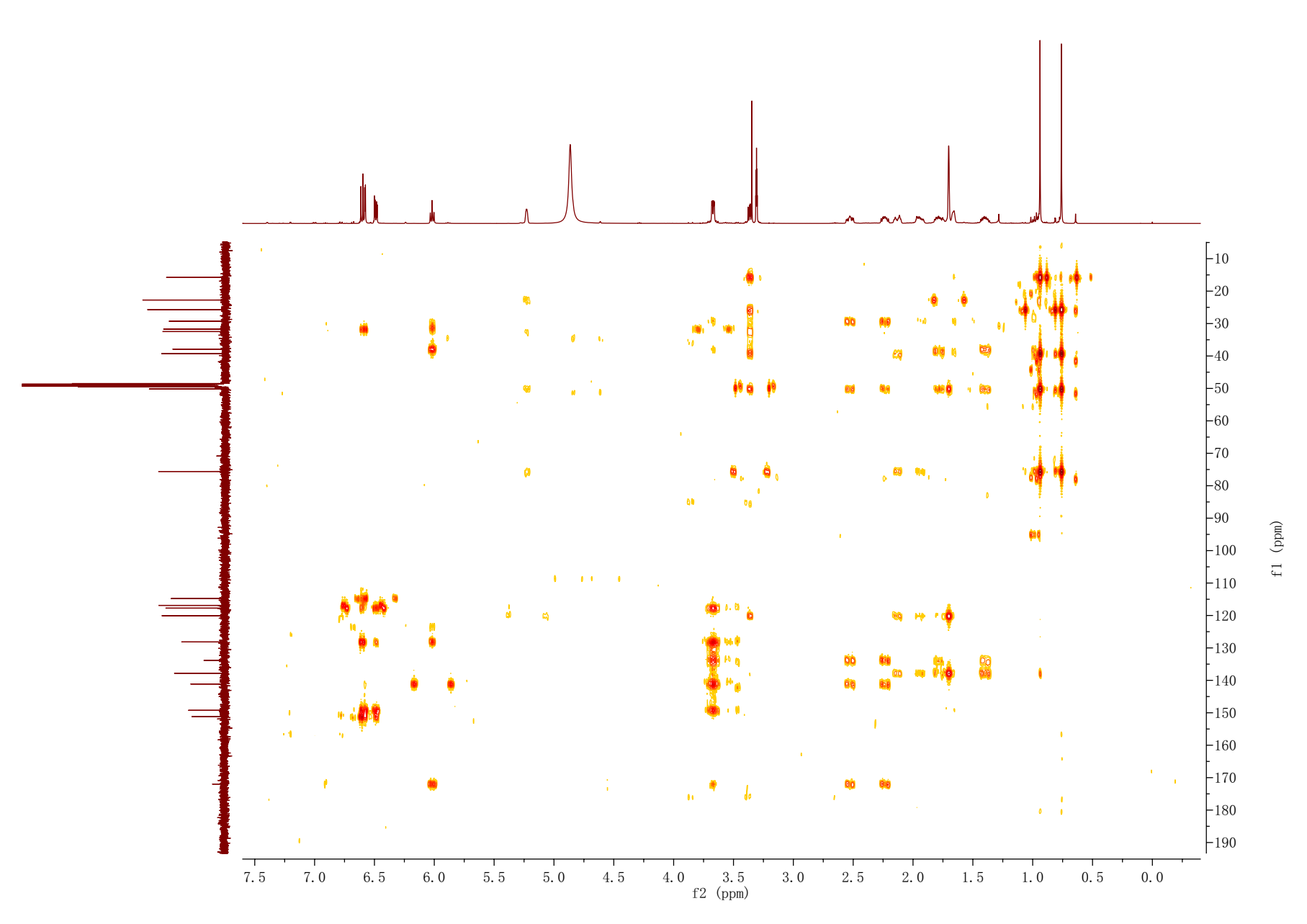


Figure S33. HMBC spectrum of **5** in methanol-*d*_4_.


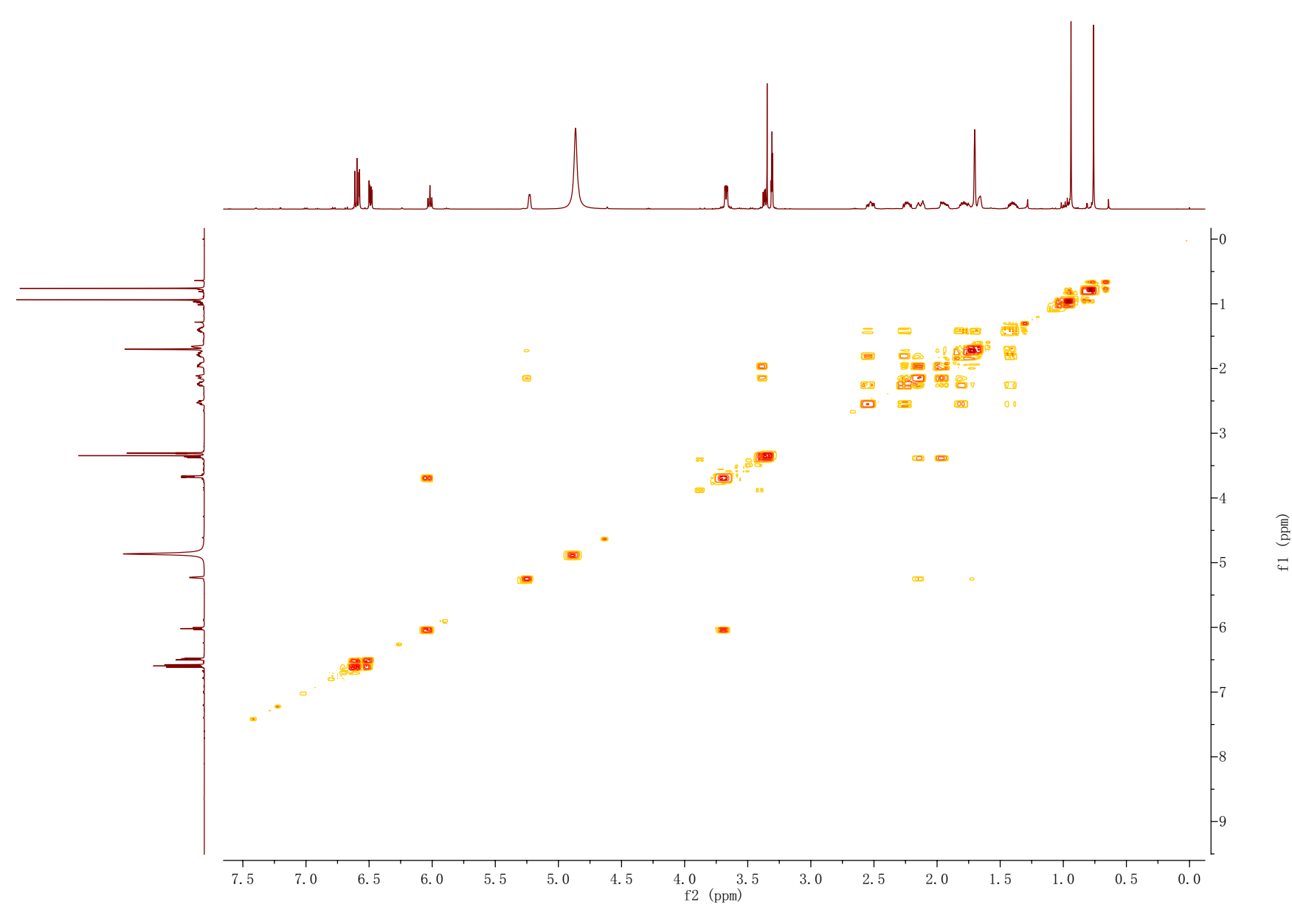


Figure S34. ^1^H-^1^H COSY spectrum of **5** in methanol-*d*_4_.


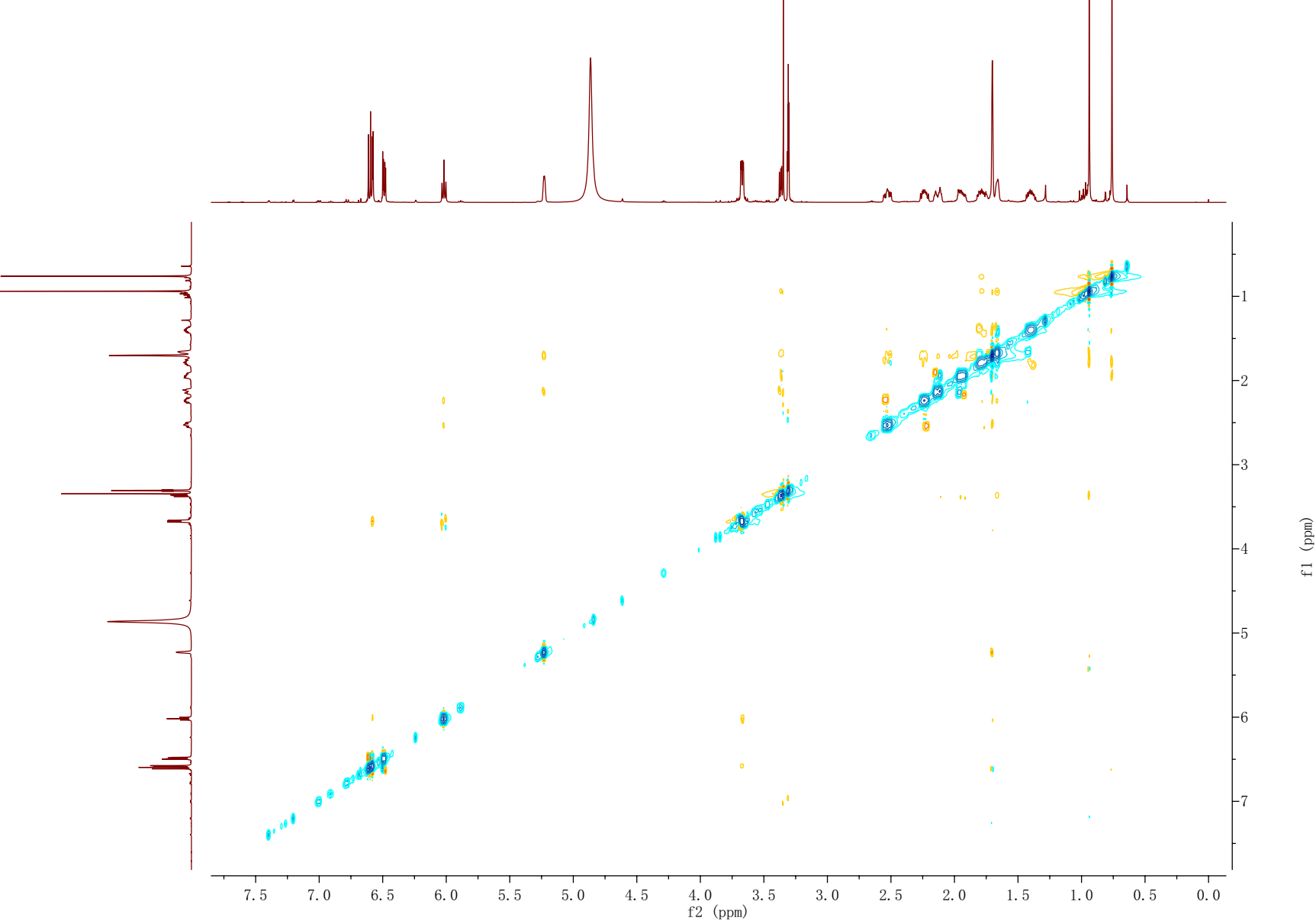


Figure S35. ROESY spectrum of **5** in methanol-*d*_4_.

[M+H]^+^ m/z 361.2008

| Hit | Formula | m/z | RDB | ppm |
| --- | --- | --- | --- | --- |
| 1 | C_21_H_29_O_5_ | 361.2010 | 8.0 | -0.4 |

Figure S36. HRESIMS of **5**.


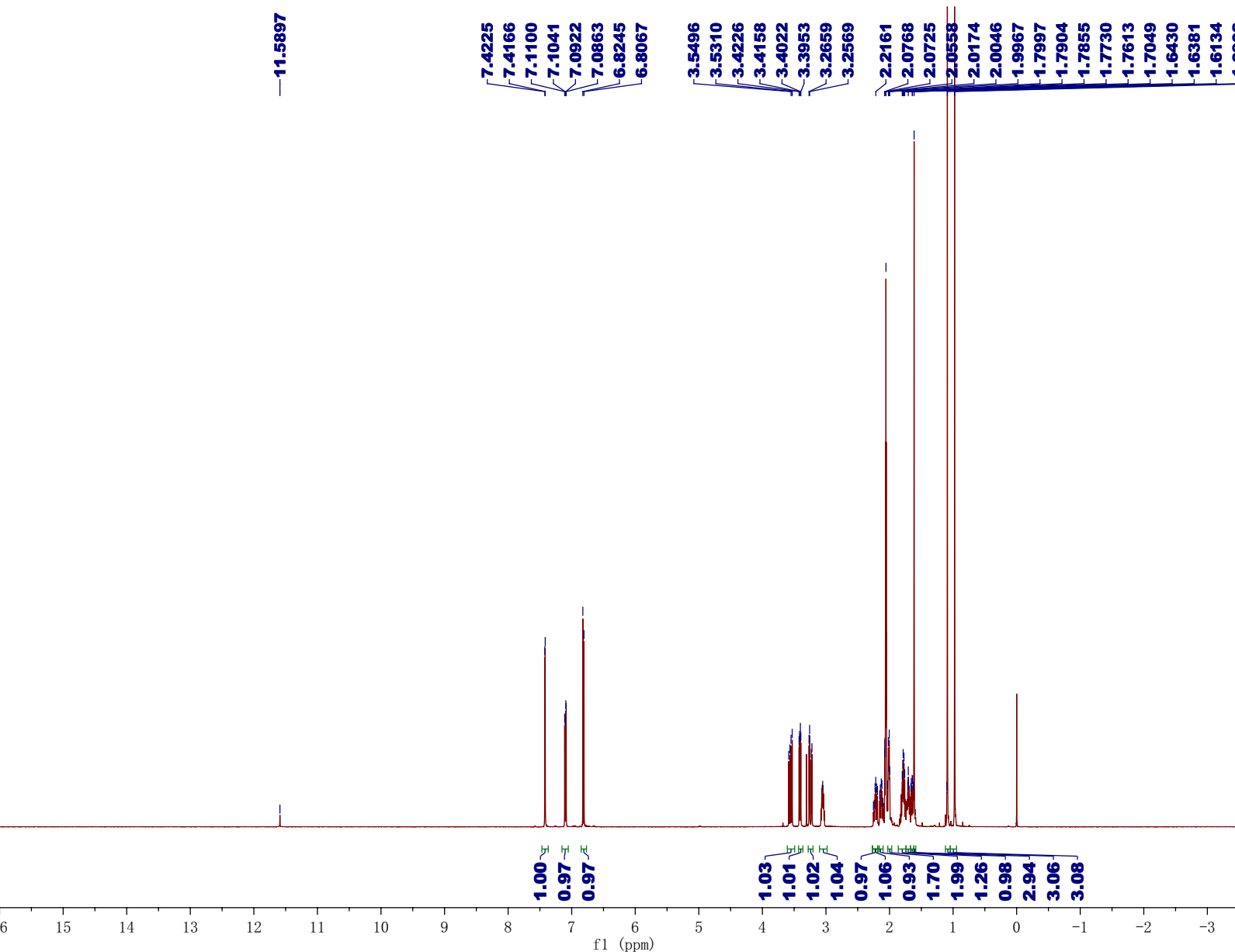


Figure S37. ^1^H NMR spectrum of **7** in acetone-*d*_6_.


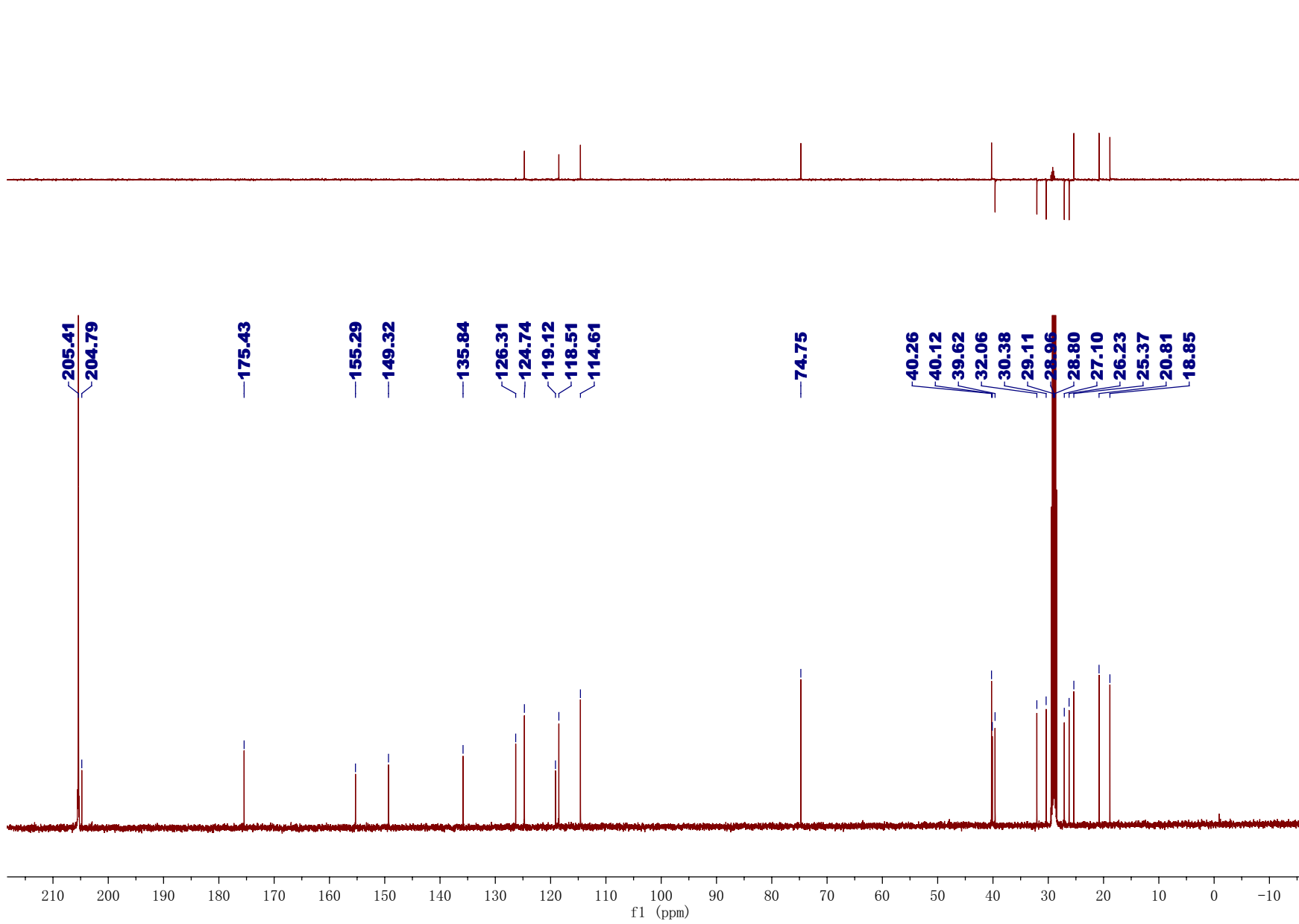


Figure S38. ^13^C NMR and DEPT spectra of **7** in acetone-*d*_6_.


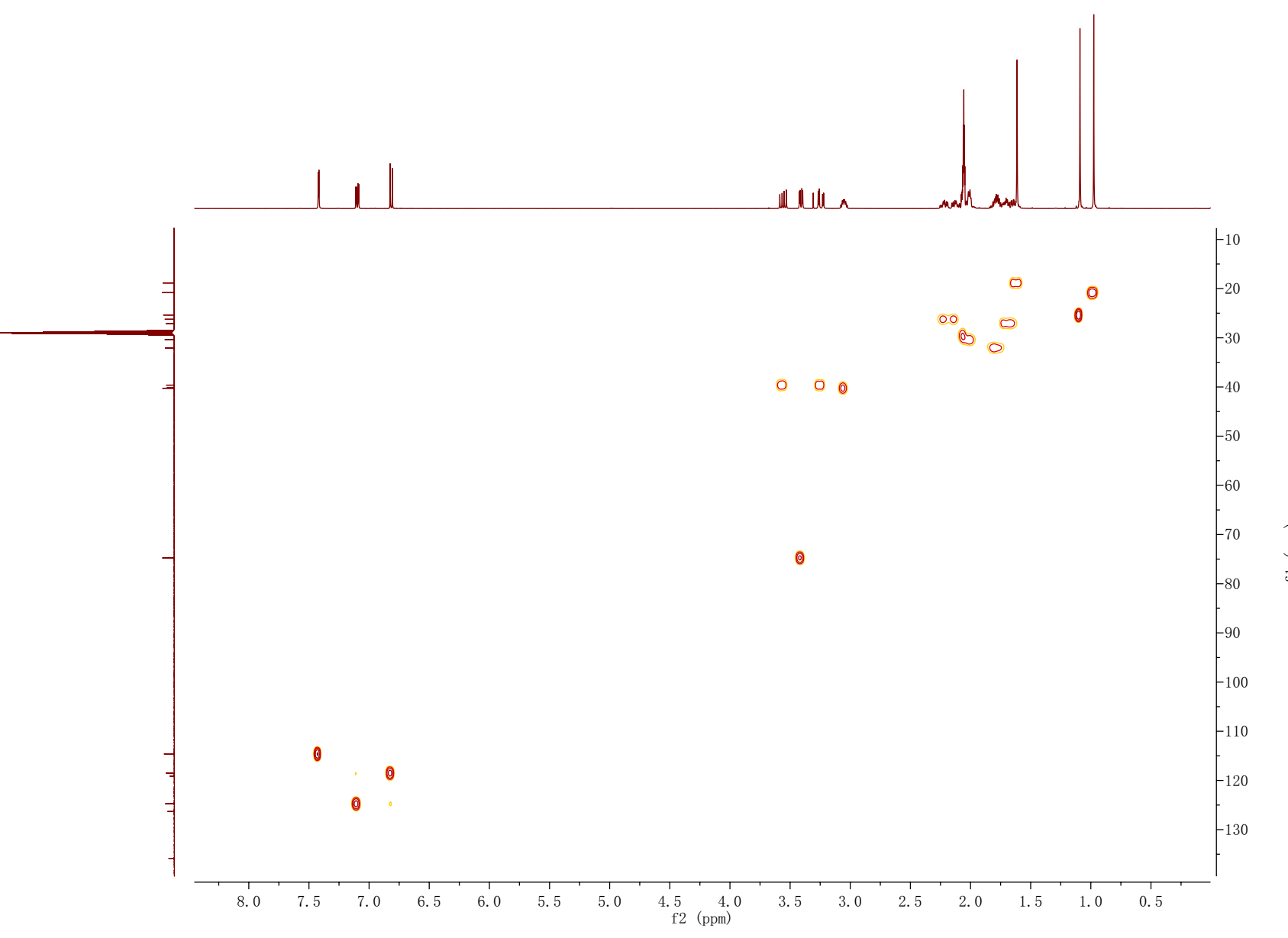


Figure S39. HSQC spectrum of **7** in acetone-*d*_6_.


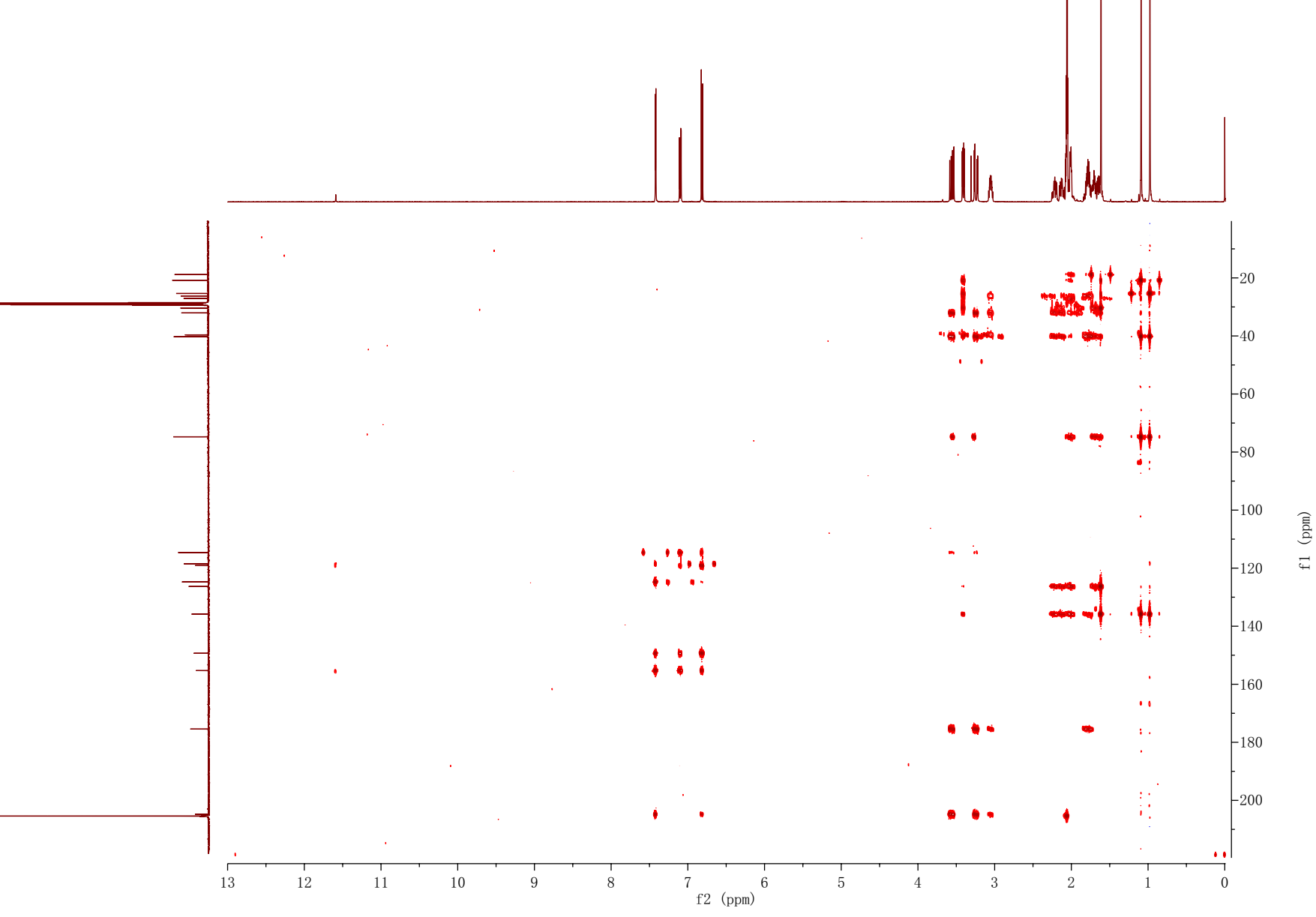


Figure S40. HMBC spectrum of **7** in acetone-*d*_6_.


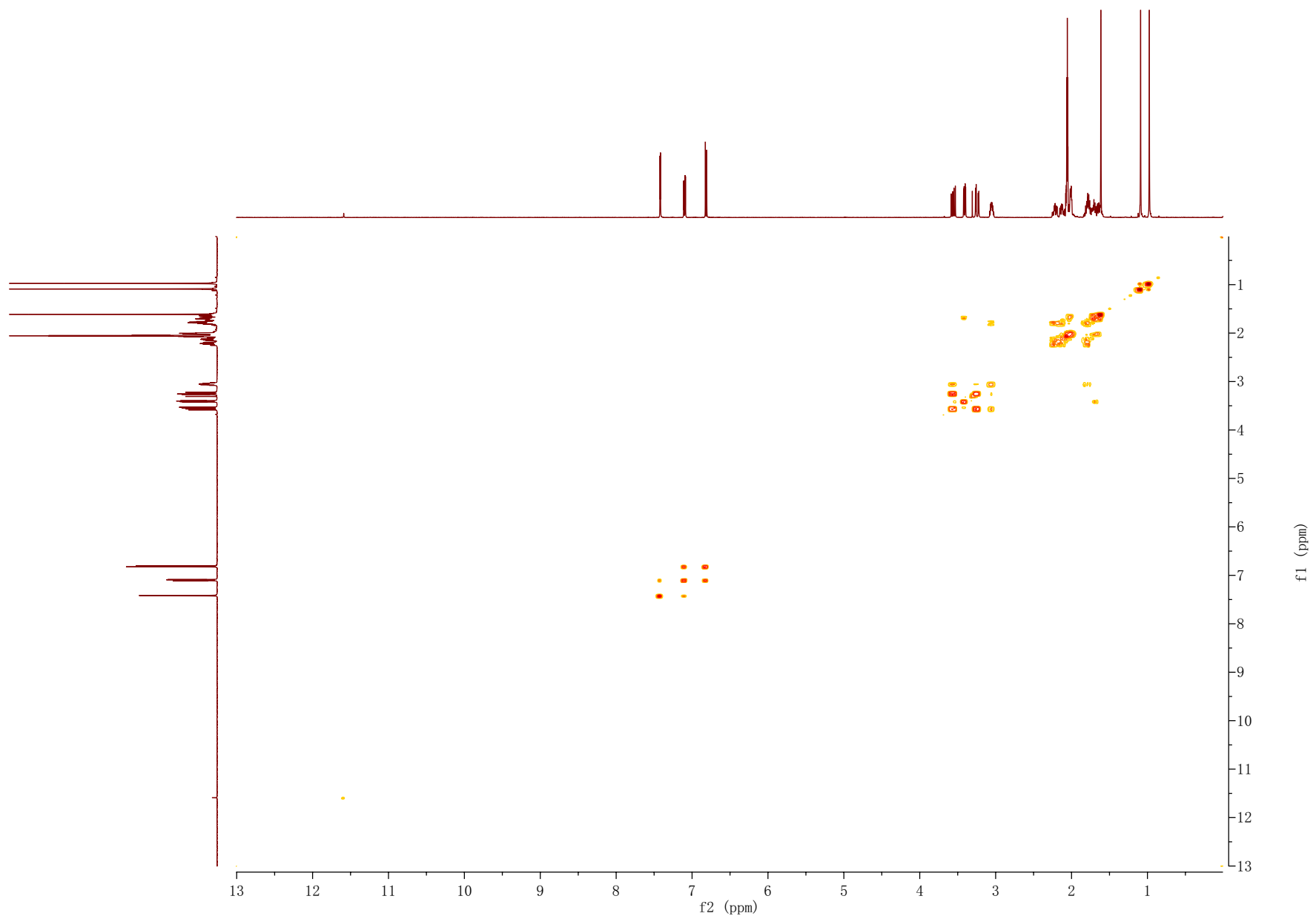


Figure S41. ^1^H-^1^H COSY spectrum of **7** in acetone-*d*_6_.


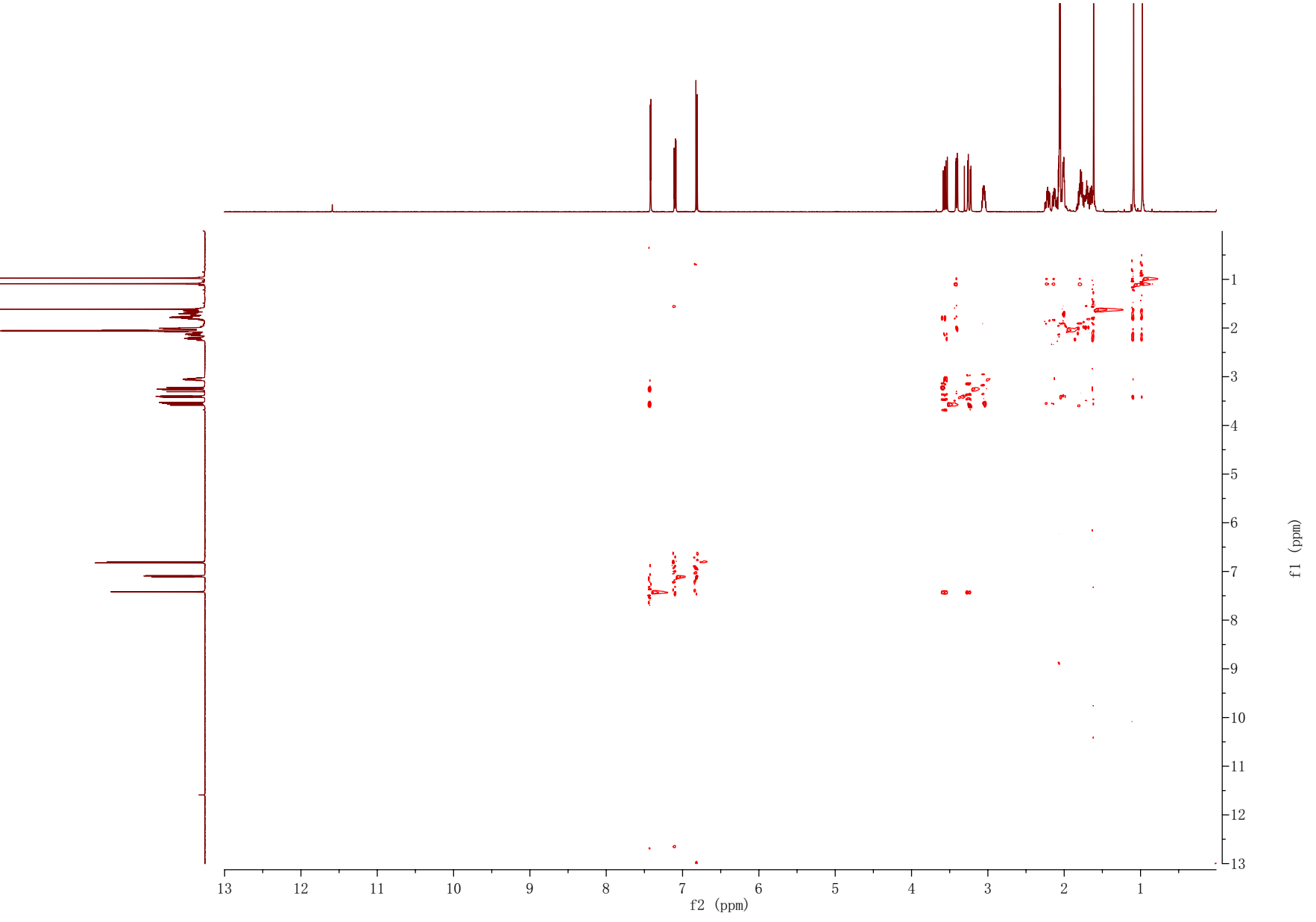


Figure S42. ROESY spectrum of **7** in acetone-*d*_6_.

[M+H]^+^ m/z 377.1964

| Hit | Formula | m/z | RDB | ppm |
| --- | --- | --- | --- | --- |
| 1 | C_21_H_29_O_6_ | 377.1959 | 8.0 | 1.4 |

Figure S43. HRESIMS of **7**.


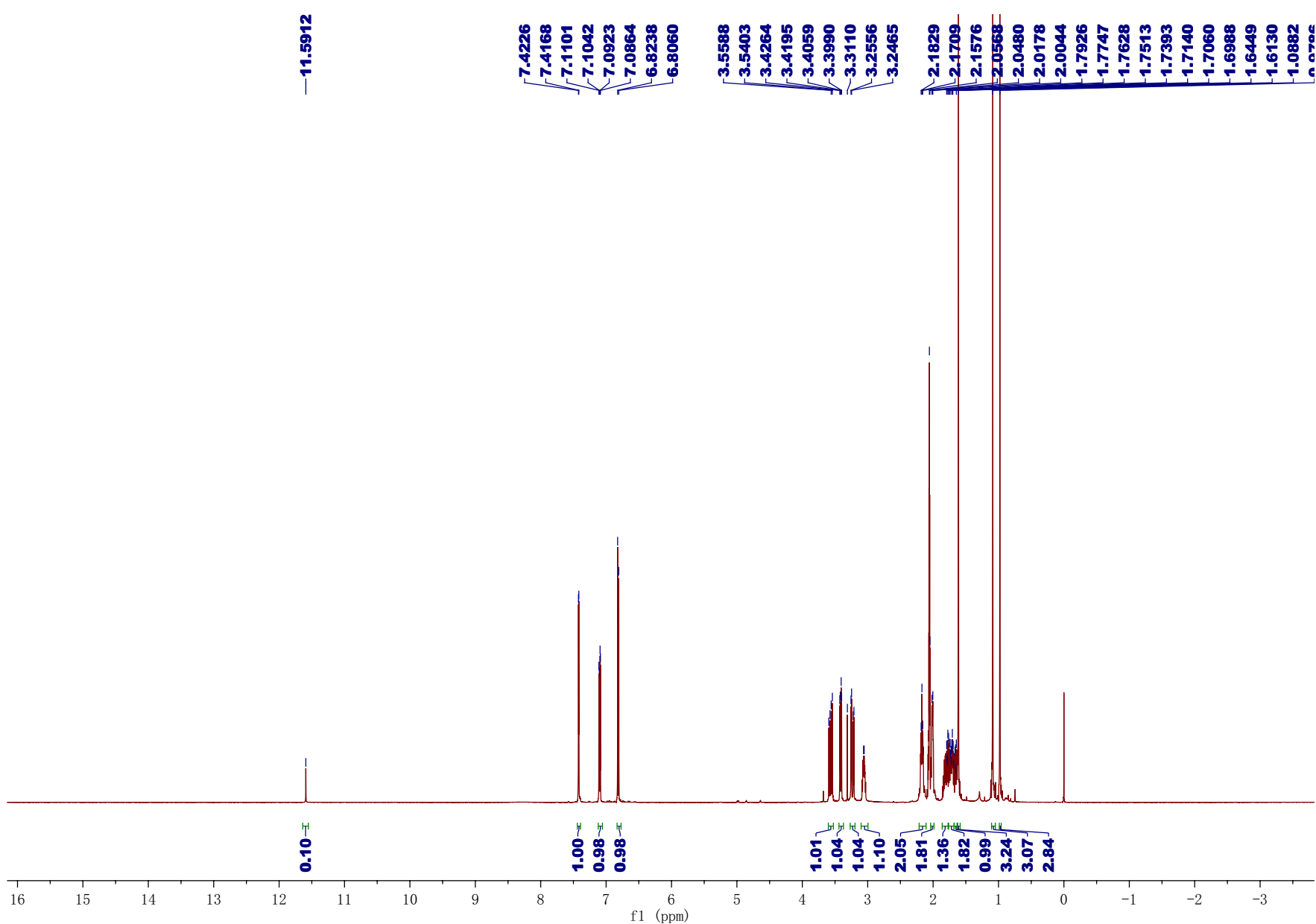


Figure S44. ^1^H NMR spectrum of **8** in acetone-*d*_6_.


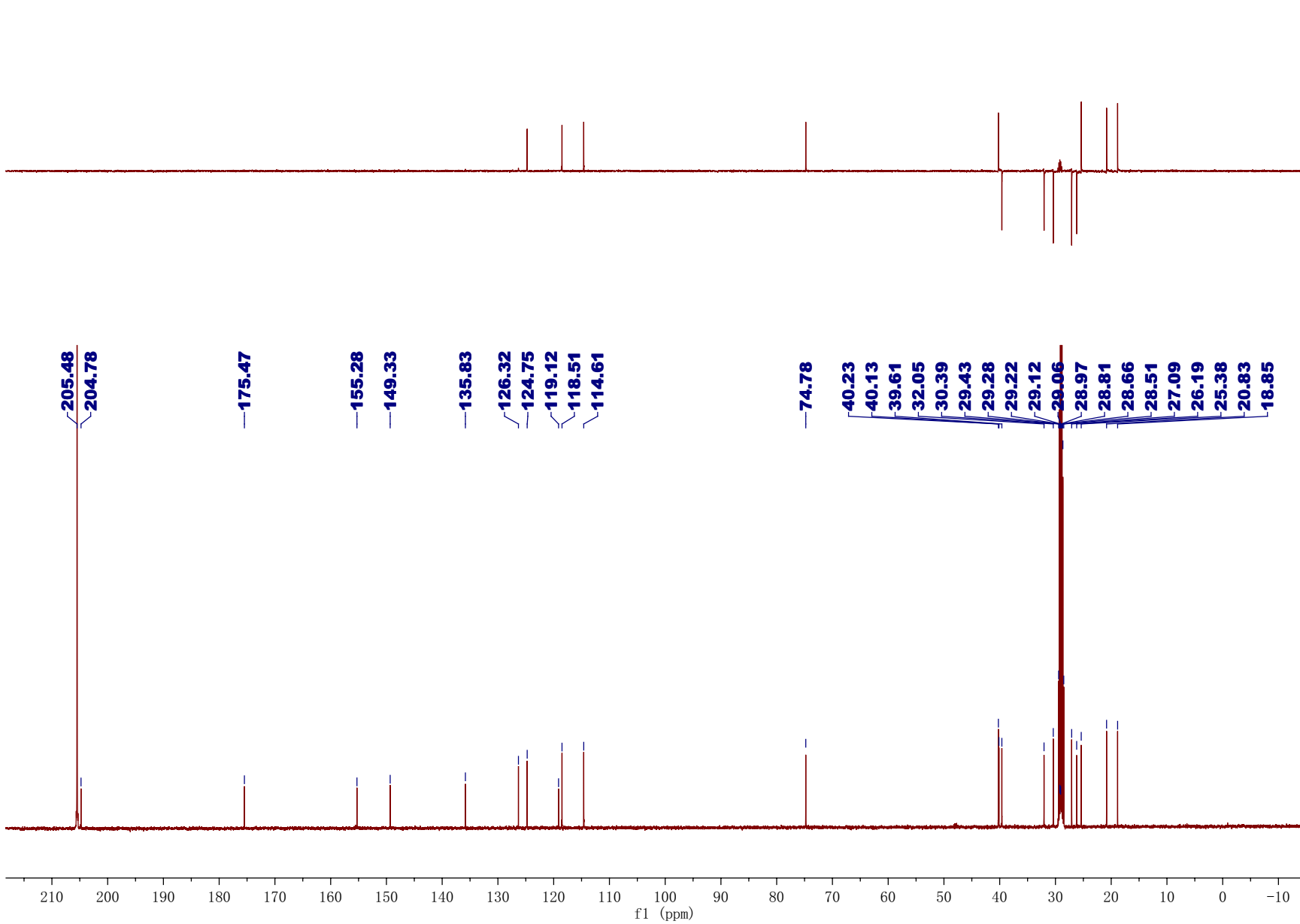


Figure S45. ^13^C NMR and DEPT spectra of **8** in acetone-*d*_6_.


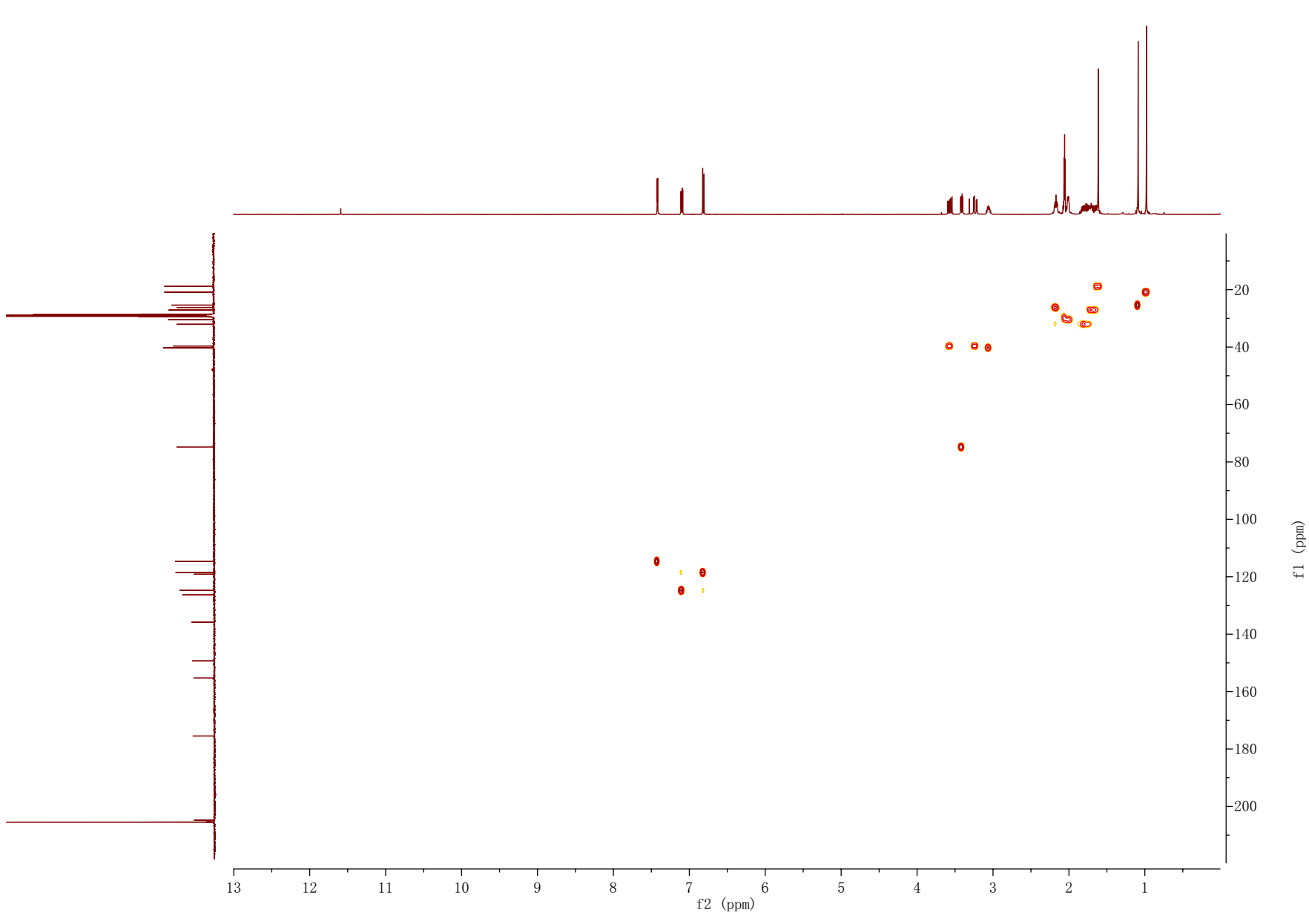


Figure S46. HSQC spectrum of **8** in acetone-*d*_6_.


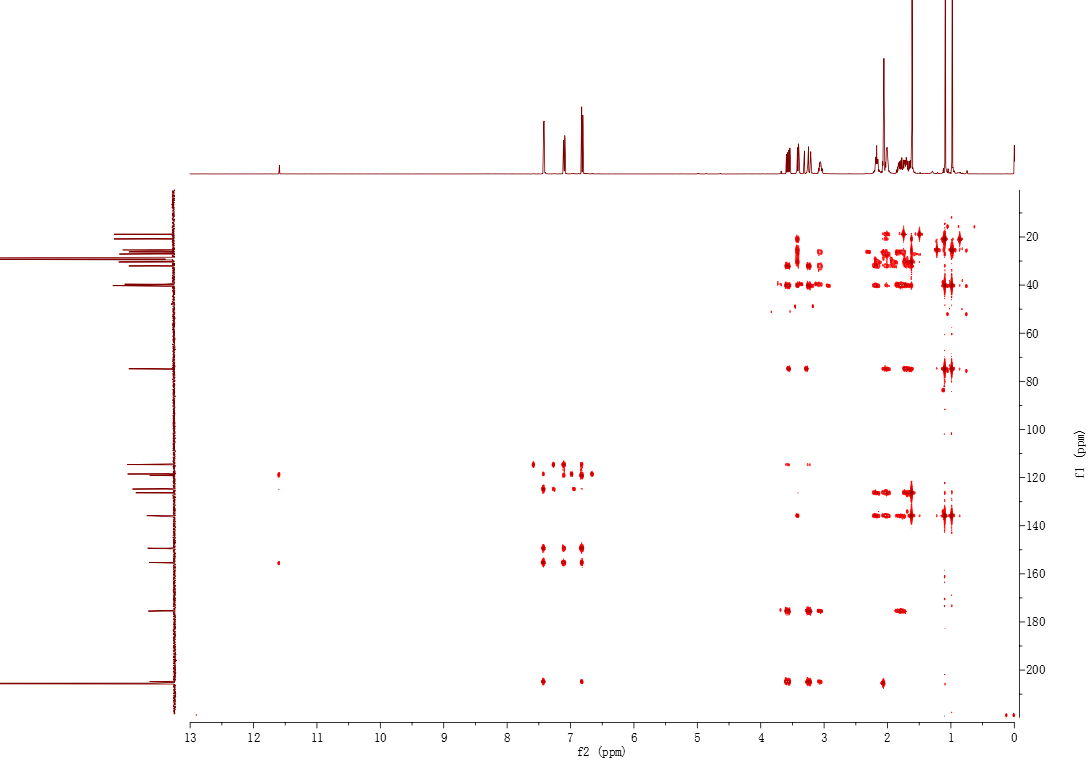


Figure S47. HMBC spectrum of **8** in acetone-*d*_6_.


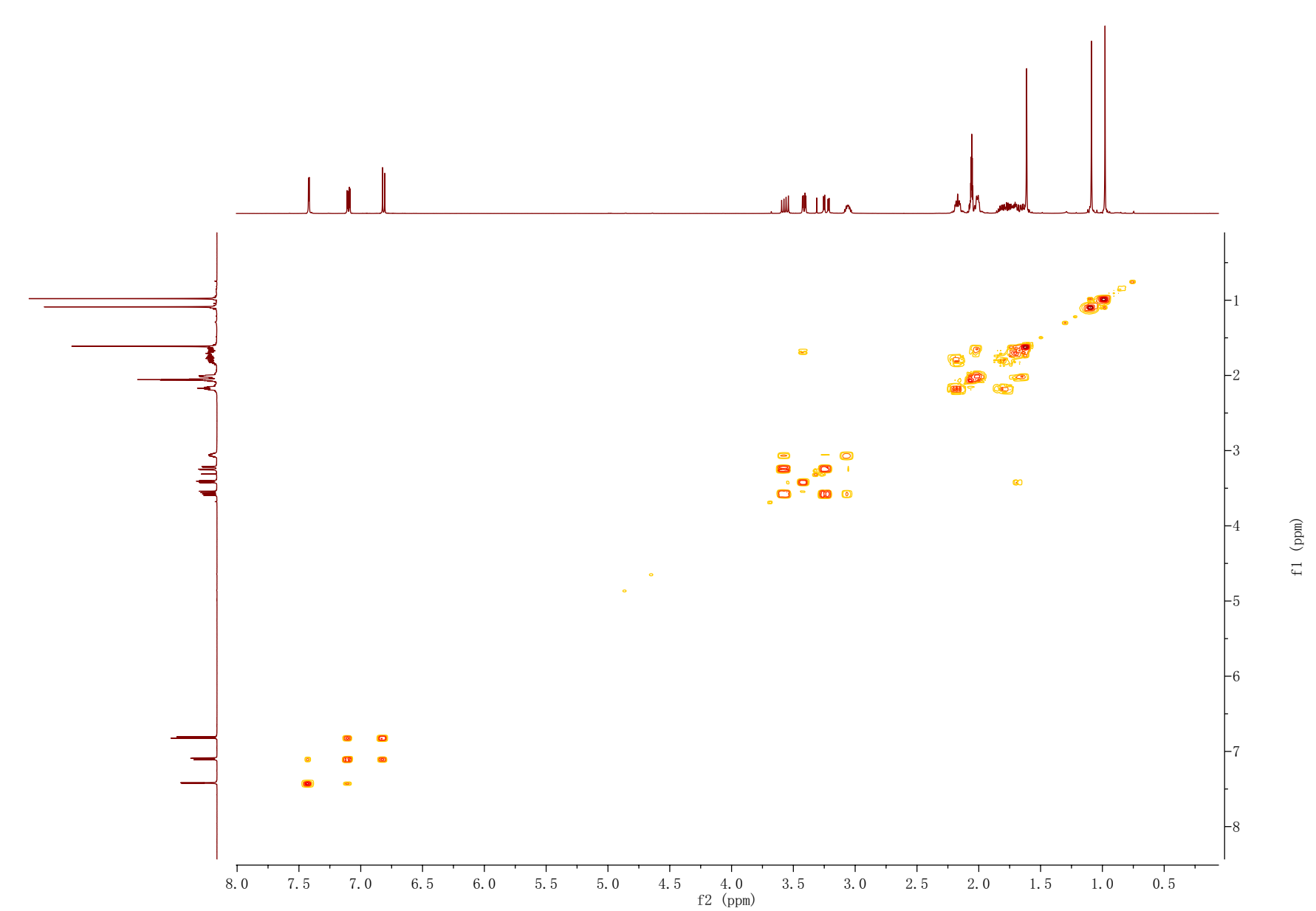


Figure S48. ^1^H-^1^H COSY spectrum of **8** in acetone-*d*_6_.


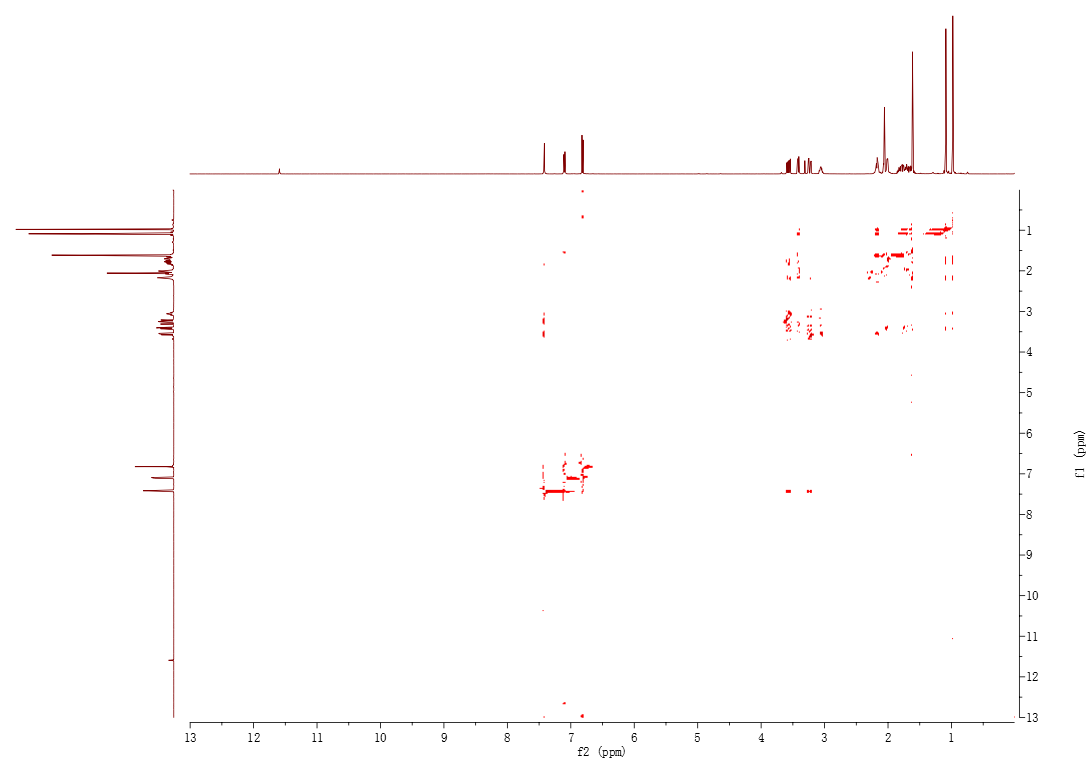


Figure S49. ROESY spectrum of **8** in acetone-*d*_6_.

[M+H]^+^ m/z 377.1964

| Hit | Formula | m/z | RDB | ppm |
| --- | --- | --- | --- | --- |
| 1 | C_21_H_29_O_6_ | 377.1959 | 8.0 | 1.4 |

Figure S50. HRESIMS of **8**.


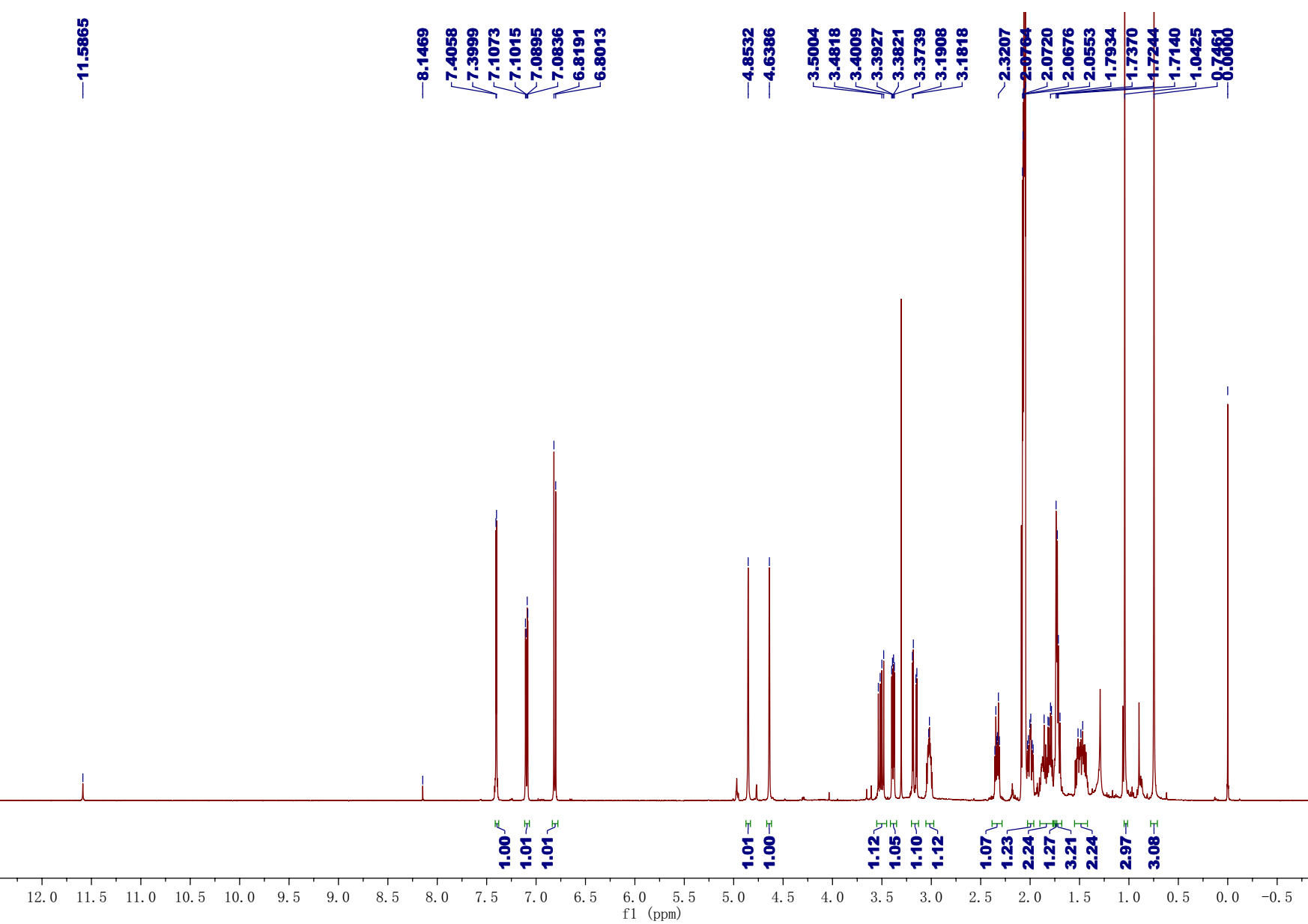


Figure S51. ^1^H NMR spectrum of **9** in acetone-*d*_6_.


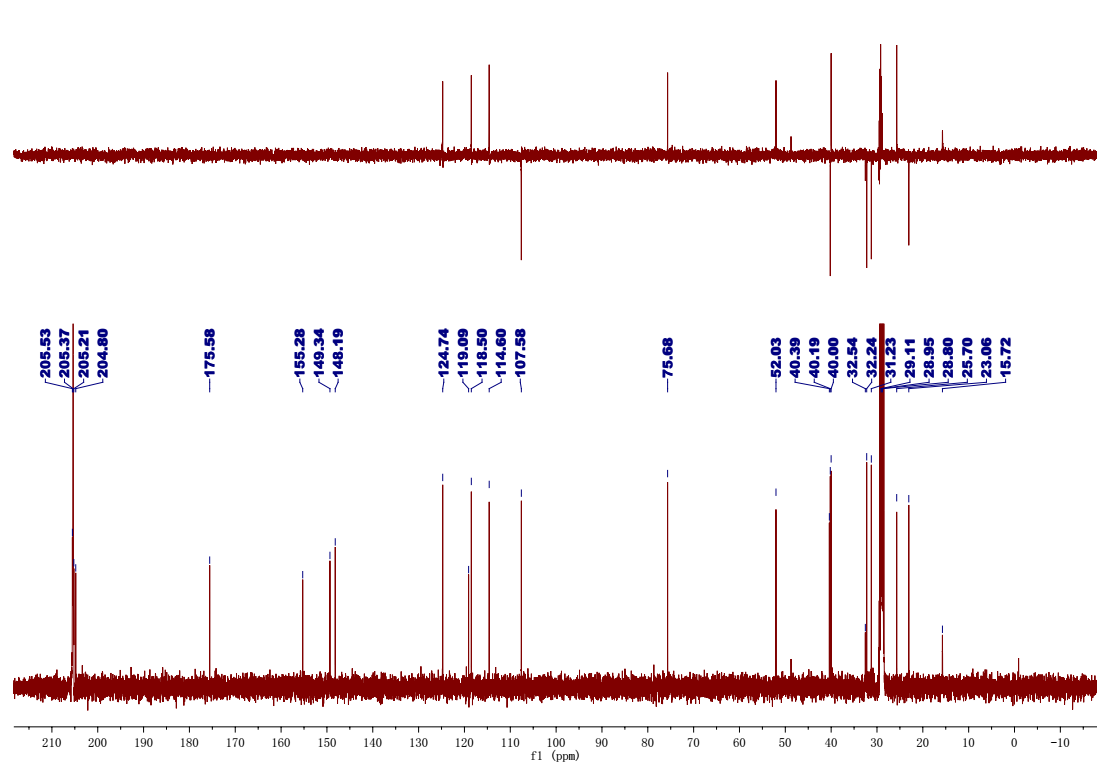


Figure S52. ^13^C NMR and DEPT spectra of **9** in acetone-*d*_6_.


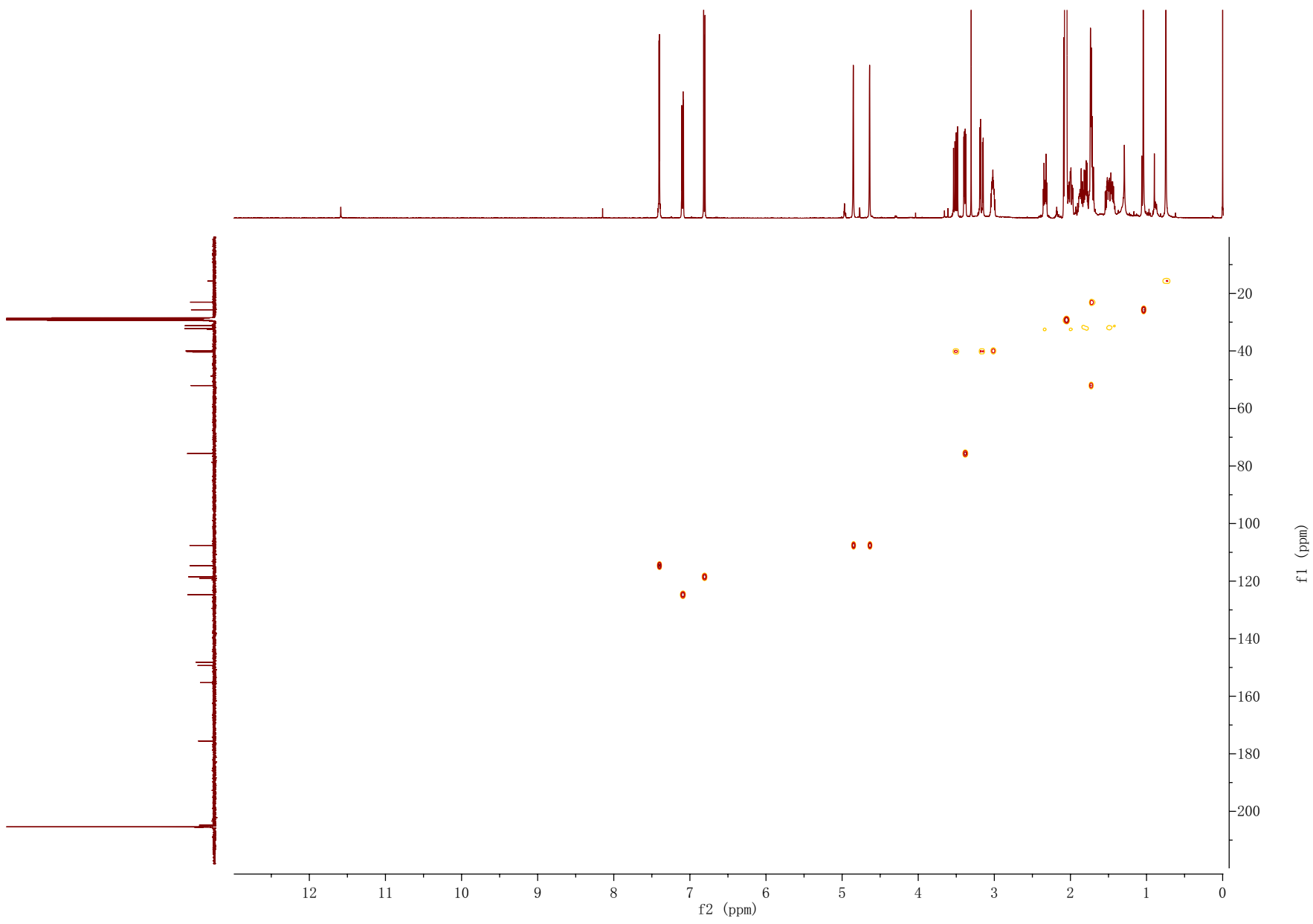


Figure S53. HSQC spectrum of **9** in acetone-*d*_6_.


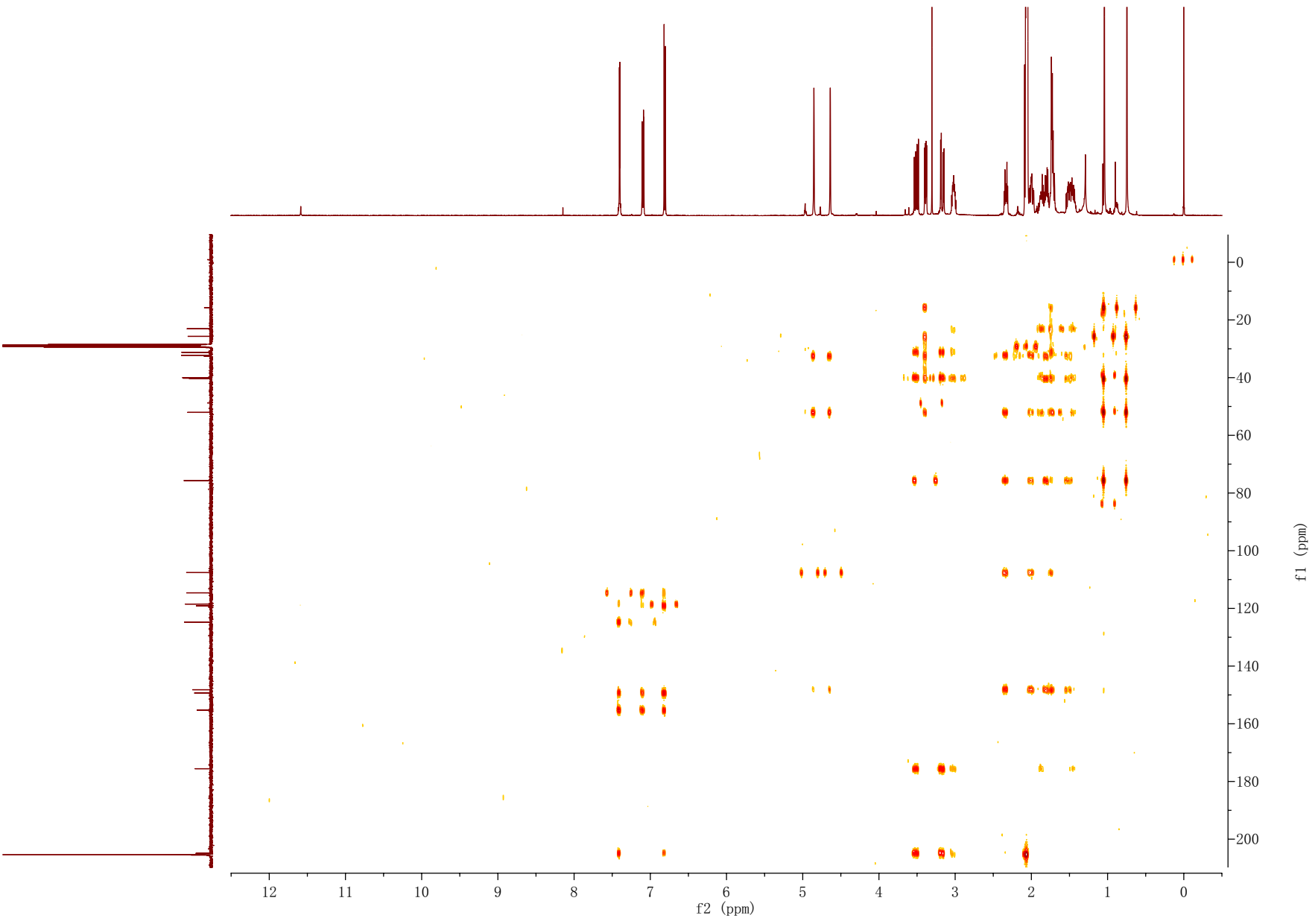


Figure S54. HMBC spectrum of **9** in acetone-*d*_6_.


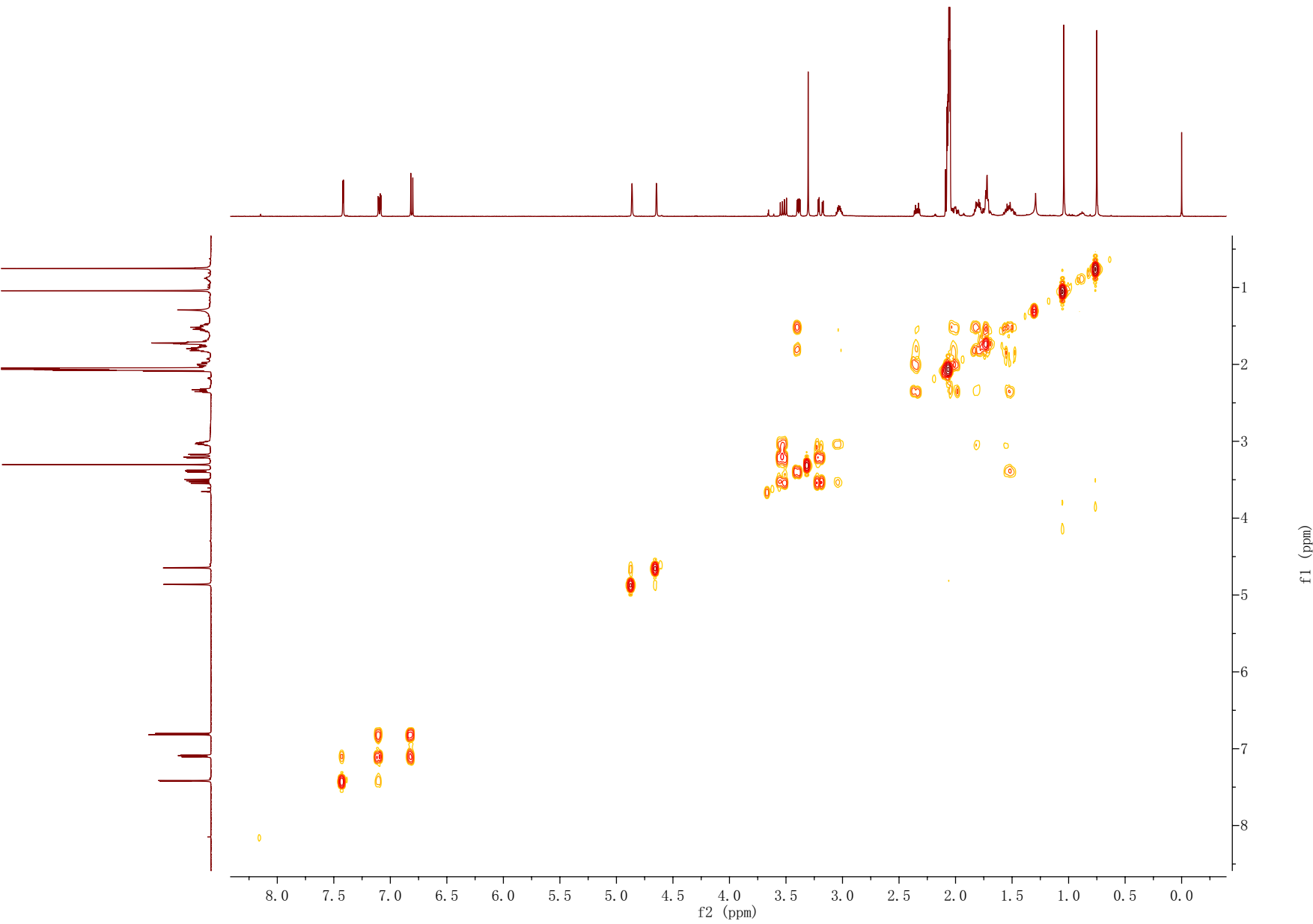


Figure S55. ^1^H-^1^H COSY spectrum of **9** in acetone-*d*_6_.


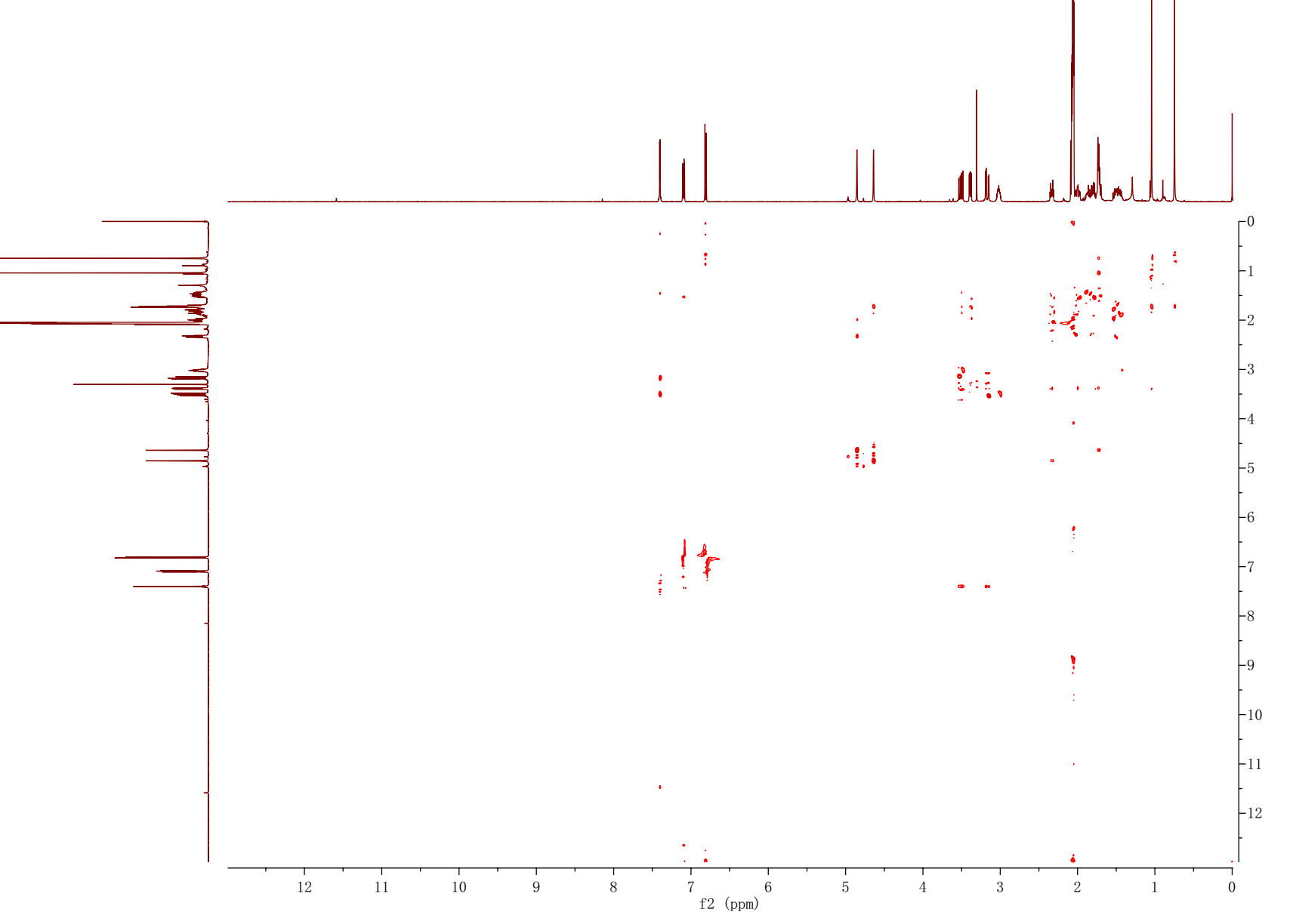


Figure S56. ROESY spectrum of **9** in acetone-*d*_6_.

[M+H]^+^ m/z 377.1964

| Hit | Formula | m/z | RDB | ppm |
| --- | --- | --- | --- | --- |
| 1 | C_21_H_29_O_6_ | 377.1959 | 8.0 | 1.4 |

Figure S57. HRESIMS of **9**.


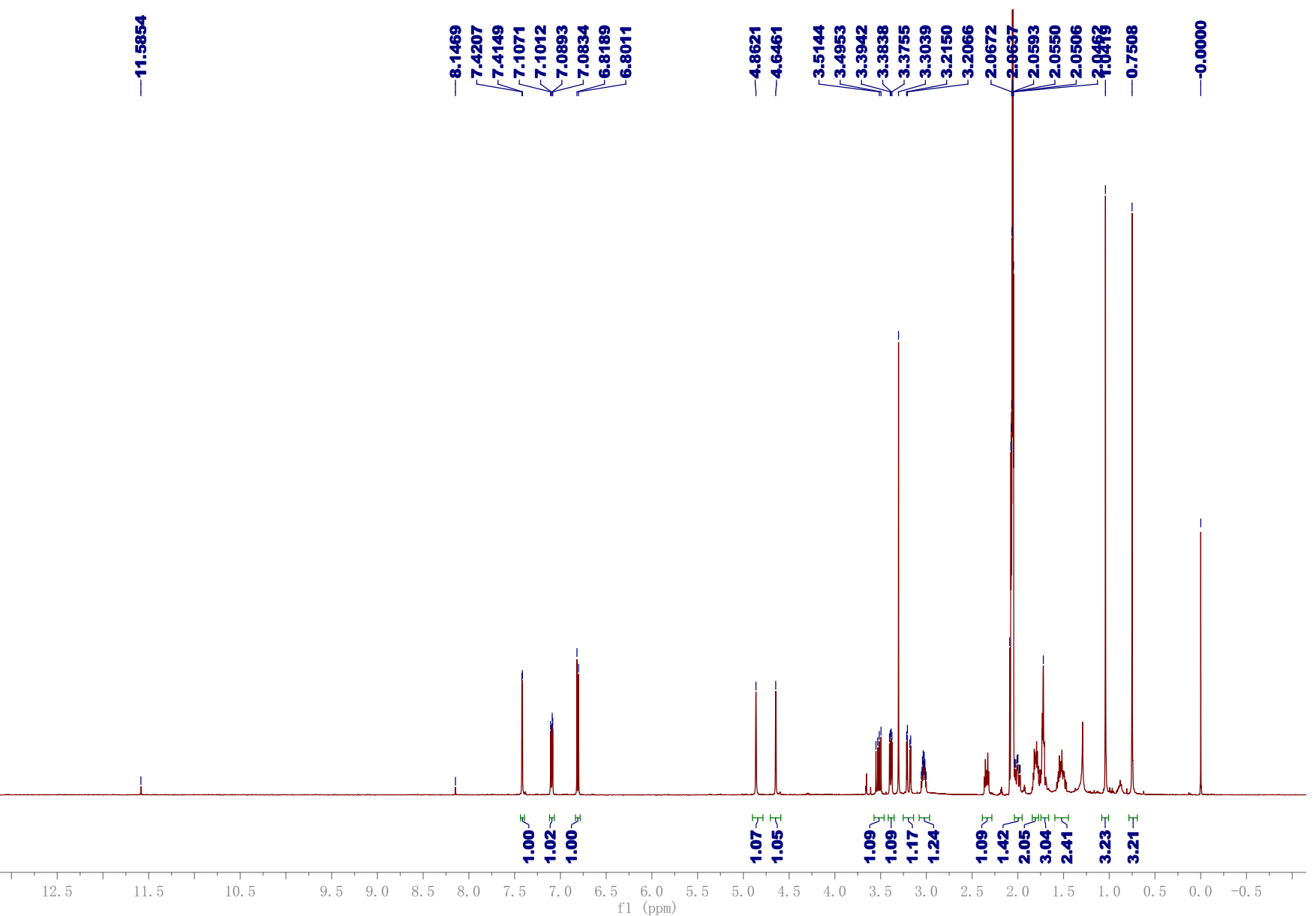


Figure S58. ^1^H NMR spectrum of **10** in acetone-*d*_6_.


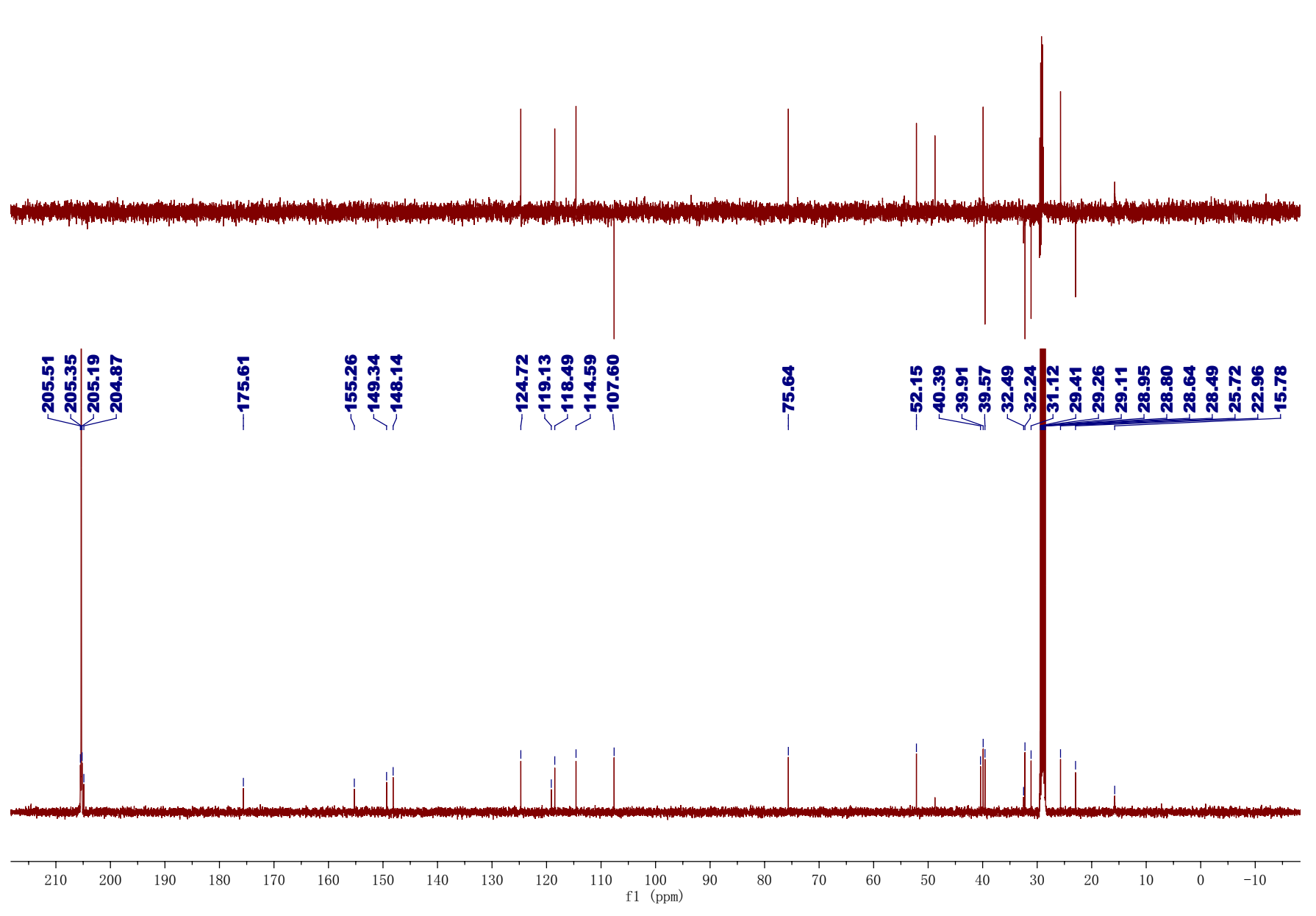


Figure S59. ^13^C NMR and DEPT spectra of **10** in acetone-*d*_6_.


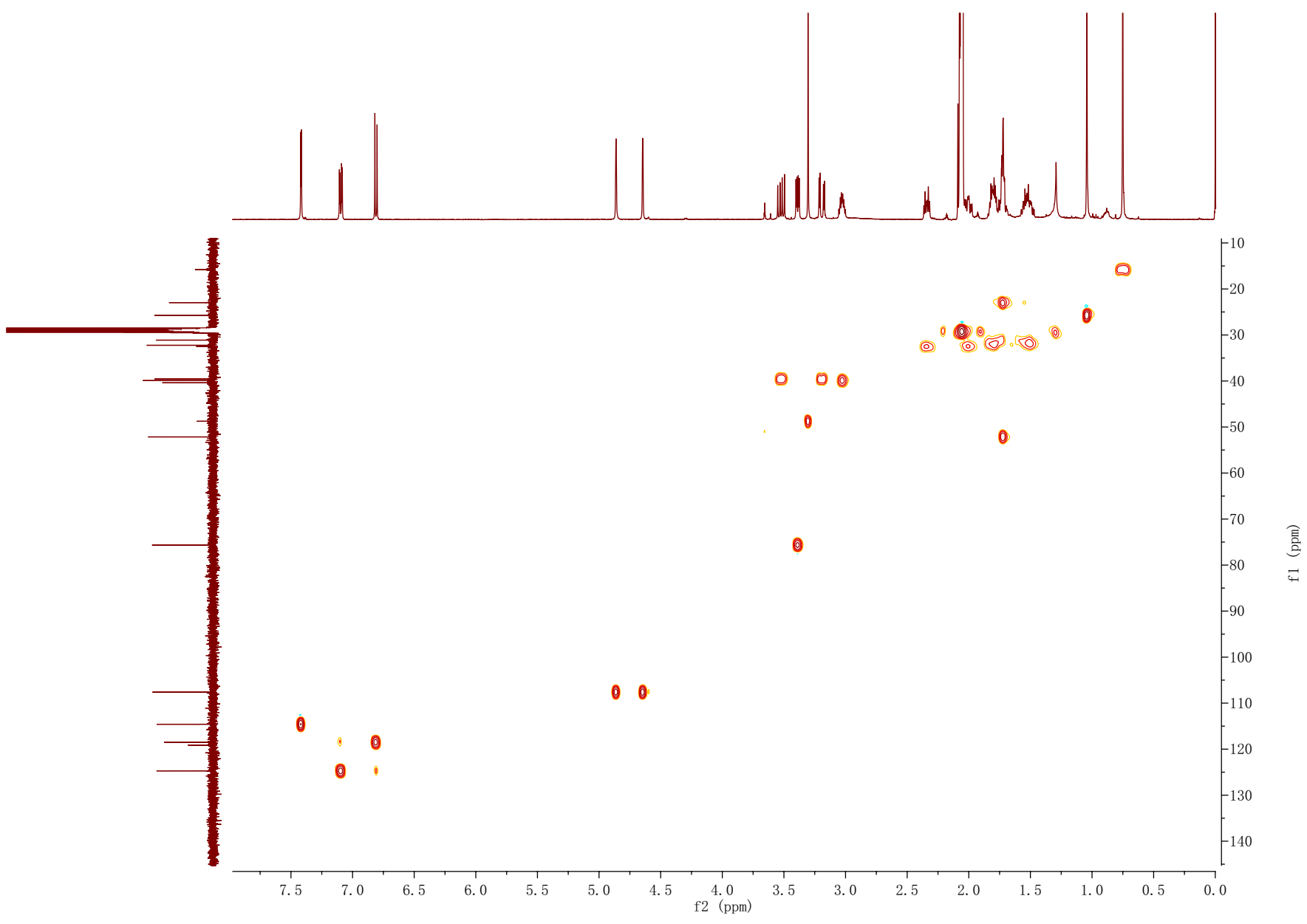


Figure S60. HSQC spectrum of **10** in acetone-*d*_6_.


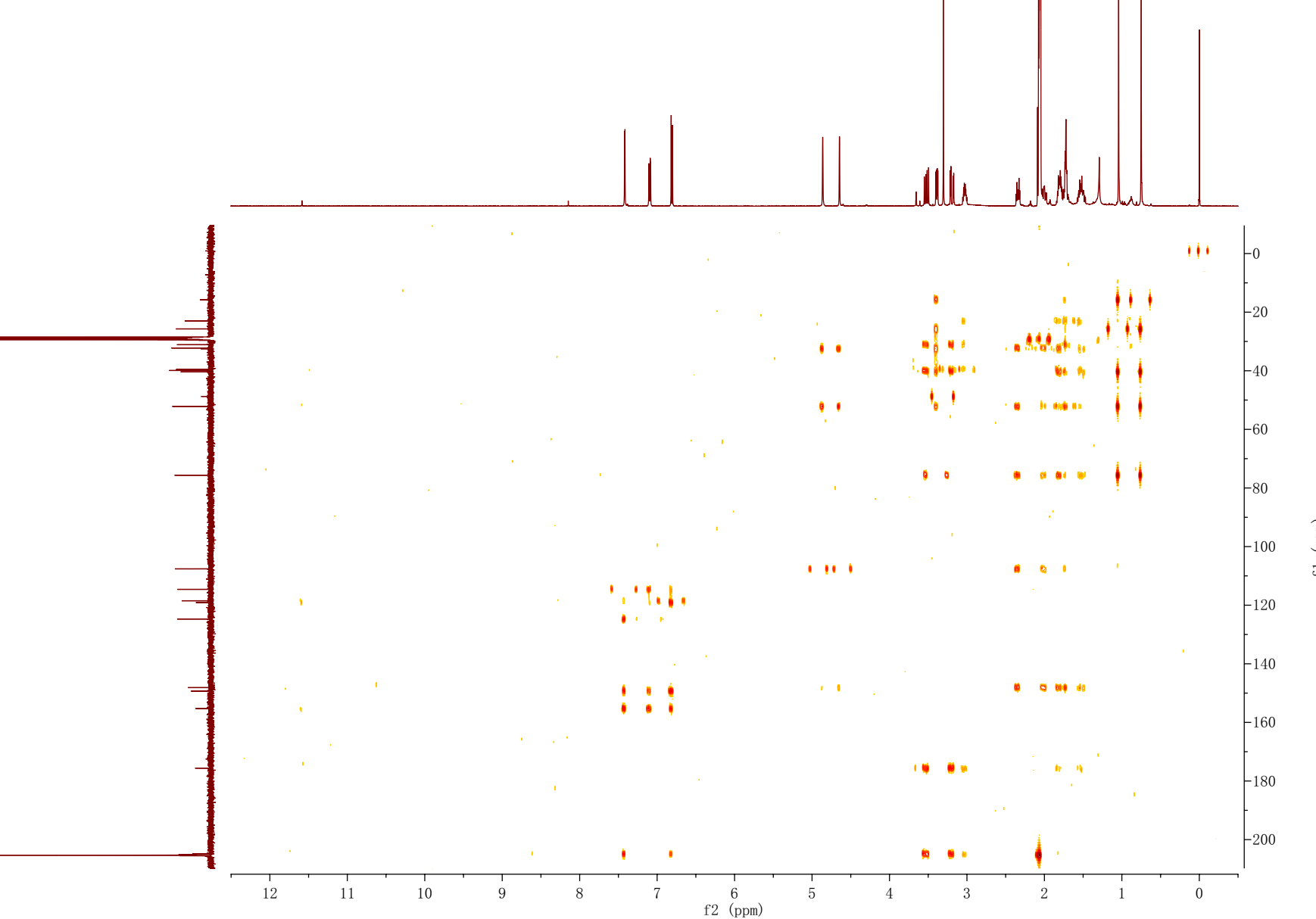


Figure S61. HMBC spectrum of **10** in acetone-*d*_6_.


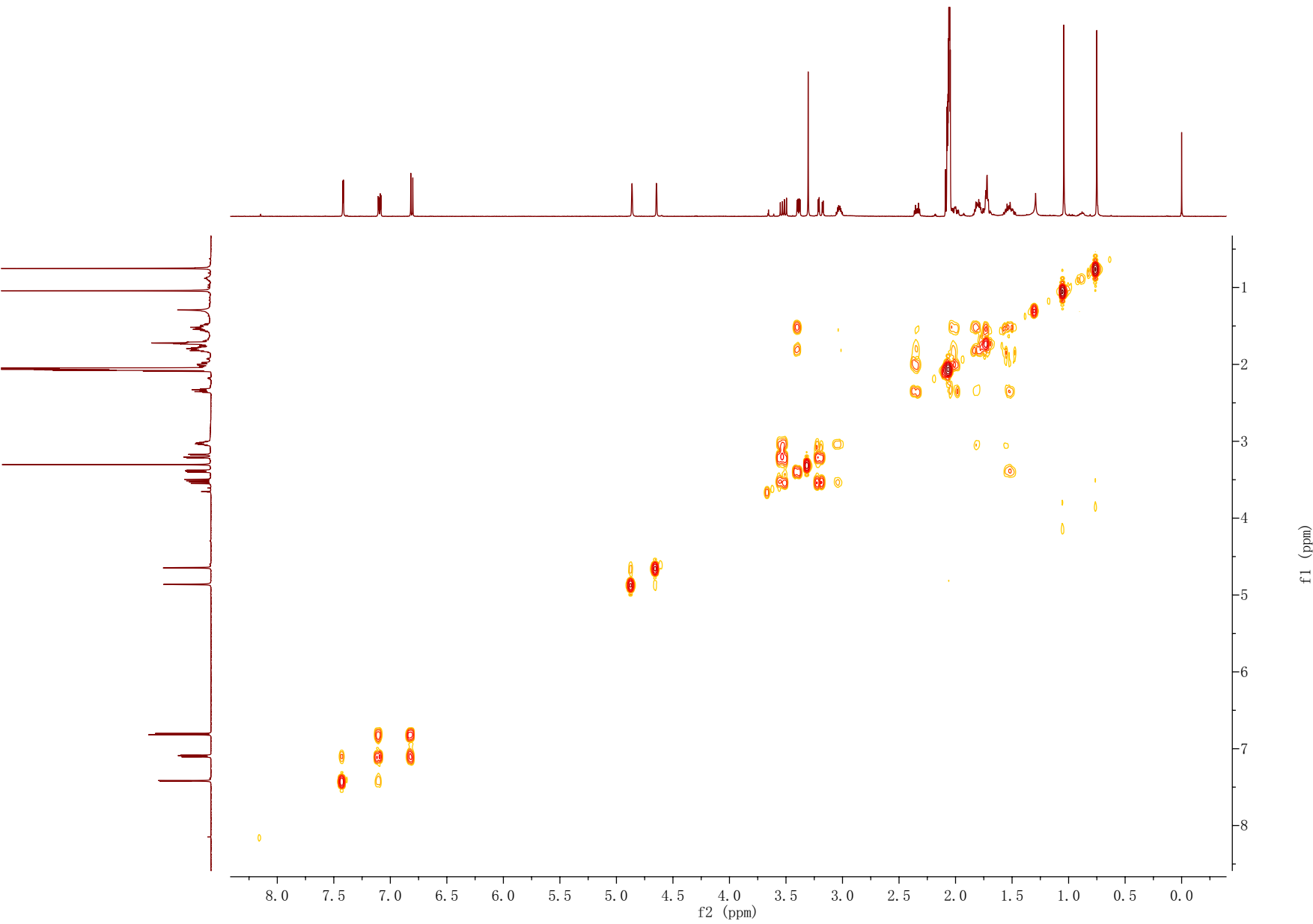


Figure S62. ^1^H-^1^H COSY spectrum of **10** in acetone-*d*_6_.


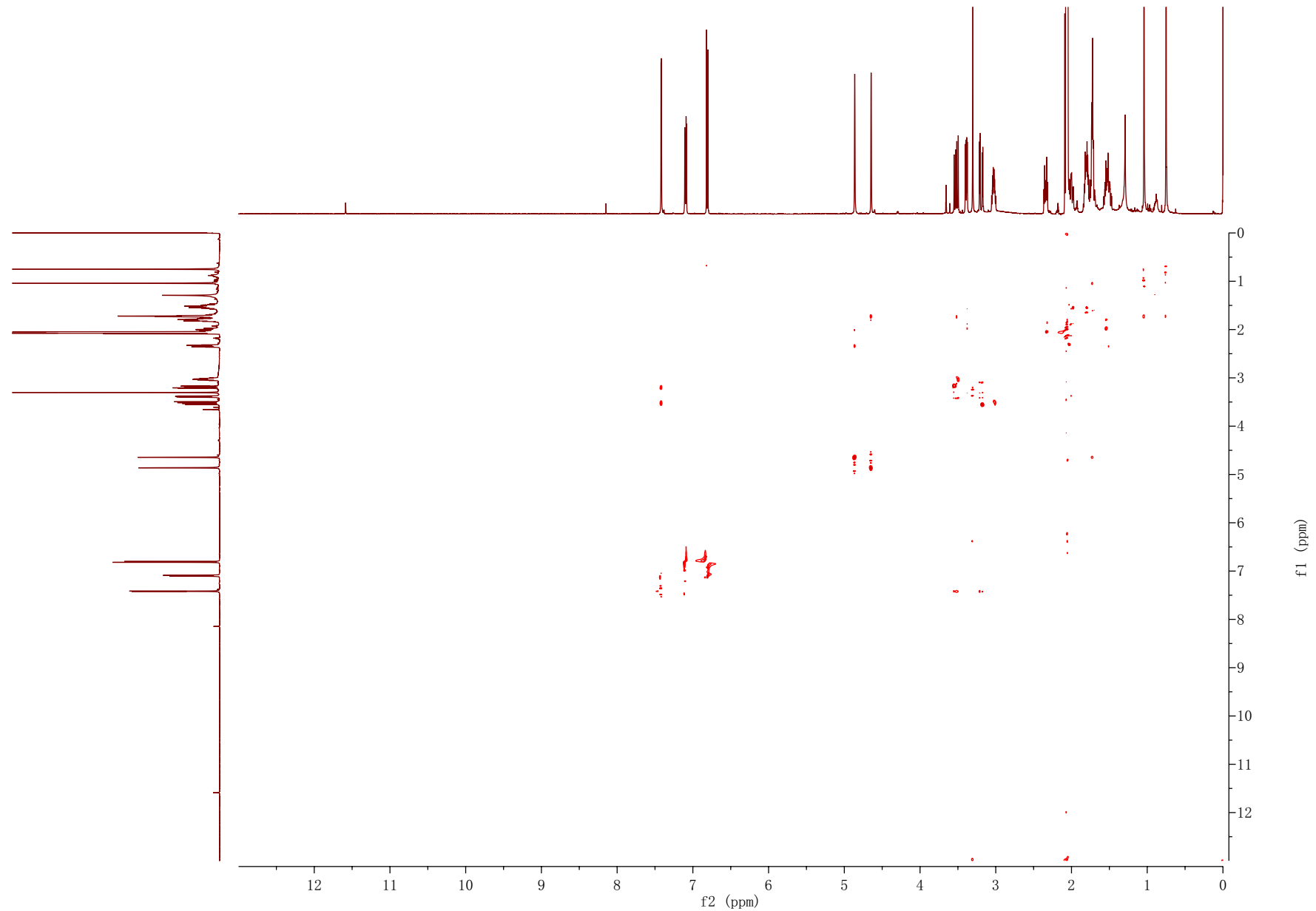


Figure S63. ROESY spectrum of **10** in acetone-*d*_6_.

[M+H]^+^ m/z 377.1961

| Hit | Formula | m/z | RDB | ppm |
| --- | --- | --- | --- | --- |
| 1 | C_21_H_29_O_6_ | 377.1959 | 8.0 | 0.6 |

Figure S64. HRESIMS of **10**.


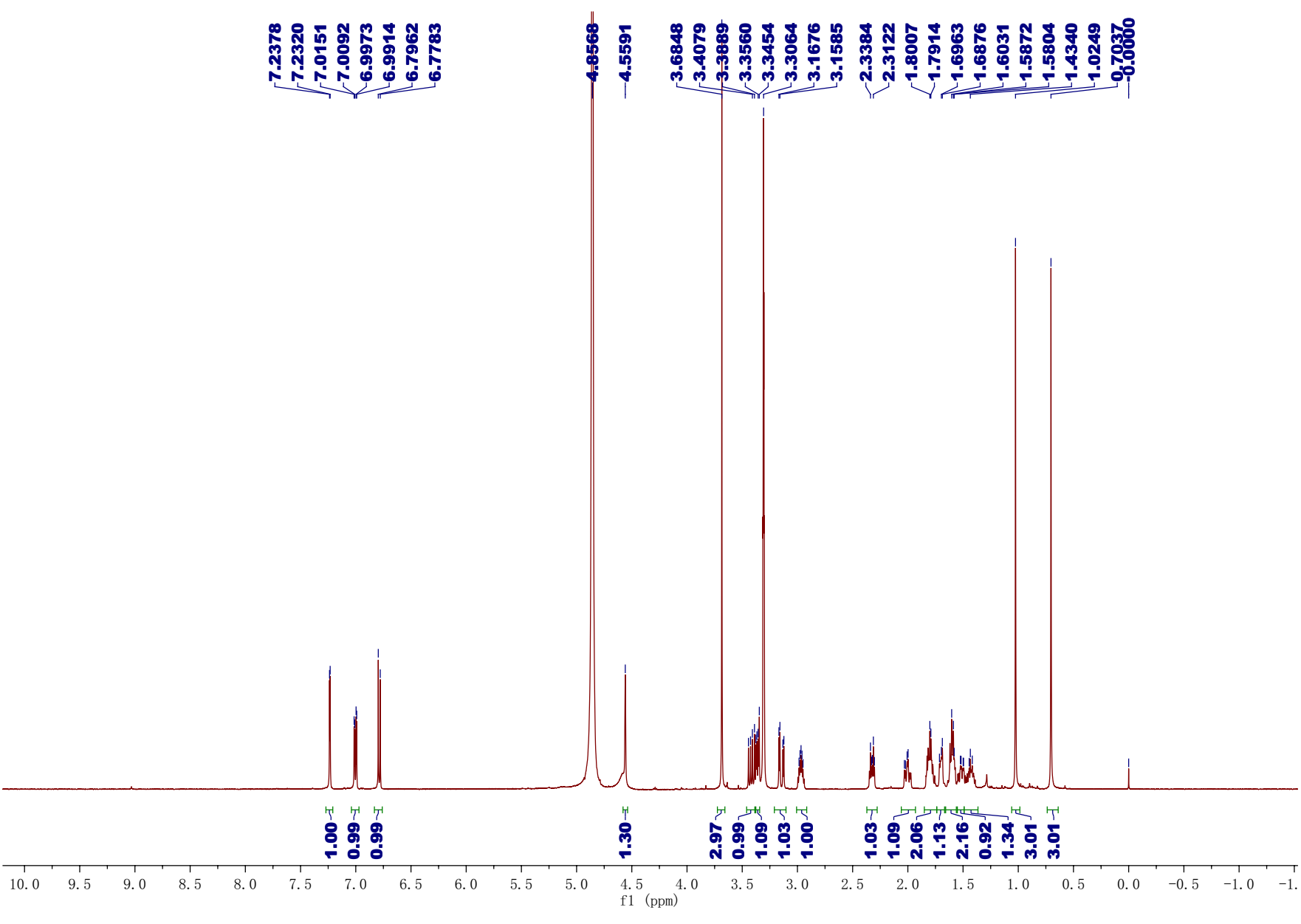


Figure S65. ^1^H NMR spectrum of **11** in methanol-*d*_4_.


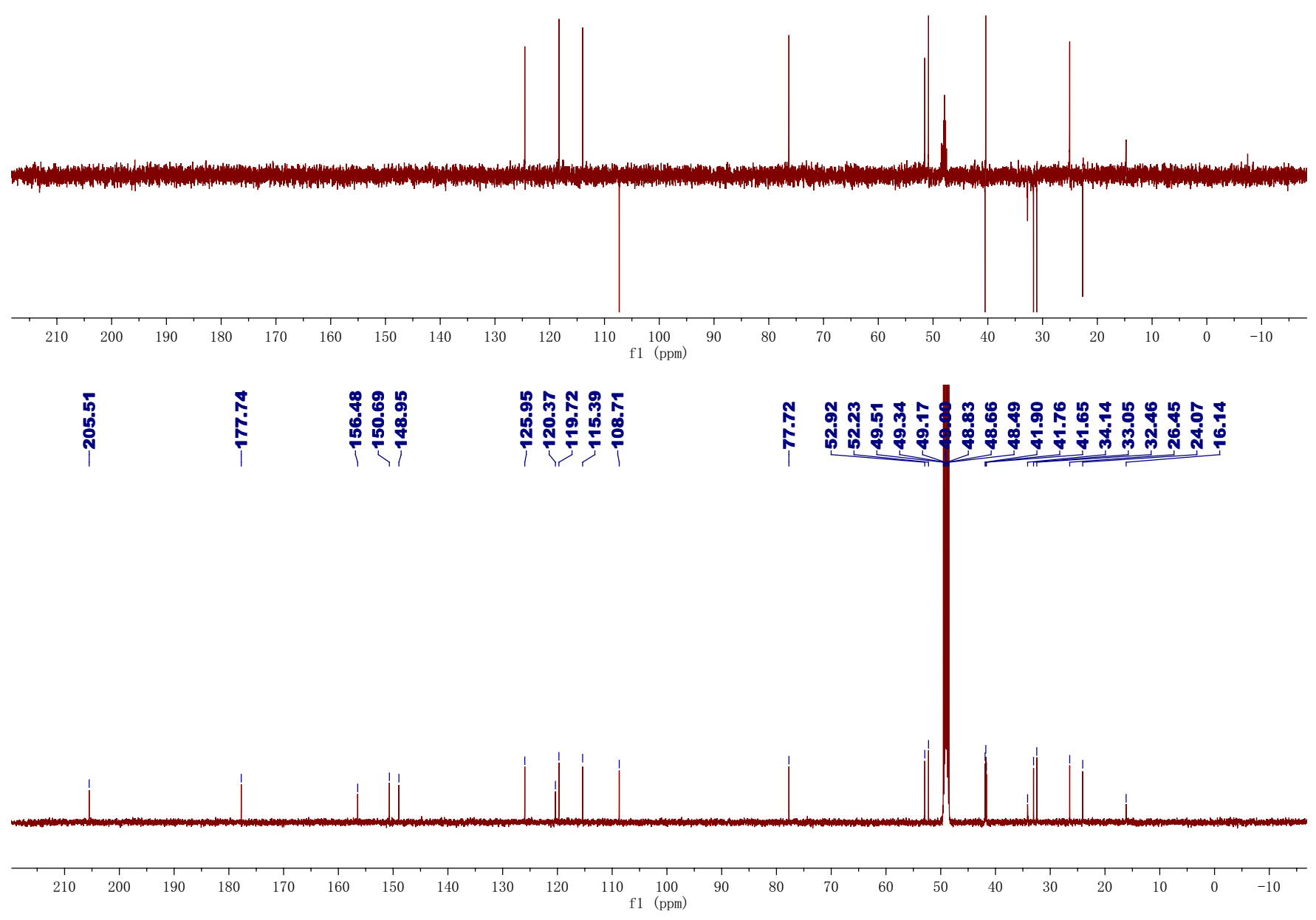


Figure S66. ^13^C NMR and DEPT spectra of **11** in methanol-*d*_4_.


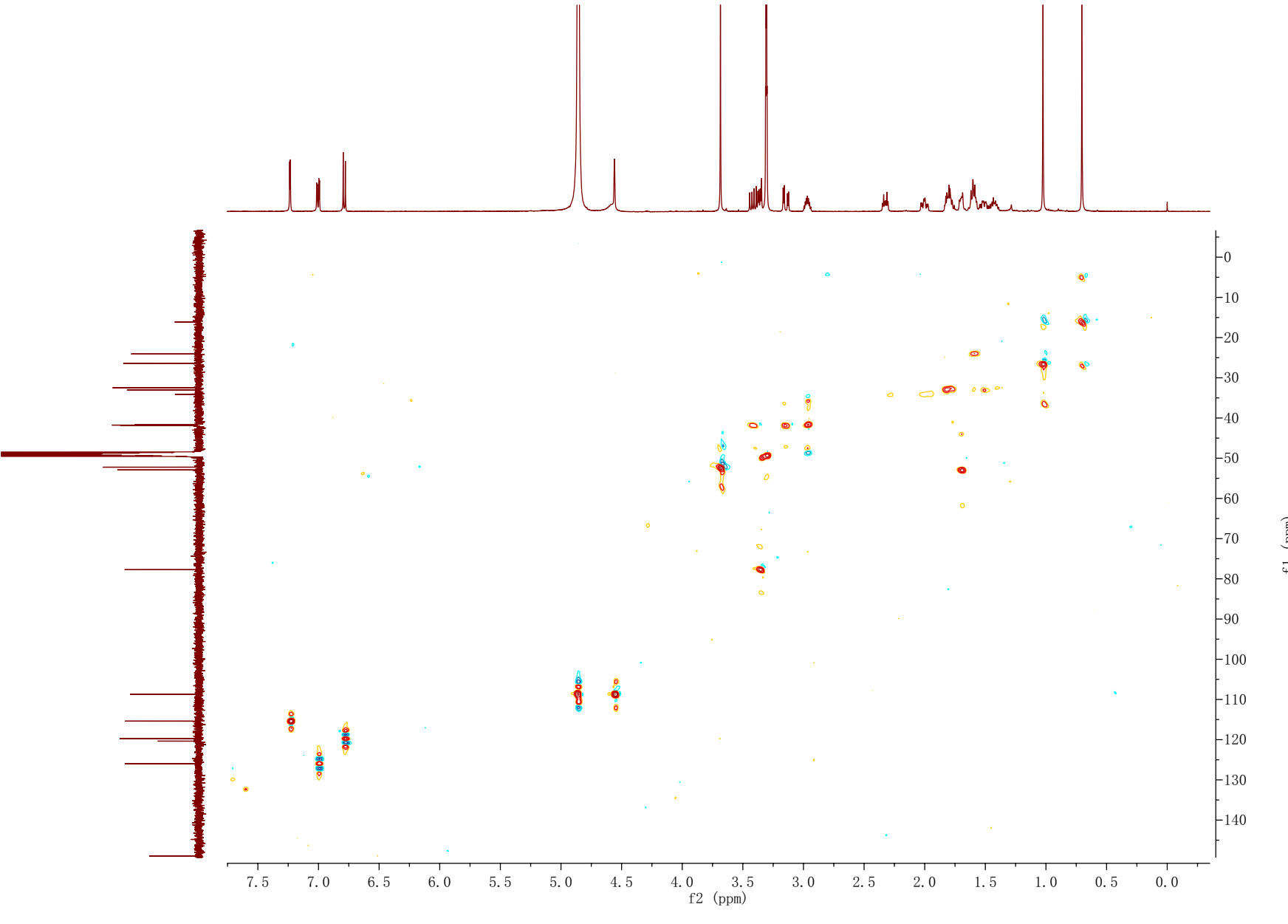


Figure S67. HSQC spectrum of **11** in methanol-*d*_4_.


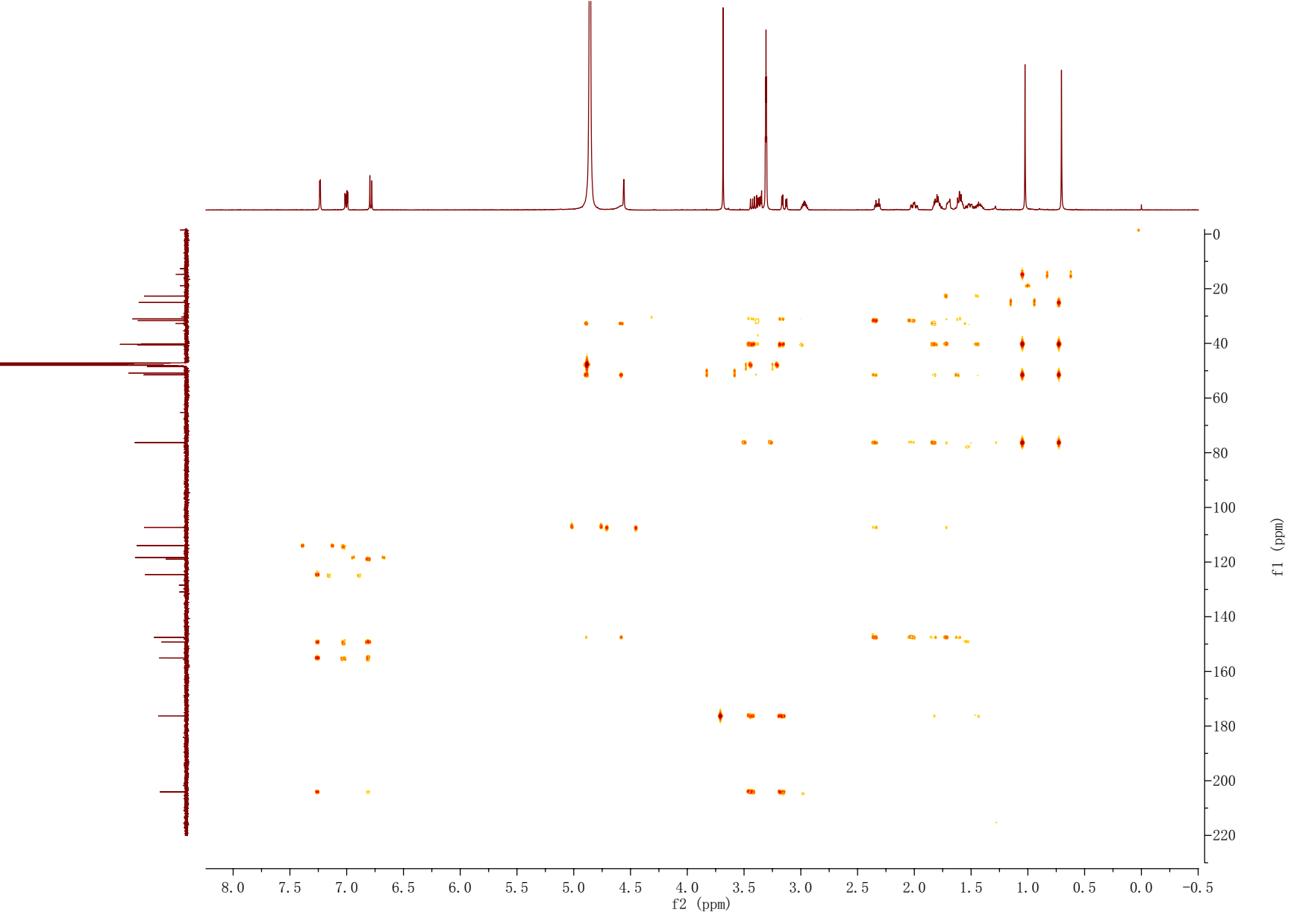


Figure S68. HMBC spectrum of **11** in methanol-*d*_4_.


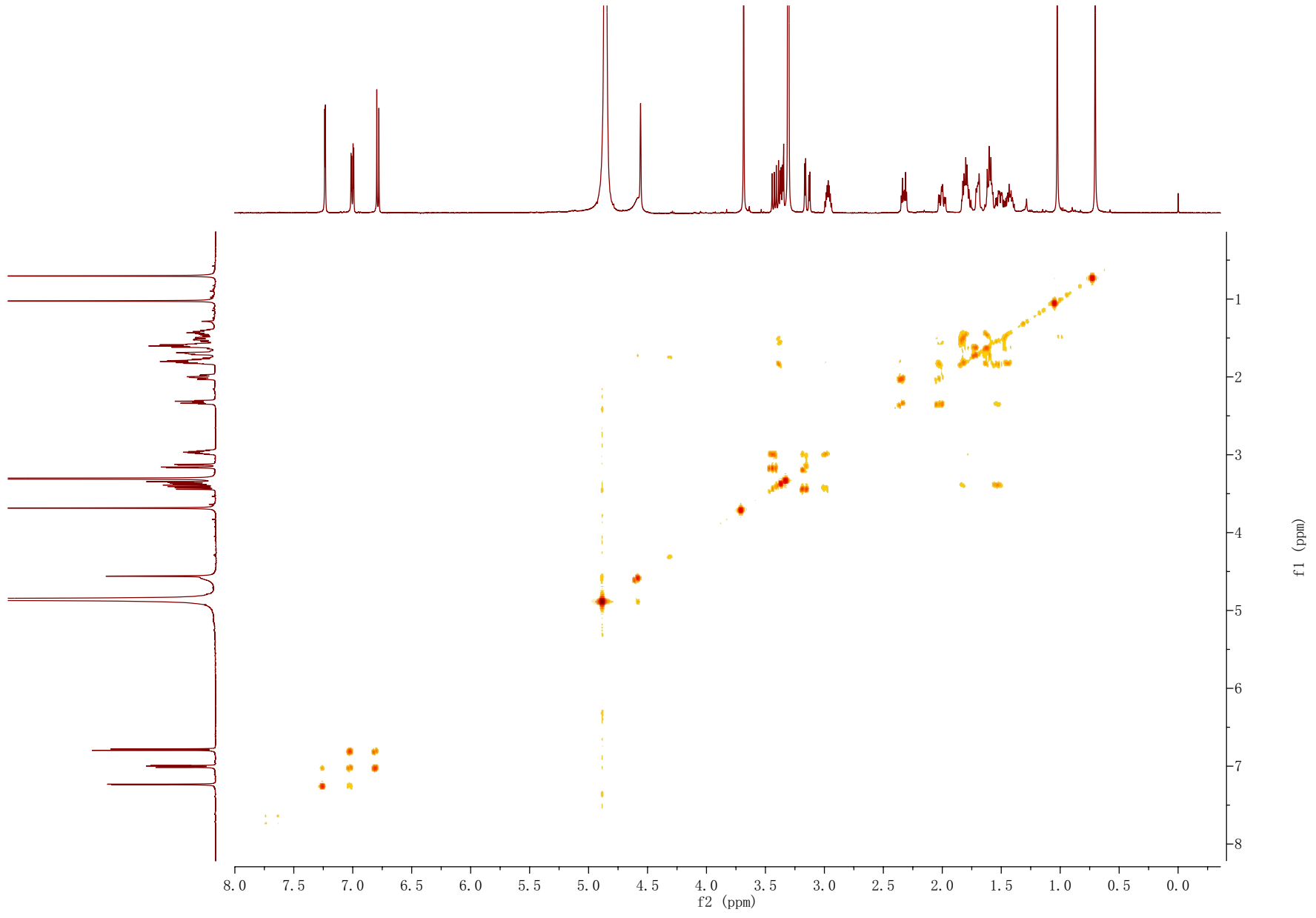


Figure S69. ^1^H-^1^H COSY spectrum of **11** in methanol-*d*_4_.


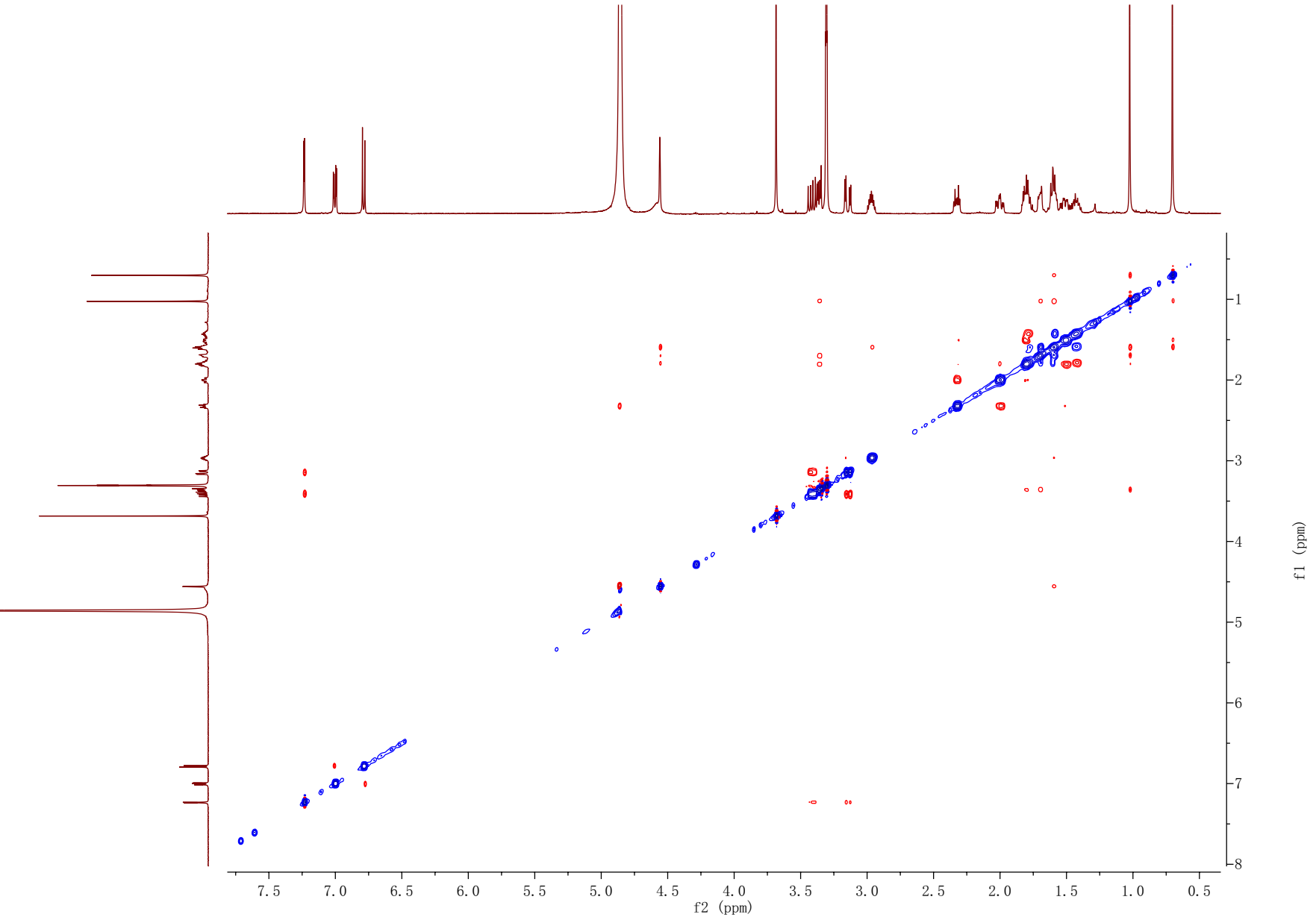


Figure S70. ROESY spectrum of **11** in methanol-*d*_4_.

[M+H]^+^ m/z 391.2120

| Hit | Formula | m/z | RDB | ppm |
| --- | --- | --- | --- | --- |
| 1 | C_22_H_31_O_6_ | 391.2115 | 8.0 | 1.2 |

Figure S71. HRESIMS of **11**.


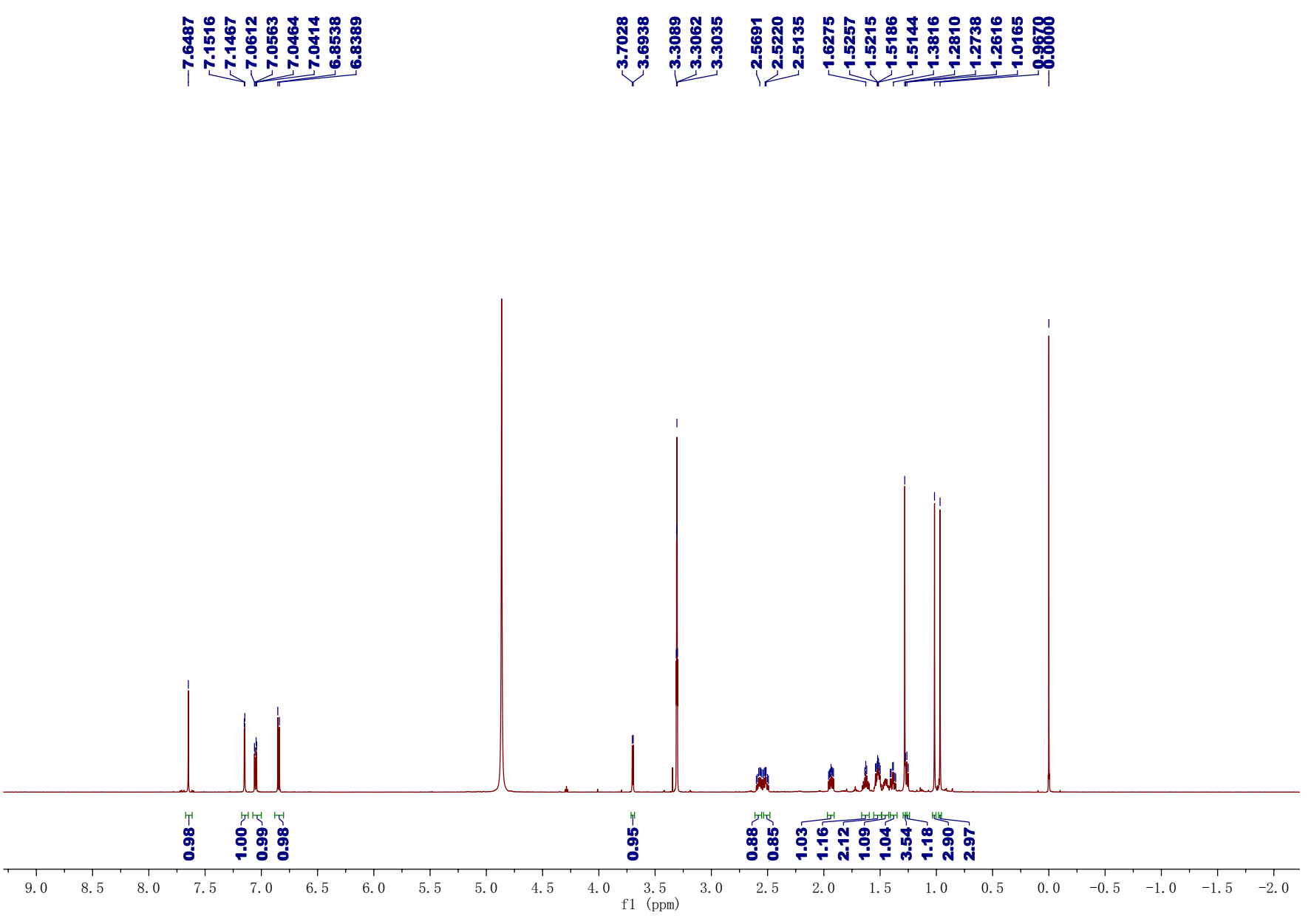


Figure S72. ^1^H NMR spectrum of **12** in methanol-*d*_4_.


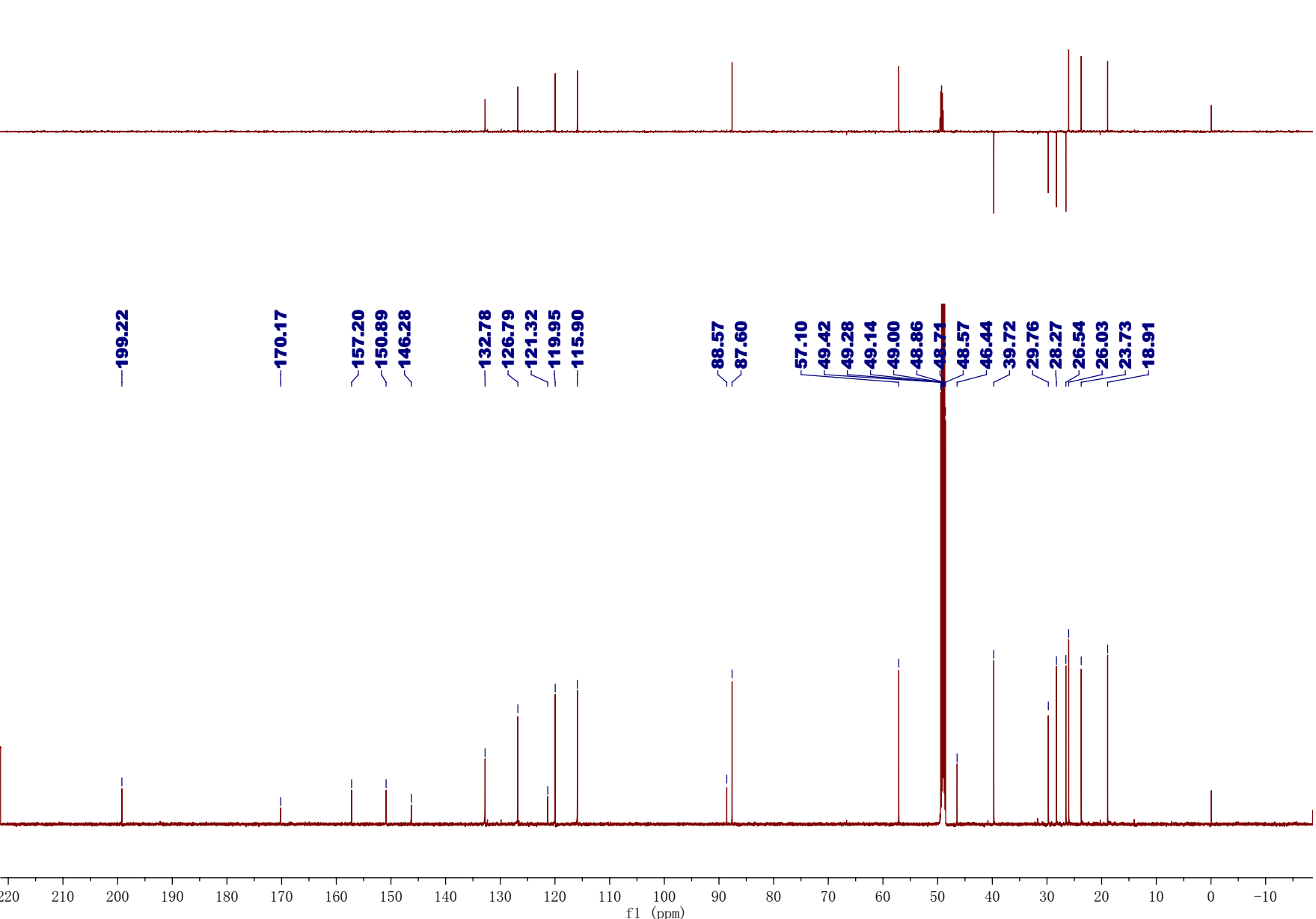


Figure S73. ^13^C NMR and DEPT spectra of **12** in methanol-*d*_4_.


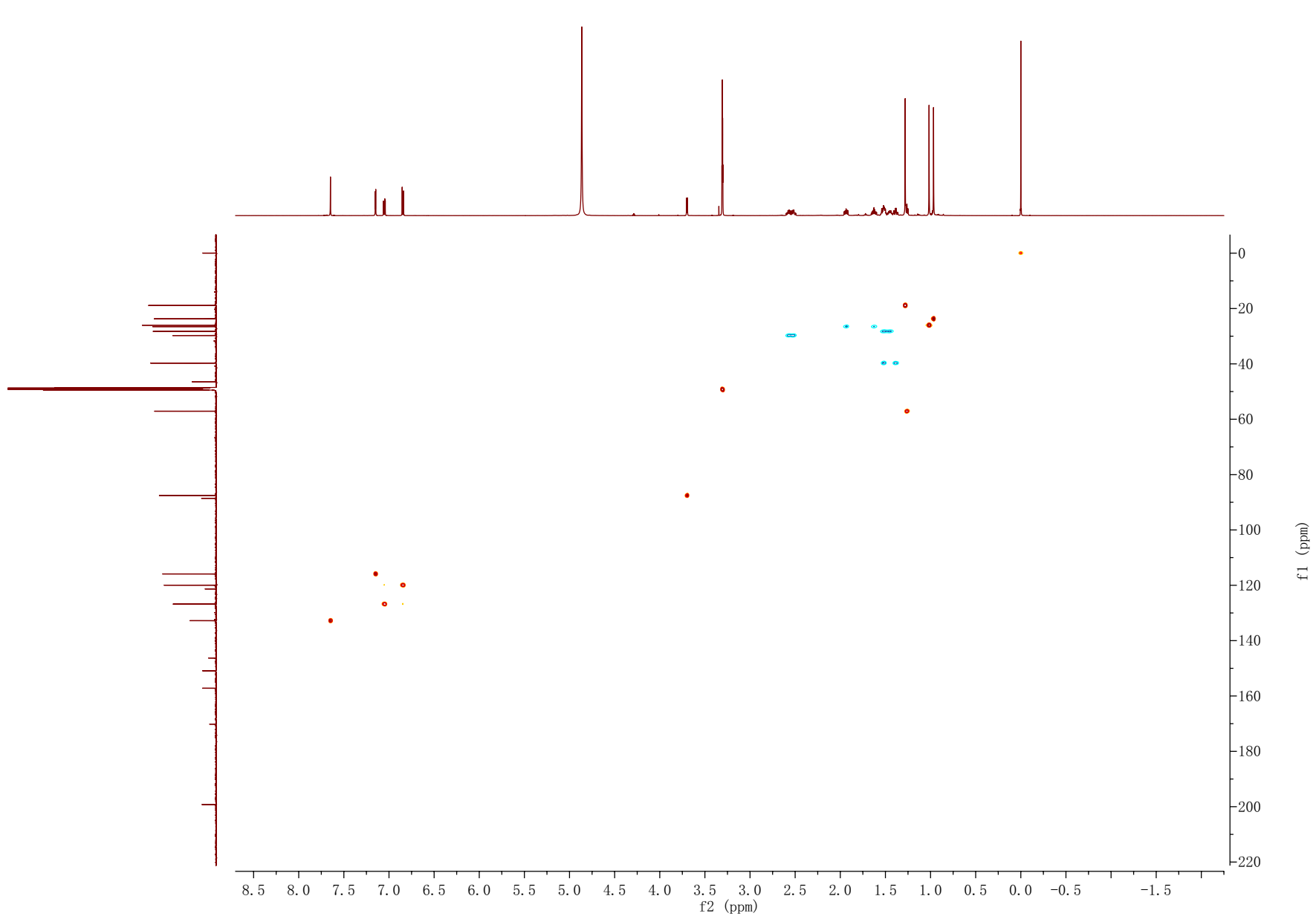


Figure S74. HSQC spectrum of **12** in methanol-*d*_4_.


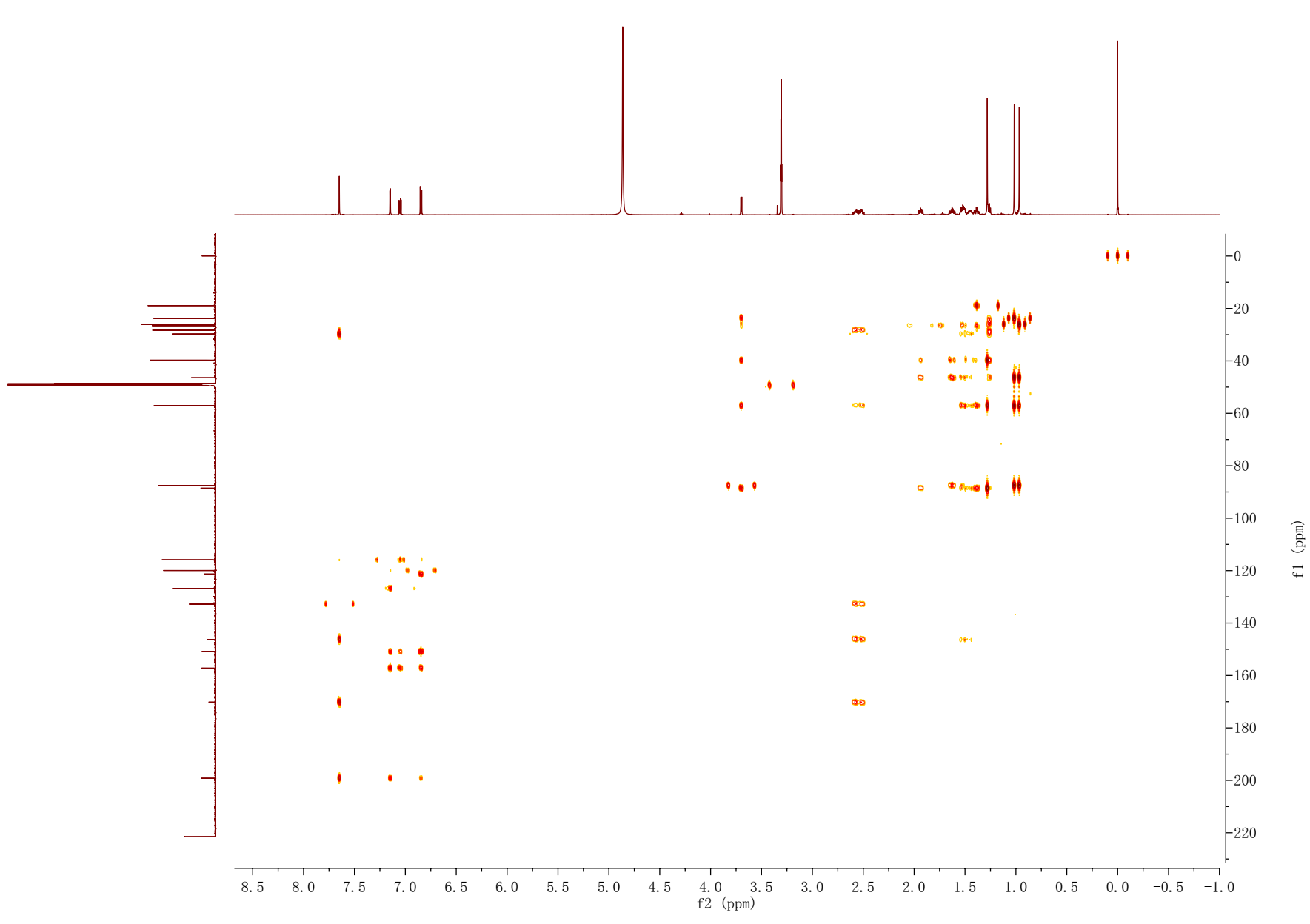


Figure S75. HMBC spectrum of **12** in methanol-*d*_4_.


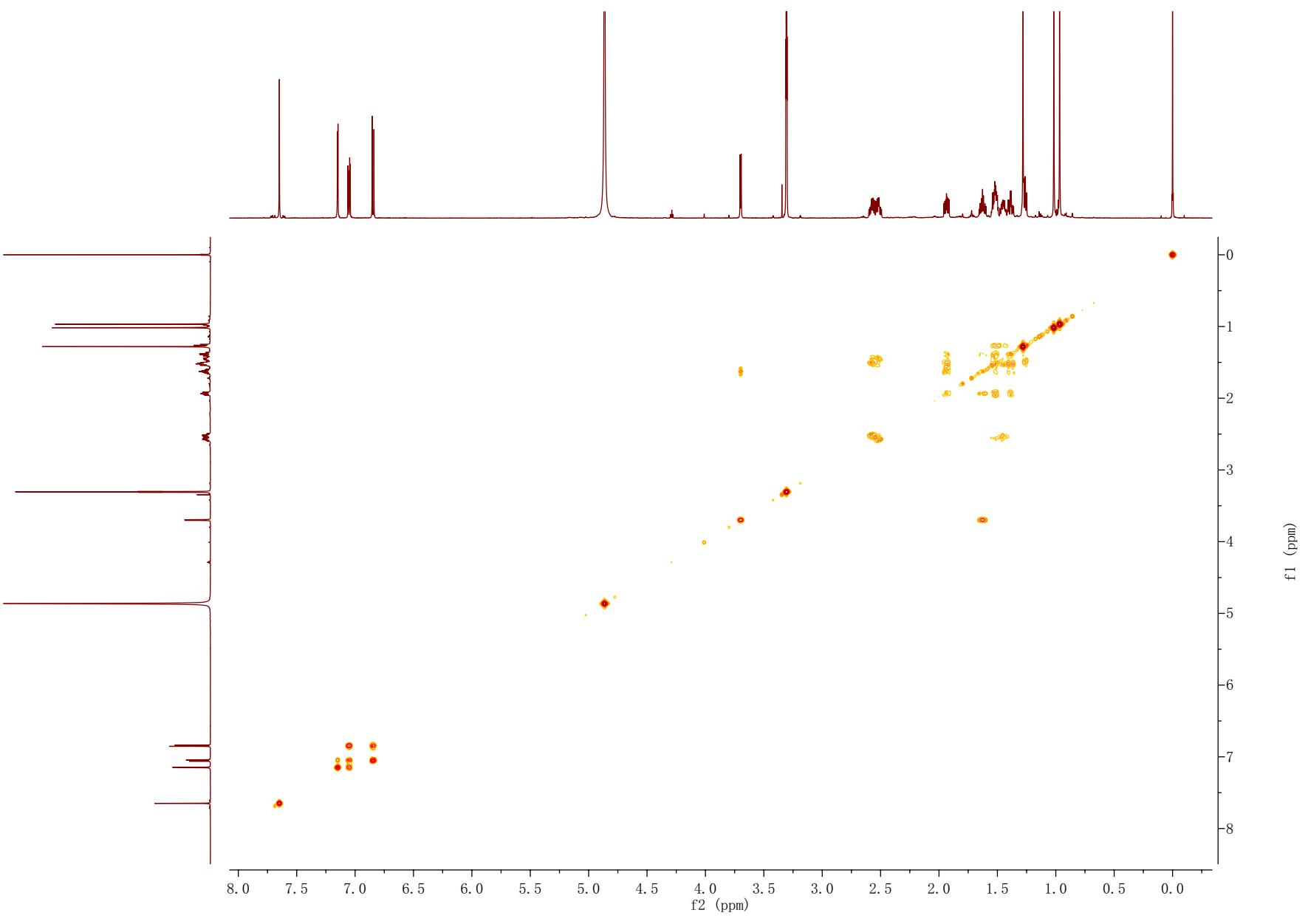


Figure S76. ^1^H-^1^H COSY spectrum of **12** in methanol-*d*_4_.


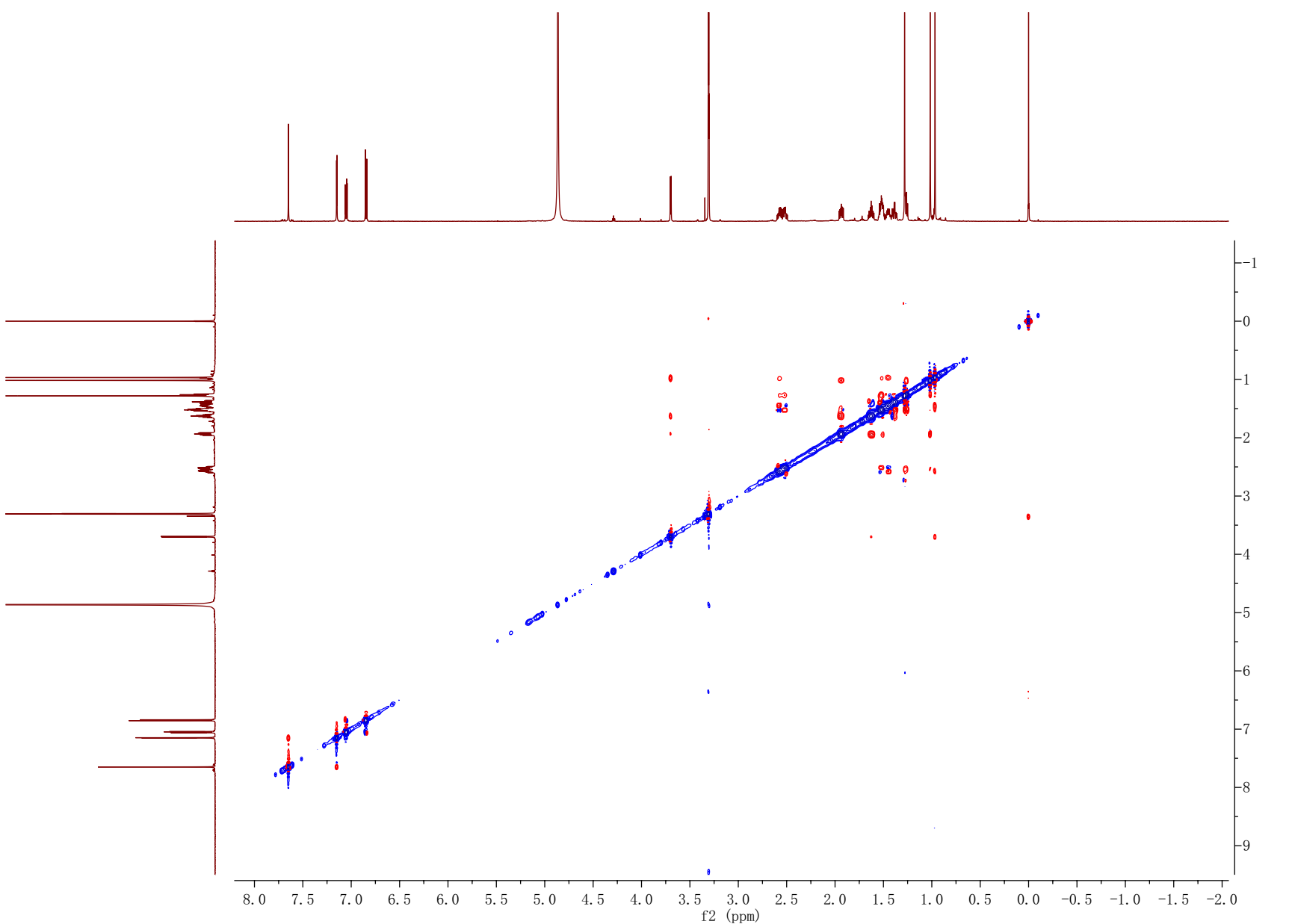


Figure S77. ROESY spectrum of **12** in methanol-*d*_4_.

[M+H]^+^ m/z 375.1812

| Hit | Formula | m/z | RDB | ppm |
| --- | --- | --- | --- | --- |
| 1 | C_21_H_26_O_6_ | 375.1802 | 9.0 | 2.6 |

Figure S78. HRESIMS of **12**.


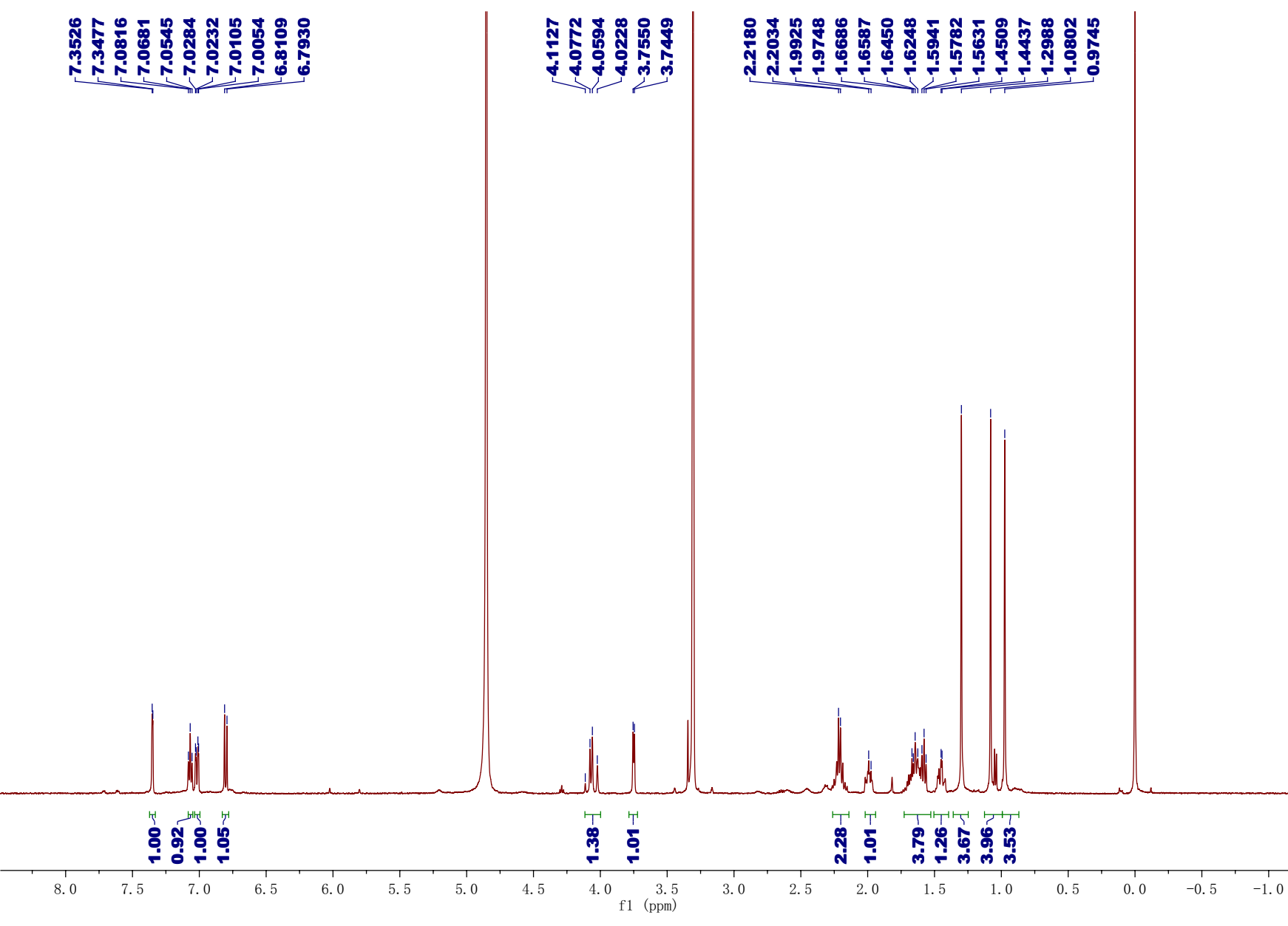


Figure S79. ^1^H NMR spectrum of **13** in methanol-*d*_4_.


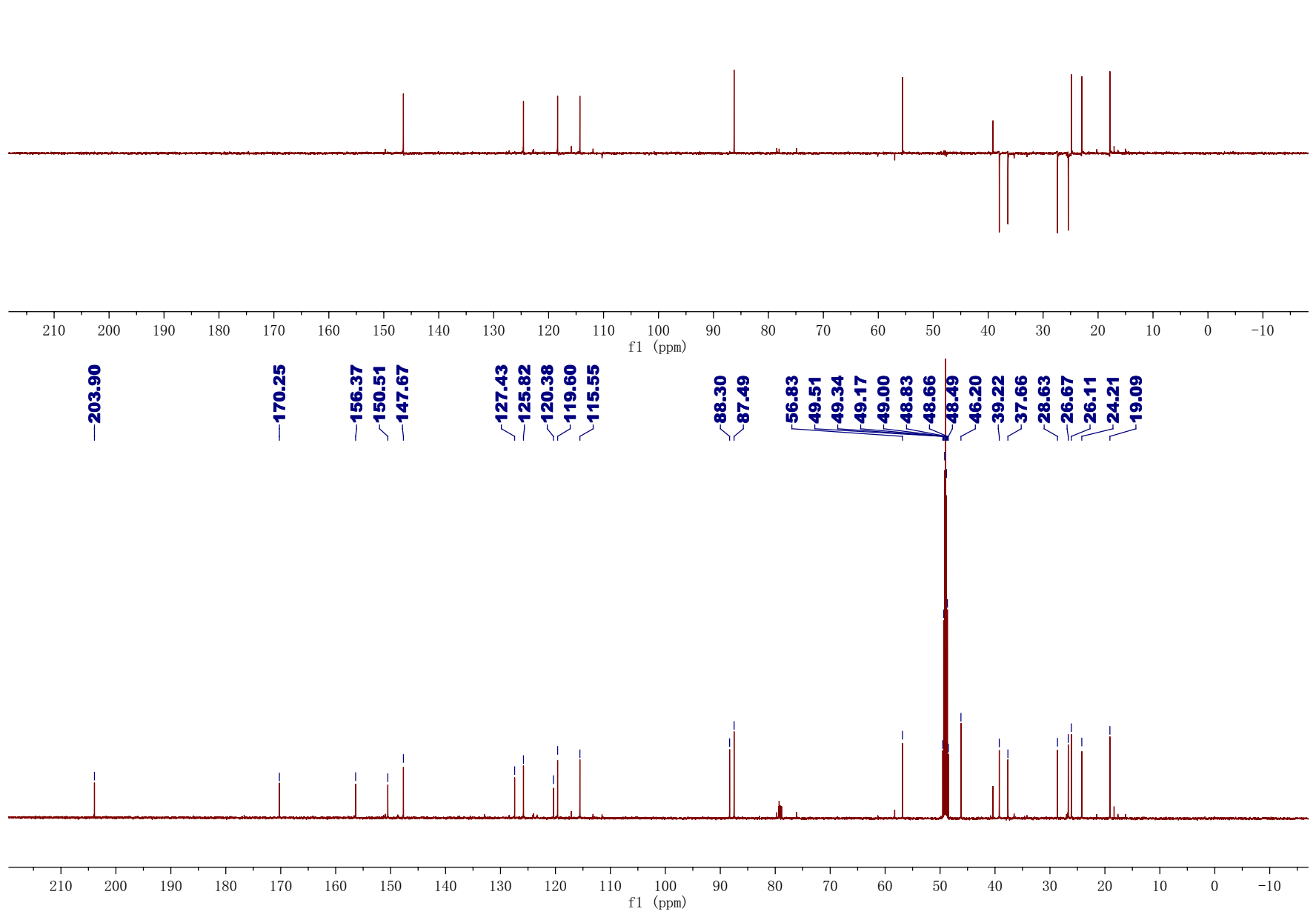


Figure S80. ^13^C NMR and DEPT spectra of **13** in methanol-*d*_4_.


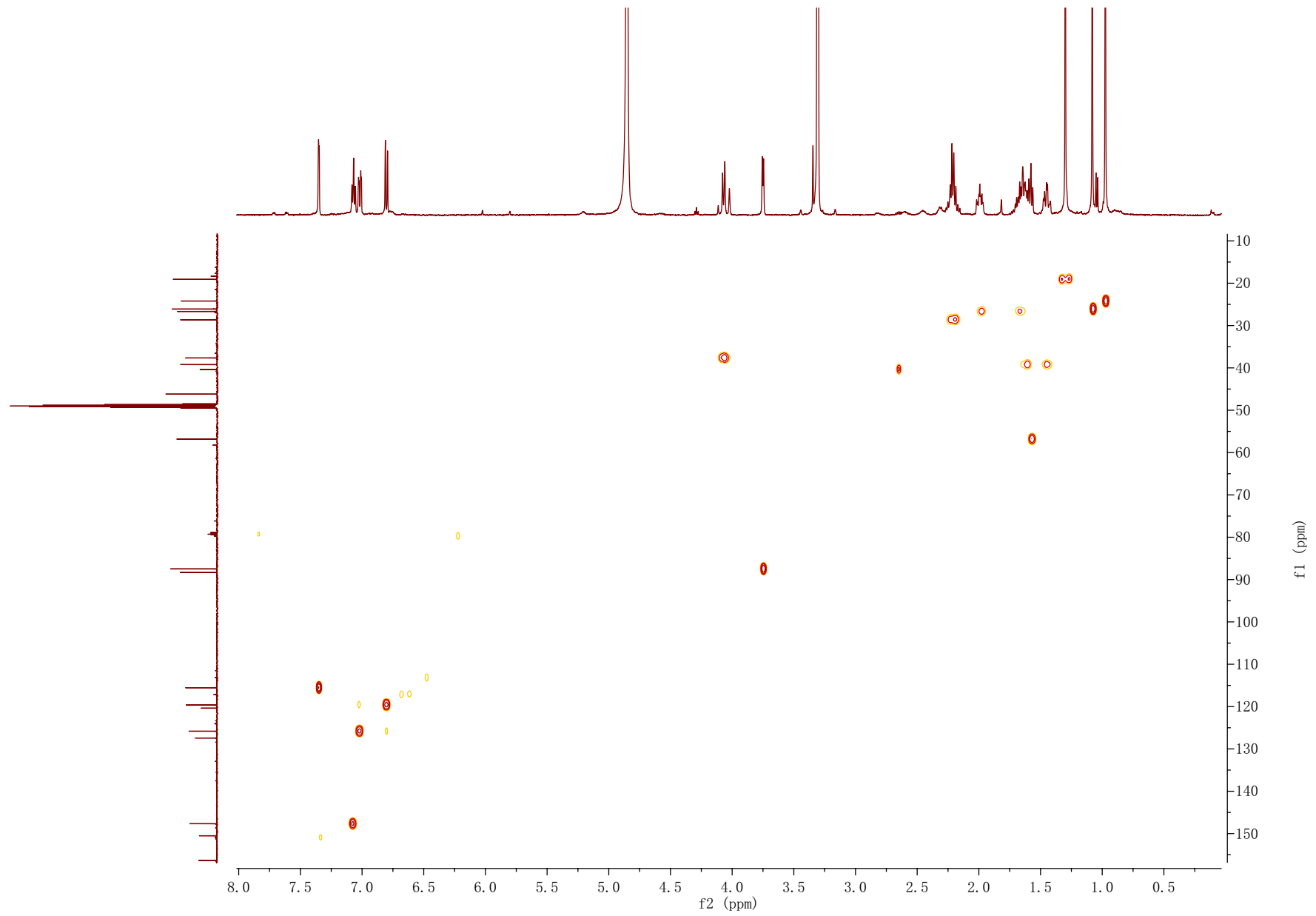


Figure S81. HSQC spectrum of **13** in methanol-*d*_4_.


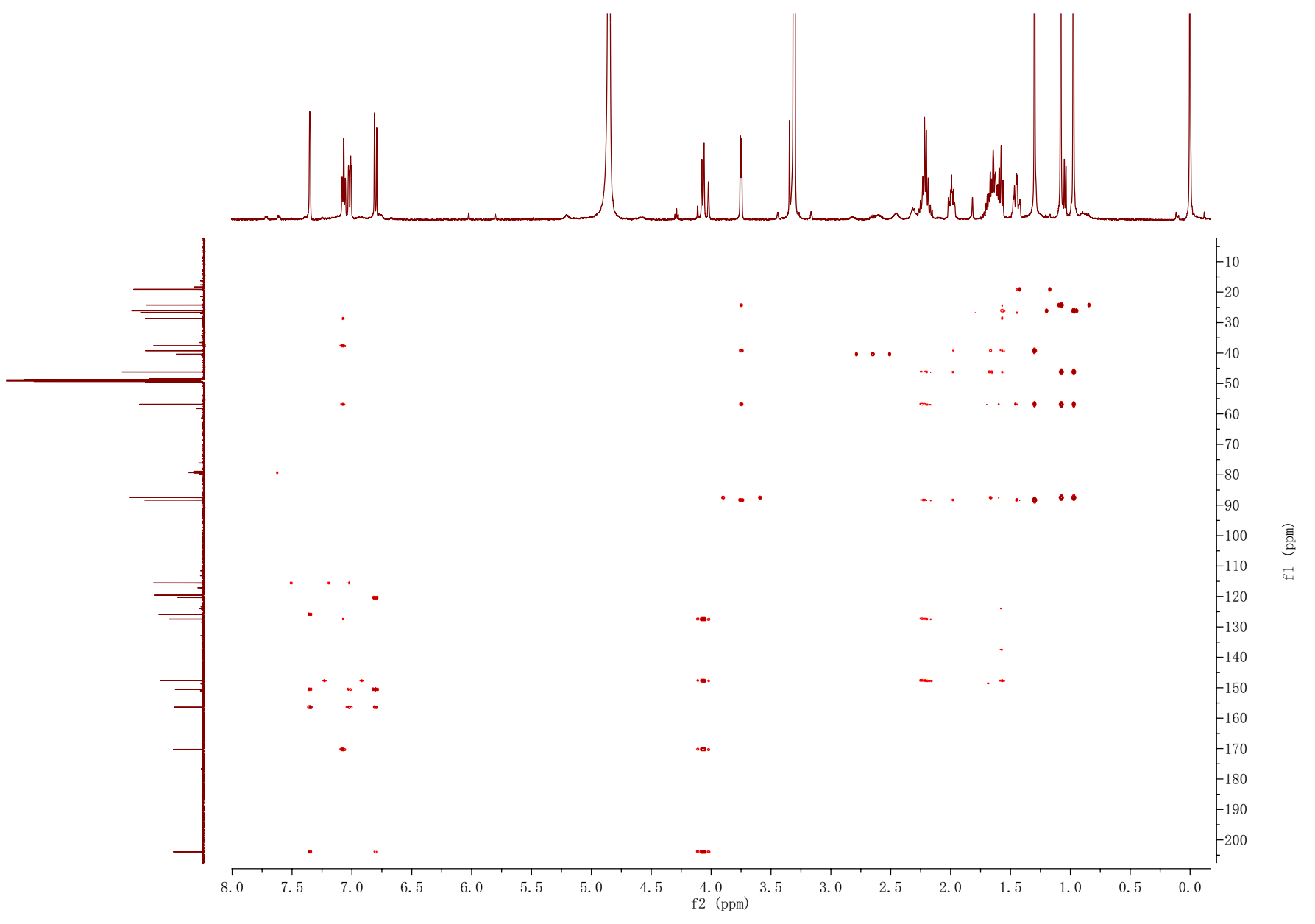


Figure S82. HMBC spectrum of **13** in methanol-*d*_4_.


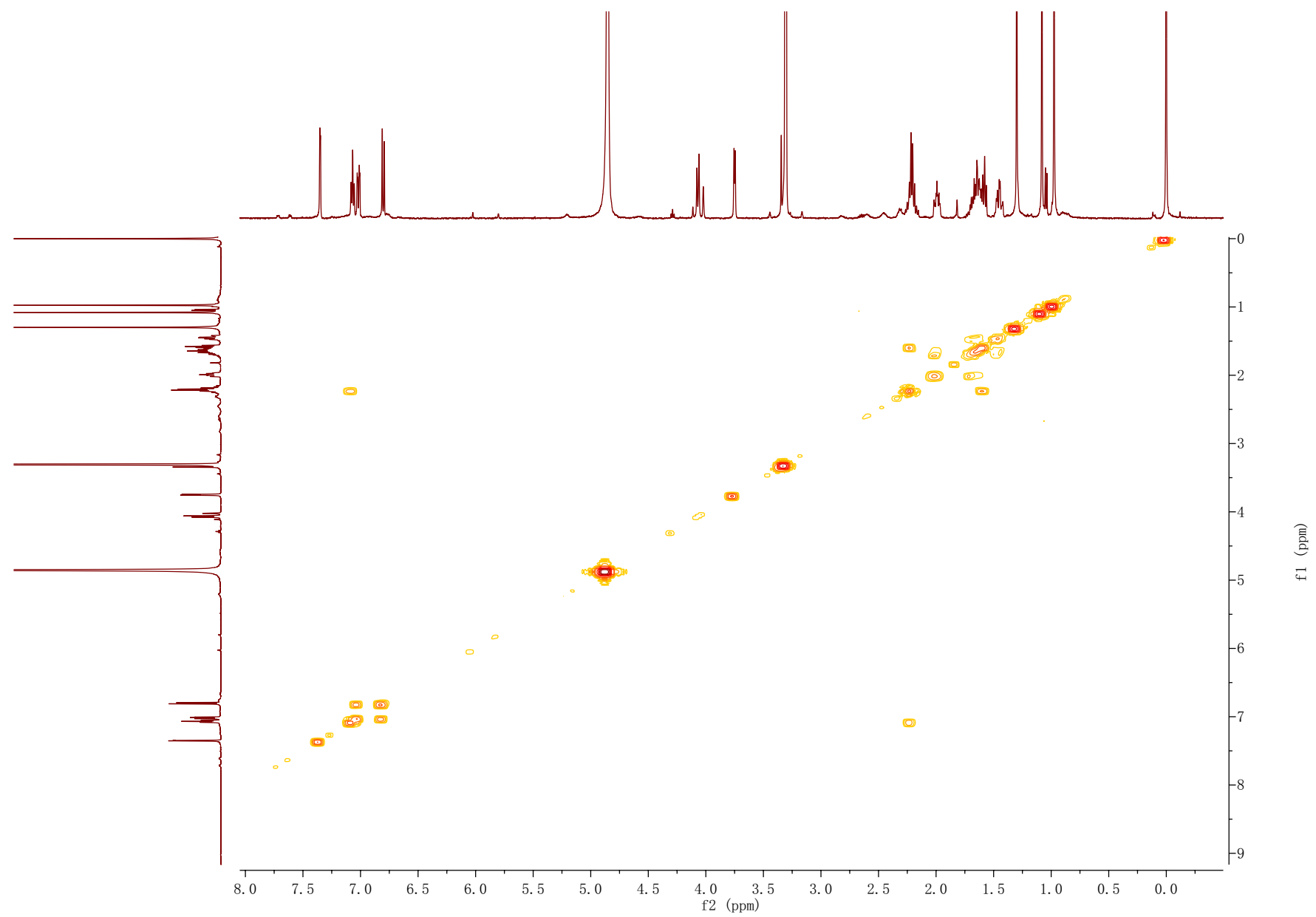


Figure S83. ^1^H-^1^H COSY spectrum of **13** in methanol-*d*_4_.


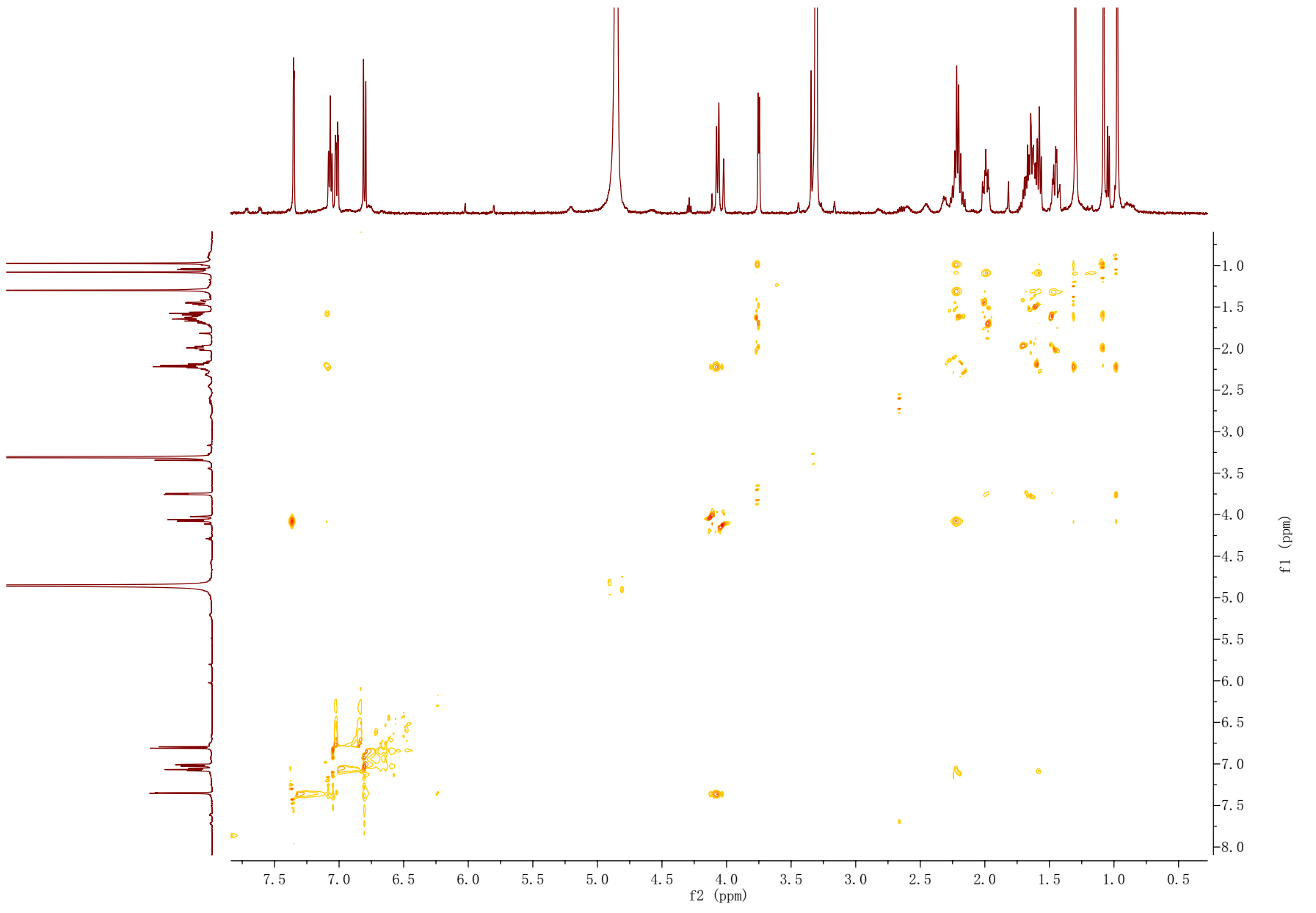


Figure S84. ROESY spectrum of **13** in methanol-*d*_4_.

[M+H]^+^ m/z 375.1805

| Hit | Formula | m/z | RDB | ppm |
| --- | --- | --- | --- | --- |
| 1 | C_21_H_27_O_6_ | 375.1802 | 9.0 | 0.8 |

Figure S85. HRESIMS of **13**.


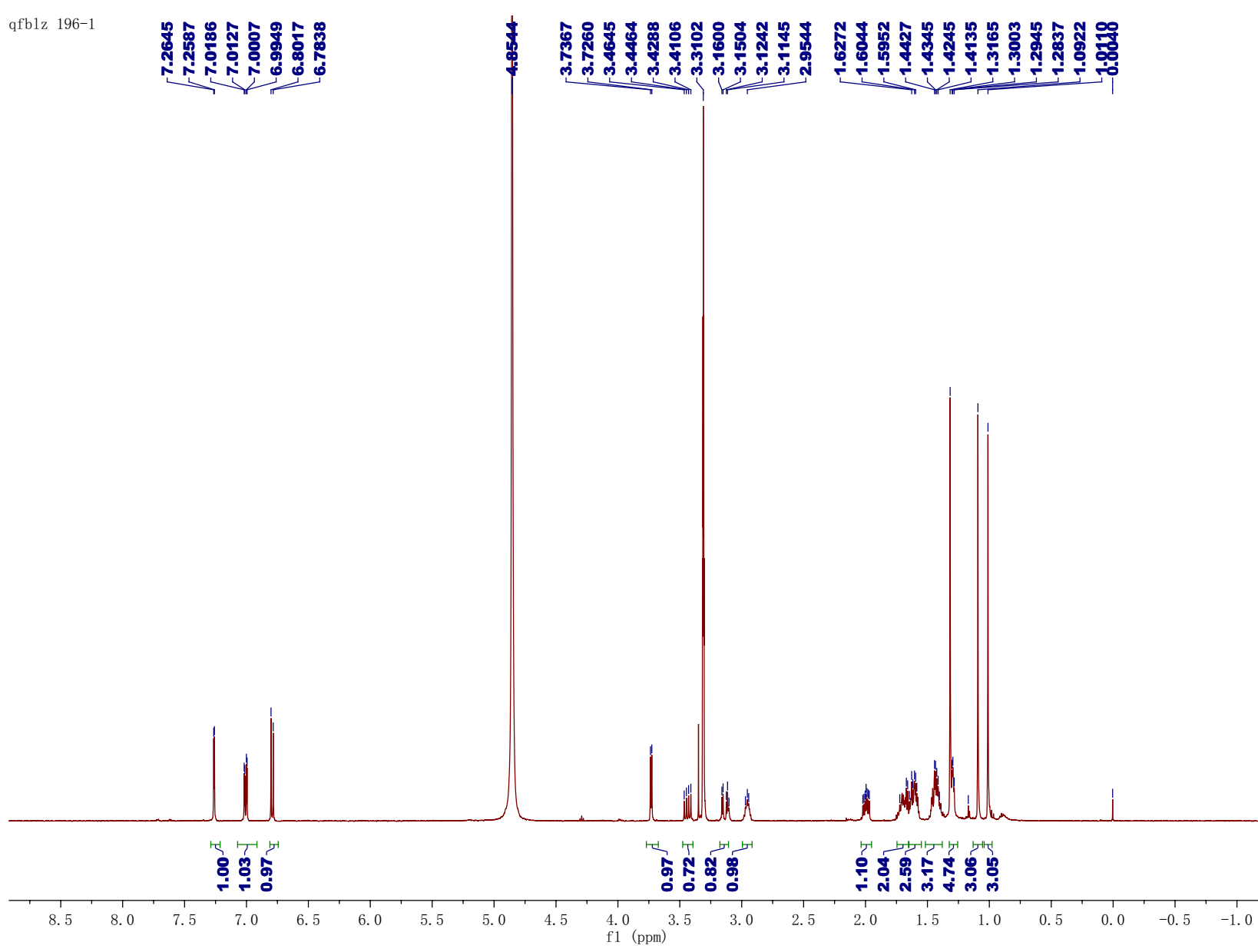


Figure S86. ^1^H NMR spectrum of **14** in methanol-*d*_4_.


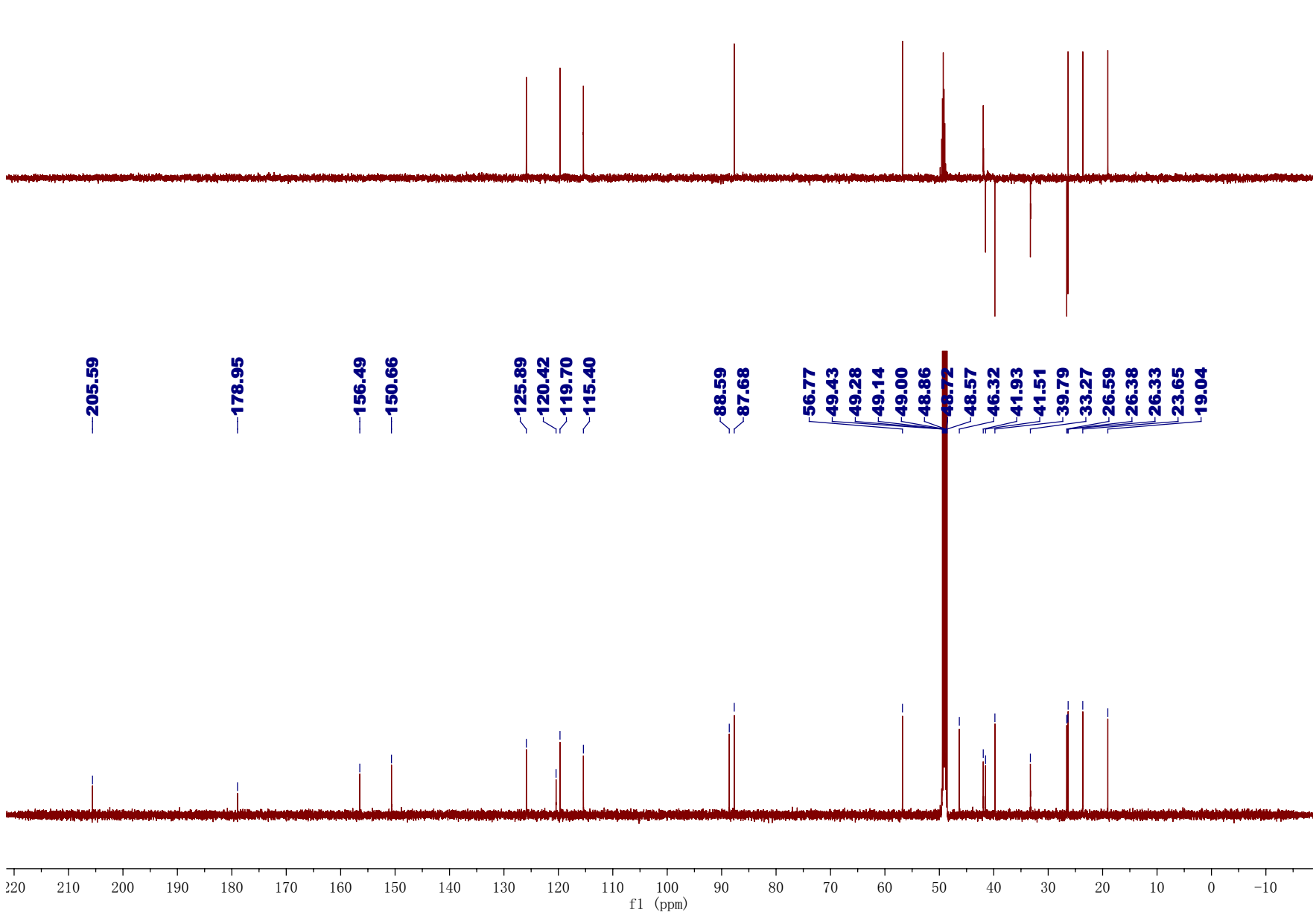


Figure S87. ^13^C NMR and DEPT spectra of **14** in methanol-*d*_4_.


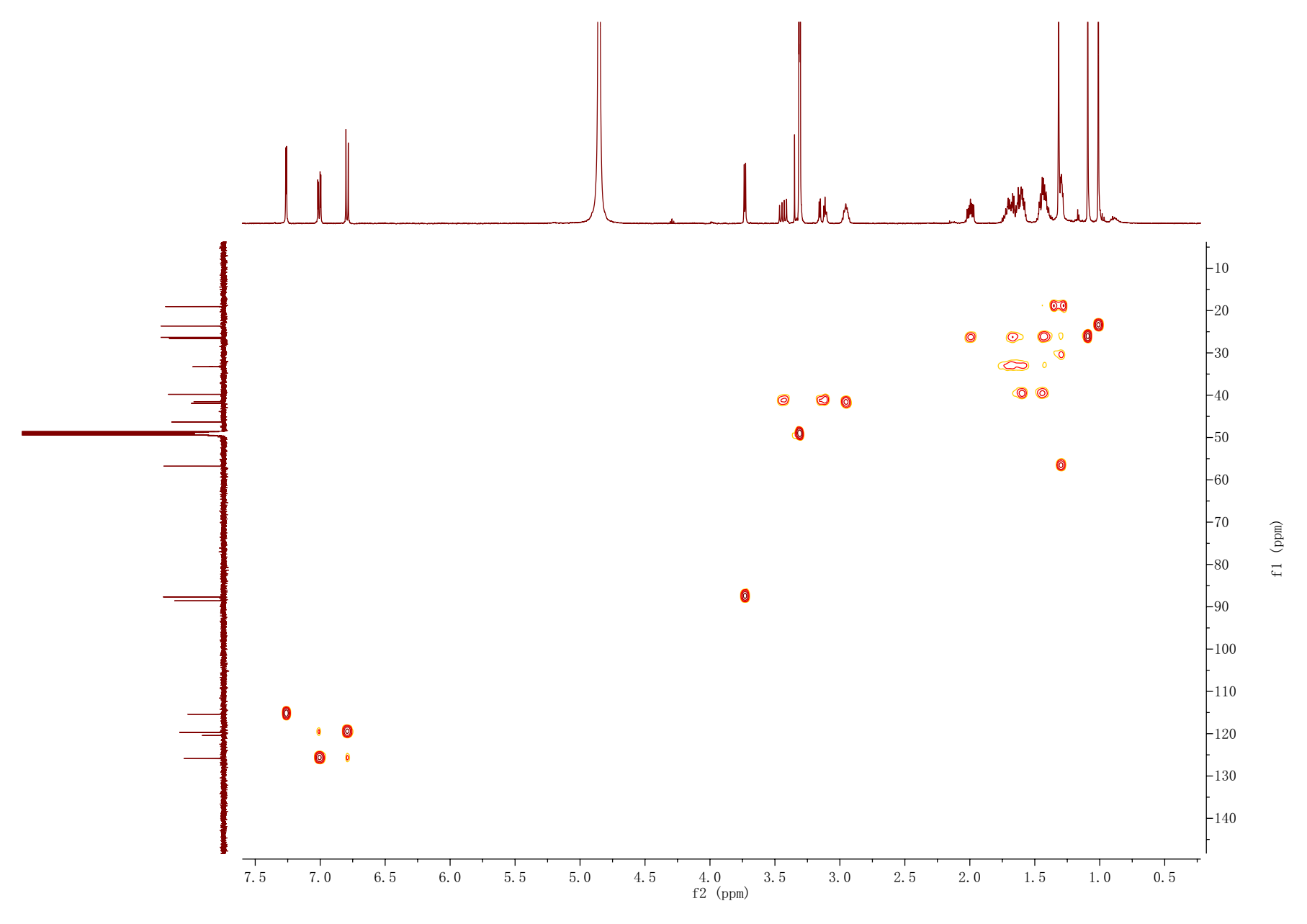


Figure S88. HSQC spectrum of **14** in methanol-*d*_4_.


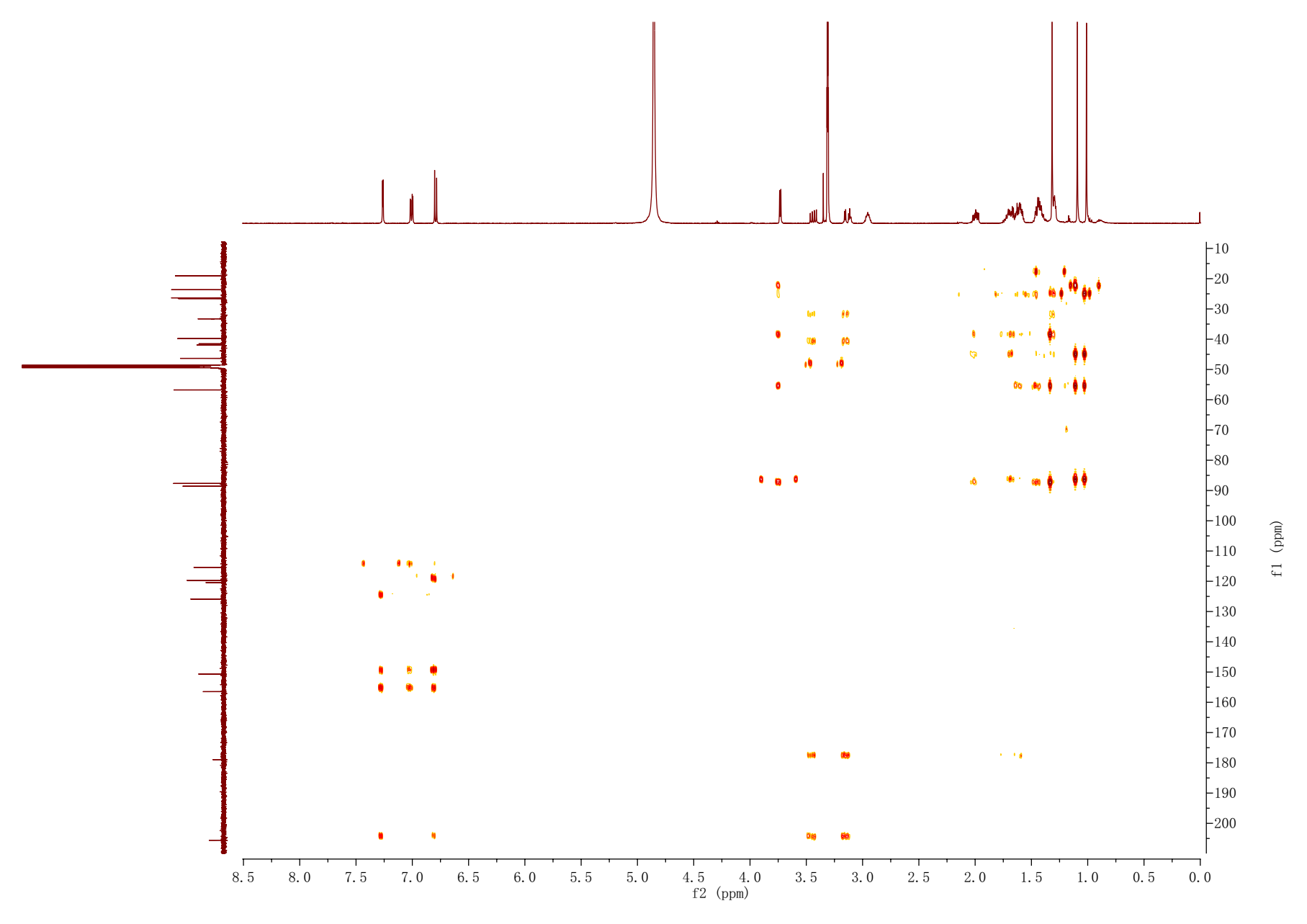


Figure S89. HMBC spectrum of **14** in methanol-*d*_4_.


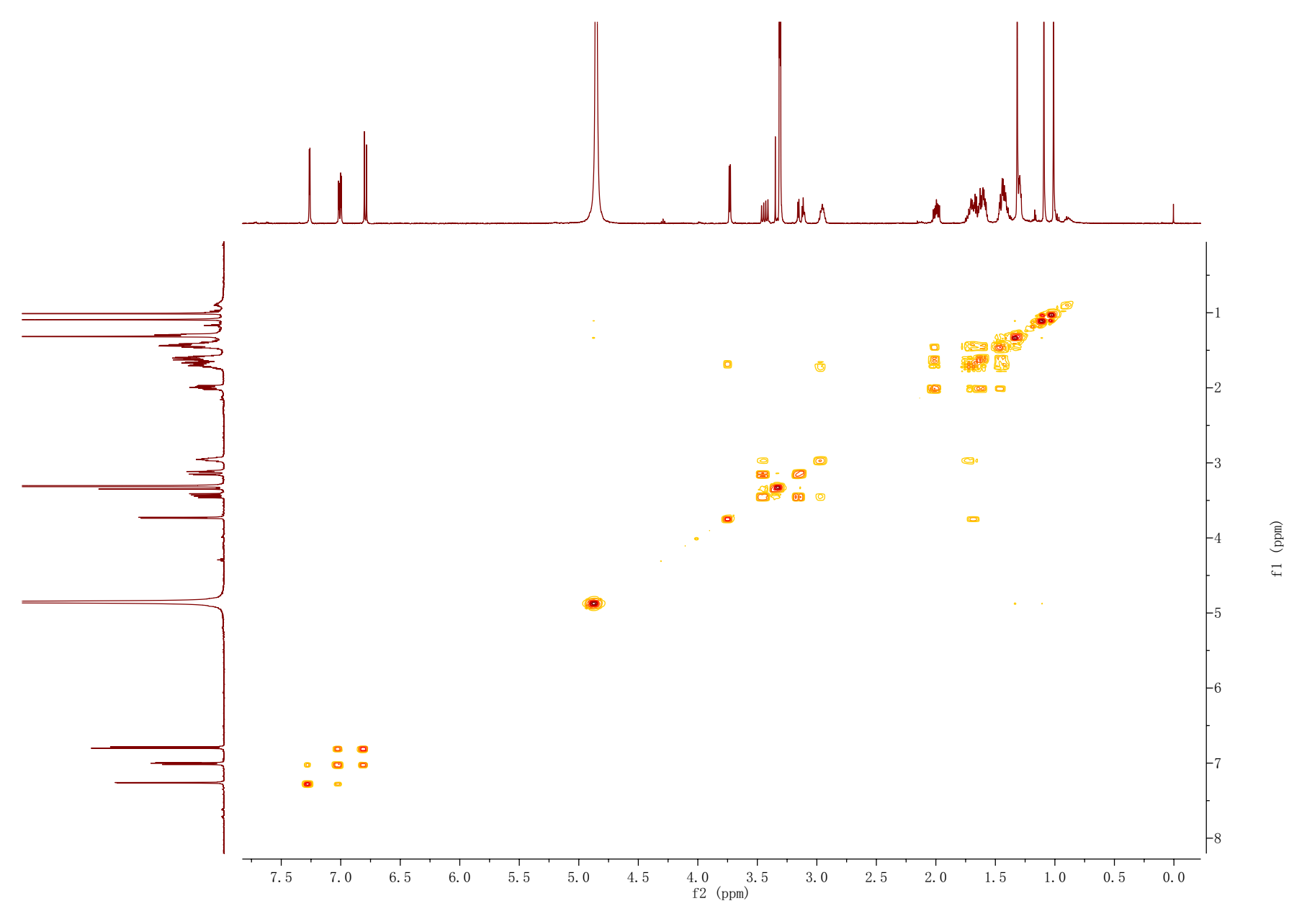


Figure S90. ^1^H-^1^H COSY spectrum of **14** in methanol-*d*_4_.


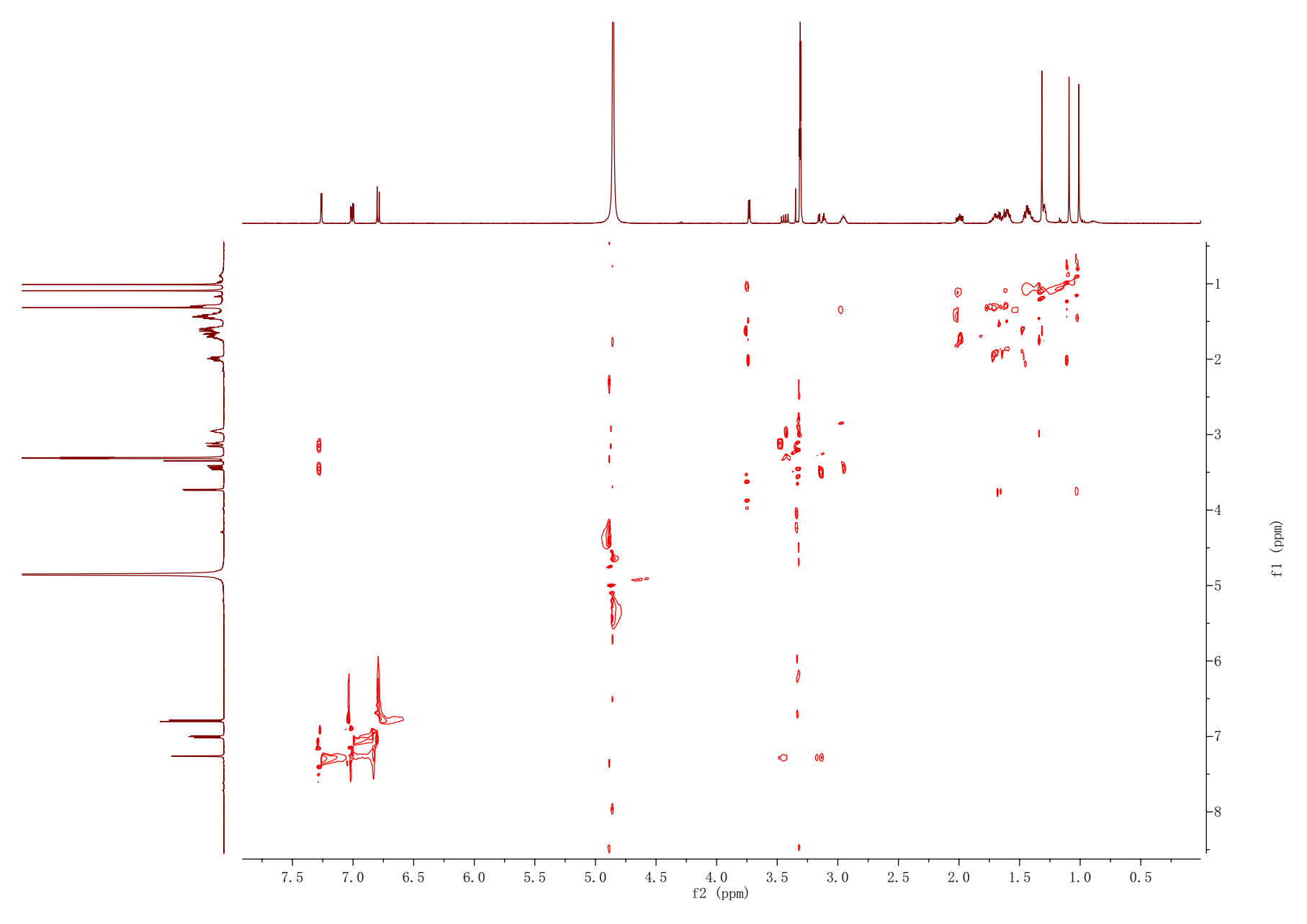


Figure S91. ROESY spectrum of **14** in methanol-*d*_4_.

[M+H]^+^ m/z 377.1965

| Hit | Formula | m/z | RDB | ppm |
| --- | --- | --- | --- | --- |
| 1 | C_21_H_29_O_6_ | 377.1959 | 8.0 | 1.7 |

Figure S92. HRESIMS of **14**.


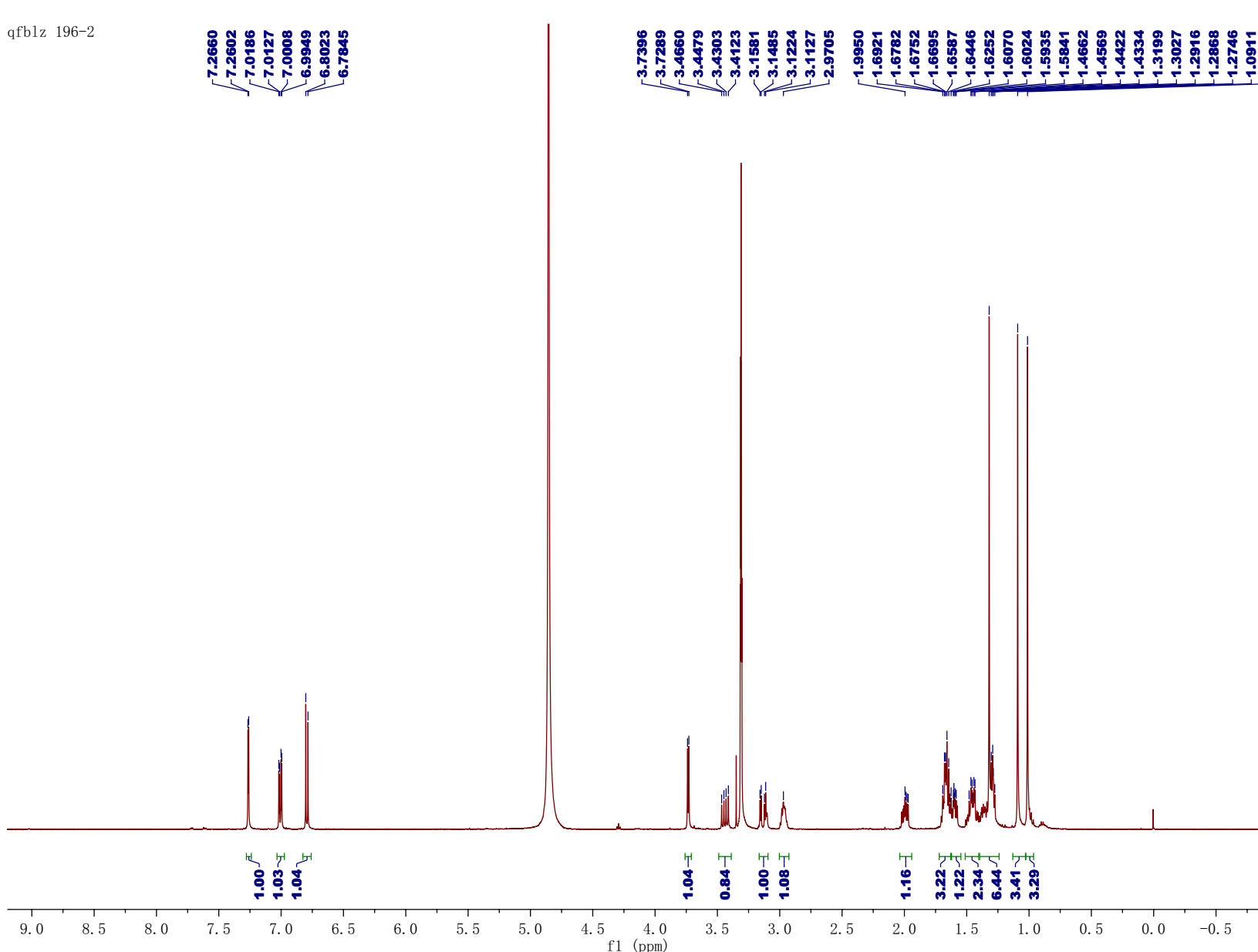


Figure S93. ^1^H NMR spectrum of **15** in methanol-*d*_4_.


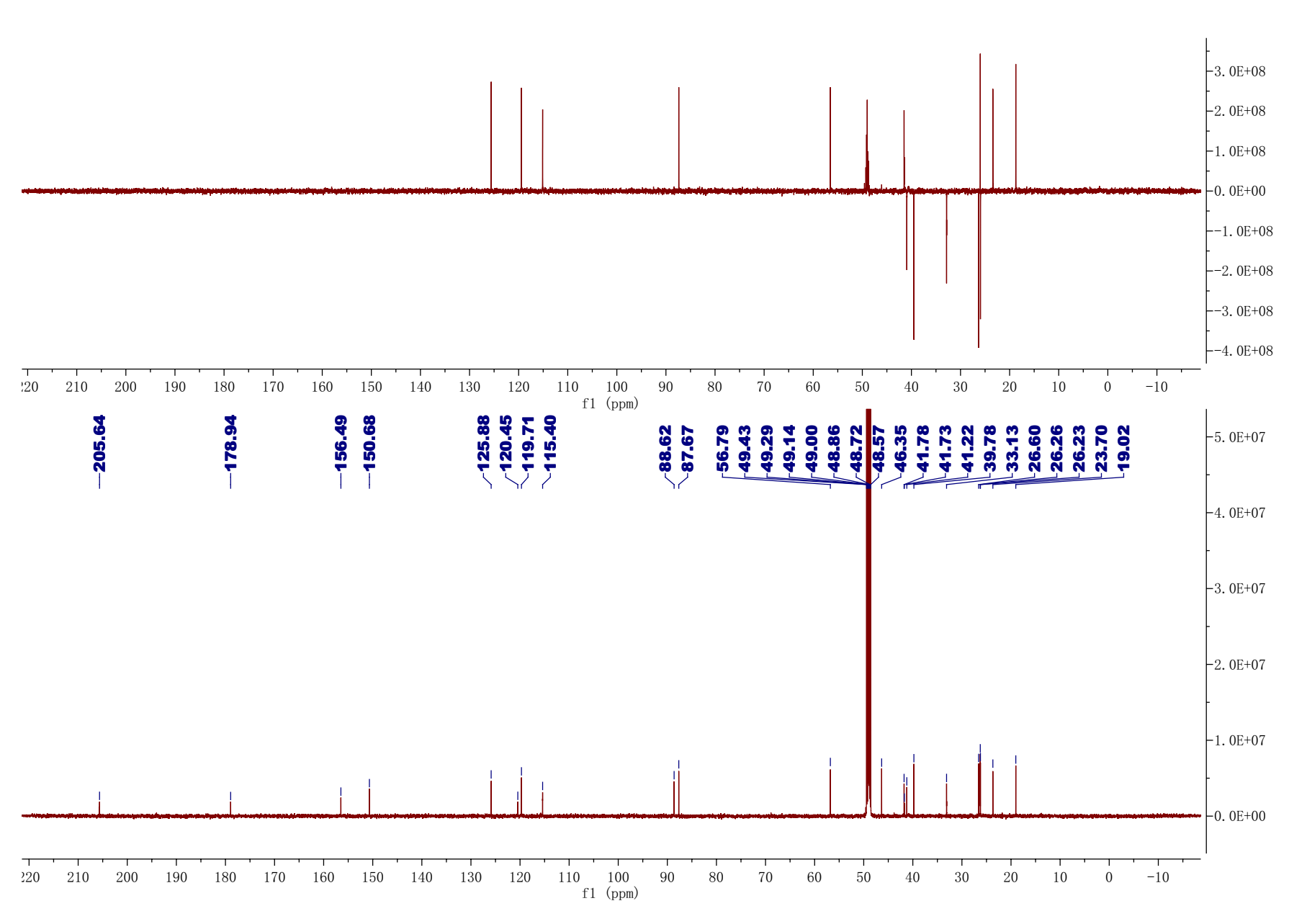


Figure S94. ^13^C NMR and DEPT spectra of **15** in methanol-*d*_4_.


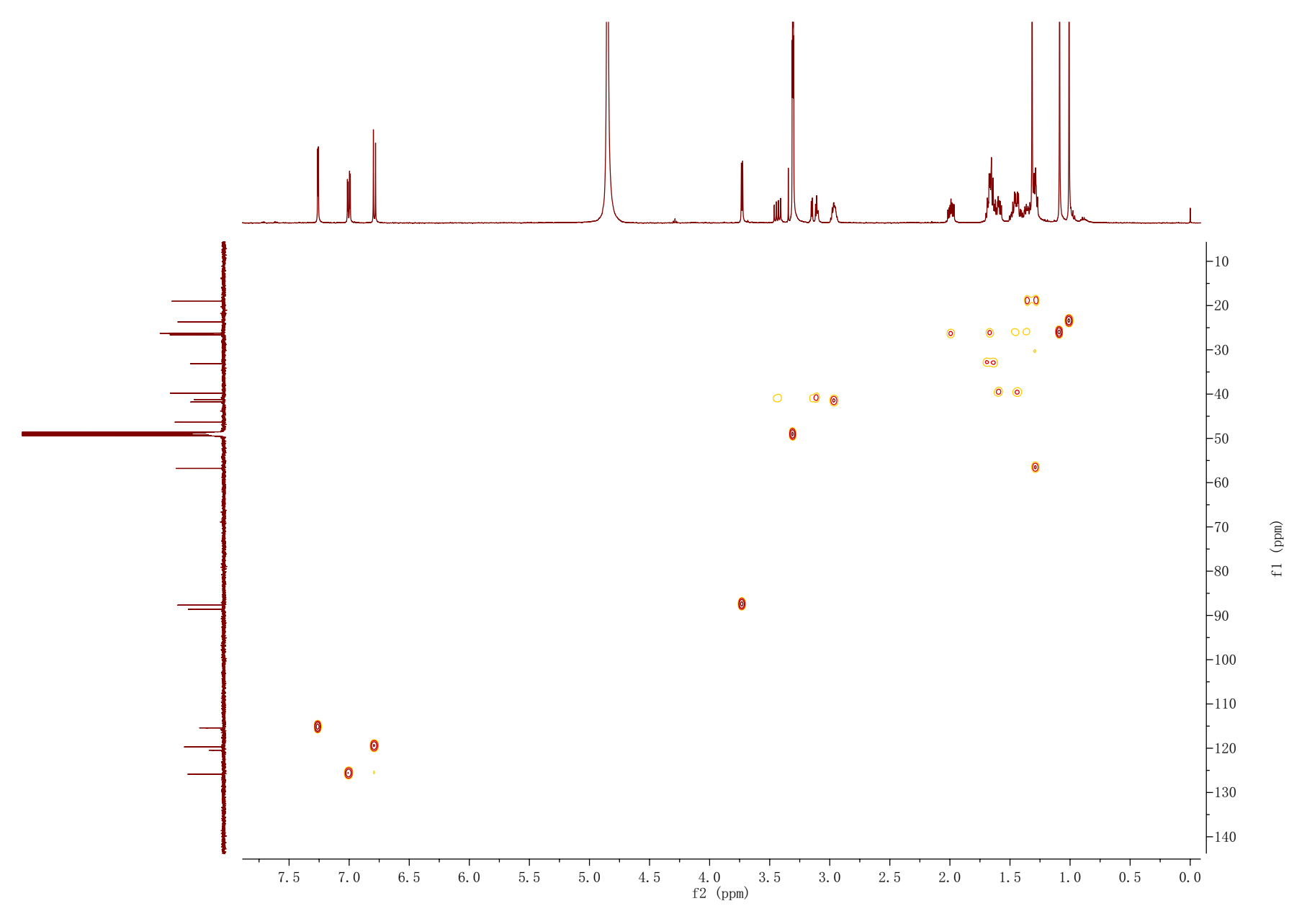


Figure S95. HSQC spectrum of **15** in methanol-*d*_4_.


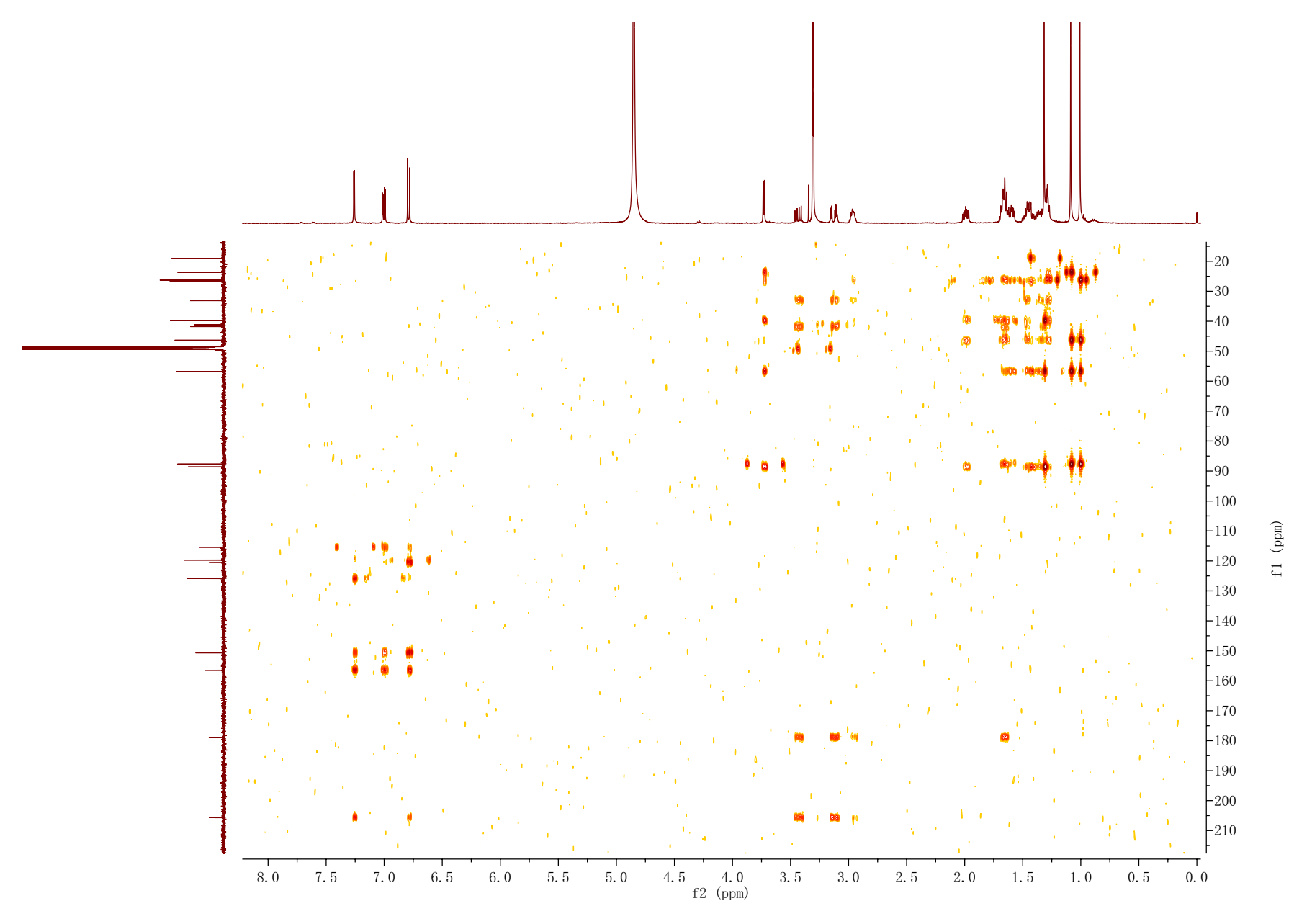


Figure S96. HMBC spectrum of **15** in methanol-*d*_4_.


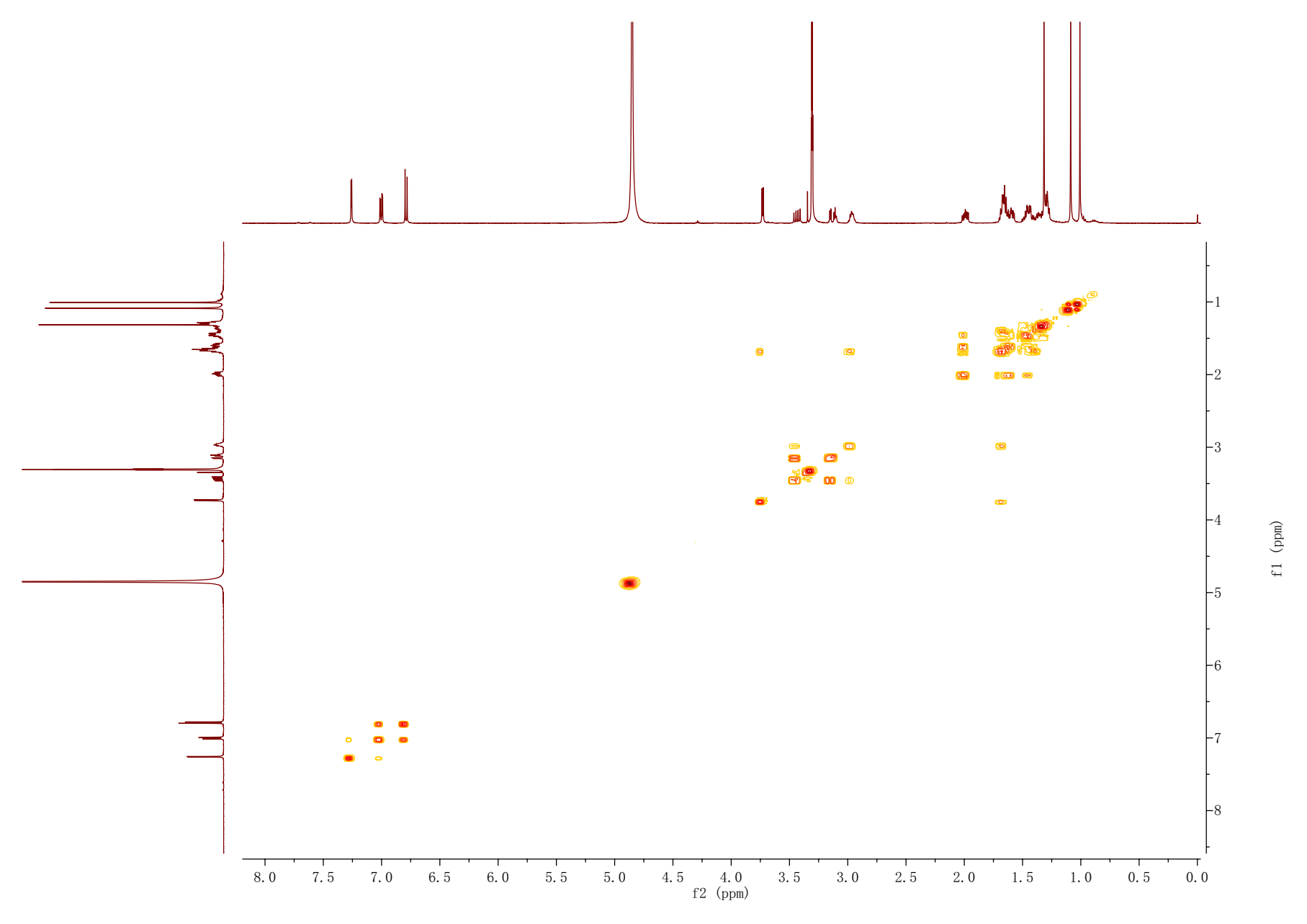


Figure S97. ^1^H-^1^H COSY spectrum of **15** in methanol-*d*_4_.


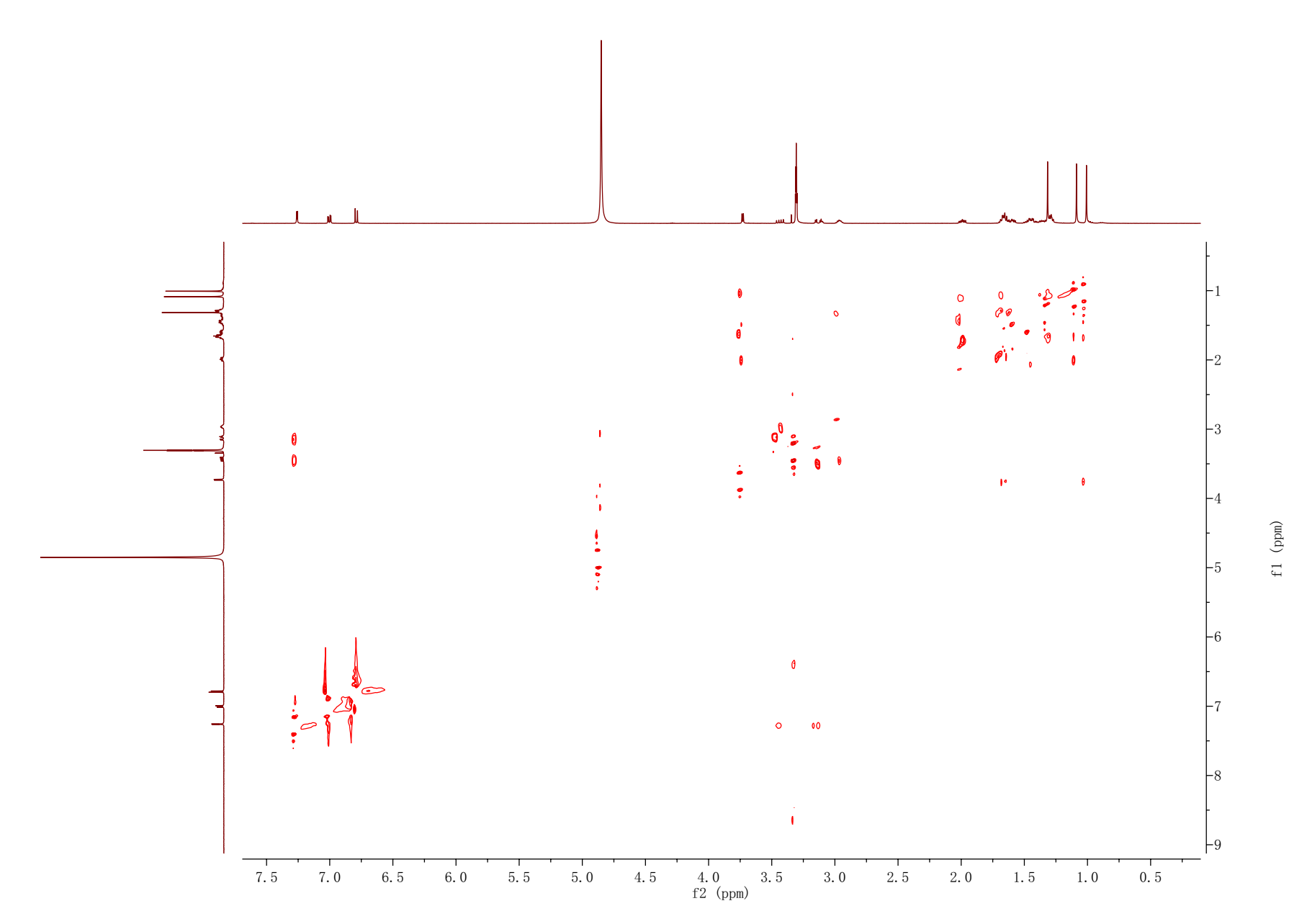


Figure S98. ROESY spectrum of **15** in methanol-*d*_4_.

[M+H]^+^ m/z 377.1968

| Hit | Formula | m/z | RDB | ppm |
| --- | --- | --- | --- | --- |
| 1 | C_21_H_29_O_6_ | 377.1959 | 8.0 | 2.5 |

Figure S99. HRESIMS of **15**.

Table S1 Crystal data and structure refinement for **15**.

| Identification code | **15** |
| --- | --- |
| Empirical formula | C_21_H_28_O_6_ |
| Formula weight | 376.43 |
| Temperature/K | 99.9(9) |
| Crystal system | monoclinic |
| Space group | P2_1_ |
| a/Å | 6.62350(10) |
| b/Å | 11.59270(10) |
| c/Å | 13.40440(10) |
| α/° | 90 |
| β/° | 100.4300(10) |
| γ/° | 90 |
| Volume/Å^3^ | 1012.240(19) |
| Z | 2 |
| ρ_calc_g/cm^3^ | 1.235 |
| μ/mm^‑1^ | 0.737 |
| F(000) | 404.0 |
| Crystal size/mm^3^ | 0.12 × 0.04 × 0.03 |
| Radiation | CuKα (λ = 1.54184) |
| 2Θ range for data collection/° | 6.704 to 148.606 |
| Index ranges | -7 ≤ h ≤ 5, -14 ≤ k ≤ 14, -16 ≤ l ≤ 16 |
| Reflections collected | 9814 |
| Independent reflections | 3973 [R_int_ = 0.0305, R_sigma_ = 0.0308] |
| Data/restraints/parameters | 3973/1/250 |
| Goodness-of-fit on F^2^ | 1.074 |
| Final R indexes [I>=2σ (I)] | R_1_ = 0.0324, wR_2_ = 0.0857 |
| Final R indexes [all data] | R_1_ = 0.0351, wR_2_ = 0.0865 |
| Largest diff. peak/hole / e Å^-3^ | 0.20/-0.20 |
| Flack parameter | -0.08(7) |

Table S2 Fractional Atomic Coordinates (×10^4^) and Equivalent Isotropic Displacement Parameters (Å^2^×10^3^) for **15**, U_eq_ is defined as 1/3 of of the trace of the orthogonalised U_IJ_ tensor.

| **Atom** | ***x*** | ***y*** | ***z*** | **U(eq)** |
| --- | --- | --- | --- | --- |
| O7 | 2343(2) | 3961.9(13) | 9189.2(10) | 26.6(3) |
| O26 | 10091(2) | 4262.9(14) | 3501.9(11) | 28.4(3) |
| O16 | 6288(3) | 7622.6(14) | 5177.5(12) | 35.9(4) |
| O13 | 8493(2) | 5001.5(15) | 4962.2(11) | 31.0(4) |
| O15 | 7844(2) | 7258.9(14) | 6768.6(11) | 32.0(4) |
| O27 | 2342(2) | 4440.4(17) | 1156.0(11) | 36.2(4) |
| C1 | 8149(3) | 4313.7(18) | 2933.0(15) | 24.0(4) |
| C2 | 6456(3) | 4650.3(18) | 3360.3(15) | 22.7(4) |
| C15' | 6568(3) | 6994.3(18) | 5909.0(15) | 24.9(4) |
| C1' | 6748(3) | 5000.0(17) | 4434.2(15) | 23.5(4) |
| C3 | 4514(3) | 4688.7(19) | 2736.2(16) | 24.9(4) |
| C2' | 4933(3) | 5346.9(19) | 4896.9(15) | 23.7(4) |
| C4 | 4272(3) | 4401.9(19) | 1719.5(15) | 26.9(4) |
| C6 | 7912(3) | 4046.6(19) | 1911.2(16) | 26.3(4) |
| C4' | 3749(3) | 5885.3(19) | 6544.2(16) | 25.9(4) |
| C10' | 713(3) | 3190(2) | 8719.3(16) | 28.2(5) |
| C3' | 5560(3) | 5833.9(18) | 5963.9(15) | 23.9(4) |
| C6' | 1552(3) | 4499.4(18) | 7470.9(15) | 24.3(4) |
| C7' | 1653(3) | 4974.8(19) | 8562.9(16) | 26.9(4) |
| C5' | 3444(3) | 4691.1(18) | 6979.0(16) | 25.6(4) |
| C5 | 5973(3) | 4087(2) | 1308.7(16) | 28.2(4) |
| C11' | 1063(4) | 3182(2) | 7617.3(17) | 29.0(5) |
| C13' | 2882(4) | 2375(2) | 7582(2) | 37.7(5) |
| C9' | -1210(4) | 3846(2) | 8874.9(19) | 37.2(6) |
| C8' | -554(4) | 5110(2) | 8759.7(18) | 35.5(5) |
| C14' | 3063(4) | 5970(2) | 8919.7(18) | 38.1(6) |
| C12' | -784(5) | 2755(3) | 6847.2(19) | 44.6(6) |

Table S3 Anisotropic Displacement Parameters (Å^2^×10^3^) for **15**.

| **The Anisotropic displacement factor exponent takes the form: -2π^2^[h^2^a*^2^U_11_+2hka*b*U_12_+…].** | | | | | | |
| --- | --- | --- | --- | --- | --- | --- |
| **Atom** | **U_11_** | **U_22_** | **U_33_** | **U_23_** | **U_13_** | **U_12_** |
| O7 | 26.1(8) | 27.4(7) | 24.9(7) | 0.4(6) | 1.0(6) | -0.7(6) |
| O26 | 20.3(7) | 34.4(8) | 29.3(7) | -0.1(7) | 0.8(6) | 5.2(6) |
| O16 | 48.1(10) | 27.0(8) | 30.4(8) | 4.4(6) | 1.0(7) | -5.6(7) |
| O13 | 22.1(7) | 40.5(9) | 28.3(7) | -2.6(7) | -0.5(6) | 2.5(7) |
| O15 | 33.2(8) | 28.1(8) | 31.5(8) | -0.5(6) | -2.6(6) | -8.9(7) |
| O27 | 22.1(8) | 61.2(12) | 24.3(7) | -5.6(7) | 1.0(6) | -0.7(7) |
| C1 | 21.0(10) | 21.1(9) | 29.0(10) | 1.9(8) | 2.1(8) | 2.0(8) |
| C2 | 21.5(10) | 21.1(9) | 25.4(9) | 2.2(8) | 4.0(8) | -1.0(8) |
| C15' | 25.4(10) | 24.3(10) | 24.8(9) | -1.5(8) | 3.8(8) | -0.1(8) |
| C1' | 21.9(10) | 21.1(9) | 26.7(10) | 0.9(8) | 2.1(8) | 0.1(8) |
| C3 | 20.7(10) | 28.0(10) | 26.5(10) | -0.2(8) | 5.8(8) | -0.7(8) |
| C2' | 21.6(9) | 24.4(10) | 24.7(9) | -0.8(8) | 3.3(7) | -1.2(8) |
| C4 | 22.1(10) | 31.7(11) | 26.0(10) | 0.2(8) | 2.1(8) | -2.0(8) |
| C6 | 23.4(10) | 25.3(10) | 31.4(10) | -0.2(8) | 7.7(8) | 1.9(8) |
| C4' | 25.9(10) | 25.5(10) | 25.9(10) | -0.9(8) | 3.9(8) | -0.7(9) |
| C10' | 25.8(11) | 28.1(11) | 30.0(11) | 2.3(9) | 3.2(8) | -5.6(9) |
| C3' | 23.8(10) | 21.9(10) | 25.1(10) | 0.3(8) | 2.1(8) | -0.4(8) |
| C6' | 23.9(10) | 24.4(10) | 23.5(9) | -0.4(8) | 1.5(8) | -1.0(8) |
| C7' | 31.1(11) | 23.8(10) | 26.0(10) | 1.1(8) | 5.7(8) | 2.1(9) |
| C5' | 27.7(10) | 23.8(10) | 25.3(9) | -1.0(8) | 4.8(8) | -0.2(8) |
| C5 | 28.5(11) | 30.8(11) | 25.5(9) | -2.1(8) | 5.2(8) | -0.5(9) |
| C11' | 31.9(12) | 24.9(10) | 30.0(11) | -1.3(8) | 5.1(9) | -6.6(9) |
| C13' | 49.3(15) | 23.8(11) | 44.2(13) | -0.8(10) | 19.7(11) | 0.2(10) |
| C9' | 26.7(12) | 48.0(15) | 38.0(12) | 4.3(11) | 9.1(9) | 0.5(10) |
| C8' | 33.5(12) | 42.1(13) | 32.2(11) | 2.4(10) | 9.8(9) | 11.5(10) |
| C14' | 54.0(15) | 32.4(12) | 28.6(11) | -8.0(9) | 9.3(10) | -9.1(12) |
| C12' | 51.8(16) | 44.5(14) | 34.2(12) | -1.3(11) | -1.3(11) | -22.3(13) |

**Table S4 Bond Lengths for 15.**

| **Atom** | **Atom** | **Length/Å** |  | **Atom** | **Atom** | **Length/Å** |
| --- | --- | --- | --- | --- | --- | --- |
| O7 | C10' | 1.455(3) |  | C4 | C5 | 1.389(3) |
| O7 | C7' | 1.467(3) |  | C6 | C5 | 1.389(3) |
| O26 | C1 | 1.372(2) |  | C4' | C3' | 1.544(3) |
| O16 | C15' | 1.209(3) |  | C4' | C5' | 1.530(3) |
| O13 | C1' | 1.241(2) |  | C10' | C11' | 1.536(3) |
| O15 | C15' | 1.336(3) |  | C10' | C9' | 1.530(3) |
| O27 | C4 | 1.363(2) |  | C6' | C7' | 1.554(3) |
| C1 | C2 | 1.404(3) |  | C6' | C5' | 1.534(3) |
| C1 | C6 | 1.385(3) |  | C6' | C11' | 1.581(3) |
| C2 | C1' | 1.474(3) |  | C7' | C8' | 1.540(3) |
| C2 | C3 | 1.402(3) |  | C7' | C14' | 1.506(3) |
| C15' | C3' | 1.509(3) |  | C11' | C13' | 1.533(3) |
| C1' | C2' | 1.504(3) |  | C11' | C12' | 1.533(3) |
| C3 | C4 | 1.384(3) |  | C9' | C8' | 1.544(4) |
| C2' | C3' | 1.524(3) |  |  |  |  |

 Table S5 Bond Angles for **15**.

| **Atom** | **Atom** | **Atom** | **Angle/˚** |  | **Atom** | **Atom** | **Atom** | **Angle/˚** |
| --- | --- | --- | --- | --- | --- | --- | --- | --- |
| C10' | O7 | C7' | 96.61(15) |  | C15' | C3' | C2' | 109.41(17) |
| O26 | C1 | C2 | 121.51(18) |  | C15' | C3' | C4' | 112.39(18) |
| O26 | C1 | C6 | 117.85(18) |  | C2' | C3' | C4' | 112.49(17) |
| C6 | C1 | C2 | 120.63(19) |  | C7' | C6' | C11' | 101.57(16) |
| C1 | C2 | C1' | 120.10(18) |  | C5' | C6' | C7' | 117.02(17) |
| C3 | C2 | C1 | 118.66(18) |  | C5' | C6' | C11' | 113.27(17) |
| C3 | C2 | C1' | 121.19(18) |  | O7 | C7' | C6' | 102.17(16) |
| O16 | C15' | O15 | 122.79(19) |  | O7 | C7' | C8' | 100.95(17) |
| O16 | C15' | C3' | 124.92(18) |  | O7 | C7' | C14' | 108.68(18) |
| O15 | C15' | C3' | 112.28(17) |  | C8' | C7' | C6' | 108.48(17) |
| O13 | C1' | C2 | 120.09(18) |  | C14' | C7' | C6' | 119.28(18) |
| O13 | C1' | C2' | 119.73(18) |  | C14' | C7' | C8' | 114.91(19) |
| C2 | C1' | C2' | 120.18(17) |  | C4' | C5' | C6' | 117.99(18) |
| C4 | C3 | C2 | 120.67(19) |  | C6 | C5 | C4 | 120.62(19) |
| C1' | C2' | C3' | 112.56(17) |  | C10' | C11' | C6' | 100.59(17) |
| O27 | C4 | C3 | 117.89(19) |  | C13' | C11' | C10' | 106.85(19) |
| O27 | C4 | C5 | 122.38(18) |  | C13' | C11' | C6' | 113.95(19) |
| C3 | C4 | C5 | 119.73(19) |  | C12' | C11' | C10' | 114.09(19) |
| C1 | C6 | C5 | 119.7(2) |  | C12' | C11' | C6' | 112.6(2) |
| C5' | C4' | C3' | 108.98(17) |  | C12' | C11' | C13' | 108.5(2) |
| O7 | C10' | C11' | 100.97(16) |  | C10' | C9' | C8' | 101.65(19) |
| O7 | C10' | C9' | 101.88(18) |  | C7' | C8' | C9' | 102.36(18) |
| C9' | C10' | C11' | 114.11(19) |  |  |  |  |  |

 Table S6 Torsion Angles for **15**.

| **A** | **B** | **C** | **D** | **Angle/˚** |  | **A** | **B** | **C** | **D** | **Angle/˚** |
| --- | --- | --- | --- | --- | --- | --- | --- | --- | --- | --- |
| O7 | C10' | C11' | C6' | -41.00(19) |  | C10' | O7 | C7' | C6' | -55.35(18) |
| O7 | C10' | C11' | C13' | 78.2(2) |  | C10' | O7 | C7' | C8' | 56.49(18) |
| O7 | C10' | C11' | C12' | -161.8(2) |  | C10' | O7 | C7' | C14' | 177.71(18) |
| O7 | C10' | C9' | C8' | 35.3(2) |  | C10' | C9' | C8' | C7' | -0.4(2) |
| O7 | C7' | C8' | C9' | -34.2(2) |  | C3' | C4' | C5' | C6' | -172.34(17) |
| O26 | C1 | C2 | C1' | 2.2(3) |  | C6' | C7' | C8' | C9' | 72.7(2) |
| O26 | C1 | C2 | C3 | 179.74(18) |  | C7' | O7 | C10' | C11' | 60.40(19) |
| O26 | C1 | C6 | C5 | 180.0(2) |  | C7' | O7 | C10' | C9' | -57.37(18) |
| O16 | C15' | C3' | C2' | 24.4(3) |  | C7' | C6' | C5' | C4' | -78.2(2) |
| O16 | C15' | C3' | C4' | -101.3(2) |  | C7' | C6' | C11' | C10' | 6.77(19) |
| O13 | C1' | C2' | C3' | 8.3(3) |  | C7' | C6' | C11' | C13' | -107.2(2) |
| O15 | C15' | C3' | C2' | -155.04(18) |  | C7' | C6' | C11' | C12' | 128.6(2) |
| O15 | C15' | C3' | C4' | 79.2(2) |  | C5' | C4' | C3' | C15' | -154.83(17) |
| O27 | C4 | C5 | C6 | -179.3(2) |  | C5' | C4' | C3' | C2' | 81.1(2) |
| C1 | C2 | C1' | O13 | 0.2(3) |  | C5' | C6' | C7' | O7 | -94.8(2) |
| C1 | C2 | C1' | C2' | -179.25(19) |  | C5' | C6' | C7' | C8' | 159.16(18) |
| C1 | C2 | C3 | C4 | -0.1(3) |  | C5' | C6' | C7' | C14' | 25.0(3) |
| C1 | C6 | C5 | C4 | 0.5(3) |  | C5' | C6' | C11' | C10' | 133.12(18) |
| C2 | C1 | C6 | C5 | -1.4(3) |  | C5' | C6' | C11' | C13' | 19.2(3) |
| C2 | C1' | C2' | C3' | -172.19(17) |  | C5' | C6' | C11' | C12' | -105.0(2) |
| C2 | C3 | C4 | O27 | 179.08(19) |  | C11' | C10' | C9' | C8' | -72.6(2) |
| C2 | C3 | C4 | C5 | -0.9(3) |  | C11' | C6' | C7' | O7 | 29.09(19) |
| C1' | C2 | C3 | C4 | 177.4(2) |  | C11' | C6' | C7' | C8' | -77.0(2) |
| C1' | C2' | C3' | C15' | 69.6(2) |  | C11' | C6' | C7' | C14' | 148.9(2) |
| C1' | C2' | C3' | C4' | -164.71(17) |  | C11' | C6' | C5' | C4' | 164.18(18) |
| C3 | C2 | C1' | O13 | -177.2(2) |  | C9' | C10' | C11' | C6' | 67.4(2) |
| C3 | C2 | C1' | C2' | 3.3(3) |  | C9' | C10' | C11' | C13' | -173.3(2) |
| C3 | C4 | C5 | C6 | 0.7(3) |  | C9' | C10' | C11' | C12' | -53.4(3) |
| C6 | C1 | C2 | C1' | -176.30(19) |  | C14' | C7' | C8' | C9' | -150.9(2) |
| C6 | C1 | C2 | C3 | 1.2(3) |  |  |  |  |  |  |

 Table S7 Hydrogen Atom Coordinates (Å×10^4^) and Isotropic Displacement Parameters (Å^2^×10^3^) for **15.**

| **Atom** | ***x*** | ***y*** | ***z*** | **U(eq)** |
| --- | --- | --- | --- | --- |
| H26 | 10039 | 4393 | 4098 | 43 |
| H15 | 8390 | 7883 | 6704 | 48 |
| H27 | 2389 | 4287 | 563 | 54 |
| H3 | 3378 | 4909 | 3008 | 30 |
| H2'A | 4059 | 4680 | 4921 | 28 |
| H2'B | 4141 | 5922 | 4469 | 28 |
| H6 | 9045 | 3841 | 1630 | 32 |
| H4'A | 4037 | 6445 | 7089 | 31 |
| H4'B | 2509 | 6122 | 6089 | 31 |
| H10' | 813 | 2420 | 9027 | 34 |
| H3' | 6593 | 5314 | 6339 | 29 |
| H6' | 364 | 4848 | 7032 | 29 |
| H5'A | 4652 | 4515 | 7482 | 31 |
| H5'B | 3392 | 4134 | 6435 | 31 |
| H5 | 5811 | 3902 | 624 | 34 |
| H13A | 4056 | 2636 | 8056 | 57 |
| H13B | 3197 | 2376 | 6910 | 57 |
| H13C | 2533 | 1606 | 7756 | 57 |
| H9'A | -1527 | 3703 | 9543 | 45 |
| H9'B | -2388 | 3642 | 8364 | 45 |
| H8'A | -1430 | 5484 | 8194 | 43 |
| H8'B | -583 | 5548 | 9373 | 43 |
| H14A | 3095 | 6096 | 9630 | 57 |
| H14B | 2570 | 6652 | 8548 | 57 |
| H14C | 4421 | 5795 | 8808 | 57 |
| H12A | -1153 | 1993 | 7030 | 67 |
| H12B | -433 | 2738 | 6183 | 67 |
| H12C | -1924 | 3267 | 6846 | 67 |

**Experimental**

Single crystals of C_21_H_28_O_6_. A suitable crystal was selected and  on a **XtaLAB AFC12 (RINC): Kappa single** diffractometer. The crystal was kept at 99.9(9) K during data collection. Using Olex2 [1], the structure was solved with the ShelXT [2] structure solution program using Intrinsic Phasing and refined with the ShelXL [3] refinement package using Least Squares minimisation.

1. Dolomanov, O.V., Bourhis, L.J., Gildea, R.J, Howard, J.A.K. & Puschmann, H. (2009), J. Appl. Cryst. 42, 339-341.
2. Sheldrick, G.M. (2015). Acta Cryst. A71, 3-8.
3. Sheldrick, G.M. (2015). Acta Cryst. C71, 3-8.

**Crystal structure determination of 15**

**Crystal Data** for C_21_H_28_O_6_ (*M*=376.43 g/mol): monoclinic, space group P2_1_ (no. 4), *a* = 6.62350(10) Å, *b* = 11.59270(10) Å, *c* = 13.40440(10) Å, *β* = 100.4300(10)°, *V*= 1012.240(19) Å^3^, *Z* = 2, *T* = 99.9(9) K, μ(CuKα) = 0.737 mm^-1^, *Dcalc* = 1.235 g/cm^3^, 9814 reflections measured (6.704° ≤ 2Θ ≤ 148.606°), 3973 unique (*R*_int_ = 0.0305, R_sigma_ = 0.0308) which were used in all calculations. The final *R*_1_ was 0.0324 (I > 2σ(I)) and *wR*_2_ was 0.0865 (all data).

**ECD calculations** The conformation search based on molecular mechanics with MMFF force fields. The low energy conformers were optimized at the B3LYP/6-31G(d,p) level using DFT/TDDFT method in the Gaussian 09 software package.^[1]^ ECD calculations were conducted at the B3LYP/6-31G(d,p) and CAM-B3LYP/def2SVP level in MeOH solution. The program SpecDis was used for comparison of the calculated curves and experimental CD spectra.

**^1^H and ^13^C NMR calculation of 12**

Conformation search and geometric optimization were adopted the same method as ECD calculations in the Gaussian 09 software package.^[1]^ Gauge-Independent Atomic Orbital (GIAO) calculations of NMR chemical shifts were submitted in Gaussian 09 by density functional theory (DFT) with the level of B3LYP/6-311G(d,p) in methanol with the PCM solvent model. The calculated NMR chemical shifts were analyzed by subtracting the isotopic shifts for TMS calculated with the same methods.^[2]^ Regression analysis of calculated versus experimental ^13^C NMR chemical shifts of **12** were carried out. Linear correlation coefficients (*R*^2^), mean absolute error (MAE), and corrected mean absolute error (CMAE) were calculated for the evaluation of the results. After Boltzmann weighing of the predicted chemical shift of each isomers, the DP4+ parameters were calculated using the excel file provided by Ariel M. Sarotti.^[3]^

Figure S100. Correlation plots of experimental and calculated ^13^C-NMR chemical shifts for **12.**

**Table S14.** Linear correlation coefficients (*R*^2^), mean absolute error (MAE) and corrected mean absolute error (CMAE) analyses of the calculated and experimental NMR data.

| Canditate configurations | *R*^2^ | MAE | CMAE |
| --- | --- | --- | --- |
| **12a** | 0.9969 | 6.01 | 2.27 |
| **12b** | 0.9907 | 6.29 | 4.05 |


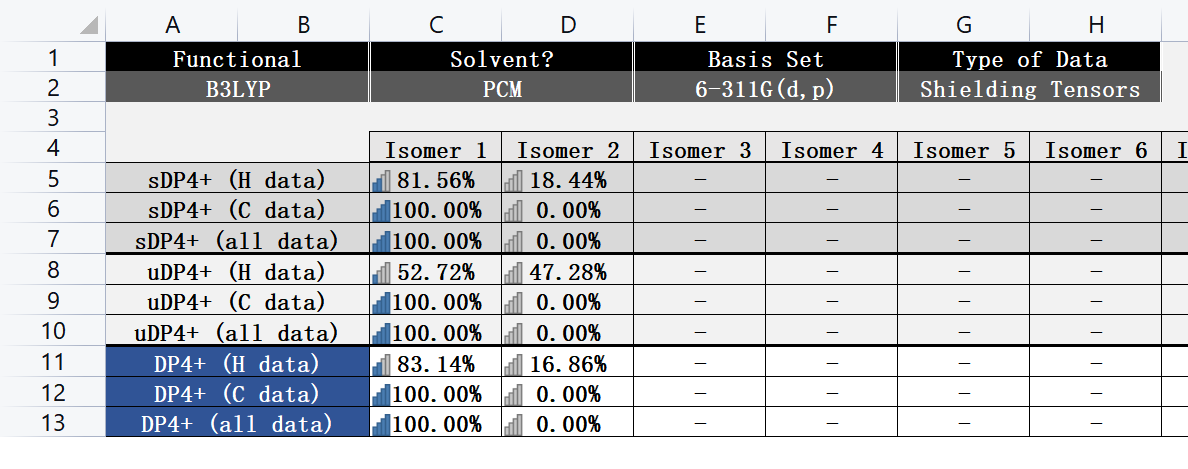


Figure 101. DP4+ results of candidate **12a** (Isomer **1**) and **12** (Isomer **2**).

**Specific optical rotation data (ORD) calculations of 12** Conformation search and geometric optimization adopt the same method as ECD calculations. The specific optical rotation calculations were performed at the B3LYP/6-311+G(2d,p) level in MeOH with PCM model. Finally, the calculated specific ORD of ten conformers were averaged according to the Boltzmann distribution theory and their relative Gibbs free energy (∆G).

Table S8 Energy analysis for conformers of **12a-1**-**12a-10** for the calculations at the B3LYP/6-311+G(2d,p) level in methanol.

| Conformers | *G* | ∆*G* (cal/mol) | PE% | [α]_D_ |
| --- | --- | --- | --- | --- |
| **12a-1** | -1266.905494 | 6.27509E-4 | 16.1% | -249.89 |
| **12a-2** | -1266.904919 | 0.36145 | 8.7% | -126.74 |
| **12a-3** | -1266.905495 | 0 | 16.1% | -249.38 |
| **12a-4** | -1266.904738 | 0.47502 | 7.2% | -13.95 |
| **12a-5** | -1266.905123 | 0.23343 | 10.9% | +495.73 |
| **12a-6** | -1266.904913 | 0.36521 | 8.7% | -126.74 |
| **12a-7** | -1266.905120 | 0.23532 | 10.8% | +224.36 |
| **12a-8** | -1266.905395 | 0.06275 | 14.5% | +346.03 |
| **12a-9** | -1266.902657 | 1.78087 | 0.8% | +418.19 |
| **12a-10** | -1266.904591 | 0.56727 | 6.2% | -99.40 |
| **Total** [α]_D_ |  |  |  | +22.18 |

References:

[1] Frisch, M. J.; Trucks, G. W.; Schlegel, H. B.; Scuseria, G. E.; Robb, M. A.; Cheeseman, J. R.; Scalmani, G.; Barone, V.; Mennucci, B.; Petersson, G. A.; Nakatsuji, H.; Caricato, M.; Li, X.; Hratchian, H. P.; Izmaylov, A. F.; Bloino, J.; Zheng, G.; Sonnenberg, J. L.; Hada, M.; Ehara, M.; Toyota, K.; Fukuda, R.; Hasegawa, J.; Ishida, M.; Nakajima, T.; Honda, Y.; Kitao, O.; Nakai, H.; Vreven, T.; Montgomery, J. A.; Peralta, J. E.; Ogliaro, F.; Bearpark, M.; Heyd, J. J.; Brothers, E.; Kudin, K. N.; Staroverov, V. N.; Keith, T.; Kobayashi, R.; Normand, J.; Raghavachari, K.; Rendell, A.; Burant, J. C.; Iyengar, S. S.; Tomasi, J.; Cossi, M.; Rega, N.; Millam, J. M.; Klene, M.; Knox, J. E.; Cross, J. B.; Bakken, V.; Adamo, C.; Jaramillo, J.; Gomperts, R.; Stratmann, R. E.; Yazyev, O.; Austin, A. J.; Cammi, R.; Pomelli, C.; Ochterski, J. W.; Martin, R. L.; Morokuma, K.; Zakrzewski, V. G.; Voth, G. A.; Salvador, P.; Dannenberg, J. J.; Dapprich, S.; Daniels, A. D.; Farkas, O.; Foresman, J. B.; Ortiz, J. V.; Cioslowski, J.; Fox, D. J. Gaussian 09, revision C.01. Gaussian, Inc.: Wallingford CT, 2010.

[2] Lodewyk, M. W.; Siebert, M. R.; Tantillo, D. J.; *Chem. Rev.* **2012**, *112*, 1839–1862.

[3] Grimblat, N.; Zanardi, M. M.; Sarotti, A. M.; *J. Org. Chem.* **2015**, *80*, 12526−12534.
